# *In silico* analysis of *ACE2* from different animal species provides new insights into SARS-CoV-2 species spillover

**DOI:** 10.2217/fvl-2022-0187

**Published:** 2023-04-11

**Authors:** Felipe Pantoja Mesquita, Pedro Filho Noronha Souza, Dyane Rocha Aragão, Expedito Maia Diógenes, Emerson Lucena da Silva, Jackson Lima Amaral, Valder Nogueira Freire, Débora de Souza Collares Maia Castelo-Branco, Raquel Carvalho Montenegro

**Affiliations:** ^1^Laboratory of Pharmacogenetics, Drug Research & Development Center (N.P.D.M.), Federal University of Ceará, Fortaleza, 60430-2752, Brazil; ^2^Department of Biochemistry & Molecular Biology, Federal University of Ceará, Fortaleza, 60440-9003, Brazil; ^3^Specialized Medical Mycology Center, Group of Applied Medical Microbiology, Federal University of Ceará, Fortaleza, 60430-1404, Brazil; ^4^Department of Physics, Federal University of Ceará, Fortaleza, 60440-900, Brazil

**Keywords:** ACE2 interactions, intermediate host, SARS-CoV-2, spike protein

## Abstract

**Aim:** This study aimed to analyze the phylogenetic relationships between the ACE2 of humans and other animals and investigate the potential interaction between SARS-CoV-2 RBD and ACE2 of different species. **Materials & methods:** The phylogenetic construction and molecular interactions were assessed using computational models. **Results & conclusion:** Despite the evolutionary distance, 11 species had a perfect fit for the interaction between their ACE2 and SARS-CoV-2 RBD (*Chinchilla lanigera, Neovison vison*, *Rhinolophus sinicus, Emballonura alecto*, *Saccopteryx bilineata*, *Numida meleagris*). Among them, the avian *N. meleagris* was reported for the first time in this study as a probable SARS-CoV-2 host due to the strong molecular interactions. Therefore, predicting potential hosts for SARS-CoV-2 for understanding the epidemiological cycle and proposal of surveillance strategies.

## Plain language summary

Here, computational analysis was employed to predict the interaction between the Spike protein from SARS-COV-2 with the ACE2 receptor with animals that could serve as a reservoir for SARS-CoV-2 spillover. Our results reported for the first time that *N. meleagris* could act as a possible host for SARS-CoV-2.

COVID-19, a novel infectious disease caused by SARS-CoV-2, was first detected in China in late 2019, becoming a pandemic by March 2020 [1]. SARS-CoV-2 belongs to the betacoronavirus genus, similar to other coronaviruses, such as SARS-CoV-1 and MERS-CoV, which have been associated with human outbreaks in the past two decades [2]. Despite being less pathogenic than SARS-CoV-1 and MERS-CoV [3], SARS-CoV-2 is highly transmissible to humans, which significantly contributed to the rapid development of the COVID-19 pandemic [4].

Effective SARS-CoV-2 cell entry is the first required step for viral infection. This virus enters target cells by binding its spike (S) protein to ACE-2 receptors. During SARS-CoV-2 infection in mammalian cells, two S protein domains are involved: the S1 domain interacts with the ACE2 receptor, and the S2 domain undergoes structural rearrangements to promote membrane fusion [5]. ACE2 is a widespread receptor in vertebrates, which potentially warrants many host species for this virus, as the affinity between viral S protein and ACE-2 determines the SARS-CoV-2 host range.

The most accepted origin of COVID-19 is the spillover of SARS-CoV-2 from animals to humans. Comparing the SARS-CoV-2 genome with other coronavirus sequences shows that SARS-CoV-2 originated from bats [6]. However, a highly similar virus has been detected in Malayan pangolins (*Manis javanica*), suggesting that these animals may have acted as an intermediate host before infecting humans [7]. Hence, based on the wide distribution of ACE2 receptors in vertebrates and the detection of SARS-CoV-2-related viruses in different animal species (bats and pangolin), it is believed that this virus has a much broader host range [8].

In this context, SARS-CoV-2 infection in humans has been followed by naturally acquired infections in various species across the world, such as cats (*Felis catus*) and dogs (*Canis lupus familiaris*) [9–11], captive tigers (*Panthera tigris*), lions (*Panthera leo*), snow leopard (*Panthera uncia*) and puma (*Puma concolor*) from zoos [12], ferrets (*Mustela putorius furo*) [13], in farmed and wild American mink (*Neovison vison*) [14] and captive and wild white-tailed deers (*Odocoileus virginianus*) [15].

It is known that the analysis of ACE2 protein sequence across different animal species may aid in the prediction of their susceptibility to SARS-CoV-2 infection [16]. These analyses may help predict the host range for SARS-CoV-2 and the most vulnerable animal species to viral infection, which would aid in understanding the viral epidemiological cycle and establishing surveillance strategies to control the disease. Thus, the goal of the current study was to analyze the phylogenetic relationships between the ACE2 of humans and other animals and investigate the potential interaction between SARS-CoV-2 RBD and ACE2 of different species, to understand better how the virus spreads across species.

## Methods

### Download of sequences, alignment & construction of three-dimensional (3D) models

The fast files of ACE2 sequences of human (*Homo sapiens*) and animals used in this study were downloaded from the freely accessible database of the National Center for Biotechnology Information (NCBI, www.ncbi.nlm.nih.gov/). The alignment of sequences was performed using the Clustal Omega (www.ebi.ac.uk/Tools/msa/clustalo/).

The 3D models of the ACE2 of different animal species were constructed by homology modeling in the SWISS-MODEL server (https://swissmodel.expasy.org/interactive), using as model human ACE2 (PDB: 1R42) downloaded from Protein Data Bank (PDB, www.rcsb.org/). The Macromolecular x-Ray Crystallography Software was used to adjust the Phi and Psi angles of the structural models. The Molprobity (http://molprobity.biochem.duke.edu/) was employed to evaluate the quality of the predicted models by Ramachandran plot analysis. The ERRAT2 (https://yeateslab.mbi.ucla.edu/structure-validation/) server was employed to validate the protein structures.

### Phylogenetic & similarity analysis

ACE2 protein-coding sequences (CDSs) of human and 50 different animal species were downloaded from the NCBI databank (www.ncbi.nlm.nih.gov/gene/). Afterward, the sequences were aligned and converted into phylip file format using MAFFT v.7 [17]. Phylogenetic analysis was done using the MEGA X, applying the Maximum likelihood method (ML) [18]. The bootstrap consensus tree inferred from 1000 replicates based on general time reversible (GTR) substitution model with gamma distribution was applied. In parallel, pairwise nucleotide p-distances were calculated. The phylogenetic trees were visualized by FigTree v1.4.4 (http://tree.bio.ed.ac.uk/software/figtree/). SimPlot 3.5.1 was used to analyze the ACE2 protein coding sequences' identity, comparing humans with other animals, using the Kimura (2-parameter) method [19].

### Molecular docking assays

The complex formed between the SARS-CoV-2 S protein and the human ACE2 protein was obtained from the Protein Data Bank with accession number 6M0J [20]. The S protein used in this study was from the wildtype SARS-CoV-2 strain that emerged in Wuhan, China, because it has been well-characterized in complex with ACE2 by x-ray crystallography and Cryo-EM. These physical studies have provided considerable data on the structure of the Spike protein [20]. The protein S and ACE2 protein were separated using Discovery Studio version 3.1, and the separate files were used as inputs to perform the redocking to validate the applied docking methodology. The redocking was performed in FRODOCK 2.0 (http://frodock.chaconlab.org/) [21].

After validating the molecular docking methodology, the ACE2 proteins of the 50 animal species modeled according to section 2.1 and the SARS-CoV-2 S protein obtained from PDB 6M0J were used as docking inputs. All 49 dockings were performed in FRODOCK 2.0, and the resulting complexes, formed by SARS-CoV-2 S protein and animal ACE2, similar to the one formed by the human ACE2 protein, were used to carry out the following methodologies.

### Energy minimization & equilibration

To minimize the energies and balance the pressure and temperature of the complexes obtained in the molecular docking, Gromacs 2021.2 was used [22]. The topology was recorded using the OPLS-AA/L all-atom force field [23]. A cubic box was created with a 2 nm edge, and the SPC/E water model was used to solvate the box. Thus, Na^+^ and Cl^-^ ions were added to the water box's final concentration of 0.15 M. The minimization was carried out until the potential energy was negative and the maximum force was less than 1000 kJ mol^-1^ nm^-1^. Next, temperature and pressure equilibration were performed for 100 ps. The final structures were visualized using the V.M.D. software, and the stable complexes were used for further analyses.

### Interaction of complex analysis

The Protein Interactions Calculator (PIC) webserver (http://pic.mbu.iisc.ernet.in/) [24] was used to analyze the interactions and Ligplot software to analyze the hydrogen bonds and the hydrophobic interactions [25]. The PyMol (https://pymol.org/2/) software was used to generate the figures with the 3D structures and to calculate the RMSD between SARS-CoV-2 S protein and human ACE2 and ACE2 of other animal species.

### Quantum Biochemistry

A quantum biochemistry study was aimed to analyze the main residues involved in the protein interactions and the total energy of interaction between the S protein of SARS-CoV-2 and the ACE2 protein of each animal species carried out. The quantum biochemistry calculation was performed according to a previously established methodology [26–28]. Briefly, all amino acid residues up to 8 Å apart between SARS-CoV-2 protein S and animal ACE2 proteins were analyzed, and molecular fractionation with conjugate caps (MFCC) was performed to analyze the energy of interaction between pairs of amino acid residues.

The interaction energy between two specific residues (R_i_ and R_j_) was calculated as follows:

$$\begin{array}{l} {\text{E}}_{\text{I}}({\text{R}}_{\text{i}}-{\text{R}}_{\text{j}})=\text{E}({\text{C}}_{\text{i}-1}\;{\text{R}}_{\text{i}}\;{\text{C}}_{\text{i}+1}\;{\text{C}}_{\text{j}-1}\;{\text{R}}_{\text{j}}\;{\text{C}}_{\text{j}+1})-\text{E}({\text{C}}_{\text{i}-1}\;{\text{R}}_{\text{i}}\;{\text{C}}_{\text{i}+1}\;{\text{C}}_{\text{j}-1}\;{\text{C}}_{\text{j}+1})-\\ \text{E}({\text{C}}_{\text{i}-1}\;{\text{C}}_{\text{i}+1}\;{\text{C}}_{\text{j}-1}\;{\text{R}}_{\text{j}}\;{\text{C}}_{\text{j}+1})+\text{E}({\text{C}}_{\text{i}-1}\;{\text{C}}_{\text{i}+1}\;{\text{C}}_{\text{j}-1}\;{\text{C}}_{\text{j}+1}) \end{array}$$

Where the Ck terms refer to the conjugate caps, which are the residues covalently bound to Rk. A hydrogen atom was added to complete the valence in the cut peptide bonds. At the right-hand side of the equation, the first term, E(C_i-1_ R_i_ C_i + 1_ C_j-1_ R_j_ C_j + 1_), is the system's total energy formed by two interacting residues with their caps. The second term, E(C_i-1_ R_i_ C_i + 1_ C_j-1_ C_j + 1_), gives the system total energy formed by the capped residue R_i_ without the residue R_j_. The third term, E(C_i-1_ C_i + 1_ C_j-1_ R_j_ C_j + 1_), is the system's total energy formed by R^j^ and without the residue R_i_. Finally, E(C_i-1_ C_i + 1_ C_j-1_ C_j + 1_) is the system's total energy formed only by the caps.

The DMOL3 code [29] was used to do the density functional theory calculations, and a Double Numerical plus Polarization (D.N.P.) basis was selected to extend the Kohn-Sham orbitals for all electrons. The Perdew–Burke–Ernzerhof (PBE) parameterization with the corrected dispersion energy proposed by Tkatchenko and Scheffler [30] (GGA + TS) was used as the theoretical calculation level with the generalized gradient approximation (GGA), which partially accounts for the relative variation in dispersion parameters of differently bonded atoms. Self-consistent field (SCF) convergence required a total energy variation of 106 Ha. A dielectric function of 40 was used, and an implicit system solvation simulation was used. A distance of up to 2.5 between explicit water molecules was also taken into account.

## Results

### Phylogenetic distance & sequence similarity

**Figure 1.**
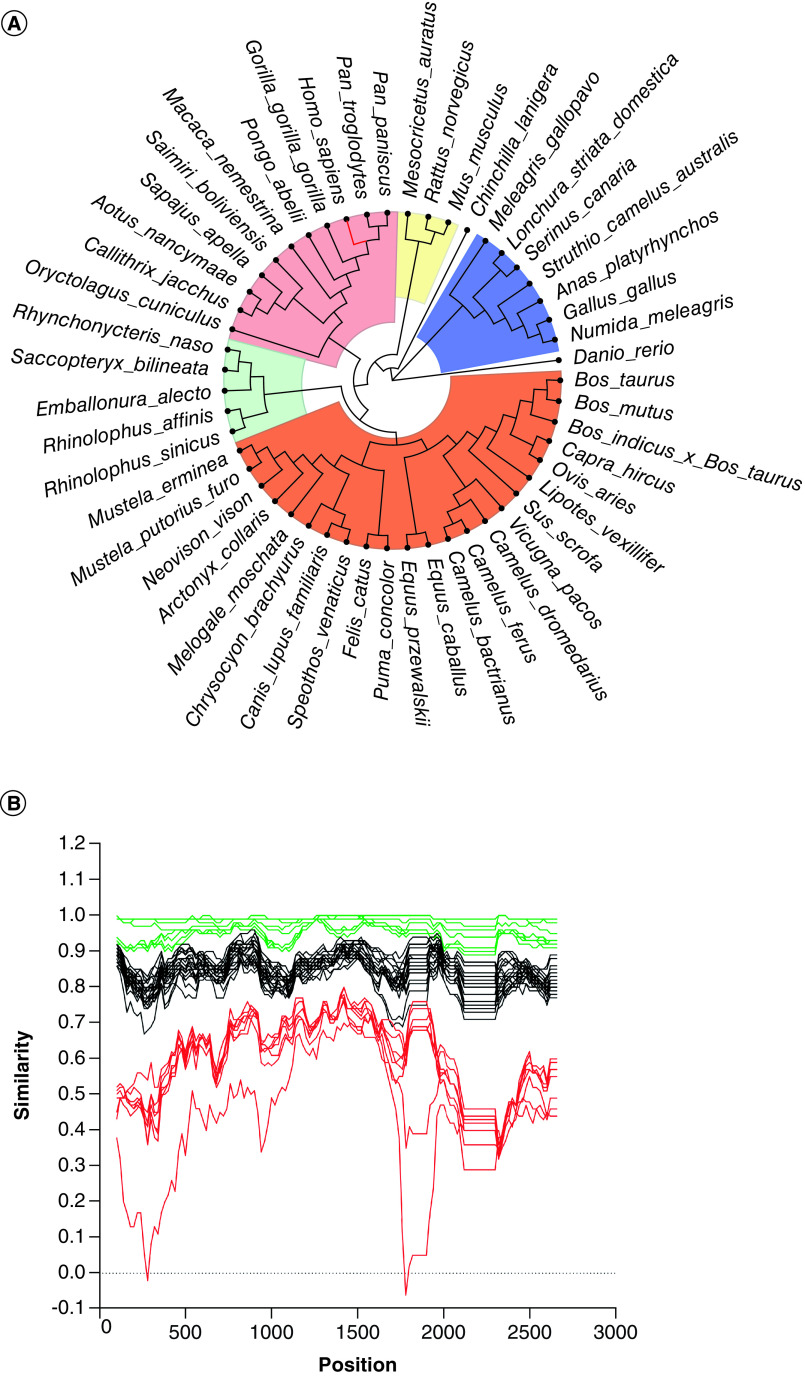

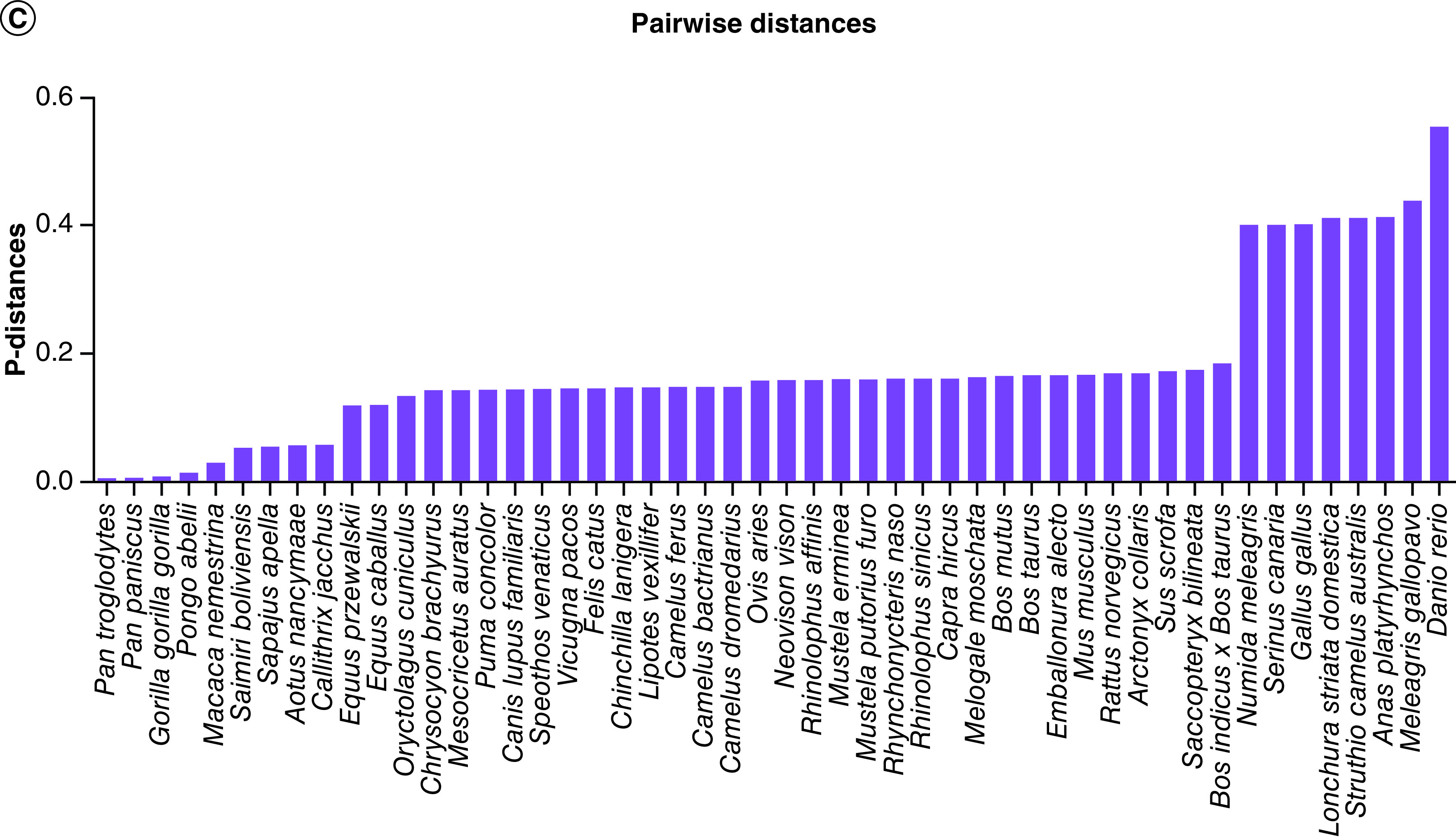
ACE2 nucleotide sequence similarity and evolutionary distance of studied species. **(A)** ACE2 and its homologues from representative species wereanalyzed phylogenetically. Based on alignments of the nucleotide sequence of ACEs, a phylogenetic tree was created using the maximum-likelihood approach and MEGA X software. **(B)** Similarity plot of whole nucleotide sequence of *ACE2* gene among animals’ species, including H. sapiens. Similarity plot was performed using the Kimura (2-parameter) method. **(C)** Pairwise nucleotide p-distances of *ACE2* gene among animals’ species.

To investigate the evolution and similarity aspects of *ACE2* in humans and other animals, a phylogenetic tree was built to cluster the *ACE2s* based on their evolutionary distance. Based on 2,779 nucleotide sequences from the *ACE2* gene, a maximum likelihood phylogenetic analysis was inferred (Figure 1a). The phylogenetic tree formed five distinct major clades, demonstrating topology conserved with *H. sapiens* closer to other primates in a monophyletic group, especially the clade with the closest species, *Pan troglodytes* and *P. paniscus*. Bats, rodents, carnivores, ungulates, and birds were clustered into different branches, with the avian clade as the most divergent from humans. The nucleotide sequence of *ACE2* in *Danio rerio* is phylogenetically further away from the human, showing that SARS-CoV-2 is unlikely to infect.

Sequence similarity and distance between *ACE2* of a broad range of animal species and human *ACE2* were also analyzed. The percentages of nucleotide sequence similarity were calculated from the alignment of the *ACE2* gene of the selected species (Figure 1B). Humans share high sequence similarity with a median of 94.4% (CI 95%: 93.9 – 99.3) with other primates (green lines, Figure 1B). Human *ACE2* showed a low degree of similarity (median = 59.7%; CI 95%: 45.7–60.8; red lines, Figure 1B) with *ACE2* sequences of birds and fish (*D. rerio*) and intermediate similarity with the other studied species (median = 84.4%; CI 95%: 83.7–85.5; black lines, Figure 1B). Genetic distances, based on the *ACE2* gene, among human and other animal species were estimated using an implemented p-distance method. Genetic inference calculations confirmed the divergence of *ACE2* sequences between *H. sapiens* and avian species and the fish *D. rerio* (Figure 1C).

### SARS-CoV-2 RBD interacts with ACE2 of animals

**Figure 2.**
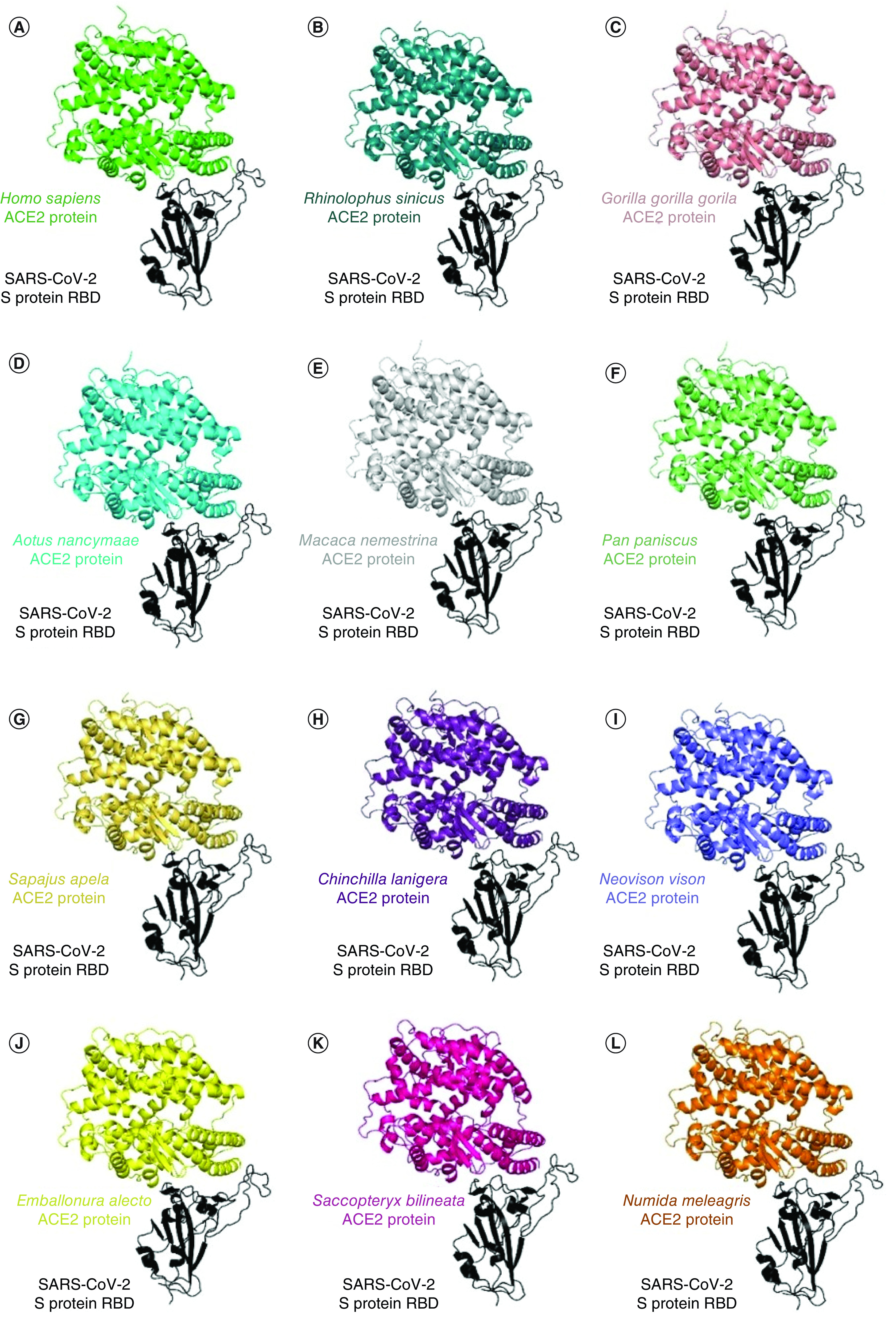
Three-dimensional structure and protein–protein interaction of angiotensin-converting enzyme 2 (ACE2) with wild-type receptor-binding domain in the best fit animals. Eleven species whose ACE2 interacted with viral RBD in a similar manner as observed for human ACE2-RBD interaction. **(A)** *H. sapiens*, **(B)** *R. sinicus*, **(C)** *G. gorilla*, **(D)** *A. nancymaae*, **(E)** *M. nemestrina*, **(F)** *P. paniscus*, **(G)** *S. apella*, **(H)** *C. lanigera*, **(I)** *N. vison*, **(J)** *E. alecto*, **(K)** *S. bilineata* and **(L)** *N. meleagris*. RBD: Receptor-binding domain.

**Figure 3.**
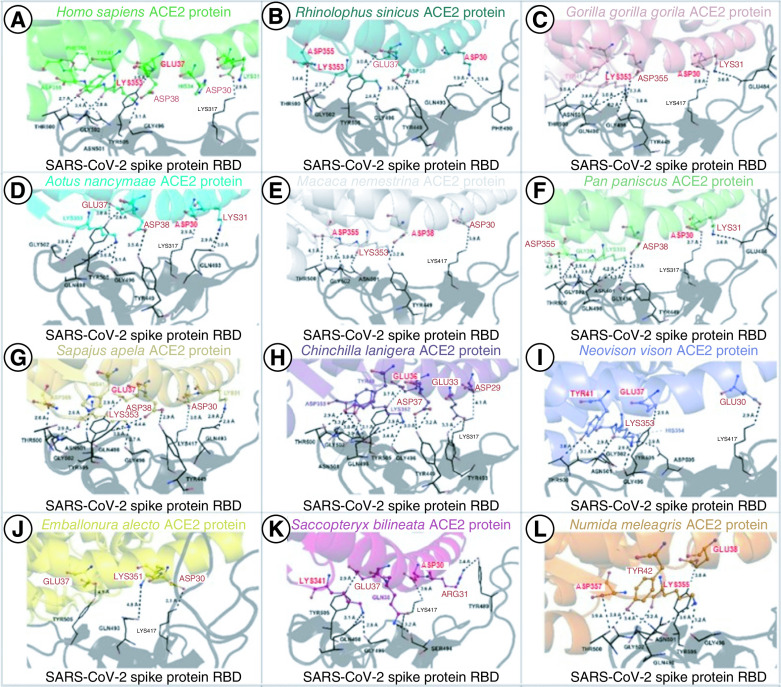
Residues at the binding interface between ACE2 and RBD. Interactions at critical binding sites are shownfor the residues found in **(A)** *H. sapiens*, **(B)** *R. sinicus*, **(C)** *G. gorilla*, **(D)** *A. nancymaae*, **(E)** *M. nemestrina*, **(F)** *P. paniscus*, **(G)** *S. apella*, **(H)** *C. lanigera*, **(I)** *N. vison*, **(J)** *E. alecto*, **(K)** *S. bilineata* and **(L)** *N. meleagris*. The main residues of ACE2 protein from human and other animals are highlighted in red.

Molecular dockings between the RBD of SARS-CoV-2 S protein with the *ACE2* proteins of 50 different species revealed that 11 had a perfect fit for the interaction between their *ACE2* and viral RBD (same configuration and position) when compared with the human ACE2:RBD interaction (Figure 2A). The species whose ACE2 similarly interacted with viral RBD as observed for human ACE2-RBD interaction were: *Rhinolophus sinicus*, *Gorilla gorilla gorilla*, *Aotus nancymaae*, *Macaca nemestrina*, *Pan paniscus*, *Sapajus apella*, *Chinchilla lanigera*, *Neovison vison*, *Emballonura alecto*, *Saccopteryx bilineata*, and *Numida meleagris* (Figure 2B–L). For a better picture, residue-by-residue analyses of these interactions were performed, with the respective energy contribution (Figure 3 and Supplementary Tables).

Human ACE2 established 4 hydrophobic interactions, 19 hydrogen bonds, three ionic interactions, two aromatic-aromatic and one aromatic-sulfur interaction with the RBD of the SARS-CoV-2 S protein (Figure 3A, Supplementary Table 1). The most important interactions observed were between Asp^30^, Glu^37^, Lys^353^, and Asp^38^ of human ACE2 and residues Lys^417^, Tyr^505^, Asn^501^ and Tyr^449^ of viral RBD, with interaction energies of -10.93, -9.36, -7.99 and -7.07 kcal.mol^-1^, respectively (Figure 3A, Supplementary Table 2). Human ACE2-viral RBD interactions were used as a control for the comparative analysis of animal ACE2-SARS-CoV-2 RBD interactions. The interactions of residues between the other ACE2 animals with SARS-CoV-2 RBD and the human ACE2 with SARS-CoV-2 RBD varied significantly. In Figure 3, the main ACE2 residues involved in the interaction with Spike are highlighted in red, and all residues' interaction energy are presented in the Supplementary Tables.

### Global energy of interactions between ACE2 of different animal species & SARS-CoV-2 RBD

**Figure 4.**
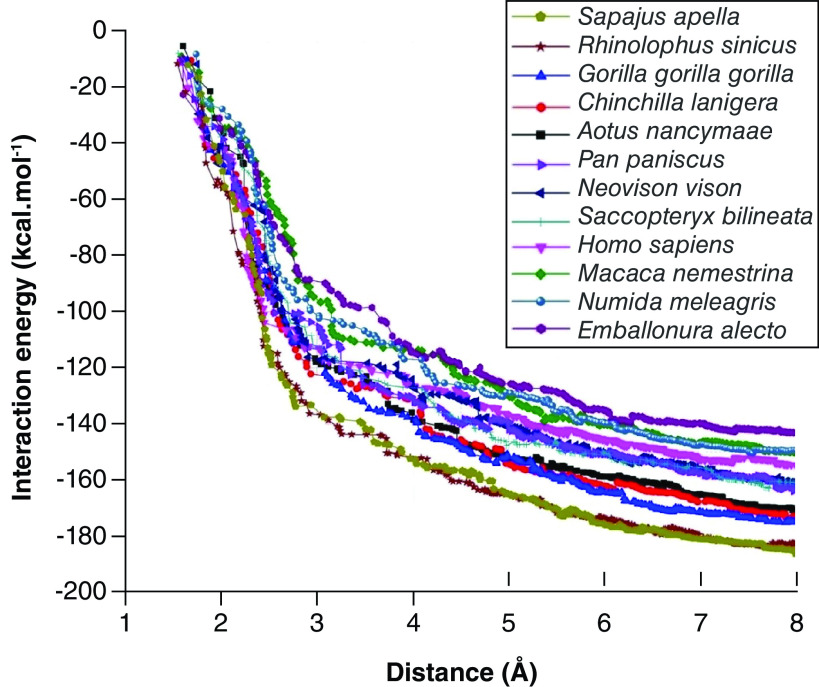
SARS-CoV-2 RBD and ACE2 total interaction energy as a function of interaction and distance.

The greatest variations in the interaction energies between animal ACE2 and SARS-CoV-2 protein S RBD were observed at a distance of up to 6 Å, with an energy convergence indicating that amino acid residues with a distance greater than 8 Å will contribute less to the interaction energy. The values of the total energy of interaction between animal ACE2 and viral RBD were -186.26, -182.58, -175.07, -172.85, -171.00, -163.84, -161.23, -160.82, -154.71, -150.11, -149.56 and -143.19 kcal.mol^-1^ for *S. apella*, *R. sinicus*, *G. gorilla gorilla*, *C. lanigera*, *A. nancymaae*, *P. paniscus*, *N. vison*, *S. bilineata*, *H. sapiens*, *M. nemestrina*, *N. meleagris* and *E. allecto*, respectively (Figure 4).

Eight of the 11 evaluated animals presented higher energy of interaction between *ACE2* and viral RBD than that observed for *H. sapiens*. This finding suggests that the *ACE2* of *S. apella*, *R. sinicus*, *G. gorilla gorilla*, *C. lanigera*, *A. nancymaae*, *P. paniscus*, *N. vison*, *S. bilineata* has a stronger interaction with SARS-CoV-2 RBD than that of human ACE2. To understand this difference, these animals' sequences and structures of ACE2 were analyzed and compared with human ACE2.

### Amino acid sequence comparison between human ACE2 & ACE2 of other animals

**Figure 5.**
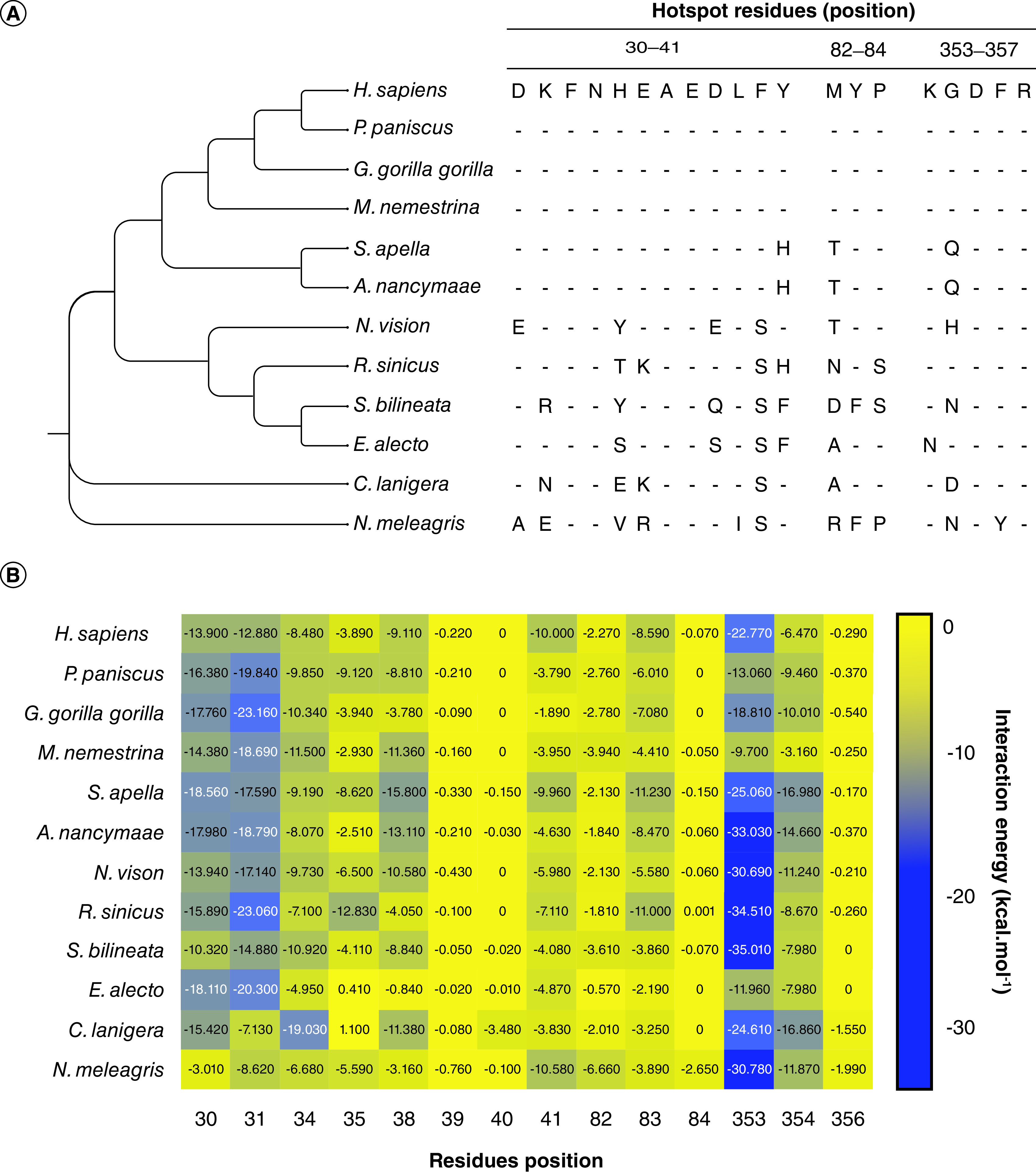

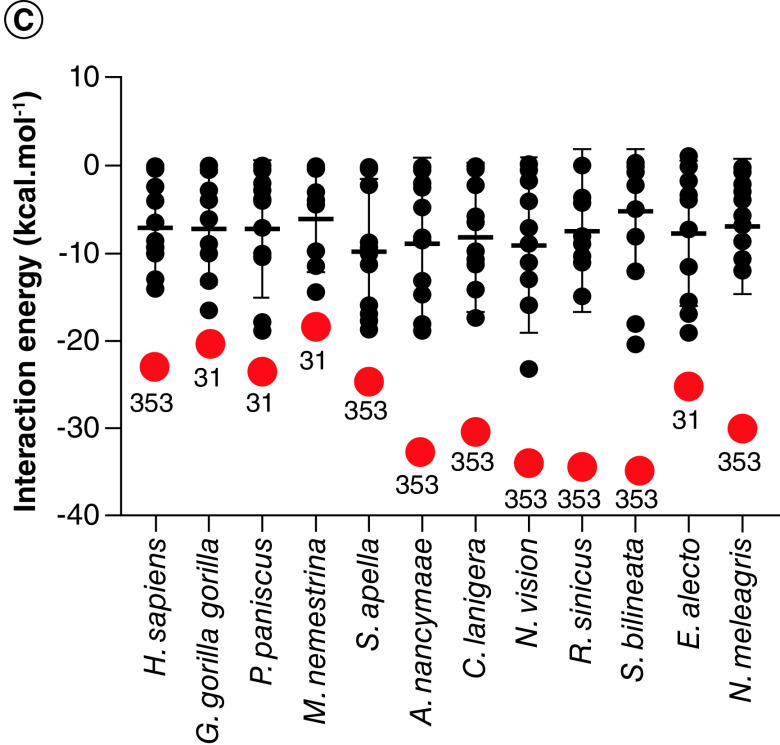
Critical variable sites in animals ACE2 protein in the interaction with RBD SARS-CoV-2.

The results obtained from molecular docking and dynamics associated with quantum biochemical analysis revealed the ACE2 residues that formed the complex between human ACE2 and RBD of SARS-CoV-2 S protein. Thus, we performed an alignment of all sequences to evaluate the presence of these residues in the ACE2 of the analyzed animal species (Figure 5A). Phe^32^, Asn^33^, Ala^36^, Glu^37^, Asp^355^ and Arg^357^ were the residues conserved among the 11 animal species with high potential of interaction with SARS-CoV-2 when compared with the human ACE2.

For Asp^30^, the alignment showed that only *N. vison* and *N. meleagris* have different amino acid residues, which were Glu^30^ and Ala^30^, respectively (Figure 5). The substitution of Asp^30^ with Glu^30^ and Ala^30^ leads to differences in energy in that position. Asp^30^ (*H. sapiens*) has an energy interaction of -13.90 kcal.mol^-1^, whereas Glu^30^ (*N. vison*) and Ala^30^ (*N. meleagris*) have -13.94 and -3.01 kcal.mol^-1^, respectively (Figure 5B). For human Lys^31^ (-12.88 kcal.mol^-1^), three animals have different amino acid residues in this position, but only *C. lanigera* and *N. meleagris* presented relevant differences in the energy of interactions, - 7.13 and -8.62 kcal.mol^-1^, respectively. The substitution of His^34^ to Glu^34^ in *C. lanigera* strength energy in this position, for Lys^35^ improves the energy interaction in R. sinicus almost threefold change compared with *H. sapiens*.

As for Asp^38^ in human ACE2, the species *E. allecto, S. bilineata*, and *N. vison* have different residues at the same position, as follows: Ser^38^ (*E. allecto*), Glu^38^ (*S. bilineata*), and Gln^38^ (*N. vison*) (Figure 5A and B), with the resulting energy of interaction of -0.84, -8.84, and -10.58, respectively, whereas Asp^38^ in human ACE2 has an energy of -9.11 kcal.mol^-1^. The residue Lys^353^ is an important amino acid for interactions between RBD:ACE2, and it is conserved in the analyzed species, except for the *E. allecto*, which has a different residue (Asn^353^) (Figure 5). In Figure 5C, it is possible to notice the hotspot residues contribution for the interaction energies. There are no significant differences in the median of interaction energy among the animals. The 353 and 31 residues were the most important energetic contributors to interaction and stabilization between the RBD and ACE2 (Figure 5C, red dots).

### Structural Aspects of ACE2 of animals

As expected, the structural alignment of human ACE2 with that of the analyzed animals revealed structural differences. The RMSD analysis was used, which calculates the atomic position, producing a score of differences between structures. The animals with the most different ACE2 compared with the human protein are *E. alecto, S. bilineata*, and *N. meleagris* with RMSD values of 1.108, 0.457, 0.423, respectively (Supplementary Figure 1). In contrast, *M. nemestrina*, *G. gorilla* and *P. paniscus* have the most similar ACE2 structure to that of human ACE2 with RMSD values of 0.129, 0.162, and 0.168, respectively (Supplementary Figure 1).

The superficial net charges of protein were also evaluated. The RBD detached from the viral S protein has a charge of 0, but when attached to the S protein, it presents a charge of -1 (data not shown). Human ACE2 has a superficial charge of -27 (Supplementary Figure 2). The analysis of superficial charges of ACE2 of animals revealed higher values than human ACE2 (Supplementary Figure 2).

## Discussion

The outbreak of SARS-CoV-2 is still ongoing, boosted by the emergence of new variants, with emphasis on Omicron, which led to a higher number of cases, infecting even vaccinated people worldwide, but with lower death rates. An important unanswered question remains: where did SARS-CoV-2 come from? However, before addressing this question, another one emerged: From where a new coronavirus could emerge? Answering this question is imperative, given the confirmed zoonotic transmission of SARS-CoV-2 [31]. Based on that, identifying or predicting animal species that could act as reservoirs or intermediate hosts of SARS-CoV-2 is essential. For instance, the recent report of a pangolin-coronavirus (pangolin-CoV) with 90% sequence identity to SARS-CoV-2 in Malayan pangolins (*M. javanica*) [7,32] partially addresses the first question, even though it does not necessarily indicate that SARS-CoV-2 used pangolins as intermediate hosts. Thus, determining the animal species that may potentially act as reservoirs or hosts for SARS-CoV-2 requires more investigation.

The first step of a viral infection is the interaction of viral attachment protein with a host cellular receptor. This interaction allows the virus to reach the cytoplasm, subvert cell machinery and start the replication process [5,33]. The recognition step is essential to determine the host range of species that can be infected and viral tissue tropism. SARS-CoV-2 uses the host ACE2 receptor to enter the cells in a species-specific manner [6]. ACE2 is a widespread receptor in vertebrates, so SARS-CoV-2 could infect all cells presenting this receptor, potentially conferring a plethora of viral hosts. However, Zhou *et al.* showed this is not entirely true, as they reported that ACE2 in humans, Chinese horseshoe bats (*R. sinicus*), civets, and pigs supported SARS-CoV-2 entry in cells, whereas ACE2 in mice did not. Even though this study showed not all animal species with ACE2 can be infected with SARS-CoV-2, the host range is still vast. Until now, it is proposed that SARS-CoV-2 spilled over from bats to humans through an unknown intermediate host [6]. For instance, Yen *et al.* [34] showed that hamsters could be naturally infected with SARS-CoV-2 and transmit the virus back to humans. These authors [34] revealed that pet hamsters contributed to the spread of the Delta variant in Hong Kong, infecting at least 50 people. Therefore, understanding which animals may act as an intermediate host for SARS-CoV-2 is still imperative.

To prospect new animal reservoirs or intermediate hosts, our group analyzed the ACE2 nucleotide and protein sequences of the ACE2 of 49 animal species belonging to different zoological classes, presenting high, intermediate, or low similarity with human ACE2. Our results predicted that 11 out of 49 evaluated animal species presented ACE2 with the same mode of interaction with the RBD of SARS-CoV-2 S protein, compared with human ACE2, most likely allowing viral entrance in animal cells and infection. Among these species with the same mode of interaction as that of human ACE2 are primates (*S. apella, G. gorilla gorilla, A. nancymaae, P. paniscus M. nemestrina*), bats (*R. sinicus*, *S. bilineata E. allecto*), rodents (*C. lanigera*), carnivores (*N. vison*) and birds (*N. meleagris*) (Figures 1–3). It is worth noting that this is the first-time different animals included in the present study were assessed, such as *N. vison*, *C. lanigera* and *N. meleagris*.

Recent *in vivo* studies reported that monkeys, bats, rodents, and *N. vison* [6,34–36] are reservoirs for SARS-CoV-2, supporting our *in silico* prediction of viral hosts. For instance, the ACE2 of primates (*G. gorilla* and *Pongo abelli*) and bats have been shown to interact with viral RBD by *in silico* and *in vitro* analyses [31,37], the variant Omicron (B.1.1.529) was associated with infections in rodents Diamond *et al.* [38] and Halfmann *et al.* [39]. A viral variant was associated with *N. vison* and detected in mink farms in Denmark [40].

On the other hand, our analyses showed that ACE2 of Helmeted guineafowl (*N. meleagris*) perfectly interacts with viral RBD. To the best of our knowledge, there are no *in silico*, *in vitro* or *in vivo* studies showing that the ACE2 of *N. meleagris* or that of any other bird allows SARS-CoV-2 cellular entry and infection. Thus, this is the first study on that Helmeted guineafowl (*N. meleagris*), a domestic bird originally from Africa [41], or any other bird species is suggested as a host for SARS-CoV-2 (Figures 1–3).

Despite the perfect interaction of ACE2 of animals with the RBD of SARS-CoV-2 S protein, the global energy revealed that the ACE2 of *R. sinicus, G. gorilla gorilla, A. nancymaae, S. apella, P. paniscus, C. lanigera, N. vison, E. Alecto, S. bilineata*, and *N. meleagris* present a higher affinity to the viral RBD than that of human ACE2, as shown by the analysis of the energy of interaction (Figure 3).

In their analysis, Li *et al.* [42] revealed that ACE2 orthologs contained the relevant amino acids at positions 31, 35, 38, 82 and 353, allowing them to function as SARS-CoV-2 receptors. Interestingly, our data corroborated with 31, 38 and 353 residues but not 35 and 82. Amino acids at position 35 and 82 exhibit high interaction energy for human (-3.89 kcal.mol^-1^ and -2.27 kcal.mol-1, respectively) and other animals, demonstrating these residues have little contribution to the ACE2 function as SARS-CoV-2 receptor. Additionally, our results show other residues as crucial for the ACE2-RBD interaction, such as the residues at positions 30, 31, 34, 38 and 353 (Figure 5).

A second study performed by Luan and co-workers [37] analyzed the interaction of ACE2 of *Bos taurus* (cattle), *Ophiophagus hannah* (king cobra), *Cricetulus griseus* (Chinese hamster), and *Pelodiscus sinensis* (Chinese softshell turtle) with SARS-CoV-2 RBD indicating that these different ACE2 proteins could support interaction with SARS-CoV-2 and these animals could act as viral reservoirs [37]. The authors also analyzed the amino acidic sequence of the ACE2 of some bird species, such as *Gallus gallus* (chicken)*, Anas platyrhynchos* (duck), and *Meleagris gallopavo* (turkey). Still, they did not perform docking analyses, only protein sequence analysis.

Interestingly, among these 11 animal species that had the best interaction between their ACE2 and viral RBD, they show differences in evolutionary distance and nucleotide sequence similarity compared with human ACE2. The five primates (*S. apella, G. gorilla gorilla, A. nancymaae, P. paniscus, M. nemestrina*) are phylogenetically closer to humans with low p-distance (0.1–0.6) values and high nucleotide sequence similarity. Also, human ACE2 differs from those of *S. bilineata*, *R. sinicus*, *E. allecto*, *N. vison*, and *C. lanigera* orthologs by only 17.55%, 16.21%, 16.79%, 15.97% and 14.80%, respectively. However, *N. meleagris* ACE2, although presenting an evolutionary difference of 40.26% from human ACE2, strongly interacted with SARS-CoV-2 S protein RBD, as shown by the high energy of interaction between these molecules.

By assessing biological behavior and molecular interactions, *in silico* approaches offer a platform for understanding possible applications for additional *in vitro* and *in vivo* experiments [43]. Even though *in silico* models are made possible by the availability of large datasets linked to high-throughput screening, bioinformatics algorithms to mine and annotate the data, the limitations of these computational methods reaffirm the requirement that experimental validation should be carried out, both *in vitro* and *in vivo*. We therefore encourage further *in vitro* or *in vivo* research to support the findings of this study.

## Conclusion

Our analyses predicted that ACE2 of eleven animal species, including primates, bats, carnivores, and birds, supports SARS-CoV-2 infection, which makes them potential candidate hosts for this emerging pandemic virus. Among animals already confirmed as hosts for SARS-CoV-2, such as *N. vison* and *C. lanigera*, we reported for the first time the prediction that the ACE2 of a bird (*N. meleagris*) supports SARS-CoV-2 infection. These results produced new information by predicting the animal species that might act as SARS-CoV-2 reservoirs or hosts, which ultimately helps in understanding the epidemiological cycle of this pathogen and establishing surveillance strategies to control COVID-19.

**Summary points**

- *ACE2* of primates is phylogenetically closer to that of humans, avian and fish *ACE2* are the most divergent from humans. Eleven species had a perfect fit for the interaction between their ACE2 and viral RBD (same configuration and position) compared with the human ACE2:RBD interaction.
- The global energy revealed that the ACE2 of *R. sinicus, G. gorilla gorilla, A. nancymaae, S. apella, P. paniscus, C. lanigera, N. vison, E. alecto, S. bilineata* and *N. meleagris* present a higher affinity to the viral RBD than human ACE2.
- Our predicted results followed recently *in vivo* reports that rodents, monkeys, and bats (probable source of SARS-CoV-2) act as reservoirs for SARS-CoV-2.
- Our analysis also predicted that *ACE2* of the *C. lanigera* and *N. vison* support viral cell entry, given their interaction with the SARS-CoV-2 RBD.
- Although *N. meleagris* is phylogenetically distant from humans, it presents a good interaction with RBD from SARS-CoV-2, suggesting that this bird may act as a reservoir for the virus.
- Our results show that other amino acid residues are crucial for the ACE2-RBD interaction, such as the residues at positions 30, 31, 34, 38, and 353.
