# Supplementary figures and images for: In silico analysis of ACE2 from different animal species provides new insights into SARS-CoV-2 species spillover
Source: Future Virol. 2023 Apr 11:10.2217/fvl-2022-0187. doi: 10.2217/fvl-2022-0187 (PMC10096339; doi:10.2217/fvl-2022-0187)

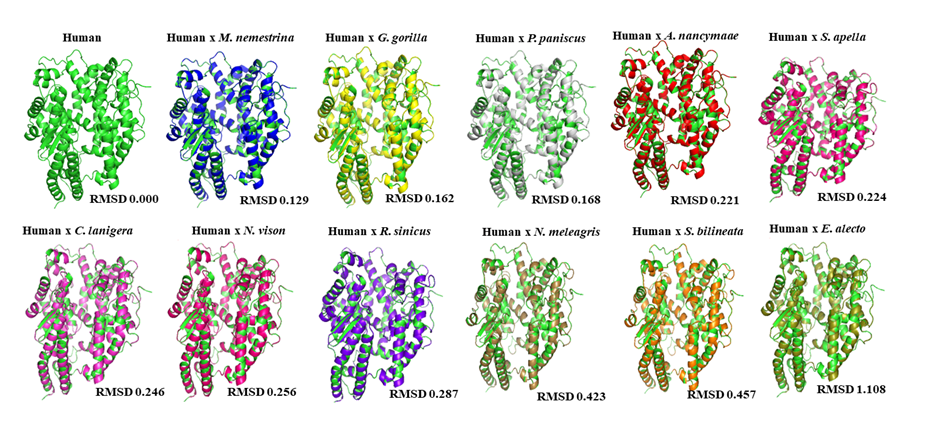

Supplement: Supplementary file 1 [file supplementary-figure-1.tif]
