# Supplementary material for: In silico analysis of ACE2 from different animal species provides new insights into SARS-CoV-2 species spillover
Source: Future Virol. 2023 Apr 11:10.2217/fvl-2022-0187. doi: 10.2217/fvl-2022-0187 (PMC10096339; doi:10.2217/fvl-2022-0187)
Supplement: Supplementary file 2 [file supplementary-tables.docx]

**Supplementary table 1.** Interactions between SARS-CoV-2 RBD e *Homo sapiens* ACE2

| **Interaction** | **SARS-CoV-2  RBD** | ***Homo sapiens*  ACE2** |
| --- | --- | --- |
|  | Residue | Residue |
| Hydrophobic | TYR489 | PHE28 |
|  | PHE486 | LEU79 |
|  | PHE486 | MET82 |
|  | PHE486 | TYR83 |
| Hydrogen bonds | GLY502 | LYS353 |
|  | GLY446 | GLN42 |
|  | GLY496 | LYS353 |
|  | THR500 | ASP355 |
|  | GLN493 | GLU35 |
|  | ASN501 | TYR41 |
|  | TYR449 | GLN42 |
|  | ASN487 | TYR83 |
|  | TYR489 | TYR83 |
|  | TYR505 | ARG393 |
|  | LYS417 | ASP30 |
|  | TYR449 | ASP38 |
|  | TYR449 | GLN42 |
|  | ASN487 | TYR83 |
|  | TYR489 | TYR83 |
|  | GLN493 | GLU35 |
|  | THR500 | TYR41 |
|  | ASN501 | TYR41 |
|  | TYR505 | GLU37 |
| Ionic | LYS417 | ASP30 |
|  | GLU484 | LYS31 |
|  | ARG403 | GLU37 |
| Aromatic-Aromatic | TYR489 | PHE28 |
|  | PHE486 | TYR83 |
| Aromatic-Sulphur | PHE486 | MET82 |

**Supplementary table 2.** Quantum biochemistry between SARS-CoV-2 RBD e *Homo sapiens* ACE2

| **Amino acid residues** | | **Distance** | **Interaction energy** | **Residue charge** | | **Residue atom** | |
| --- | --- | --- | --- | --- | --- | --- | --- |
| SARS-CoV-2  RBD | *Homo sapiens* ACE2 | (Å) | (kcal mol-1) | SARS-CoV-2  RBD | *Homo sapiens* ACE2 | SARS-CoV-2  RBD | *Homo sapiens* ACE2 |
| LYS417 | ASP30 | 1.59 | -10.93 | 1 | -1 | HZ1 | OD2 |
| TYR505 | GLU37 | 1.66 | -9.36 | 0 | -1 | HH | OE2 |
| GLN493 | GLU35 | 1.74 | -4.74 | 0 | -1 | HE22 | OE2 |
| TYR449 | ASP38 | 1.76 | -7.07 | 0 | -1 | HH | OD2 |
| GLY496 | LYS353 | 1.81 | -2.91 | 0 | 1 | O | HZ1 |
| THR500 | TYR41 | 1.82 | -3.22 | 0 | 0 | HG1 | OH |
| GLY502 | LYS353 | 1.87 | -2.93 | 0 | 1 | H | O |
| TYR449 | GLN42 | 2.04 | -1.04 | 0 | 0 | OH | HE21 |
| GLY446 | GLN42 | 2.10 | -2.49 | 0 | 0 | O | HE22 |
| LEU455 | LYS31 | 2.11 | -1.54 | 0 | 1 | HD23 | HA |
| THR500 | ASN330 | 2.12 | -1.51 | 0 | 0 | HA | HD22 |
| TYR489 | LYS31 | 2.13 | -5.35 | 0 | 1 | HB2 | HD2 |
| ASN487 | TYR83 | 2.15 | -4.16 | 0 | 0 | OD1 | HH |
| PHE456 | ASP30 | 2.18 | -1.78 | 0 | -1 | HE1 | HB2 |
| PHE486 | LEU79 | 2.19 | -3.03 | 0 | 0 | HD1 | HD21 |
| TYR473 | THR27 | 2.23 | -1.36 | 0 | 0 | HE2 | HG21 |
| ASN501 | LYS353 | 2.24 | -7.99 | 0 | 1 | HD22 | HD2 |
| TYR489 | THR27 | 2.25 | -1.84 | 0 | 0 | HE2 | HG22 |
| THR500 | ARG357 | 2.26 | -0.52 | 0 | 1 | HB | HH11 |
| TYR505 | LYS353 | 2.27 | -6.73 | 0 | 1 | HB2 | HB2 |
| PHE486 | MET82 | 2.28 | -1.76 | 0 | 0 | HZ | HB1 |
| TYR489 | PHE28 | 2.28 | -2.68 | 0 | 0 | HH | HD1 |
| PHE456 | THR27 | 2.31 | -2.14 | 0 | 0 | HE1 | HG23 |
| LEU455 | HIS34 | 2.32 | -1.29 | 0 | 0 | HD13 | HD1 |
| ALA475 | GLN24 | 2.35 | -2.29 | 0 | 0 | O | HG1 |
| TYR505 | ARG393 | 2.36 | 0.71 | 0 | 1 | HH | HH22 |
| GLN493 | HIS34 | 2.36 | -1.90 | 0 | 0 | HG1 | HB2 |
| ALA475 | THR27 | 2.39 | -1.86 | 0 | 0 | HB3 | HG21 |
| ASN501 | TYR41 | 2.42 | -2.40 | 0 | 0 | HD21 | OH |
| GLY502 | GLY354 | 2.44 | -2.71 | 0 | 0 | HA2 | HA2 |
| PHE456 | LYS31 | 2.45 | -2.34 | 0 | 1 | HZ | HB1 |
| GLN498 | TYR41 | 2.45 | -2.79 | 0 | 0 | HG1 | HE2 |
| TYR453 | HIS34 | 2.68 | -2.59 | 0 | 0 | HH | CD2 |
| THR500 | ASP355 | 2.77 | 0.36 | 0 | -1 | HB | OD2 |
| PHE486 | TYR83 | 2.78 | -3.19 | 0 | 0 | HE1 | HE1 |
| LEU455 | ASP30 | 2.81 | -0.01 | 0 | -1 | HD13 | O |
| ASN501 | ASP355 | 2.83 | 0.34 | 0 | -1 | HA | HB1 |
| GLN498 | LYS353 | 2.84 | -1.42 | 0 | 1 | HE22 | HZ3 |
| ASN487 | GLN24 | 2.87 | -1.38 | 0 | 0 | OD1 | HB2 |
| GLY476 | GLN24 | 2.87 | -1.27 | 0 | 0 | HA1 | HE21 |
| GLN493 | LYS31 | 2.92 | 0.72 | 0 | 1 | HE22 | HZ3 |
| TYR489 | TYR83 | 3.02 | -0.67 | 0 | 0 | HH | OH |
| LYS417 | HIS34 | 3.05 | -1.24 | 1 | 0 | HE2 | HE1 |
| GLU484 | LYS31 | 3.05 | -3.16 | -1 | 1 | OE1 | HZ1 |
| GLY496 | ASP38 | 3.13 | -0.39 | 0 | -1 | HA2 | OD1 |
| TYR505 | GLY354 | 3.35 | -1.03 | 0 | 0 | HD1 | N |
| GLN498 | LEU45 | 3.45 | -0.59 | 0 | 0 | HG1 | HD22 |
| GLY502 | THR324 | 3.54 | -0.38 | 0 | 0 | HA2 | HG21 |
| GLY447 | GLN42 | 3.57 | -0.81 | 0 | 0 | HA2 | HE22 |
| GLN498 | GLN42 | 3.57 | -0.70 | 0 | 0 | HE21 | HE22 |
| GLY502 | ASP355 | 3.73 | -0.80 | 0 | -1 | H | N |
| TYR449 | LYS353 | 3.85 | -0.05 | 0 | 1 | HH | HZ3 |
| ALA475 | SER19 | 3.89 | -0.35 | 0 | 1 | O | H2 |
| THR500 | LEU45 | 3.93 | -0.67 | 0 | 0 | HG23 | HD21 |
| PHE497 | LYS353 | 3.93 | -2.41 | 0 | 1 | HA | HZ1 |
| GLY502 | GLY326 | 3.94 | -0.13 | 0 | 0 | HA1 | HA2 |
| PHE490 | LYS31 | 4.01 | -0.76 | 0 | 1 | H | HZ1 |
| THR500 | GLY326 | 4.02 | -0.38 | 0 | 0 | HA | HA1 |
| ALA475 | TYR83 | 4.14 | -0.23 | 0 | 0 | HB1 | HH |
| PRO499 | ASN330 | 4.15 | -0.07 | 0 | 0 | O | HD22 |
| GLN506 | GLN325 | 4.20 | -0.33 | 0 | 0 | HE22 | HB2 |
| GLY504 | GLY354 | 4.26 | -0.35 | 0 | 0 | H | HA2 |
| VAL445 | LEU45 | 4.29 | -0.28 | 0 | 0 | HG11 | HD21 |
| GLN506 | GLY326 | 4.33 | -0.14 | 0 | 0 | HE22 | H |
| TYR505 | ALA386 | 4.33 | -0.29 | 0 | 0 | HE1 | HB2 |
| ARG403 | HIS34 | 4.36 | -0.54 | 1 | 0 | HH11 | HD2 |
| SER477 | GLN24 | 4.40 | -0.38 | 0 | 0 | H | HE21 |
| TYR489 | LEU79 | 4.42 | -0.34 | 0 | 0 | HE1 | HD13 |
| TYR495 | LYS353 | 4.45 | 2.14 | 0 | 1 | HA | HZ2 |
| ASN487 | PHE28 | 4.46 | -0.56 | 0 | 0 | H | HD1 |
| ALA475 | GLU23 | 4.46 | -0.15 | 0 | -1 | HB2 | O |
| VAL503 | THR324 | 4.47 | -0.32 | 0 | 0 | H | HG21 |
| ASN501 | GLY352 | 4.47 | -0.15 | 0 | 0 | HD21 | O |
| ASN501 | GLY326 | 4.49 | -0.15 | 0 | 0 | O | HA2 |
| GLN498 | ASP38 | 4.49 | -0.31 | 0 | -1 | HE22 | OD1 |
| GLY446 | LEU45 | 4.53 | -0.19 | 0 | 0 | H | HD23 |
| PHE486 | PHE28 | 4.54 | -0.62 | 0 | 0 | HE1 | HE1 |
| TYR421 | ASP30 | 4.55 | -0.17 | 0 | -1 | HE2 | OD2 |
| VAL503 | GLN325 | 4.55 | -0.28 | 0 | 0 | HG21 | HB2 |
| ASN487 | LEU79 | 4.56 | -0.30 | 0 | 0 | H | HD21 |
| ASN501 | GLY354 | 4.61 | -1.65 | 0 | 0 | C | HA2 |
| ALA475 | PHE28 | 4.65 | -0.23 | 0 | 0 | HB2 | H |
| PHE456 | PHE28 | 4.67 | -0.15 | 0 | 0 | HZ | HA |
| TYR489 | GLN24 | 4.69 | -0.26 | 0 | 0 | HE2 | O |
| GLY502 | PHE356 | 4.71 | -0.29 | 0 | 0 | HA2 | HD2 |
| PHE486 | ALA80 | 4.72 | -0.19 | 0 | 0 | HE1 | N |
| VAL503 | GLY354 | 4.74 | -0.13 | 0 | 0 | H | HA2 |
| TYR489 | GLN76 | 4.76 | -0.22 | 0 | 0 | HE1 | HE22 |
| SER494 | ASP38 | 4.83 | -0.11 | 0 | -1 | O | HB1 |
| GLN474 | THR27 | 4.86 | -0.11 | 0 | 0 | C | HG21 |
| CYS488 | LYS31 | 4.89 | -0.14 | 0 | 1 | O | HE1 |
| ARG403 | GLU37 | 4.90 | -1.91 | 1 | -1 | HH12 | OE2 |
| PRO499 | GLU329 | 4.97 | 0.01 | 0 | -1 | O | OE2 |
| ASN487 | THR27 | 4.98 | -0.37 | 0 | 0 | OD1 | HB |
| LEU455 | GLU35 | 4.98 | -0.31 | 0 | -1 | HD21 | HB1 |
| PHE456 | LYS26 | 4.99 | -0.19 | 0 | 1 | HE1 | O |
| TYR505 | HIS34 | 4.99 | -0.16 | 0 | 0 | HE2 | HD2 |
| PHE456 | PHE32 | 5.05 | -0.12 | 0 | 0 | HZ | H |
| GLY496 | GLN42 | 5.05 | -0.09 | 0 | 0 | HA2 | HE21 |
| GLY502 | GLY352 | 5.08 | 0.10 | 0 | 0 | H | O |
| GLN493 | ASP38 | 5.14 | -0.62 | 0 | -1 | HG2 | HB1 |
| VAL503 | GLY326 | 5.14 | -0.08 | 0 | 0 | HG21 | H |
| GLY485 | LEU79 | 5.15 | -0.17 | 0 | 0 | O | HD21 |
| TYR495 | ASP38 | 5.17 | -0.80 | 0 | -1 | C | OD1 |
| THR500 | LEU351 | 5.18 | -0.23 | 0 | 0 | HB | HD22 |
| SER494 | LYS353 | 5.18 | -0.15 | 0 | 1 | O | HZ2 |
| THR500 | LYS353 | 5.19 | -0.38 | 0 | 1 | O | O |
| ALA475 | ALA25 | 5.21 | -0.08 | 0 | 0 | HB2 | N |
| LEU455 | THR27 | 5.23 | -0.33 | 0 | 0 | HD23 | O |
| GLY447 | LYS353 | 5.25 | 0.04 | 0 | 1 | HA1 | HZ1 |
| GLY504 | LYS353 | 5.25 | -0.27 | 0 | 1 | H | O |
| ASN501 | ASN330 | 5.26 | -0.22 | 0 | 0 | N | HD22 |
| TYR489 | ASP30 | 5.27 | -0.28 | 0 | -1 | HD2 | HB2 |
| GLN506 | LYS353 | 5.27 | -0.39 | 0 | 1 | H | O |
| PRO499 | GLY326 | 5.32 | -0.12 | 0 | 0 | O | HA1 |
| THR500 | PHE356 | 5.32 | 0.01 | 0 | 0 | O | H |
| ARG403 | LYS353 | 5.34 | 0.94 | 1 | 1 | HD1 | HG1 |
| THR500 | GLY354 | 5.37 | -0.43 | 0 | 0 | O | C |
| THR500 | GLU329 | 5.39 | -0.28 | 0 | -1 | HA | OE2 |
| LEU455 | PHE32 | 5.42 | -0.19 | 0 | 0 | HD23 | N |
| LYS417 | THR27 | 5.43 | -0.32 | 1 | 0 | HZ1 | HA |
| VAL445 | ASN49 | 5.43 | -0.05 | 0 | 0 | HG11 | HD22 |
| GLN493 | ASP30 | 5.44 | -0.03 | 0 | -1 | OE1 | O |
| ASN501 | ARG357 | 5.46 | -0.17 | 0 | 1 | HD21 | HH11 |
| PHE456 | HIS34 | 5.47 | -0.27 | 0 | 0 | HZ | HD1 |
| PHE456 | LEU29 | 5.49 | -0.22 | 0 | 0 | HZ | C |
| GLY446 | TYR41 | 5.50 | -0.17 | 0 | 0 | H | HE2 |
| PHE486 | THR78 | 5.51 | -0.05 | 0 | 0 | HE1 | O |
| GLN498 | ASP355 | 5.52 | -0.10 | 0 | -1 | HB1 | OD2 |
| TYR505 | GLY352 | 5.53 | -0.62 | 0 | 0 | HH | HA1 |
| ARG403 | ARG393 | 5.53 | 1.27 | 1 | 1 | HH12 | HH22 |
| GLY476 | SER19 | 5.53 | -0.35 | 0 | 1 | HA1 | H2 |
| TYR489 | LEU29 | 5.55 | -0.29 | 0 | 0 | HE2 | N |
| GLY447 | TYR41 | 5.56 | -0.13 | 0 | 0 | HA1 | HE2 |
| TYR449 | LEU39 | 5.56 | -0.22 | 0 | 0 | HH | N |
| TYR449 | TYR41 | 5.58 | -0.18 | 0 | 0 | HH | HB2 |
| VAL503 | LYS353 | 5.60 | 0.09 | 0 | 1 | N | O |
| ASN487 | ILE21 | 5.61 | -0.08 | 0 | 0 | HD22 | HD2 |
| ASN439 | GLU329 | 5.61 | -0.28 | 0 | -1 | HD22 | OE2 |
| GLU484 | GLU35 | 5.62 | 1.67 | -1 | -1 | OE1 | OE2 |
| GLN493 | GLU37 | 5.63 | -0.15 | 0 | -1 | HG1 | HB2 |
| TYR505 | ASN33 | 5.64 | -0.11 | 0 | 0 | HH | O |
| GLN506 | THR324 | 5.68 | -0.07 | 0 | 0 | HE21 | HB |
| TYR505 | ASP355 | 5.68 | -0.24 | 0 | -1 | HB1 | H |
| PHE486 | GLN24 | 5.70 | -0.32 | 0 | 0 | HZ | HB2 |
| ASN487 | ALA25 | 5.72 | -0.05 | 0 | 0 | OD1 | N |
| TYR505 | PHE390 | 5.74 | -0.08 | 0 | 0 | HH | HD2 |
| GLY502 | TYR41 | 5.74 | -0.12 | 0 | 0 | H | HE1 |
| TYR453 | ASP30 | 5.76 | -0.22 | 0 | -1 | HH | O |
| ASN501 | LEU45 | 5.76 | -0.08 | 0 | 0 | HD21 | HD22 |
| SER494 | HIS34 | 5.77 | -0.13 | 0 | 0 | O | O |
| ALA475 | THR20 | 5.78 | -0.01 | 0 | 0 | O | O |
| GLN498 | ARG357 | 5.78 | -0.06 | 0 | 1 | HB2 | HH12 |
| ASN487 | MET82 | 5.80 | -0.33 | 0 | 0 | HD22 | HB1 |
| GLY476 | TYR83 | 5.80 | -0.09 | 0 | 0 | HA1 | HE2 |
| GLY447 | ASP38 | 5.81 | -0.11 | 0 | -1 | HA2 | OD1 |
| CYS488 | TYR83 | 5.81 | -0.13 | 0 | 0 | H | HH |
| TYR505 | ALA387 | 5.82 | -0.06 | 0 | 0 | HE1 | HA |
| GLY502 | GLN325 | 5.85 | -0.06 | 0 | 0 | HA1 | H |
| TYR473 | GLU23 | 5.87 | -0.20 | 0 | -1 | HH | HB2 |
| ASN501 | THR324 | 5.88 | -0.04 | 0 | 0 | O | HB |
| GLY502 | PHE327 | 5.88 | -0.03 | 0 | 0 | HA1 | H |
| THR500 | GLY352 | 5.90 | -0.04 | 0 | 0 | O | O |
| GLN493 | ALA36 | 5.91 | -0.15 | 0 | 0 | HE22 | N |
| ASN501 | LEU351 | 5.92 | -0.09 | 0 | 0 | HD21 | HB1 |
| LEU455 | ASN33 | 5.92 | -0.15 | 0 | 0 | HD13 | HB1 |
| SER477 | SER19 | 5.94 | -0.13 | 0 | 1 | HB2 | HA |
| ASN439 | ASN330 | 5.95 | -0.02 | 0 | 0 | HD22 | HD22 |
| TYR505 | PHE356 | 5.98 | -0.13 | 0 | 0 | HD1 | HE2 |
| ARG403 | ALA387 | 5.98 | -0.11 | 1 | 0 | HH22 | HA |
| ASN439 | GLN325 | 5.98 | -0.08 | 0 | 0 | HD21 | HE21 |
| GLN506 | GLY354 | 6.01 | -0.11 | 0 | 0 | H | HA2 |
| TYR489 | PHE32 | 6.01 | -0.22 | 0 | 0 | HE2 | H |
| GLN506 | GLU329 | 6.03 | -0.14 | 0 | -1 | HE22 | OE2 |
| TYR473 | ASP30 | 6.03 | -0.19 | 0 | -1 | HE2 | HB2 |
| PRO499 | LEU45 | 6.08 | -0.09 | 0 | 0 | HD1 | HD21 |
| GLY496 | TYR41 | 6.08 | -0.13 | 0 | 0 | O | HD2 |
| LYS417 | LYS31 | 6.10 | 0.67 | 1 | 1 | HZ1 | N |
| ARG403 | ALA386 | 6.10 | -0.20 | 1 | 0 | HH22 | O |
| TYR453 | GLU37 | 6.11 | -0.07 | 0 | -1 | HH | HG1 |
| GLN493 | PHE32 | 6.11 | 0.07 | 0 | 0 | OE1 | N |
| PHE486 | GLN81 | 6.12 | -0.18 | 0 | 0 | HE1 | H |
| THR500 | ASN49 | 6.13 | -0.04 | 0 | 0 | HG23 | HD22 |
| PRO499 | TYR41 | 6.13 | -0.29 | 0 | 0 | HD1 | HE2 |
| VAL445 | TYR41 | 6.14 | -0.09 | 0 | 0 | HA | HE2 |
| ARG457 | THR27 | 6.15 | -0.08 | 1 | 0 | H | HG23 |
| LEU492 | LYS31 | 6.16 | -0.14 | 0 | 1 | O | HZ1 |
| SER494 | GLU35 | 6.17 | -0.05 | 0 | -1 | H | OE2 |
| TYR489 | GLU35 | 6.17 | -0.21 | 0 | -1 | HB2 | OE2 |
| CYS488 | PHE28 | 6.17 | -0.11 | 0 | 0 | H | HD1 |
| PHE497 | ASP38 | 6.18 | 0.08 | 0 | -1 | N | OD1 |
| GLY504 | THR324 | 6.18 | -0.01 | 0 | 0 | H | HG21 |
| TYR473 | LYS31 | 6.18 | -0.14 | 0 | 1 | HD2 | HB1 |
| ASN448 | LYS353 | 6.18 | -0.19 | 0 | 1 | H | HZ1 |
| GLY485 | LYS31 | 6.19 | -0.17 | 0 | 1 | H | HE1 |
| GLN493 | PHE72 | 6.20 | -0.05 | 0 | 0 | HE22 | HE1 |
| GLY476 | THR27 | 6.20 | -0.10 | 0 | 0 | H | HG21 |
| CYS488 | THR27 | 6.21 | -0.12 | 0 | 0 | HA | HG21 |
| GLY447 | LEU45 | 6.23 | -0.04 | 0 | 0 | N | HD23 |
| TYR489 | LYS26 | 6.23 | -0.06 | 0 | 1 | HE2 | C |
| LYS417 | LYS26 | 6.25 | 0.77 | 1 | 1 | HZ1 | O |
| ARG408 | ALA387 | 6.26 | -0.09 | 1 | 0 | HH12 | HB1 |
| PRO491 | LYS31 | 6.31 | -0.27 | 0 | 1 | HA | HD2 |
| ASN501 | PHE356 | 6.32 | -0.08 | 0 | 0 | HA | H |
| TYR473 | GLN24 | 6.35 | -0.10 | 0 | 0 | HE2 | HA |
| TYR505 | GLN388 | 6.37 | -0.04 | 0 | 0 | HH | O |
| GLN474 | GLN24 | 6.37 | 0.01 | 0 | 0 | C | HG1 |
| TYR453 | GLU35 | 6.37 | -0.03 | 0 | -1 | OH | N |
| PHE486 | ILE21 | 6.38 | -0.08 | 0 | 0 | HZ | HD2 |
| ASN439 | GLY326 | 6.39 | -0.02 | 0 | 0 | HD22 | HA1 |
| TYR495 | HIS34 | 6.40 | -0.18 | 0 | 0 | HD1 | HD2 |
| CYS488 | LEU79 | 6.40 | -0.04 | 0 | 0 | H | HD21 |
| TYR453 | ASN33 | 6.41 | -0.05 | 0 | 0 | HH | O |
| PHE486 | PRO84 | 6.42 | -0.07 | 0 | 0 | HZ | HD2 |
| LEU455 | LEU29 | 6.44 | -0.21 | 0 | 0 | HD13 | O |
| ASP405 | ALA387 | 6.46 | -0.02 | -1 | 0 | OD2 | HA |
| VAL445 | ARG357 | 6.47 | 0.12 | 0 | 1 | HG11 | HH12 |
| ALA475 | LYS26 | 6.47 | -0.08 | 0 | 1 | HB2 | H |
| PHE456 | GLU35 | 6.51 | -0.01 | 0 | -1 | HE2 | HB1 |
| GLN506 | ASN330 | 6.51 | -0.01 | 0 | 0 | HE21 | HD22 |
| GLY476 | ILE21 | 6.51 | -0.02 | 0 | 0 | HA1 | HD3 |
| GLN493 | ASN33 | 6.52 | -0.17 | 0 | 0 | HG1 | C |
| TYR505 | PRO389 | 6.54 | -0.02 | 0 | 0 | HH | HA |
| GLY485 | PHE28 | 6.55 | -0.04 | 0 | 0 | O | HD1 |
| PRO499 | GLN325 | 6.56 | -0.08 | 0 | 0 | O | HG1 |
| PHE490 | GLU35 | 6.57 | 0.06 | 0 | -1 | HB2 | OE2 |
| ASN448 | GLN42 | 6.58 | -0.08 | 0 | 0 | N | HE22 |
| GLY476 | THR20 | 6.58 | 0.00 | 0 | 0 | HA1 | O |
| TYR489 | ALA25 | 6.59 | -0.06 | 0 | 0 | HE2 | O |
| GLY504 | PHE356 | 6.60 | -0.03 | 0 | 0 | H | HE2 |
| THR500 | PHE327 | 6.61 | -0.09 | 0 | 0 | O | N |
| GLY502 | ASN330 | 6.61 | -0.02 | 0 | 0 | HA1 | HD21 |
| ARG457 | ASP30 | 6.62 | -0.69 | 1 | -1 | H | OD2 |
| GLY446 | LYS353 | 6.64 | -0.09 | 0 | 1 | O | HZ3 |
| GLN506 | ASP355 | 6.66 | -0.15 | 0 | -1 | HE21 | HA |
| VAL503 | PHE356 | 6.67 | -0.05 | 0 | 0 | H | HD2 |
| VAL445 | GLN42 | 6.67 | -0.09 | 0 | 0 | C | HE22 |
| ALA475 | LYS31 | 6.67 | 0.03 | 0 | 1 | HB3 | HB1 |
| ALA475 | ILE21 | 6.67 | -0.05 | 0 | 0 | HB2 | O |
| GLN493 | LEU39 | 6.68 | -0.06 | 0 | 0 | HE22 | HG |
| TYR495 | GLU37 | 6.69 | -0.13 | 0 | -1 | HD1 | HG1 |
| GLY485 | TYR83 | 6.70 | -0.09 | 0 | 0 | O | HH |
| PHE497 | TYR41 | 6.70 | -0.19 | 0 | 0 | C | HE2 |
| GLU406 | HIS34 | 6.70 | 0.24 | -1 | 0 | OE2 | HD2 |
| GLY446 | ASP38 | 6.71 | 0.17 | 0 | -1 | O | OD2 |
| TYR421 | THR27 | 6.72 | -0.05 | 0 | 0 | HE2 | HG23 |
| LEU455 | PHE28 | 6.72 | -0.09 | 0 | 0 | HD23 | O |
| GLY496 | GLU37 | 6.72 | 0.07 | 0 | -1 | H | HB2 |
| ASN501 | GLN325 | 6.73 | -0.07 | 0 | 0 | O | HB2 |
| ASP405 | LYS353 | 6.73 | -0.83 | -1 | 1 | OD2 | HA |
| GLN498 | GLU37 | 6.77 | 0.11 | 0 | -1 | HE22 | O |
| TYR449 | GLU37 | 6.79 | 0.12 | 0 | -1 | HH | C |
| PRO499 | ARG357 | 6.80 | 0.17 | 0 | 1 | HD1 | HH12 |
| VAL503 | ASP355 | 6.81 | 0.07 | 0 | -1 | H | HA |
| ASP405 | ALA386 | 6.81 | 0.01 | -1 | 0 | OD2 | O |
| LYS444 | TYR41 | 6.82 | -0.06 | 1 | 0 | O | HE2 |
| ASN487 | GLU23 | 6.82 | -0.01 | 0 | -1 | OD1 | O |
| TYR473 | PHE28 | 6.82 | -0.08 | 0 | 0 | HE2 | N |
| THR478 | GLN24 | 6.83 | -0.06 | 0 | 0 | H | HE21 |
| SER494 | GLU37 | 6.83 | -0.01 | 0 | -1 | O | HB2 |
| ASN501 | ASP38 | 6.84 | -0.04 | 0 | -1 | HD22 | HA |
| GLN474 | SER19 | 6.86 | -0.01 | 0 | 1 | O | H2 |
| TYR453 | ASP38 | 6.88 | 0.05 | 0 | -1 | HE1 | HB1 |
| ASP405 | GLY354 | 6.89 | 0.03 | -1 | 0 | OD2 | HA2 |
| TYR453 | LYS31 | 6.89 | -0.14 | 0 | 1 | HH | HA |
| LEU492 | GLU35 | 6.90 | -0.26 | 0 | -1 | O | OE2 |
| THR500 | GLN325 | 6.91 | -0.05 | 0 | 0 | HA | C |
| ASN501 | PHE327 | 6.91 | -0.02 | 0 | 0 | O | H |
| GLY496 | HIS34 | 6.92 | -0.02 | 0 | 0 | H | O |
| THR500 | TRP48 | 6.93 | -0.07 | 0 | 0 | HG23 | HD1 |
| GLN498 | ASN330 | 6.94 | -0.11 | 0 | 0 | HB2 | HD22 |
| TYR489 | GLU23 | 6.95 | 0.04 | 0 | -1 | HE2 | O |
| SER494 | LYS31 | 6.95 | -0.13 | 0 | 1 | H | HZ3 |
| ASN487 | LYS31 | 6.95 | -0.08 | 0 | 1 | HA | HG1 |
| ARG403 | GLY354 | 6.95 | -0.09 | 1 | 0 | HH21 | N |
| PHE486 | GLN76 | 6.96 | -0.12 | 0 | 0 | HE1 | O |
| GLN498 | GLY352 | 6.96 | -0.06 | 0 | 0 | OE1 | O |
| PRO499 | ASP355 | 6.97 | -0.45 | 0 | -1 | C | OD2 |
| VAL445 | ASN330 | 6.97 | -0.05 | 0 | 0 | HG22 | OD1 |
| PHE456 | GLU23 | 6.97 | -0.09 | 0 | -1 | HE1 | O |
| PHE456 | ASN33 | 6.97 | -0.04 | 0 | 0 | HZ | H |
| TYR505 | ASP38 | 6.98 | -0.06 | 0 | -1 | HE2 | N |
| GLN493 | LYS353 | 6.98 | 0.18 | 0 | 1 | HG2 | HZ2 |
| SER477 | ILE21 | 6.98 | -0.03 | 0 | 0 | HG | HD3 |
| TYR505 | TYR41 | 6.99 | -0.14 | 0 | 0 | HB2 | HE1 |
| TYR453 | LYS353 | 7.00 | -0.10 | 0 | 1 | HE1 | HZ2 |
| THR500 | THR324 | 7.01 | -0.03 | 0 | 0 | O | HB |
| TYR473 | SER19 | 7.01 | 0.04 | 0 | 1 | HH | H1 |
| LYS417 | ASN33 | 7.04 | 0.06 | 1 | 0 | HE2 | HB1 |
| PHE486 | GLU75 | 7.04 | -0.14 | 0 | -1 | HD1 | OE2 |
| ARG403 | ASN33 | 7.05 | -0.14 | 1 | 0 | HH12 | O |
| GLY502 | MET383 | 7.10 | -0.02 | 0 | 0 | HA2 | HE2 |
| PHE486 | ALA25 | 7.11 | -0.05 | 0 | 0 | HZ | HA |
| PHE456 | GLN24 | 7.11 | -0.04 | 0 | 0 | HE1 | O |
| GLY476 | GLU23 | 7.12 | 0.05 | 0 | -1 | HA1 | HB1 |
| ASN448 | ASP38 | 7.14 | 0.12 | 0 | -1 | O | OD1 |
| TYR473 | LYS26 | 7.15 | 0.05 | 0 | 1 | HE2 | C |
| LYS444 | GLN42 | 7.15 | -0.06 | 1 | 0 | O | HE22 |
| VAL503 | PHE327 | 7.17 | -0.01 | 0 | 0 | H | H |
| ASN501 | GLU329 | 7.18 | 0.10 | 0 | -1 | O | OE2 |
| GLN498 | LEU351 | 7.18 | -0.08 | 0 | 0 | HG1 | HD22 |
| GLY485 | GLU75 | 7.20 | -0.08 | 0 | -1 | HA1 | OE2 |
| TYR489 | ALA80 | 7.21 | -0.02 | 0 | 0 | HH | HA |
| PRO507 | LYS353 | 7.23 | -0.04 | 0 | 1 | HD2 | HD2 |
| GLY446 | ASN49 | 7.25 | -0.02 | 0 | 0 | H | HD22 |
| TYR489 | MET82 | 7.25 | -0.03 | 0 | 0 | HH | HB1 |
| GLN506 | PHE327 | 7.26 | -0.03 | 0 | 0 | HE21 | H |
| TYR449 | GLU35 | 7.27 | -0.09 | 0 | -1 | HH | O |
| TYR489 | GLU75 | 7.29 | -0.06 | 0 | -1 | HE1 | HG2 |
| GLY504 | ASP355 | 7.30 | -0.13 | 0 | -1 | H | N |
| TYR449 | HIS34 | 7.32 | -0.05 | 0 | 0 | HE1 | O |
| PHE486 | LEU97 | 7.32 | -0.03 | 0 | 0 | HE1 | HD23 |
| LYS444 | LEU45 | 7.32 | -0.03 | 1 | 0 | O | HD21 |
| ALA475 | ASP30 | 7.33 | 0.00 | 0 | -1 | HB3 | HB2 |
| GLN498 | LEU39 | 7.34 | -0.04 | 0 | 0 | HE22 | N |
| ASP420 | ASP30 | 7.36 | 1.22 | -1 | -1 | OD2 | OD2 |
| GLY485 | GLN76 | 7.36 | -0.03 | 0 | 0 | O | HE22 |
| LYS417 | LEU29 | 7.37 | 0.34 | 1 | 0 | HZ1 | C |
| ALA475 | LEU29 | 7.39 | -0.04 | 0 | 0 | HB2 | H |
| ASN501 | GLU37 | 7.39 | -0.12 | 0 | -1 | HD22 | O |
| PHE486 | SER77 | 7.40 | -0.04 | 0 | 0 | HE1 | O |
| ASN487 | PRO84 | 7.42 | -0.01 | 0 | 0 | HD22 | HD1 |
| ILE472 | LYS31 | 7.46 | 0.07 | 0 | 1 | HG21 | HE1 |
| GLY504 | ALA386 | 7.53 | -0.01 | 0 | 0 | HA2 | HB2 |
| ASN501 | GLN42 | 7.54 | -0.07 | 0 | 0 | HD22 | HG1 |
| THR500 | SER331 | 7.56 | -0.07 | 0 | 0 | HG21 | N |
| ASN487 | THR20 | 7.56 | 0.02 | 0 | 0 | OD1 | O |
| ALA475 | GLU22 | 7.57 | -0.02 | 0 | -1 | HB2 | O |
| TYR505 | THR324 | 7.57 | -0.02 | 0 | 0 | HD1 | HG21 |
| GLU484 | GLN76 | 7.57 | -0.06 | -1 | 0 | HB2 | HE22 |
| GLY502 | ARG357 | 7.58 | -0.08 | 0 | 1 | H | HH11 |
| GLY446 | ARG357 | 7.58 | 0.03 | 0 | 1 | H | HH12 |
| PHE497 | GLN42 | 7.60 | -0.04 | 0 | 0 | H | HE22 |
| LEU455 | LYS26 | 7.63 | -0.06 | 0 | 1 | HD13 | O |
| PHE456 | ALA25 | 7.64 | -0.02 | 0 | 0 | HZ | O |
| GLN474 | GLU23 | 7.64 | 0.03 | 0 | -1 | O | HB2 |
| PHE486 | LYS31 | 7.64 | 0.00 | 0 | 1 | HA | HG1 |
| GLY502 | LEU351 | 7.65 | -0.01 | 0 | 0 | H | HB1 |
| TYR421 | HIS34 | 7.65 | -0.05 | 0 | 0 | HE2 | HE1 |
| SER477 | TYR83 | 7.67 | -0.02 | 0 | 0 | H | HE2 |
| SER494 | GLN42 | 7.68 | -0.03 | 0 | 0 | HB2 | HE21 |
| ASN448 | TYR41 | 7.69 | -0.03 | 0 | 0 | H | HE2 |
| GLU484 | LEU79 | 7.69 | -0.07 | -1 | 0 | HB2 | HD11 |
| PRO491 | THR27 | 7.70 | 0.01 | 0 | 0 | HA | HG23 |
| VAL503 | MET383 | 7.71 | -0.03 | 0 | 0 | H | HE2 |
| TYR505 | ASP350 | 7.72 | -0.01 | 0 | -1 | HE1 | OD1 |
| ASN487 | GLN76 | 7.73 | -0.02 | 0 | 0 | H | HE22 |
| GLN498 | ASN49 | 7.73 | 0.00 | 0 | 0 | HG1 | HD22 |
| GLY476 | ALA25 | 7.73 | -0.02 | 0 | 0 | HA1 | H |
| LEU455 | ALA36 | 7.76 | -0.01 | 0 | 0 | HD21 | H |
| ARG454 | ASP30 | 7.78 | -1.00 | 1 | -1 | O | OD2 |
| ASN437 | GLN325 | 7.78 | -0.03 | 0 | 0 | HD22 | HB2 |
| LEU492 | HIS34 | 7.79 | -0.07 | 0 | 0 | C | HB2 |
| PHE486 | GLN101 | 7.80 | -0.01 | 0 | 0 | HZ | HE21 |
| TYR449 | PHE40 | 7.82 | 0.00 | 0 | 0 | HH | H |
| TYR505 | MET383 | 7.82 | -0.03 | 0 | 0 | HE1 | HA |
| LYS458 | THR27 | 7.83 | -0.05 | 1 | 0 | HA | HG21 |
| TYR489 | PHE72 | 7.83 | -0.03 | 0 | 0 | HE1 | HE1 |
| PRO499 | LYS353 | 7.87 | -0.14 | 0 | 1 | N | HD2 |
| GLN409 | HIS34 | 7.87 | -0.03 | 0 | 0 | HE22 | NE2 |
| GLN498 | SER43 | 7.87 | -0.03 | 0 | 0 | HE22 | H |
| ASN487 | LYS26 | 7.87 | -0.16 | 0 | 1 | OD1 | H |
| SER443 | LYS353 | 7.88 | 0.13 | 0 | 1 | HB1 | HZ1 |
| CYS488 | GLN76 | 7.88 | -0.02 | 0 | 0 | O | HE22 |
| ASN487 | SER19 | 7.90 | -0.20 | 0 | 1 | OD1 | H2 |
| ARG403 | PRO389 | 7.91 | -0.05 | 1 | 0 | HH12 | HA |
| GLY485 | MET82 | 7.95 | -0.15 | 0 | 0 | C | HE1 |
| GLN474 | TYR83 | 7.99 | 0.00 | 0 | 0 | C | HH |

**Supplementary table 3**. Interactions between SARS-CoV-2 RBD e *Rhinolophus sinicus* ACE2

| **Interaction** | **SARS-CoV-2  RBD** | ***Rhinolophus sinicus*  ACE2** |
| --- | --- | --- |
|  | Residue | Residue |
| Hydrophobic | PHE456 | MET27 |
|  | TYR473 | MET27 |
|  | ALA475 | MET27 |
|  | TYR489 | PHE28 |
|  | PHE486 | LEU79 |
|  | PHE486 | TYR83 |
| Hydrogen bonds | GLY502 | LYS353 |
|  | PHE490 | LYS31 |
|  | GLY496 | LYS353 |
|  | THR500 | ASP355 |
|  | GLN493 | LYS31 |
|  | GLN493 | LYS35 |
|  | GLN498 | GLN42 |
|  | ASN487 | TYR83 |
|  | TYR489 | TYR83 |
|  | TYR505 | ARG393 |
|  | TYR449 | ASP38 |
|  | ASN487 | TYR83 |
|  | TYR489 | TYR83 |
|  | GLN498 | GLN42 |
|  | TYR505 | GLU37 |
| Ionic | LYS417 | ASP30 |
|  | GLU484 | LYS31 |
|  | ARG403 | GLU37 |
| Aromatic-Aromatic | TYR489 | PHE28 |
|  | PHE486 | TYR83 |
| Cation-Pi | PHE456 | LYS31 |

**Supplementary table 4**. Quantum biochemistry between SARS-CoV-2 RBD e *Rhinolophus sinicus* ACE2.

| **Amino acid residues** | | **Distance** | **Interaction energy** | **Residue charge** | |  | **Residue atom** | |
| --- | --- | --- | --- | --- | --- | --- | --- | --- |
| SARS-CoV-2  RBD | *Rhinolophus sinicus*  ACE2 | (Å) | (kcal mol-1) | SARS-CoV-2  RBD | *Rhinolophus sinicus*  ACE2 |  | SARS-CoV-2  RBD | *Rhinolophus sinicus*  ACE2 |
| LYS417 | ASP30 | 1.55 | -11.76 | 1 | -1 |  | HZ1 | OD2 |
| TYR505 | GLU37 | 1.63 | -10.03 | 0 | -1 |  | HH | OE1 |
| GLN498 | LYS353 | 1.81 | -5.61 | 0 | 1 |  | OE1 | HZ2 |
| ASN487 | TYR83 | 1.81 | -6.64 | 0 | 0 |  | OD1 | HH |
| GLN493 | LYS31 | 1.84 | -7.82 | 0 | 1 |  | OE1 | HZ1 |
| GLY502 | LYS353 | 1.84 | -5.33 | 0 | 1 |  | H | O |
| THR500 | ASP355 | 1.95 | -8.05 | 0 | -1 |  | HG1 | OD2 |
| THR500 | ARG357 | 1.97 | 1.99 | 0 | 1 |  | HG1 | HH11 |
| LEU455 | THR34 | 2.01 | -1.43 | 0 | 0 |  | HD13 | HG21 |
| PHE456 | LYS31 | 2.02 | -1.47 | 0 | 1 |  | HE2 | HD2 |
| PHE456 | ASP30 | 2.07 | -1.40 | 0 | -1 |  | HE1 | HB1 |
| PHE486 | ASN82 | 2.08 | -1.53 | 0 | 0 |  | HE1 | HB1 |
| ASN501 | LYS353 | 2.13 | -12.47 | 0 | 1 |  | HD22 | HD1 |
| GLU484 | LYS31 | 2.18 | -7.59 | -1 | 1 |  | OE1 | HZ3 |
| GLN498 | GLN42 | 2.19 | -0.58 | 0 | 0 |  | HE22 | HE22 |
| GLY496 | LYS353 | 2.22 | -2.20 | 0 | 1 |  | O | HZ2 |
| GLN498 | LEU45 | 2.22 | -1.46 | 0 | 0 |  | HG1 | HD12 |
| LEU455 | LYS31 | 2.33 | -1.64 | 0 | 1 |  | HD21 | HA |
| TYR505 | ARG393 | 2.33 | 1.62 | 0 | 1 |  | HH | HH22 |
| ASN501 | ASP355 | 2.34 | -1.92 | 0 | -1 |  | HA | HB1 |
| TYR489 | LEU79 | 2.35 | -0.33 | 0 | 0 |  | OH | HD23 |
| GLN493 | THR34 | 2.36 | -2.02 | 0 | 0 |  | HG1 | HB |
| ALA475 | GLU24 | 2.37 | -2.27 | 0 | -1 |  | O | HG1 |
| PHE486 | LEU79 | 2.38 | -3.50 | 0 | 0 |  | HD1 | HG |
| GLY502 | GLY354 | 2.38 | -2.96 | 0 | 0 |  | HA2 | HA2 |
| TYR489 | LYS31 | 2.40 | -2.09 | 0 | 1 |  | HB2 | HD2 |
| PHE490 | LYS31 | 2.42 | -2.34 | 0 | 1 |  | O | HZ2 |
| LEU455 | ASP30 | 2.43 | -1.94 | 0 | -1 |  | HD11 | HB2 |
| TYR505 | LYS353 | 2.44 | -7.38 | 0 | 1 |  | HB1 | O |
| PHE456 | MET27 | 2.44 | -2.28 | 0 | 0 |  | HD1 | HE1 |
| GLN493 | LYS35 | 2.59 | -2.60 | 0 | 1 |  | HE22 | HD2 |
| THR500 | ASN330 | 2.59 | -3.44 | 0 | 0 |  | HB | HD22 |
| TYR489 | TYR83 | 2.61 | -1.49 | 0 | 0 |  | HH | OH |
| TYR453 | THR34 | 2.67 | -2.32 | 0 | 0 |  | HH | HB |
| TYR449 | ASP38 | 2.67 | -0.85 | 0 | -1 |  | HE1 | OD2 |
| TYR489 | PHE28 | 2.71 | -2.06 | 0 | 0 |  | HH | HB1 |
| GLN498 | HIS41 | 2.79 | -1.46 | 0 | 0 |  | HG1 | HD2 |
| TYR421 | MET27 | 2.79 | -1.03 | 0 | 0 |  | OH | HE2 |
| ASN501 | HIS41 | 2.83 | -1.31 | 0 | 0 |  | HD22 | HE2 |
| TYR473 | MET27 | 2.84 | -2.86 | 0 | 0 |  | HE2 | HB2 |
| THR500 | LEU45 | 2.89 | -0.92 | 0 | 0 |  | HG1 | HD22 |
| GLY476 | GLU24 | 2.92 | -3.70 | 0 | -1 |  | HA1 | HG1 |
| ASN487 | LEU79 | 3.04 | -0.43 | 0 | 0 |  | H | HD23 |
| ALA475 | MET27 | 3.11 | -1.16 | 0 | 0 |  | HB3 | HB1 |
| TYR449 | GLN42 | 3.12 | -0.69 | 0 | 0 |  | OH | NE2 |
| PHE486 | TYR83 | 3.21 | -2.27 | 0 | 0 |  | HE1 | HE1 |
| GLN498 | ASP38 | 3.24 | -0.39 | 0 | -1 |  | HE22 | OD1 |
| ASN487 | GLU24 | 3.24 | -1.17 | 0 | -1 |  | HD22 | HB2 |
| TYR505 | GLY354 | 3.26 | -1.10 | 0 | 0 |  | HD1 | HA2 |
| GLY496 | ASP38 | 3.36 | -0.19 | 0 | -1 |  | HA2 | OD1 |
| THR500 | HIS41 | 3.52 | -0.13 | 0 | 0 |  | HG1 | HE2 |
| LYS417 | MET27 | 3.66 | -0.14 | 1 | 0 |  | HZ3 | HE2 |
| ARG457 | MET27 | 3.68 | -0.20 | 1 | 0 |  | H | HE2 |
| GLY502 | ASP355 | 3.72 | -1.03 | 0 | -1 |  | H | N |
| GLU484 | LYS35 | 3.73 | -5.11 | -1 | 1 |  | OE2 | HZ1 |
| GLY446 | GLN42 | 3.75 | -0.07 | 0 | 0 |  | O | OE1 |
| ASN501 | GLY352 | 3.83 | -0.25 | 0 | 0 |  | HD21 | O |
| GLY485 | LEU79 | 3.84 | -0.10 | 0 | 0 |  | O | HD12 |
| TYR449 | LYS353 | 3.84 | -0.39 | 0 | 1 |  | HE1 | HZ1 |
| TYR489 | MET27 | 3.98 | -0.66 | 0 | 0 |  | HD2 | O |
| ALA475 | TYR83 | 4.09 | -0.25 | 0 | 0 |  | HB2 | HH |
| GLY446 | LEU45 | 4.15 | -0.48 | 0 | 0 |  | HA1 | HD11 |
| ASN501 | GLY354 | 4.21 | -3.92 | 0 | 0 |  | HA | C |
| PHE456 | THR34 | 4.27 | -0.42 | 0 | 0 |  | HZ | HG1 |
| GLY502 | GLY326 | 4.29 | -0.06 | 0 | 0 |  | HA1 | HA2 |
| THR500 | GLY326 | 4.34 | -0.47 | 0 | 0 |  | O | HA2 |
| VAL503 | GLU325 | 4.35 | -0.54 | 0 | -1 |  | HG21 | HB1 |
| LEU455 | MET27 | 4.37 | -0.41 | 0 | 0 |  | O | HE2 |
| PHE497 | LYS353 | 4.40 | -1.61 | 0 | 1 |  | N | HZ2 |
| ASN487 | PHE28 | 4.42 | -0.48 | 0 | 0 |  | OD1 | HB1 |
| LYS417 | THR34 | 4.48 | -0.04 | 1 | 0 |  | HE2 | HG21 |
| GLY504 | GLY354 | 4.56 | -0.25 | 0 | 0 |  | H | HA2 |
| TYR489 | GLU24 | 4.59 | -0.26 | 0 | -1 |  | HE2 | O |
| ASN501 | LEU45 | 4.60 | -0.25 | 0 | 0 |  | HD22 | HD12 |
| PRO491 | LYS31 | 4.64 | -1.01 | 0 | 1 |  | N | HZ2 |
| THR500 | LEU351 | 4.64 | -0.23 | 0 | 0 |  | HG1 | HD22 |
| SER477 | GLU24 | 4.68 | -1.04 | 0 | -1 |  | H | OE2 |
| GLY502 | THR324 | 4.73 | -0.07 | 0 | 0 |  | HA1 | HB |
| TYR505 | ALA386 | 4.75 | 0.41 | 0 | 0 |  | HE1 | O |
| ASN501 | ASN330 | 4.76 | -0.88 | 0 | 0 |  | N | HD22 |
| GLY447 | GLN42 | 4.78 | 0.18 | 0 | 0 |  | HA2 | HE22 |
| PHE456 | PHE28 | 4.78 | -0.07 | 0 | 0 |  | HZ | HA |
| TYR421 | ASP30 | 4.80 | -0.16 | 0 | -1 |  | HE2 | OD2 |
| PHE456 | PHE32 | 4.82 | -0.23 | 0 | 0 |  | HZ | H |
| PHE486 | ALA80 | 4.83 | -0.23 | 0 | 0 |  | HE1 | HA |
| GLY496 | GLN42 | 4.84 | -0.17 | 0 | 0 |  | HA2 | HE22 |
| GLY502 | GLY352 | 4.86 | -0.10 | 0 | 0 |  | H | O |
| TYR495 | LYS353 | 4.88 | 0.68 | 0 | 1 |  | C | HZ3 |
| THR500 | GLY354 | 4.89 | -0.29 | 0 | 0 |  | O | O |
| LEU492 | LYS31 | 4.90 | -0.24 | 0 | 1 |  | H | HZ2 |
| VAL503 | GLY326 | 4.93 | -0.11 | 0 | 0 |  | HG23 | H |
| VAL503 | GLY354 | 4.93 | -0.03 | 0 | 0 |  | H | O |
| VAL503 | THR324 | 4.97 | -0.32 | 0 | 0 |  | H | HB |
| PHE456 | LYS26 | 5.04 | -0.22 | 0 | 1 |  | HE1 | O |
| ASN501 | GLY326 | 5.06 | -0.55 | 0 | 0 |  | O | HA2 |
| GLY502 | PHE356 | 5.10 | -0.26 | 0 | 0 |  | HA2 | HE2 |
| PRO499 | ASN330 | 5.12 | -0.24 | 0 | 0 |  | O | HD22 |
| THR500 | ASN329 | 5.13 | -0.30 | 0 | 0 |  | HG23 | HB1 |
| GLN506 | GLU325 | 5.25 | 0.05 | 0 | -1 |  | HE22 | HB1 |
| ALA475 | PHE28 | 5.25 | -0.19 | 0 | 0 |  | HB3 | HB1 |
| LEU455 | LYS35 | 5.27 | -0.13 | 0 | 1 |  | HD22 | HG1 |
| ASN501 | ARG357 | 5.28 | -0.39 | 0 | 1 |  | N | HH11 |
| THR500 | PHE356 | 5.31 | -0.17 | 0 | 0 |  | O | H |
| LYS417 | LYS26 | 5.32 | 0.79 | 1 | 1 |  | HZ1 | O |
| LYS458 | MET27 | 5.33 | -0.10 | 1 | 0 |  | HA | HE3 |
| GLN506 | GLY326 | 5.33 | -0.04 | 0 | 0 |  | HE22 | HA1 |
| ASN487 | ALA25 | 5.34 | 0.05 | 0 | 0 |  | OD1 | HA |
| ARG403 | GLU37 | 5.34 | -1.90 | 1 | -1 |  | HH12 | OE1 |
| ALA475 | ASP23 | 5.35 | -0.37 | 0 | -1 |  | HB3 | O |
| PRO499 | LEU45 | 5.40 | -0.22 | 0 | 0 |  | HD1 | HD12 |
| GLY447 | LYS353 | 5.40 | 0.07 | 0 | 1 |  | HA2 | HZ2 |
| GLY504 | LYS353 | 5.45 | -0.19 | 0 | 1 |  | H | O |
| CYS488 | LEU79 | 5.45 | -0.13 | 0 | 0 |  | H | HD23 |
| VAL445 | LYS61 | 5.45 | 0.01 | 0 | 1 |  | HG12 | HZ1 |
| SER494 | THR34 | 5.46 | -0.13 | 0 | 0 |  | O | O |
| TYR489 | ALA25 | 5.46 | -0.16 | 0 | 0 |  | HH | HA |
| THR500 | LYS353 | 5.48 | -0.49 | 0 | 1 |  | O | O |
| GLN506 | LYS353 | 5.49 | -0.38 | 0 | 1 |  | HG2 | O |
| SER494 | ASP38 | 5.50 | -0.55 | 0 | -1 |  | O | HB1 |
| ASN501 | GLN42 | 5.50 | -0.10 | 0 | 0 |  | HD22 | HE22 |
| ASN487 | THR21 | 5.51 | -0.07 | 0 | 0 |  | HD22 | HG23 |
| CYS488 | LYS31 | 5.51 | 0.26 | 0 | 1 |  | O | HE1 |
| GLN493 | ASP30 | 5.52 | -0.09 | 0 | -1 |  | HG1 | O |
| LEU455 | PHE32 | 5.54 | -0.22 | 0 | 0 |  | HD21 | N |
| PHE456 | LEU29 | 5.54 | -0.31 | 0 | 0 |  | HE1 | C |
| GLY476 | TYR83 | 5.54 | -0.11 | 0 | 0 |  | HA2 | HH |
| ASN487 | MET27 | 5.55 | -0.25 | 0 | 0 |  | OD1 | HB1 |
| TYR495 | ASP38 | 5.58 | -0.40 | 0 | -1 |  | HA | HB1 |
| ARG403 | LYS353 | 5.59 | 1.01 | 1 | 1 |  | HD1 | HD2 |
| ASN501 | LEU351 | 5.62 | -0.10 | 0 | 0 |  | HD21 | HB1 |
| TYR505 | SER387 | 5.62 | -0.09 | 0 | 0 |  | HE1 | HA |
| GLN498 | ASP355 | 5.66 | -0.18 | 0 | -1 |  | HB2 | OD2 |
| PHE486 | LYS78 | 5.67 | -0.06 | 0 | 1 |  | HD1 | O |
| TYR473 | GLU24 | 5.67 | -0.13 | 0 | -1 |  | HE2 | HA |
| VAL503 | LYS353 | 5.67 | 0.21 | 0 | 1 |  | N | O |
| ARG403 | THR34 | 5.68 | -0.13 | 1 | 0 |  | HH11 | HG22 |
| TYR489 | GLN76 | 5.69 | -0.16 | 0 | 0 |  | HE1 | OE1 |
| ALA475 | ALA25 | 5.71 | -0.02 | 0 | 0 |  | HB3 | N |
| TYR505 | THR34 | 5.72 | -0.18 | 0 | 0 |  | HE2 | HA |
| VAL445 | LEU45 | 5.76 | -0.32 | 0 | 0 |  | HG13 | HD23 |
| GLY447 | ASP38 | 5.77 | -0.06 | 0 | -1 |  | HA2 | OD1 |
| GLY502 | ASN330 | 5.78 | -0.06 | 0 | 0 |  | HA1 | HD22 |
| GLY496 | HIS41 | 5.80 | -0.11 | 0 | 0 |  | O | HD2 |
| PHE486 | LYS81 | 5.80 | -0.07 | 0 | 1 |  | HE1 | C |
| TYR505 | ASN33 | 5.82 | -0.27 | 0 | 0 |  | HH | O |
| GLN506 | ASN330 | 5.83 | 0.01 | 0 | 0 |  | HE21 | HD22 |
| ARG408 | SER387 | 5.84 | -0.06 | 1 | 0 |  | HH12 | HB1 |
| TYR505 | GLY352 | 5.87 | -0.14 | 0 | 0 |  | HB1 | O |
| LEU455 | ASN33 | 5.89 | -0.26 | 0 | 0 |  | HD11 | HB1 |
| ARG403 | SER387 | 5.89 | -0.11 | 1 | 0 |  | HH22 | HA |
| PHE456 | LYS35 | 5.90 | -0.14 | 0 | 1 |  | HZ | HG1 |
| PHE490 | LYS35 | 5.90 | -0.13 | 0 | 1 |  | HB2 | HD2 |
| GLN498 | ARG357 | 5.94 | 0.02 | 0 | 1 |  | HB2 | HH12 |
| TYR453 | LYS35 | 5.94 | -0.28 | 0 | 1 |  | HH | N |
| ARG403 | ARG393 | 5.94 | 1.43 | 1 | 1 |  | HH12 | HH22 |
| GLY502 | HIS41 | 5.94 | -0.11 | 0 | 0 |  | H | HE1 |
| PRO499 | GLY326 | 5.96 | -0.06 | 0 | 0 |  | O | HA1 |
| GLY502 | PHE327 | 5.97 | -0.01 | 0 | 0 |  | HA1 | H |
| TYR505 | ASP355 | 5.99 | -0.23 | 0 | -1 |  | HB1 | H |
| SER494 | LYS35 | 6.00 | -0.17 | 0 | 1 |  | H | HD1 |
| TYR449 | LEU45 | 6.01 | -0.06 | 0 | 0 |  | HH | HD13 |
| TYR449 | HIS41 | 6.03 | -0.23 | 0 | 0 |  | OH | HD2 |
| TYR473 | LYS31 | 6.03 | -0.27 | 0 | 1 |  | HB2 | HD2 |
| CYS488 | TYR83 | 6.07 | -0.16 | 0 | 0 |  | H | HH |
| LYS417 | LYS31 | 6.07 | 0.71 | 1 | 1 |  | HZ1 | N |
| GLN493 | ASP38 | 6.09 | -0.40 | 0 | -1 |  | HG2 | HB2 |
| TYR505 | PHE356 | 6.09 | -0.10 | 0 | 0 |  | HE1 | HZ |
| PHE486 | GLU24 | 6.10 | -0.22 | 0 | -1 |  | HZ | OE1 |
| SER494 | LYS31 | 6.11 | -0.14 | 0 | 1 |  | H | HZ1 |
| TYR505 | GLN388 | 6.13 | 0.01 | 0 | 0 |  | HH | O |
| ASN487 | ASN82 | 6.15 | -0.27 | 0 | 0 |  | HD22 | HB1 |
| TYR505 | TYR390 | 6.16 | -0.06 | 0 | 0 |  | HH | HD2 |
| PHE486 | THR21 | 6.17 | -0.06 | 0 | 0 |  | HZ | HG23 |
| TYR453 | ASP30 | 6.17 | -0.03 | 0 | -1 |  | OH | O |
| THR500 | GLY352 | 6.17 | -0.07 | 0 | 0 |  | O | O |
| PRO499 | HIS41 | 6.19 | -0.06 | 0 | 0 |  | HD1 | HE2 |
| LEU455 | LEU29 | 6.19 | -0.07 | 0 | 0 |  | HD21 | O |
| SER494 | LYS353 | 6.20 | -0.21 | 0 | 1 |  | O | HZ3 |
| GLY485 | LYS31 | 6.22 | -0.27 | 0 | 1 |  | O | HE1 |
| ASN501 | ASP38 | 6.23 | -0.13 | 0 | -1 |  | HD22 | HA |
| ASN501 | PHE356 | 6.23 | -0.24 | 0 | 0 |  | HA | H |
| PHE497 | ASP38 | 6.24 | 0.09 | 0 | -1 |  | N | OD1 |
| GLY476 | THR21 | 6.24 | -0.08 | 0 | 0 |  | HA1 | HA |
| GLY502 | GLU325 | 6.24 | 0.30 | 0 | -1 |  | HA1 | H |
| GLY446 | LYS61 | 6.25 | 0.05 | 0 | 1 |  | HA1 | HZ1 |
| GLY446 | ASP38 | 6.25 | 0.07 | 0 | -1 |  | O | OD1 |
| GLY447 | LEU45 | 6.25 | -0.07 | 0 | 0 |  | N | HD11 |
| GLY446 | HIS41 | 6.28 | -0.14 | 0 | 0 |  | O | HD2 |
| PHE486 | PHE28 | 6.28 | -0.28 | 0 | 0 |  | HE1 | HB1 |
| GLN506 | GLY354 | 6.28 | -0.11 | 0 | 0 |  | HE21 | O |
| PHE456 | ASN33 | 6.31 | -0.09 | 0 | 0 |  | HZ | H |
| TYR453 | GLU37 | 6.32 | -0.11 | 0 | -1 |  | HH | HB2 |
| PRO499 | ASP355 | 6.34 | -1.02 | 0 | -1 |  | C | OD2 |
| THR500 | PHE327 | 6.35 | -0.12 | 0 | 0 |  | O | H |
| ASN448 | LYS353 | 6.37 | -0.12 | 0 | 1 |  | H | HZ2 |
| TYR473 | PHE28 | 6.43 | -0.10 | 0 | 0 |  | HE2 | N |
| TYR489 | ALA80 | 6.44 | -0.06 | 0 | 0 |  | HH | HA |
| TYR473 | ASP23 | 6.45 | -0.03 | 0 | -1 |  | HE2 | O |
| GLN474 | MET27 | 6.45 | -0.08 | 0 | 0 |  | O | HG2 |
| PRO499 | ARG357 | 6.45 | 0.63 | 0 | 1 |  | HG1 | HH12 |
| ILE472 | LYS31 | 6.45 | 0.19 | 0 | 1 |  | HG23 | HZ2 |
| GLN506 | THR324 | 6.46 | -0.02 | 0 | 0 |  | HE22 | HB |
| GLN506 | ASP355 | 6.46 | -0.15 | 0 | -1 |  | HE21 | HB1 |
| TYR489 | LEU29 | 6.46 | -0.24 | 0 | 0 |  | HE2 | N |
| GLU484 | LEU79 | 6.46 | -0.11 | -1 | 0 |  | HG1 | HD12 |
| LEU455 | PHE28 | 6.48 | -0.12 | 0 | 0 |  | HD21 | O |
| GLN498 | GLU37 | 6.48 | 0.08 | 0 | -1 |  | OE1 | O |
| TYR505 | PRO389 | 6.48 | -0.02 | 0 | 0 |  | HH | HA |
| TYR449 | LEU39 | 6.49 | -0.10 | 0 | 0 |  | HE1 | N |
| VAL445 | ASP49 | 6.52 | -0.20 | 0 | -1 |  | HG13 | OD2 |
| VAL503 | ASP355 | 6.52 | 0.07 | 0 | -1 |  | H | HA |
| TYR449 | LYS68 | 6.52 | -0.22 | 0 | 1 |  | OH | HZ2 |
| PHE486 | SER84 | 6.52 | -0.07 | 0 | 0 |  | HZ | H |
| ALA475 | THR20 | 6.53 | -0.21 | 0 | 1 |  | O | O |
| TYR495 | THR34 | 6.53 | -0.15 | 0 | 0 |  | HA | O |
| THR500 | TRP48 | 6.54 | -0.10 | 0 | 0 |  | HG22 | HE1 |
| GLY446 | LYS353 | 6.54 | -0.04 | 0 | 1 |  | O | HZ2 |
| TYR453 | ASN33 | 6.55 | -0.09 | 0 | 0 |  | HH | C |
| GLY447 | HIS41 | 6.56 | -0.05 | 0 | 0 |  | HA2 | HD2 |
| ARG403 | ALA386 | 6.59 | -0.29 | 1 | 0 |  | HH22 | O |
| ASN501 | GLU37 | 6.59 | -0.35 | 0 | -1 |  | HD22 | O |
| GLU484 | GLU75 | 6.62 | 1.98 | -1 | -1 |  | OE2 | OE2 |
| GLN493 | ASN33 | 6.64 | 0.00 | 0 | 0 |  | HG1 | C |
| GLY476 | THR20 | 6.66 | 0.06 | 0 | 1 |  | HA1 | H1 |
| PHE497 | GLN42 | 6.66 | -0.05 | 0 | 0 |  | N | HE22 |
| TYR453 | ASP38 | 6.67 | -0.07 | 0 | -1 |  | HH | HB1 |
| LYS417 | ASN33 | 6.67 | 0.16 | 1 | 0 |  | HE2 | HB1 |
| TYR489 | ASN82 | 6.68 | -0.05 | 0 | 0 |  | OH | HD21 |
| LYS417 | LEU29 | 6.70 | 0.42 | 1 | 0 |  | HZ1 | C |
| ASP405 | GLY354 | 6.71 | 0.00 | -1 | 0 |  | OD2 | HA2 |
| TYR489 | LYS35 | 6.72 | -0.03 | 0 | 1 |  | HD1 | HD2 |
| GLN474 | GLU24 | 6.74 | -0.36 | 0 | -1 |  | O | HG1 |
| ASP405 | SER387 | 6.74 | -0.07 | -1 | 0 |  | OD2 | HA |
| GLY476 | MET27 | 6.74 | -0.08 | 0 | 0 |  | N | HB1 |
| TYR489 | GLU75 | 6.76 | -0.13 | 0 | -1 |  | HE1 | HG1 |
| GLU406 | THR34 | 6.78 | -0.07 | -1 | 0 |  | OE2 | HG22 |
| ASN487 | LYS31 | 6.78 | -0.06 | 0 | 1 |  | HA | HG1 |
| THR500 | GLU325 | 6.78 | -0.15 | 0 | -1 |  | HA | O |
| ALA475 | THR21 | 6.78 | -0.01 | 0 | 0 |  | O | HA |
| TYR505 | ASP38 | 6.80 | -0.15 | 0 | -1 |  | HE2 | N |
| GLN493 | PHE32 | 6.80 | -0.08 | 0 | 0 |  | OE1 | N |
| ALA475 | LYS31 | 6.82 | 0.00 | 0 | 1 |  | HB1 | HB1 |
| ASN501 | THR324 | 6.84 | -0.12 | 0 | 0 |  | O | HB |
| ALA475 | LYS26 | 6.84 | -0.14 | 0 | 1 |  | HB3 | N |
| TYR489 | ASP30 | 6.84 | -0.08 | 0 | -1 |  | HD2 | HB1 |
| GLY485 | GLU75 | 6.85 | 0.02 | 0 | -1 |  | HA1 | OE2 |
| GLY504 | THR324 | 6.88 | -0.01 | 0 | 0 |  | H | HG22 |
| PHE486 | GLU75 | 6.89 | -0.10 | 0 | -1 |  | HD1 | O |
| ASN439 | ASN330 | 6.89 | -0.01 | 0 | 0 |  | HD22 | HD22 |
| GLY496 | THR34 | 6.89 | -0.07 | 0 | 0 |  | H | O |
| VAL503 | PHE356 | 6.91 | -0.05 | 0 | 0 |  | H | HE2 |
| TYR489 | PHE32 | 6.92 | -0.18 | 0 | 0 |  | CE2 | H |
| LYS444 | LEU45 | 6.92 | -0.01 | 1 | 0 |  | O | HD12 |
| GLU484 | GLN76 | 6.93 | -0.12 | -1 | 0 |  | OE2 | HE22 |
| TYR453 | LYS31 | 6.94 | -0.21 | 0 | 1 |  | OH | HA |
| PHE497 | HIS41 | 6.94 | -0.05 | 0 | 0 |  | C | HD2 |
| VAL445 | ARG357 | 6.95 | 0.06 | 0 | 1 |  | HG13 | HH12 |
| GLN498 | GLY352 | 6.96 | -0.08 | 0 | 0 |  | HB1 | O |
| ARG403 | GLY354 | 6.97 | -0.06 | 1 | 0 |  | HH21 | HA2 |
| GLY504 | PHE356 | 6.97 | -0.03 | 0 | 0 |  | H | HE2 |
| CYS488 | PHE28 | 6.99 | -0.10 | 0 | 0 |  | H | HB1 |
| ASP405 | LYS353 | 7.03 | -0.91 | -1 | 1 |  | OD2 | HA |
| SER459 | MET27 | 7.03 | -0.05 | 0 | 0 |  | N | HE3 |
| PHE486 | ALA25 | 7.07 | -0.06 | 0 | 0 |  | HE1 | HA |
| GLN493 | PHE72 | 7.07 | -0.02 | 0 | 0 |  | HE22 | HE1 |
| GLN493 | ALA36 | 7.09 | -0.08 | 0 | 0 |  | HG1 | H |
| ASN487 | ASP23 | 7.11 | 0.06 | 0 | -1 |  | OD1 | O |
| PRO499 | ASN329 | 7.11 | -0.06 | 0 | 0 |  | O | HB1 |
| PHE486 | GLN76 | 7.11 | -0.15 | 0 | 0 |  | HD1 | HA |
| ASN501 | GLU325 | 7.14 | -0.32 | 0 | -1 |  | O | C |
| ASN448 | GLN42 | 7.16 | -0.05 | 0 | 0 |  | H | HE22 |
| GLY496 | GLU37 | 7.16 | -0.06 | 0 | -1 |  | H | HG1 |
| GLY485 | PHE28 | 7.17 | -0.02 | 0 | 0 |  | O | HD1 |
| LEU492 | LYS35 | 7.17 | -0.15 | 0 | 1 |  | O | HD2 |
| PHE456 | ALA25 | 7.18 | -0.02 | 0 | 0 |  | HE1 | O |
| VAL503 | PHE327 | 7.18 | -0.03 | 0 | 0 |  | H | H |
| VAL445 | GLN42 | 7.18 | -0.30 | 0 | 0 |  | C | OE1 |
| ALA475 | LEU79 | 7.19 | -0.02 | 0 | 0 |  | HB2 | HD23 |
| ASN439 | GLY326 | 7.22 | -0.02 | 0 | 0 |  | HD22 | HA1 |
| GLN498 | LEU39 | 7.22 | -0.04 | 0 | 0 |  | HE22 | N |
| ASP420 | MET27 | 7.24 | 0.02 | -1 | 0 |  | HB2 | HE2 |
| GLN506 | ASN329 | 7.24 | 0.01 | 0 | 0 |  | HE22 | HD21 |
| ASN501 | PHE327 | 7.27 | -0.04 | 0 | 0 |  | O | H |
| THR478 | GLU24 | 7.28 | 0.10 | 0 | -1 |  | H | OE1 |
| THR500 | THR324 | 7.29 | -0.05 | 0 | 0 |  | O | HB |
| TYR505 | HIS41 | 7.29 | -0.11 | 0 | 0 |  | HB1 | HE1 |
| GLY496 | LEU45 | 7.29 | -0.02 | 0 | 0 |  | O | HD12 |
| TYR473 | LYS26 | 7.31 | -0.07 | 0 | 1 |  | HE2 | C |
| PHE497 | LEU45 | 7.33 | -0.03 | 0 | 0 |  | C | HD12 |
| GLN498 | ASN330 | 7.34 | -0.09 | 0 | 0 |  | O | HD22 |
| PRO499 | LYS353 | 7.34 | -0.10 | 0 | 1 |  | N | HD1 |
| ARG403 | ASN33 | 7.34 | -0.13 | 1 | 0 |  | HH12 | HB2 |
| GLY476 | ALA25 | 7.35 | -0.03 | 0 | 0 |  | HA1 | N |
| LYS417 | PHE28 | 7.35 | -0.22 | 1 | 0 |  | HZ1 | N |
| TYR473 | ASP30 | 7.36 | 0.00 | 0 | -1 |  | HE2 | HB1 |
| LEU455 | LYS26 | 7.36 | -0.06 | 0 | 1 |  | HD11 | O |
| PRO507 | LYS353 | 7.37 | -0.04 | 0 | 1 |  | HD2 | HD2 |
| GLN493 | LEU39 | 7.38 | -0.06 | 0 | 0 |  | NE2 | HD11 |
| PHE486 | LYS31 | 7.38 | -0.07 | 0 | 1 |  | HA | HG1 |
| TYR489 | LYS26 | 7.39 | -0.04 | 0 | 1 |  | HE2 | C |
| TYR453 | LYS353 | 7.41 | 0.06 | 0 | 1 |  | HE1 | HZ3 |
| GLY485 | TYR83 | 7.43 | -0.02 | 0 | 0 |  | O | HH |
| GLY502 | LEU351 | 7.43 | -0.01 | 0 | 0 |  | H | HB1 |
| THR500 | SER331 | 7.43 | -0.07 | 0 | 0 |  | HB | N |
| ASP405 | ALA386 | 7.44 | 0.04 | -1 | 0 |  | OD2 | O |
| GLY504 | ASP355 | 7.46 | -0.07 | 0 | -1 |  | H | N |
| TYR495 | GLU37 | 7.48 | -0.09 | 0 | -1 |  | HD1 | HG1 |
| GLY476 | ASP23 | 7.49 | 0.07 | 0 | -1 |  | HA1 | C |
| GLU484 | PHE72 | 7.51 | 0.03 | -1 | 0 |  | OE2 | HE1 |
| SER477 | THR20 | 7.52 | 0.01 | 0 | 1 |  | HB2 | H1 |
| VAL445 | HIS41 | 7.54 | -0.02 | 0 | 0 |  | HA | HD2 |
| ASN448 | ASP38 | 7.55 | -0.04 | 0 | -1 |  | O | OD1 |
| GLY446 | ALA46 | 7.56 | -0.04 | 0 | 0 |  | HA1 | H |
| VAL503 | ASN330 | 7.58 | -0.01 | 0 | 0 |  | H | HD22 |
| SER477 | TYR83 | 7.59 | -0.04 | 0 | 0 |  | H | HE2 |
| TYR473 | TYR83 | 7.60 | -0.02 | 0 | 0 |  | HE2 | HH |
| GLY485 | GLN76 | 7.60 | -0.06 | 0 | 0 |  | O | HE22 |
| LYS444 | GLN42 | 7.60 | -0.13 | 1 | 0 |  | O | HE22 |
| ASN487 | THR20 | 7.60 | -0.02 | 0 | 1 |  | HD22 | O |
| GLN493 | GLU37 | 7.61 | -0.03 | 0 | -1 |  | HG1 | HB2 |
| ARG454 | THR34 | 7.61 | 0.02 | 1 | 0 |  | O | HG21 |
| GLY502 | ARG357 | 7.62 | -0.10 | 0 | 1 |  | H | HH11 |
| TYR489 | ASP23 | 7.63 | -0.05 | 0 | -1 |  | HE2 | O |
| PHE486 | LEU97 | 7.63 | -0.02 | 0 | 0 |  | HE1 | HD21 |
| PHE456 | GLU24 | 7.64 | -0.05 | 0 | -1 |  | HE1 | O |
| LEU492 | THR34 | 7.68 | -0.04 | 0 | 0 |  | C | HB |
| GLY446 | ASP49 | 7.70 | -0.16 | 0 | -1 |  | HA1 | OD2 |
| GLN506 | PHE327 | 7.70 | -0.02 | 0 | 0 |  | HE22 | H |
| GLY502 | MET383 | 7.71 | -0.02 | 0 | 0 |  | HA2 | HG1 |
| ASN501 | SER44 | 7.71 | 0.01 | 0 | 0 |  | HD21 | HB1 |
| ARG454 | MET27 | 7.71 | -0.07 | 1 | 0 |  | HG1 | HE2 |
| TYR449 | GLU37 | 7.72 | -0.05 | 0 | -1 |  | HE1 | C |
| GLN498 | LEU351 | 7.73 | -0.04 | 0 | 0 |  | HB2 | HD22 |
| LYS444 | LYS353 | 7.74 | 0.67 | 1 | 1 |  | O | HZ2 |
| ALA475 | LEU29 | 7.74 | -0.03 | 0 | 0 |  | HB3 | H |
| GLY416 | ASP30 | 7.74 | -0.29 | 0 | -1 |  | HA1 | OD2 |
| TYR505 | ASP350 | 7.75 | -0.05 | 0 | -1 |  | HE1 | OD1 |
| ASP420 | ASP30 | 7.75 | 1.23 | -1 | -1 |  | OD2 | OD2 |
| ASN487 | LYS26 | 7.76 | -0.09 | 0 | 1 |  | OD1 | N |
| TYR449 | LYS35 | 7.76 | -0.13 | 0 | 1 |  | HE1 | HA |
| ASN487 | SER84 | 7.76 | 0.01 | 0 | 0 |  | HD22 | H |
| GLY496 | LEU39 | 7.80 | 0.00 | 0 | 0 |  | HA2 | N |
| SER443 | LYS353 | 7.82 | 0.10 | 0 | 1 |  | HB1 | HZ2 |
| LYS444 | HIS41 | 7.83 | 0.00 | 1 | 0 |  | O | HD2 |
| THR500 | SER44 | 7.84 | -0.03 | 0 | 0 |  | HG1 | HB1 |
| PRO491 | MET27 | 7.85 | 0.00 | 0 | 0 |  | HA | HE1 |
| GLN474 | TYR83 | 7.86 | -0.01 | 0 | 0 |  | C | HH |
| ARG457 | ASP30 | 7.86 | -0.63 | 1 | -1 |  | H | OD2 |
| GLN498 | ALA46 | 7.87 | -0.04 | 0 | 0 |  | HG1 | H |
| PRO499 | GLU325 | 7.87 | 0.08 | 0 | -1 |  | O | O |
| GLY446 | ARG357 | 7.88 | 0.09 | 0 | 1 |  | H | HH12 |
| ASN439 | GLU325 | 7.88 | -0.14 | 0 | -1 |  | HD21 | HB1 |
| TYR495 | GLN42 | 7.89 | -0.01 | 0 | 0 |  | C | HE21 |
| SER477 | THR21 | 7.89 | -0.04 | 0 | 0 |  | H | HA |
| TYR489 | THR34 | 7.89 | -0.02 | 0 | 0 |  | HB2 | HG1 |
| LEU455 | ALA36 | 7.90 | -0.02 | 0 | 0 |  | HD21 | H |
| PRO499 | GLN42 | 7.90 | -0.07 | 0 | 0 |  | HD1 | HE22 |
| ASN501 | ASN329 | 7.92 | -0.13 | 0 | 0 |  | O | HB1 |
| PHE486 | SER77 | 7.93 | -0.04 | 0 | 0 |  | HE1 | O |
| ARG454 | LYS31 | 7.93 | 0.98 | 1 | 1 |  | HA | HZ2 |
| ALA475 | ASP30 | 7.96 | 0.03 | 0 | -1 |  | HB1 | HB1 |
| TYR449 | THR34 | 7.96 | -0.03 | 0 | 0 |  | HE1 | O |
| TYR421 | LYS26 | 7.96 | -0.06 | 0 | 1 |  | HE2 | O |
| TYR489 | PHE72 | 7.97 | -0.02 | 0 | 0 |  | HE1 | HE1 |
| VAL503 | ASN329 | 7.99 | 0.00 | 0 | 0 |  | HG23 | HD21 |

**Supplementary table 5**. Interactions between SARS-CoV-2 RBD e *Gorilla gorilla gorilla* ACE2.

| **Interaction** | **SARS-CoV-2  RBD** | ***Gorilla gorilla gorilla*  ACE2** |
| --- | --- | --- |
|  | Residue | Residue |
| Hydrophobic | TYR489 | PHE28 |
|  | PHE486 | LEU79 |
|  | PHE486 | MET82 |
|  | TYR505 | ALA386 |
| Hydrogen bonds | GLY502 | GLY354 |
|  | GLY496 | LYS353 |
|  | ASN501 | LYS353 |
|  | TYR505 | ALA386 |
|  | GLU484 | LYS31 |
|  | GLN493 | GLU35 |
|  | ASN487 | TYR83 |
|  | TYR449 | LYS353 |
|  | GLN498 | LYS353 |
|  | TYR505 | ARG393 |
|  | LYS417 | ASP30 |
|  | TYR449 | ASP38 |
|  | ANS487 | TYR83 |
|  | GLN493 | GLU35 |
|  | THR500 | TYR41 |
|  | THR500 | ASP355 |
| Ionic | LYS417 | ASP30 |
|  | GLU484 | LYS31 |
|  | ARG403 | GLU37 |
| Aromatic-Aromatic | PHE486 | TYR83 |
| Aromatic-Sulphur | PHE486 | MET82 |
| Cation-Pi | TYR449 | LYS353 |
|  | TYR489 | LYS31 |
|  | TYR505 | ARG393 |

**Supplementary table 6**. Quantum biochemistry between SARS-CoV-2 RBD e *Gorilla gorilla gorilla* ACE2.

| **Amino acid residues** | | **Distance** | **Interaction energy** | **Residue charge** | |  | **Residue atom** | |
| --- | --- | --- | --- | --- | --- | --- | --- | --- |
| SARS-CoV-2  RBD | *Gorilla gorilla gorilla* ACE2 | (Å) | (kcal mol-1) | SARS-CoV-2  RBD | *Gorilla gorilla gorilla* ACE2 |  | SARS-CoV-2  RBD | *Gorilla gorilla gorilla* ACE2 |
| LYS417 | ASP30 | 1.65 | -10.72 | 1 | -1 |  | HZ1 | OD2 |
| GLU484 | LYS31 | 1.78 | -11.01 | -1 | 1 |  | OE1 | HZ1 |
| THR500 | ASP355 | 1.81 | -9.70 | 0 | -1 |  | HG1 | OD2 |
| THR500 | ASN330 | 1.82 | 1.61 | 0 | 0 |  | HB | HD22 |
| TYR505 | ALA386 | 1.85 | -5.97 | 0 | 0 |  | HH | O |
| GLN498 | LYS353 | 1.89 | -6.86 | 0 | 1 |  | OE1 | HZ3 |
| ASN487 | TYR83 | 2.03 | -5.29 | 0 | 0 |  | OD1 | HH |
| PHE486 | LEU79 | 2.07 | -2.60 | 0 | 0 |  | HE1 | HD22 |
| THR500 | GLY326 | 2.14 | -2.12 | 0 | 0 |  | HA | HA1 |
| PHE456 | LYS31 | 2.17 | -2.37 | 0 | 1 |  | HZ | HB1 |
| PHE486 | MET82 | 2.21 | -2.50 | 0 | 0 |  | HE1 | HE2 |
| GLN493 | GLU35 | 2.23 | -4.13 | 0 | -1 |  | HE22 | OE2 |
| GLY502 | GLY354 | 2.26 | -0.38 | 0 | 0 |  | H | O |
| ASN501 | LYS353 | 2.26 | -4.40 | 0 | 1 |  | HD22 | HB1 |
| THR500 | TYR41 | 2.27 | 0.29 | 0 | 0 |  | HG1 | HH |
| VAL503 | THR324 | 2.30 | -0.95 | 0 | 0 |  | HG23 | HG23 |
| TYR449 | ASP38 | 2.31 | -3.15 | 0 | -1 |  | HH | OD1 |
| PHE456 | THR27 | 2.32 | -2.25 | 0 | 0 |  | HE1 | HA |
| TYR489 | LYS31 | 2.34 | -5.05 | 0 | 1 |  | HB2 | HD2 |
| GLY496 | LYS353 | 2.38 | -1.39 | 0 | 1 |  | HA2 | HZ1 |
| THR500 | ARG357 | 2.39 | 0.25 | 0 | 1 |  | HG1 | HH11 |
| GLN498 | TYR41 | 2.40 | -1.62 | 0 | 0 |  | OE1 | HE2 |
| PHE456 | ASP30 | 2.40 | -2.34 | 0 | -1 |  | HE1 | HB1 |
| ALA475 | GLN24 | 2.45 | -2.99 | 0 | 0 |  | O | HG1 |
| GLN493 | HIS34 | 2.46 | -3.25 | 0 | 0 |  | HG1 | HB2 |
| GLY502 | THR324 | 2.49 | -0.34 | 0 | 0 |  | HA2 | HG22 |
| TYR473 | THR27 | 2.55 | -2.19 | 0 | 0 |  | HH | HG23 |
| TYR489 | PHE28 | 2.57 | -1.54 | 0 | 0 |  | HE2 | HA |
| TYR505 | GLY354 | 2.62 | -1.89 | 0 | 0 |  | HD1 | HA2 |
| ASN501 | ASP355 | 2.64 | -4.38 | 0 | -1 |  | HD21 | HB1 |
| TYR505 | LYS353 | 2.67 | -4.04 | 0 | 1 |  | CD2 | HA |
| LEU455 | HIS34 | 2.69 | -2.98 | 0 | 0 |  | HD12 | ND1 |
| TYR453 | HIS34 | 2.73 | -2.00 | 0 | 0 |  | HH | HD2 |
| TYR449 | LYS353 | 2.73 | -0.21 | 0 | 1 |  | HH | HZ2 |
| LEU455 | ASP30 | 2.75 | -2.13 | 0 | -1 |  | HD23 | O |
| TYR449 | GLN42 | 2.77 | -0.59 | 0 | 0 |  | HH | HE21 |
| GLY502 | LYS353 | 2.88 | -1.25 | 0 | 1 |  | H | O |
| GLN498 | GLN42 | 2.90 | -0.48 | 0 | 0 |  | HE22 | HE22 |
| ALA475 | THR27 | 3.07 | -0.95 | 0 | 0 |  | HB2 | OG1 |
| ASN501 | GLY354 | 3.08 | -8.12 | 0 | 0 |  | HA | O |
| ARG403 | ALA387 | 3.10 | -0.24 | 1 | 0 |  | HH22 | HA |
| LEU455 | LYS31 | 3.10 | -0.88 | 0 | 1 |  | HD23 | HA |
| TYR489 | THR27 | 3.14 | -1.00 | 0 | 0 |  | HE2 | O |
| TYR505 | ARG393 | 3.16 | -1.61 | 0 | 1 |  | OH | NH2 |
| PHE490 | LYS31 | 3.21 | -0.51 | 0 | 1 |  | H | HZ1 |
| TYR489 | TYR83 | 3.24 | -0.28 | 0 | 0 |  | HH | HH |
| ASN501 | TYR41 | 3.26 | -1.22 | 0 | 0 |  | HD22 | CZ |
| GLN493 | LYS31 | 3.36 | -2.04 | 0 | 1 |  | HE22 | HZ2 |
| ASP405 | ALA387 | 3.37 | -0.98 | -1 | 0 |  | OD2 | HB2 |
| TYR505 | GLU37 | 3.40 | -0.31 | 0 | -1 |  | HE2 | OE1 |
| GLY502 | PHE356 | 3.42 | -0.54 | 0 | 0 |  | HA2 | HE2 |
| ARG408 | ALA387 | 3.49 | -0.60 | 1 | 0 |  | HH12 | HB3 |
| TYR505 | ALA387 | 3.55 | -2.54 | 0 | 0 |  | HH | HA |
| GLN498 | LEU45 | 3.62 | -0.49 | 0 | 0 |  | HG1 | HD12 |
| GLY502 | ASP355 | 3.66 | -0.69 | 0 | -1 |  | H | N |
| THR500 | GLY354 | 3.68 | 0.54 | 0 | 0 |  | O | O |
| GLY476 | GLN24 | 3.75 | -0.59 | 0 | 0 |  | HA1 | HG1 |
| TYR489 | LEU79 | 3.75 | -0.57 | 0 | 0 |  | OH | HD23 |
| ARG403 | ALA386 | 3.79 | 0.15 | 1 | 0 |  | HH22 | O |
| ASN487 | LEU79 | 3.89 | -0.53 | 0 | 0 |  | H | HD23 |
| THR500 | GLU329 | 3.94 | -0.84 | 0 | -1 |  | HG22 | HB1 |
| THR500 | PHE356 | 4.03 | -0.73 | 0 | 0 |  | O | H |
| ASN487 | GLN24 | 4.04 | -1.05 | 0 | 0 |  | HD22 | OE1 |
| LYS417 | THR27 | 4.05 | -0.21 | 1 | 0 |  | HZ1 | HG21 |
| ALA475 | SER19 | 4.06 | -3.33 | 0 | 1 |  | O | HB1 |
| GLN498 | ASP38 | 4.08 | -0.12 | 0 | -1 |  | HE22 | OD1 |
| GLN506 | GLN325 | 4.12 | -1.04 | 0 | 0 |  | HE22 | HB1 |
| PRO499 | GLY326 | 4.13 | -0.33 | 0 | 0 |  | O | HA1 |
| GLY446 | GLN42 | 4.15 | -0.11 | 0 | 0 |  | O | HE22 |
| ASN501 | GLY352 | 4.22 | -0.52 | 0 | 0 |  | HD21 | O |
| GLY502 | MET383 | 4.23 | -0.19 | 0 | 0 |  | HA2 | HE1 |
| THR500 | LEU45 | 4.26 | -0.22 | 0 | 0 |  | HG1 | HD22 |
| GLN506 | GLY326 | 4.29 | -0.03 | 0 | 0 |  | HE22 | H |
| TYR421 | THR27 | 4.33 | -0.22 | 0 | 0 |  | HH | HG21 |
| VAL503 | MET383 | 4.37 | -0.30 | 0 | 0 |  | HG23 | HE2 |
| GLN506 | THR324 | 4.39 | -0.15 | 0 | 0 |  | HE22 | HB |
| ALA475 | TYR83 | 4.40 | -0.24 | 0 | 0 |  | HB3 | HH |
| TYR505 | GLN388 | 4.43 | -0.20 | 0 | 0 |  | HH | O |
| LYS417 | HIS34 | 4.47 | -0.36 | 1 | 0 |  | HE2 | HE1 |
| ASN487 | PHE28 | 4.47 | -0.49 | 0 | 0 |  | OD1 | HB1 |
| ASN501 | GLY326 | 4.49 | -0.78 | 0 | 0 |  | O | HA2 |
| PHE497 | LYS353 | 4.52 | -0.64 | 0 | 1 |  | HA | HD2 |
| PHE456 | PHE28 | 4.53 | -0.24 | 0 | 0 |  | HE1 | N |
| GLY502 | GLY326 | 4.56 | -0.20 | 0 | 0 |  | HA1 | HA2 |
| PHE456 | LYS26 | 4.57 | -0.09 | 0 | 1 |  | HE1 | O |
| VAL503 | GLN325 | 4.60 | -0.53 | 0 | 0 |  | HG22 | H |
| TYR505 | PHE356 | 4.61 | -0.40 | 0 | 0 |  | HE1 | HZ |
| TYR449 | TYR41 | 4.62 | -0.32 | 0 | 0 |  | OH | HD2 |
| PHE486 | TYR83 | 4.73 | -1.00 | 0 | 0 |  | HE1 | OH |
| THR500 | PHE327 | 4.73 | -0.46 | 0 | 0 |  | O | H |
| ASN439 | GLN325 | 4.75 | -0.55 | 0 | 0 |  | HD21 | OE1 |
| TYR489 | GLN24 | 4.77 | -0.32 | 0 | 0 |  | HE2 | O |
| ASN501 | THR324 | 4.79 | -0.07 | 0 | 0 |  | O | HB |
| GLN498 | ASP355 | 4.86 | -0.39 | 0 | -1 |  | HB2 | OD2 |
| GLY496 | ASP38 | 4.88 | -0.16 | 0 | -1 |  | HA2 | OD1 |
| ALA475 | GLU23 | 4.88 | -0.44 | 0 | -1 |  | HB1 | O |
| GLU484 | GLU35 | 4.92 | 1.85 | -1 | -1 |  | OE1 | OE2 |
| THR500 | THR324 | 4.93 | 0.11 | 0 | 0 |  | O | OG1 |
| ASN501 | PHE356 | 4.97 | -0.46 | 0 | 0 |  | HA | H |
| ARG403 | ARG393 | 4.99 | 1.69 | 1 | 1 |  | HH12 | HH22 |
| TYR473 | GLU23 | 5.00 | -0.58 | 0 | -1 |  | HH | O |
| TYR421 | ASP30 | 5.05 | -0.41 | 0 | -1 |  | HE2 | OD2 |
| GLY496 | TYR41 | 5.06 | -0.21 | 0 | 0 |  | O | HE2 |
| THR500 | GLN325 | 5.07 | -0.50 | 0 | 0 |  | HA | C |
| ASP405 | ALA386 | 5.09 | -0.07 | -1 | 0 |  | OD2 | O |
| GLY504 | GLY354 | 5.09 | -0.26 | 0 | 0 |  | H | HA2 |
| TYR473 | SER19 | 5.11 | -0.23 | 0 | 1 |  | HH | HG |
| GLY485 | LYS31 | 5.12 | -0.40 | 0 | 1 |  | O | HE1 |
| CYS488 | LYS31 | 5.13 | 0.05 | 0 | 1 |  | O | HE1 |
| GLY447 | GLN42 | 5.14 | 0.00 | 0 | 0 |  | HA2 | HE22 |
| SER477 | GLN24 | 5.14 | -0.15 | 0 | 0 |  | H | OE1 |
| GLY446 | LEU45 | 5.15 | -0.14 | 0 | 0 |  | HA1 | HD11 |
| PHE456 | PHE32 | 5.21 | -0.28 | 0 | 0 |  | HZ | H |
| GLY447 | LYS353 | 5.22 | 0.02 | 0 | 1 |  | HA1 | HZ3 |
| LYS417 | LYS26 | 5.23 | 1.01 | 1 | 1 |  | HZ1 | O |
| PHE456 | HIS34 | 5.23 | -0.37 | 0 | 0 |  | HZ | HD1 |
| TYR473 | GLN24 | 5.24 | -0.28 | 0 | 0 |  | HH | HA |
| ARG403 | GLU37 | 5.25 | -1.97 | 1 | -1 |  | HH12 | OE1 |
| ARG403 | HIS34 | 5.25 | -0.87 | 1 | 0 |  | HH12 | NE2 |
| GLY502 | GLN325 | 5.26 | -0.28 | 0 | 0 |  | HA1 | H |
| TYR495 | LYS353 | 5.27 | 0.67 | 0 | 1 |  | C | HZ1 |
| GLY485 | LEU79 | 5.29 | -0.03 | 0 | 0 |  | O | HD12 |
| PRO499 | TYR41 | 5.30 | 0.00 | 0 | 0 |  | HD1 | OH |
| ASN501 | ARG357 | 5.30 | -0.19 | 0 | 1 |  | HD21 | HH11 |
| ARG457 | THR27 | 5.33 | -0.20 | 1 | 0 |  | H | HG23 |
| GLY447 | TYR41 | 5.34 | -0.11 | 0 | 0 |  | HA1 | HE2 |
| VAL503 | PHE356 | 5.34 | -0.20 | 0 | 0 |  | H | HE2 |
| THR500 | LYS353 | 5.34 | -0.86 | 0 | 1 |  | O | O |
| LEU455 | ASN33 | 5.36 | -0.06 | 0 | 0 |  | HD12 | HB1 |
| GLY446 | TYR41 | 5.36 | -0.20 | 0 | 0 |  | O | HE2 |
| GLY502 | PHE327 | 5.41 | -0.04 | 0 | 0 |  | HA1 | H |
| ALA475 | PHE28 | 5.42 | -0.15 | 0 | 0 |  | HB2 | H |
| GLN498 | ARG357 | 5.43 | 0.01 | 0 | 1 |  | HB2 | HH12 |
| SER494 | HIS34 | 5.43 | -0.25 | 0 | 0 |  | O | HB2 |
| GLY504 | THR324 | 5.46 | -0.07 | 0 | 0 |  | H | HG22 |
| TYR505 | ASP355 | 5.46 | -0.36 | 0 | -1 |  | HB1 | N |
| PRO499 | GLN325 | 5.48 | -0.68 | 0 | 0 |  | O | HB1 |
| TYR489 | GLN76 | 5.50 | -0.13 | 0 | 0 |  | HE1 | HE22 |
| GLY476 | TYR83 | 5.50 | -0.08 | 0 | 0 |  | HA1 | HE2 |
| VAL503 | GLY354 | 5.51 | 0.29 | 0 | 0 |  | N | HA2 |
| PRO499 | GLU329 | 5.54 | -0.11 | 0 | -1 |  | HB1 | OE1 |
| GLY504 | ALA387 | 5.54 | -0.08 | 0 | 0 |  | HA2 | HB2 |
| GLY476 | SER19 | 5.55 | -0.72 | 0 | 1 |  | HA1 | H3 |
| THR500 | LEU351 | 5.55 | -0.16 | 0 | 0 |  | HG1 | HD22 |
| ARG408 | ARG559 | 5.55 | 1.36 | 1 | 1 |  | HH12 | HH12 |
| TYR505 | TYR385 | 5.56 | 0.32 | 0 | 0 |  | HH | O |
| PHE486 | ALA80 | 5.57 | -0.12 | 0 | 0 |  | HE1 | N |
| LEU455 | GLU35 | 5.57 | -0.39 | 0 | -1 |  | HD23 | H |
| ASN487 | LYS31 | 5.59 | -0.12 | 0 | 1 |  | H | HG1 |
| GLY504 | PHE356 | 5.65 | -0.06 | 0 | 0 |  | H | HE2 |
| ASN487 | THR27 | 5.65 | -0.21 | 0 | 0 |  | OD1 | HG1 |
| PHE486 | THR78 | 5.66 | -0.04 | 0 | 0 |  | HE1 | O |
| ASN501 | ASN330 | 5.67 | -0.29 | 0 | 0 |  | N | HD22 |
| ASN501 | LEU45 | 5.67 | -0.08 | 0 | 0 |  | HD22 | HD12 |
| TYR505 | HIS34 | 5.68 | -0.03 | 0 | 0 |  | HE2 | HD2 |
| LEU492 | LYS31 | 5.68 | -0.34 | 0 | 1 |  | O | HZ3 |
| PHE486 | PHE28 | 5.69 | -0.29 | 0 | 0 |  | HD1 | HE1 |
| VAL503 | PRO321 | 5.73 | -0.07 | 0 | 0 |  | HG23 | HB1 |
| ARG403 | GLN388 | 5.73 | -0.49 | 1 | 0 |  | HH22 | O |
| ALA475 | ALA25 | 5.73 | -0.04 | 0 | 0 |  | HB1 | N |
| GLN493 | ASP38 | 5.73 | -0.34 | 0 | -1 |  | HE21 | HB1 |
| PRO499 | ASN330 | 5.74 | 0.37 | 0 | 0 |  | C | HD22 |
| VAL503 | ASN322 | 5.75 | -0.39 | 0 | 0 |  | HG23 | O |
| TYR489 | LEU29 | 5.75 | -0.23 | 0 | 0 |  | HE2 | N |
| LEU455 | THR27 | 5.77 | -0.31 | 0 | 0 |  | O | HG21 |
| TYR473 | LYS31 | 5.79 | -0.28 | 0 | 1 |  | HD2 | HB1 |
| GLY502 | GLY352 | 5.82 | -0.04 | 0 | 0 |  | H | O |
| LEU455 | PHE32 | 5.84 | -0.24 | 0 | 0 |  | HD23 | N |
| TYR489 | ASP30 | 5.85 | -0.39 | 0 | -1 |  | HD2 | HB1 |
| TYR505 | GLY352 | 5.85 | -0.31 | 0 | 0 |  | CE2 | C |
| PHE456 | LEU29 | 5.85 | -0.13 | 0 | 0 |  | HE1 | N |
| GLY446 | LYS353 | 5.86 | -0.10 | 0 | 1 |  | O | HZ3 |
| VAL503 | GLY326 | 5.87 | -0.07 | 0 | 0 |  | HG22 | H |
| GLY504 | ALA386 | 5.88 | -0.11 | 0 | 0 |  | HA2 | HB3 |
| GLN506 | LYS353 | 5.88 | -0.24 | 0 | 1 |  | N | O |
| ASN439 | GLY326 | 5.89 | -0.02 | 0 | 0 |  | HD22 | H |
| GLN493 | ASP30 | 5.89 | -0.11 | 0 | -1 |  | HG1 | O |
| PHE490 | GLU35 | 5.91 | -0.09 | 0 | -1 |  | HB2 | OE2 |
| TYR473 | PHE28 | 5.93 | -0.14 | 0 | 0 |  | HE2 | N |
| ARG403 | LYS353 | 5.95 | 0.93 | 1 | 1 |  | HH22 | HA |
| ASN487 | MET82 | 5.96 | -0.25 | 0 | 0 |  | HD22 | HB2 |
| TYR489 | ALA25 | 5.97 | -0.13 | 0 | 0 |  | HH | HA |
| TYR489 | PHE32 | 5.99 | -0.24 | 0 | 0 |  | HE2 | H |
| TYR505 | MET383 | 5.99 | -0.14 | 0 | 0 |  | HE1 | HA |
| GLN506 | GLY354 | 6.05 | -0.13 | 0 | 0 |  | HE21 | O |
| GLY496 | GLN42 | 6.05 | -0.08 | 0 | 0 |  | HA2 | HE21 |
| GLY504 | MET383 | 6.08 | -0.07 | 0 | 0 |  | H | HE1 |
| TYR505 | PHE390 | 6.09 | -0.13 | 0 | 0 |  | OH | HA |
| VAL503 | MET323 | 6.11 | -0.03 | 0 | 0 |  | HG23 | C |
| ASN501 | LEU351 | 6.12 | -0.07 | 0 | 0 |  | HD21 | HB1 |
| PHE497 | TYR41 | 6.13 | -0.19 | 0 | 0 |  | C | HE2 |
| PRO499 | ARG357 | 6.14 | 0.67 | 0 | 1 |  | HD1 | HH12 |
| CYS488 | LEU79 | 6.17 | -0.06 | 0 | 0 |  | H | HD12 |
| GLY496 | GLU37 | 6.18 | 0.00 | 0 | -1 |  | H | HG1 |
| PRO499 | ASP355 | 6.20 | -1.95 | 0 | -1 |  | C | OD2 |
| ASN501 | GLN325 | 6.21 | -1.04 | 0 | 0 |  | O | HB1 |
| PHE456 | GLU35 | 6.21 | -0.01 | 0 | -1 |  | HZ | HG1 |
| SER494 | LYS353 | 6.22 | -0.05 | 0 | 1 |  | O | HZ1 |
| TYR505 | PRO389 | 6.23 | -0.07 | 0 | 0 |  | OH | HA |
| PHE486 | LYS31 | 6.30 | -0.16 | 0 | 1 |  | HA | HG1 |
| ASN501 | PHE327 | 6.31 | -0.08 | 0 | 0 |  | O | H |
| THR500 | GLY352 | 6.32 | -0.07 | 0 | 0 |  | HG1 | O |
| PRO491 | LYS31 | 6.35 | -0.51 | 0 | 1 |  | N | HZ3 |
| TYR505 | ASP382 | 6.35 | -0.08 | 0 | -1 |  | HE1 | O |
| SER477 | SER19 | 6.38 | 0.00 | 0 | 1 |  | H | H3 |
| GLN498 | GLY352 | 6.39 | -0.10 | 0 | 0 |  | HB1 | O |
| GLN474 | SER19 | 6.42 | -0.25 | 0 | 1 |  | O | OG |
| LYS458 | THR27 | 6.43 | -0.07 | 1 | 0 |  | HA | HG23 |
| TYR489 | LYS26 | 6.46 | -0.04 | 0 | 1 |  | HE2 | C |
| ASN487 | ALA25 | 6.49 | -0.03 | 0 | 0 |  | OD1 | N |
| TYR495 | HIS34 | 6.49 | -0.18 | 0 | 0 |  | HA | HD2 |
| PRO499 | THR324 | 6.49 | -0.15 | 0 | 0 |  | O | HB |
| ASN439 | GLU329 | 6.50 | -0.26 | 0 | -1 |  | HD21 | OE2 |
| GLY504 | LYS353 | 6.51 | -0.32 | 0 | 1 |  | H | O |
| GLN498 | ASN330 | 6.52 | -0.08 | 0 | 0 |  | HB2 | HD22 |
| TYR505 | THR324 | 6.53 | -0.02 | 0 | 0 |  | H | HG22 |
| TYR489 | GLU35 | 6.54 | -0.27 | 0 | -1 |  | HB2 | OE2 |
| PHE486 | GLN76 | 6.55 | -0.16 | 0 | 0 |  | HA | HE22 |
| ASN448 | LYS353 | 6.56 | -0.08 | 0 | 1 |  | H | HZ3 |
| VAL445 | ARG357 | 6.56 | 0.07 | 0 | 1 |  | HG13 | HH12 |
| GLY502 | ALA386 | 6.57 | -0.01 | 0 | 0 |  | HA2 | HB1 |
| TYR473 | ASP30 | 6.58 | -0.04 | 0 | -1 |  | HE2 | HB1 |
| PHE486 | GLN81 | 6.59 | -0.03 | 0 | 0 |  | HE1 | H |
| PHE456 | GLN24 | 6.60 | -0.05 | 0 | 0 |  | HE1 | O |
| ASN437 | GLN325 | 6.62 | -0.12 | 0 | 0 |  | HD22 | OE1 |
| TYR453 | ASP30 | 6.62 | -0.14 | 0 | -1 |  | HH | O |
| ALA475 | THR20 | 6.65 | 0.08 | 0 | 0 |  | O | O |
| GLN474 | THR27 | 6.66 | -0.23 | 0 | 0 |  | O | HG23 |
| ARG403 | GLY354 | 6.66 | -0.08 | 1 | 0 |  | HH21 | HA2 |
| PRO499 | LEU45 | 6.67 | -0.03 | 0 | 0 |  | HD1 | HD12 |
| GLN493 | ALA36 | 6.67 | -0.07 | 0 | 0 |  | HE22 | H |
| GLY447 | ASP38 | 6.68 | 0.01 | 0 | -1 |  | HA2 | OD1 |
| GLN506 | PHE327 | 6.69 | -0.02 | 0 | 0 |  | HE21 | H |
| GLN493 | ASN33 | 6.69 | 0.04 | 0 | 0 |  | HG1 | C |
| LEU455 | LEU29 | 6.69 | -0.07 | 0 | 0 |  | HD23 | O |
| GLN474 | GLN24 | 6.69 | -0.05 | 0 | 0 |  | C | HG1 |
| GLY446 | ASP38 | 6.71 | 0.12 | 0 | -1 |  | O | OD1 |
| TYR473 | LYS26 | 6.71 | 0.02 | 0 | 1 |  | HE2 | C |
| ASN501 | GLU37 | 6.71 | -0.22 | 0 | -1 |  | HD22 | HG2 |
| PHE456 | ALA25 | 6.72 | -0.03 | 0 | 0 |  | HE1 | O |
| LYS458 | SER19 | 6.72 | 0.80 | 1 | 1 |  | HA | HG |
| CYS488 | TYR83 | 6.72 | -0.11 | 0 | 0 |  | H | HH |
| GLN493 | GLU37 | 6.72 | -0.16 | 0 | -1 |  | HE21 | HB2 |
| SER494 | ASP38 | 6.72 | -0.10 | 0 | -1 |  | O | HB1 |
| LYS417 | LYS31 | 6.73 | 0.58 | 1 | 1 |  | HZ1 | N |
| GLU406 | HIS34 | 6.74 | 0.40 | -1 | 0 |  | OE2 | NE2 |
| TYR505 | ASP350 | 6.77 | -0.10 | 0 | -1 |  | HE1 | OD1 |
| PHE486 | GLN24 | 6.79 | -0.19 | 0 | 0 |  | HZ | OE1 |
| TYR421 | LYS26 | 6.79 | -0.04 | 0 | 1 |  | HH | HB2 |
| PHE456 | ASN33 | 6.80 | -0.08 | 0 | 0 |  | HZ | H |
| TYR449 | LEU39 | 6.80 | -0.09 | 0 | 0 |  | HH | N |
| PHE486 | GLU75 | 6.81 | -0.24 | 0 | -1 |  | HD1 | O |
| TYR453 | ASN33 | 6.81 | 0.04 | 0 | 0 |  | HH | C |
| VAL445 | LEU45 | 6.81 | -0.08 | 0 | 0 |  | HG13 | HD11 |
| THR500 | TRP328 | 6.82 | -0.15 | 0 | 0 |  | HA | H |
| CYS488 | PHE28 | 6.82 | -0.06 | 0 | 0 |  | H | HD1 |
| TYR449 | LEU45 | 6.84 | -0.03 | 0 | 0 |  | OH | HD13 |
| SER494 | GLU35 | 6.86 | -0.05 | 0 | -1 |  | H | OE2 |
| VAL503 | LYS353 | 6.87 | -0.01 | 0 | 1 |  | N | O |
| ILE472 | LYS31 | 6.88 | 0.12 | 0 | 1 |  | HG23 | HZ1 |
| ALA475 | ILE21 | 6.88 | -0.03 | 0 | 0 |  | O | HA |
| GLY476 | THR27 | 6.89 | -0.01 | 0 | 0 |  | N | OG1 |
| ARG403 | PRO389 | 6.90 | -0.14 | 1 | 0 |  | HH12 | HA |
| GLY485 | GLU75 | 6.91 | -0.14 | 0 | -1 |  | HA1 | OE1 |
| LEU492 | GLU35 | 6.92 | 0.08 | 0 | -1 |  | O | OE2 |
| THR500 | SER331 | 6.92 | -0.07 | 0 | 0 |  | HG22 | H |
| GLU406 | ALA387 | 6.93 | -0.03 | -1 | 0 |  | HG2 | HB1 |
| TYR505 | ASN33 | 6.93 | -0.07 | 0 | 0 |  | HE2 | O |
| GLY447 | LEU45 | 6.99 | -0.04 | 0 | 0 |  | N | HD12 |
| PRO499 | PHE327 | 6.99 | -0.05 | 0 | 0 |  | O | H |
| GLN409 | ALA387 | 7.00 | -0.04 | 0 | 0 |  | HE21 | HB1 |
| VAL503 | PHE327 | 7.00 | -0.03 | 0 | 0 |  | H | H |
| THR500 | TRP48 | 7.00 | -0.06 | 0 | 0 |  | HG21 | HE1 |
| TYR449 | HIS34 | 7.02 | -0.06 | 0 | 0 |  | HE1 | O |
| ASP405 | GLY354 | 7.03 | 0.02 | -1 | 0 |  | OD2 | HA2 |
| GLY485 | GLN76 | 7.06 | -0.05 | 0 | 0 |  | O | HE22 |
| GLN506 | ASP355 | 7.07 | -0.15 | 0 | -1 |  | HE21 | HA |
| ASN501 | GLN42 | 7.07 | -0.06 | 0 | 0 |  | HD22 | HE22 |
| VAL445 | TYR41 | 7.07 | -0.13 | 0 | 0 |  | HA | HE2 |
| TYR495 | GLU37 | 7.09 | -0.12 | 0 | -1 |  | HA | HG1 |
| VAL445 | ASN330 | 7.09 | -0.07 | 0 | 0 |  | HG23 | OD1 |
| GLY502 | TYR41 | 7.10 | -0.07 | 0 | 0 |  | H | HH |
| LYS417 | ASN33 | 7.11 | 0.09 | 1 | 0 |  | HE2 | HB1 |
| ALA475 | LYS31 | 7.11 | 0.02 | 0 | 1 |  | HB2 | HB1 |
| PHE456 | GLU23 | 7.12 | -0.16 | 0 | -1 |  | HE1 | O |
| GLY496 | HIS34 | 7.12 | -0.04 | 0 | 0 |  | H | HD2 |
| TYR453 | GLU37 | 7.12 | -0.03 | 0 | -1 |  | OH | HG1 |
| SER477 | TYR83 | 7.13 | -0.01 | 0 | 0 |  | H | HE2 |
| ARG408 | ALA386 | 7.15 | 0.07 | 1 | 0 |  | HH22 | HB3 |
| GLY485 | PHE28 | 7.15 | -0.03 | 0 | 0 |  | O | HD1 |
| LYS417 | LEU29 | 7.15 | 0.39 | 1 | 0 |  | HZ1 | C |
| ASN501 | ASP38 | 7.16 | -0.07 | 0 | -1 |  | HD22 | HA |
| LEU455 | PHE28 | 7.16 | -0.07 | 0 | 0 |  | HD23 | O |
| ALA475 | LYS26 | 7.16 | -0.15 | 0 | 1 |  | HB2 | C |
| LYS444 | TYR41 | 7.18 | -0.07 | 1 | 0 |  | O | HE2 |
| ASP405 | GLN388 | 7.19 | 0.12 | -1 | 0 |  | OD2 | N |
| GLY446 | ARG357 | 7.20 | 0.08 | 0 | 1 |  | H | HH12 |
| ASN487 | GLN76 | 7.21 | -0.02 | 0 | 0 |  | H | HE22 |
| ASN487 | ILE21 | 7.24 | -0.01 | 0 | 0 |  | HD22 | HA |
| TYR489 | GLU23 | 7.25 | -0.05 | 0 | -1 |  | HE2 | O |
| GLY502 | MET323 | 7.28 | 0.21 | 0 | 0 |  | HA1 | C |
| ASN440 | GLN325 | 7.28 | -0.04 | 0 | 0 |  | HB2 | OE1 |
| GLN506 | GLU329 | 7.28 | -0.17 | 0 | -1 |  | HE22 | OE2 |
| GLN493 | LEU39 | 7.30 | -0.02 | 0 | 0 |  | HE22 | HD13 |
| ARG403 | ASN33 | 7.30 | -0.17 | 1 | 0 |  | HH12 | HB2 |
| GLN498 | GLY326 | 7.33 | -0.06 | 0 | 0 |  | O | HA1 |
| GLY476 | ILE21 | 7.34 | -0.01 | 0 | 0 |  | HA1 | HA |
| ARG408 | GLN388 | 7.34 | -0.19 | 1 | 0 |  | HH12 | N |
| TYR449 | GLU37 | 7.35 | 0.03 | 0 | -1 |  | HH | C |
| GLN498 | GLU37 | 7.35 | 0.12 | 0 | -1 |  | OE1 | HG2 |
| GLN493 | PHE72 | 7.35 | -0.02 | 0 | 0 |  | HE22 | HZ |
| TYR489 | ALA80 | 7.37 | -0.04 | 0 | 0 |  | HH | HA |
| LEU455 | LYS26 | 7.39 | -0.07 | 0 | 1 |  | HD12 | O |
| SER494 | GLU37 | 7.40 | -0.01 | 0 | -1 |  | O | HB2 |
| ARG457 | ASP30 | 7.40 | -0.67 | 1 | -1 |  | H | OD2 |
| GLN493 | PHE32 | 7.40 | -0.08 | 0 | 0 |  | HE22 | O |
| ASN448 | TYR41 | 7.41 | -0.02 | 0 | 0 |  | H | HE2 |
| VAL445 | GLU329 | 7.44 | -0.12 | 0 | -1 |  | HG23 | OE1 |
| PRO499 | GLY354 | 7.46 | 0.04 | 0 | 0 |  | O | O |
| GLN506 | PHE356 | 7.48 | -0.06 | 0 | 0 |  | HE21 | HD2 |
| LEU455 | ALA36 | 7.50 | -0.02 | 0 | 0 |  | HD23 | H |
| ASN501 | MET383 | 7.50 | -0.04 | 0 | 0 |  | C | HE1 |
| ARG403 | TYR385 | 7.51 | -0.06 | 1 | 0 |  | HH22 | O |
| ASN439 | THR324 | 7.53 | 0.00 | 0 | 0 |  | HD22 | HB |
| LYS417 | PHE28 | 7.54 | -0.16 | 1 | 0 |  | HZ1 | N |
| TYR449 | GLU35 | 7.60 | -0.04 | 0 | -1 |  | HE1 | HA |
| GLY496 | LEU45 | 7.60 | -0.02 | 0 | 0 |  | O | HD12 |
| TYR421 | GLU23 | 7.61 | -0.09 | 0 | -1 |  | HH | OE1 |
| GLN498 | GLY354 | 7.62 | -0.04 | 0 | 0 |  | O | O |
| TYR495 | ASP38 | 7.62 | -0.09 | 0 | -1 |  | HA | HB1 |
| PRO499 | LYS353 | 7.62 | -0.05 | 0 | 1 |  | N | HD1 |
| VAL503 | ASP355 | 7.65 | 0.11 | 0 | -1 |  | H | HA |
| SER494 | LYS31 | 7.66 | -0.11 | 0 | 1 |  | H | HZ3 |
| GLY404 | ALA387 | 7.66 | -0.02 | 0 | 0 |  | C | HB2 |
| ALA475 | LEU79 | 7.67 | -0.01 | 0 | 0 |  | HB3 | HD23 |
| THR478 | GLN24 | 7.68 | -0.03 | 0 | 0 |  | H | OE1 |
| TYR453 | GLU35 | 7.69 | 0.06 | 0 | -1 |  | OH | N |
| ASP405 | LYS353 | 7.71 | -0.75 | -1 | 1 |  | OD2 | HA |
| PHE497 | ASP38 | 7.74 | 0.02 | 0 | -1 |  | N | OD1 |
| ALA475 | ASP30 | 7.76 | 0.00 | 0 | -1 |  | HB2 | HB1 |
| LEU492 | HIS34 | 7.78 | -0.09 | 0 | 0 |  | O | HB2 |
| TYR473 | TYR83 | 7.79 | -0.01 | 0 | 0 |  | HE2 | HH |
| TYR489 | HIS34 | 7.81 | -0.02 | 0 | 0 |  | HB2 | HB1 |
| GLY502 | LEU351 | 7.82 | 0.00 | 0 | 0 |  | H | H |
| PRO507 | LYS353 | 7.82 | -0.04 | 0 | 1 |  | HD2 | HB2 |
| GLN498 | LEU351 | 7.83 | -0.04 | 0 | 0 |  | HB1 | HB1 |
| TYR473 | ALA25 | 7.89 | -0.03 | 0 | 0 |  | HE2 | N |
| VAL503 | ALA386 | 7.89 | -0.02 | 0 | 0 |  | N | HB1 |
| ARG403 | PHE390 | 7.90 | -0.05 | 1 | 0 |  | HH12 | H |
| GLN493 | LYS353 | 7.90 | 0.25 | 0 | 1 |  | HE21 | HE1 |
| ASP405 | MET383 | 7.90 | 0.19 | -1 | 0 |  | OD1 | O |
| GLY496 | ASP355 | 7.90 | 0.08 | 0 | -1 |  | O | HB1 |
| GLY496 | GLY352 | 7.91 | 0.00 | 0 | 0 |  | O | O |
| TYR489 | MET82 | 7.91 | -0.03 | 0 | 0 |  | HH | HE2 |
| CYS488 | THR27 | 7.92 | -0.07 | 0 | 0 |  | N | HB |
| PHE490 | HIS34 | 7.92 | -0.09 | 0 | 0 |  | O | HB1 |
| ASN487 | GLU23 | 7.95 | 0.04 | 0 | -1 |  | OD1 | O |
| GLY502 | TRP328 | 7.95 | -0.01 | 0 | 0 |  | HA1 | H |
| GLY502 | ARG357 | 7.95 | -0.09 | 0 | 1 |  | H | HH11 |
| ASN448 | GLN42 | 7.96 | 0.00 | 0 | 0 |  | H | HE22 |
| PRO491 | THR27 | 7.96 | 0.00 | 0 | 0 |  | HA | HB |
| GLY504 | ASP355 | 7.97 | -0.09 | 0 | -1 |  | H | N |
| PHE497 | GLN42 | 7.98 | -0.02 | 0 | 0 |  | C | HE22 |
| TYR453 | ARG393 | 8.00 | -0.02 | 0 | 1 |  | HH | HH22 |

**Supplementary** **Table 7**. Interactions between SARS-CoV-2 RBD e *Aotus nancymaae* ACE2.

| **Interaction** | **SARS-CoV-2  RBD** | ***Aotus nancymaae*  ACE2** |
| --- | --- | --- |
|  | Residue | Residue |
| Hydrophobic | TYR489 | PHE28 |
|  | PHE486 | LEU79 |
|  | PHE486 | TYR83 |
| Hydrogen bonds | GLY502 | LYS353 |
|  | ALA475 | SER19 |
|  | GLY496 | LYS353 |
|  | THR500 | ASP355 |
|  | GLN493 | LYS31 |
|  | GLN493 | GLU35 |
|  | ANS487 | TYR83 |
|  | GLN498 | LYS353 |
|  | LYS417 | ASP30 |
|  | TYR449 | ASP38 |
|  | ASN487 | TYR83 |
|  | GLN493 | GLU35 |
|  | TYR505 | GLU37 |
| Ionic | LYS417 | ASP30 |
|  | GLU484 | LYS31 |
| Aromatic-Aromatic | PHE486 | TYR83 |

**Supplementary** **table 8**. Quantum biochemistry between SARS-CoV-2 RBD e *Aotus nancymaae* ACE2.

| **Amino acid residues** | | **Distance** | **Interaction energy** | **Residue charge** | |  | **Residue atom** | |
| --- | --- | --- | --- | --- | --- | --- | --- | --- |
| SARS-CoV-2  RBD | *Aotus nancymaae* ACE2 | (Å) | (kcal mol-1) | SARS-CoV-2  RBD | *Aotus nancymaae* ACE2 |  | SARS-CoV-2  RBD | *Aotus nancymaae* ACE2 |
| TYR505 | GLU37 | 1.61 | -5.70 | 0 | -1 |  | HH | OE1 |
| LYS417 | ASP30 | 1.77 | -11.90 | 1 | -1 |  | HZ1 | OD2 |
| GLY502 | LYS353 | 1.89 | -4.06 | 0 | 1 |  | H | O |
| TYR449 | ASP38 | 1.93 | -9.59 | 0 | -1 |  | HH | OD2 |
| GLY496 | LYS353 | 2.03 | -5.30 | 0 | 1 |  | O | HZ2 |
| ASN487 | TYR83 | 2.06 | -5.38 | 0 | 0 |  | OD1 | HH |
| PHE456 | LYS31 | 2.20 | -3.05 | 0 | 1 |  | HZ | HB1 |
| PHE456 | ASP30 | 2.23 | -2.19 | 0 | -1 |  | HE1 | HB1 |
| TYR453 | HIS34 | 2.24 | -0.48 | 0 | 0 |  | HH | HB2 |
| ASN501 | LYS353 | 2.24 | -12.40 | 0 | 1 |  | HD22 | HD1 |
| PHE486 | LEU79 | 2.25 | -2.56 | 0 | 0 |  | HD1 | HD23 |
| LEU455 | LYS31 | 2.25 | -1.93 | 0 | 1 |  | HD21 | HA |
| TYR505 | GLN354 | 2.29 | -3.53 | 0 | 0 |  | HD1 | HG2 |
| ASN501 | ASP355 | 2.29 | -1.96 | 0 | -1 |  | HA | HB1 |
| GLN493 | GLU35 | 2.30 | -3.21 | 0 | -1 |  | HE22 | OE2 |
| TYR505 | LYS353 | 2.32 | -7.80 | 0 | 1 |  | HB2 | HB2 |
| GLN498 | LYS353 | 2.35 | -2.07 | 0 | 1 |  | HE22 | HZ1 |
| PHE456 | THR27 | 2.35 | -1.99 | 0 | 0 |  | HE1 | HA |
| TYR505 | ARG393 | 2.38 | 1.36 | 0 | 1 |  | HH | HH22 |
| GLY502 | GLN354 | 2.38 | -5.13 | 0 | 0 |  | HA2 | HB1 |
| GLN493 | LYS31 | 2.47 | -1.29 | 0 | 1 |  | HE22 | HZ3 |
| GLY504 | GLN354 | 2.48 | -0.23 | 0 | 0 |  | HA2 | HG2 |
| TYR489 | PHE28 | 2.50 | -1.92 | 0 | 0 |  | HH | HB1 |
| LEU455 | HIS34 | 2.53 | -2.68 | 0 | 0 |  | HD12 | HD1 |
| ALA475 | SER19 | 2.56 | -0.76 | 0 | 1 |  | O | HB1 |
| TYR473 | THR27 | 2.57 | -2.13 | 0 | 0 |  | HE2 | HG22 |
| LEU455 | ASP30 | 2.62 | -2.05 | 0 | -1 |  | HD12 | HB2 |
| GLN498 | HIS41 | 2.64 | -1.42 | 0 | 0 |  | OE1 | HD2 |
| ALA475 | GLN24 | 2.65 | -1.54 | 0 | 0 |  | O | HG1 |
| PHE486 | THR82 | 2.67 | -1.59 | 0 | 0 |  | HZ | OG1 |
| THR500 | ARG357 | 2.69 | -0.86 | 0 | 1 |  | HB | HH11 |
| THR500 | ASP355 | 2.81 | -1.53 | 0 | -1 |  | O | HB1 |
| THR500 | HIS41 | 2.82 | -1.23 | 0 | 0 |  | OG1 | HE2 |
| GLN493 | HIS34 | 2.82 | -2.22 | 0 | 0 |  | HG1 | HB2 |
| THR500 | ASN330 | 2.82 | -2.05 | 0 | 0 |  | HA | HD22 |
| TYR489 | LYS31 | 2.98 | -4.43 | 0 | 1 |  | HD1 | HE1 |
| ASN501 | HIS41 | 2.99 | -1.30 | 0 | 0 |  | HD21 | HE2 |
| TYR489 | THR27 | 3.05 | -0.78 | 0 | 0 |  | HE2 | HB |
| LYS417 | HIS34 | 3.12 | -0.94 | 1 | 0 |  | HE2 | HE1 |
| TYR489 | TYR83 | 3.21 | -0.60 | 0 | 0 |  | HH | OH |
| ALA475 | THR27 | 3.22 | -0.67 | 0 | 0 |  | HB2 | HB |
| GLN498 | LEU45 | 3.26 | -0.59 | 0 | 0 |  | HG1 | HD13 |
| THR500 | LEU45 | 3.39 | -0.97 | 0 | 0 |  | OG1 | HD23 |
| GLY476 | GLN24 | 3.52 | -0.50 | 0 | 0 |  | HA1 | NE2 |
| GLN498 | ASP38 | 3.52 | -2.18 | 0 | -1 |  | HE22 | OD1 |
| GLY496 | ASP38 | 3.55 | -0.15 | 0 | -1 |  | HA2 | OD1 |
| ASN487 | GLN24 | 3.58 | -0.73 | 0 | 0 |  | OD1 | HB2 |
| PHE486 | TYR83 | 3.74 | -2.09 | 0 | 0 |  | HE1 | OH |
| GLU484 | LYS31 | 3.74 | -4.75 | -1 | 1 |  | OE1 | HZ2 |
| GLY502 | ASP355 | 3.76 | -1.54 | 0 | -1 |  | H | HB1 |
| ASP405 | GLN354 | 3.78 | -0.78 | -1 | 0 |  | OD2 | HE21 |
| GLY476 | SER19 | 3.79 | 0.54 | 0 | 1 |  | HA1 | HB1 |
| TYR449 | LYS353 | 3.82 | 0.41 | 0 | 1 |  | HH | HZ3 |
| GLN498 | GLU42 | 3.84 | -0.91 | 0 | -1 |  | HE22 | HG2 |
| VAL503 | GLN354 | 3.89 | -0.07 | 0 | 0 |  | N | HB1 |
| TYR449 | GLU42 | 3.99 | -0.77 | 0 | -1 |  | HH | OE2 |
| PHE490 | LYS31 | 4.01 | -2.51 | 0 | 1 |  | O | HZ1 |
| ARG403 | HIS34 | 4.08 | -0.70 | 1 | 0 |  | HH11 | HD2 |
| GLY502 | THR324 | 4.10 | -0.02 | 0 | 0 |  | HA2 | HG21 |
| PHE497 | LYS353 | 4.26 | -1.53 | 0 | 1 |  | N | HZ2 |
| GLY502 | GLY326 | 4.29 | -0.03 | 0 | 0 |  | HA1 | HA2 |
| VAL503 | THR324 | 4.30 | -0.52 | 0 | 0 |  | HG21 | HB |
| SER477 | GLN24 | 4.33 | 0.01 | 0 | 0 |  | H | HE22 |
| PHE456 | LYS26 | 4.38 | -0.14 | 0 | 1 |  | HE1 | O |
| TYR489 | LEU79 | 4.41 | -0.27 | 0 | 0 |  | HE1 | HD12 |
| GLY502 | PHE356 | 4.42 | -0.37 | 0 | 0 |  | HA2 | HE2 |
| GLN474 | SER19 | 4.45 | -0.25 | 0 | 1 |  | O | OG |
| PHE456 | PHE28 | 4.46 | -0.14 | 0 | 0 |  | HZ | HA |
| ASN501 | GLN354 | 4.46 | -3.77 | 0 | 0 |  | C | HB1 |
| LYS417 | THR27 | 4.47 | -0.36 | 1 | 0 |  | HZ1 | HA |
| TYR489 | GLN24 | 4.53 | -0.62 | 0 | 0 |  | HH | O |
| ASN487 | LEU79 | 4.57 | -0.30 | 0 | 0 |  | H | HD23 |
| SER477 | SER19 | 4.58 | 0.14 | 0 | 1 |  | H | H1 |
| ASN501 | LEU45 | 4.60 | -0.17 | 0 | 0 |  | HD22 | HD13 |
| ARG403 | GLN354 | 4.63 | -0.11 | 1 | 0 |  | HH21 | HG2 |
| ASN487 | PHE28 | 4.64 | -0.51 | 0 | 0 |  | OD1 | HB1 |
| VAL503 | GLN325 | 4.69 | -0.27 | 0 | 0 |  | HG23 | HB1 |
| ASN501 | GLY352 | 4.72 | -0.31 | 0 | 0 |  | HA | O |
| GLN506 | GLY326 | 4.73 | -0.02 | 0 | 0 |  | HE22 | HA2 |
| ASN501 | GLY326 | 4.74 | -0.27 | 0 | 0 |  | O | HA2 |
| TYR505 | ALA386 | 4.74 | -0.39 | 0 | 0 |  | HE1 | O |
| PRO499 | ASN330 | 4.76 | -0.13 | 0 | 0 |  | O | HD22 |
| ALA475 | TYR83 | 4.77 | -0.17 | 0 | 0 |  | HB1 | HH |
| LEU455 | GLU35 | 4.78 | -0.42 | 0 | -1 |  | HD21 | HG1 |
| TYR421 | ASP30 | 4.81 | -0.16 | 0 | -1 |  | HE2 | OD2 |
| GLN506 | GLN354 | 4.83 | -0.40 | 0 | 0 |  | H | HB1 |
| ARG403 | ALA387 | 4.87 | -0.13 | 1 | 0 |  | HH22 | HA |
| PHE456 | PHE32 | 4.92 | -0.21 | 0 | 0 |  | HZ | H |
| GLN506 | GLN325 | 4.95 | 0.11 | 0 | 0 |  | HE22 | HB1 |
| TYR505 | HIS34 | 4.96 | -0.31 | 0 | 0 |  | OH | HD2 |
| VAL503 | GLY326 | 4.98 | -0.02 | 0 | 0 |  | HG23 | H |
| ASP405 | ALA387 | 4.98 | -0.62 | -1 | 0 |  | OD1 | HB3 |
| THR500 | GLY326 | 4.99 | -0.37 | 0 | 0 |  | O | HA2 |
| TYR489 | GLN76 | 5.03 | -0.27 | 0 | 0 |  | HE1 | HE22 |
| GLY504 | LYS353 | 5.04 | -0.45 | 0 | 1 |  | H | O |
| ARG408 | ALA387 | 5.08 | -0.13 | 1 | 0 |  | HH12 | HB3 |
| LYS417 | LYS26 | 5.09 | 1.01 | 1 | 1 |  | HZ1 | O |
| GLN506 | LYS353 | 5.14 | -0.51 | 0 | 1 |  | H | O |
| GLY502 | GLY352 | 5.14 | -0.03 | 0 | 0 |  | H | O |
| ALA475 | GLU23 | 5.20 | -0.19 | 0 | -1 |  | HB1 | O |
| PHE456 | HIS34 | 5.21 | -0.41 | 0 | 0 |  | HZ | HD1 |
| TYR505 | ALA387 | 5.23 | -0.16 | 0 | 0 |  | HE1 | HA |
| SER494 | HIS34 | 5.25 | -0.28 | 0 | 0 |  | O | HB2 |
| GLY446 | GLU42 | 5.28 | 0.72 | 0 | -1 |  | O | OE2 |
| GLY447 | LYS353 | 5.29 | 0.08 | 0 | 1 |  | HA2 | HZ1 |
| TYR495 | LYS353 | 5.29 | 1.10 | 0 | 1 |  | C | HZ2 |
| TYR505 | PHE390 | 5.29 | -0.12 | 0 | 0 |  | HH | HD1 |
| THR500 | LEU351 | 5.29 | -0.18 | 0 | 0 |  | HB | HD21 |
| TYR473 | GLN24 | 5.30 | -0.11 | 0 | 0 |  | HE2 | HA |
| TYR505 | ASN33 | 5.31 | -0.42 | 0 | 0 |  | HH | O |
| TYR421 | THR27 | 5.34 | -0.15 | 0 | 0 |  | HE2 | HG23 |
| ASN501 | ARG357 | 5.34 | -0.23 | 0 | 1 |  | HA | HH11 |
| ARG403 | GLU37 | 5.36 | -1.91 | 1 | -1 |  | HD1 | HG1 |
| LEU455 | THR27 | 5.37 | -0.38 | 0 | 0 |  | HD21 | O |
| ASN501 | ASN330 | 5.39 | -0.62 | 0 | 0 |  | N | HD22 |
| VAL503 | PHE356 | 5.40 | -0.16 | 0 | 0 |  | H | HE2 |
| LYS458 | SER19 | 5.42 | 0.97 | 1 | 1 |  | HG1 | HG |
| THR500 | LYS353 | 5.43 | -0.56 | 0 | 1 |  | O | O |
| ARG403 | ARG393 | 5.45 | 1.73 | 1 | 1 |  | HH12 | HH21 |
| ASN487 | THR27 | 5.46 | -0.40 | 0 | 0 |  | OD1 | OG1 |
| LEU455 | PHE32 | 5.46 | -0.25 | 0 | 0 |  | HD21 | N |
| LEU455 | ASN33 | 5.47 | -0.19 | 0 | 0 |  | HD12 | HB1 |
| TYR473 | SER19 | 5.49 | -0.31 | 0 | 1 |  | OH | HG |
| VAL503 | LYS353 | 5.50 | 0.13 | 0 | 1 |  | N | O |
| TYR505 | ASP355 | 5.50 | -0.39 | 0 | -1 |  | HB1 | H |
| ASN487 | THR82 | 5.52 | -0.24 | 0 | 0 |  | HD22 | OG1 |
| ALA475 | PHE28 | 5.52 | -0.15 | 0 | 0 |  | HB1 | H |
| GLY446 | LEU45 | 5.55 | -0.08 | 0 | 0 |  | HA1 | HD12 |
| PHE486 | PHE28 | 5.55 | -0.16 | 0 | 0 |  | HE1 | HD1 |
| ARG403 | LYS353 | 5.56 | 1.06 | 1 | 1 |  | HD1 | HA |
| PHE456 | LEU29 | 5.57 | -0.27 | 0 | 0 |  | HZ | C |
| LEU492 | LYS31 | 5.59 | -0.13 | 0 | 1 |  | O | HZ1 |
| ARG457 | THR27 | 5.59 | -0.06 | 1 | 0 |  | H | HG23 |
| THR500 | GLN354 | 5.60 | -0.55 | 0 | 0 |  | O | O |
| TYR473 | GLU23 | 5.62 | -0.01 | 0 | -1 |  | HE2 | O |
| GLY447 | ASP38 | 5.65 | -0.07 | 0 | -1 |  | HA2 | OD1 |
| TYR505 | GLY352 | 5.69 | -0.65 | 0 | 0 |  | HH | HA1 |
| GLY504 | PHE356 | 5.71 | -0.07 | 0 | 0 |  | H | HE2 |
| GLN506 | THR324 | 5.71 | -0.05 | 0 | 0 |  | HE22 | HB |
| GLY476 | TYR83 | 5.71 | -0.07 | 0 | 0 |  | HA2 | HE2 |
| THR500 | PHE356 | 5.71 | 0.10 | 0 | 0 |  | O | H |
| ARG403 | ALA386 | 5.78 | -0.43 | 1 | 0 |  | HH22 | O |
| GLY496 | HIS41 | 5.78 | -0.09 | 0 | 0 |  | O | HD2 |
| PHE486 | ALA80 | 5.81 | -0.06 | 0 | 0 |  | HE1 | N |
| PHE486 | LYS78 | 5.83 | -0.06 | 0 | 1 |  | HE1 | O |
| THR500 | GLU329 | 5.86 | -0.12 | 0 | -1 |  | HA | OE1 |
| TYR505 | ASP38 | 5.87 | -0.13 | 0 | -1 |  | HE2 | H |
| TYR453 | GLU35 | 5.87 | -0.19 | 0 | -1 |  | HH | N |
| GLY504 | THR324 | 5.89 | -0.03 | 0 | 0 |  | H | HG21 |
| TYR489 | LEU29 | 5.89 | -0.23 | 0 | 0 |  | HH | N |
| GLN493 | ASP30 | 5.89 | -0.01 | 0 | -1 |  | HG1 | O |
| ARG408 | GLN354 | 5.93 | 0.01 | 1 | 0 |  | HH12 | HE22 |
| PRO491 | LYS31 | 5.95 | -0.59 | 0 | 1 |  | HA | HZ1 |
| GLN506 | ASN330 | 5.96 | 0.04 | 0 | 0 |  | HE21 | HD22 |
| PRO499 | GLY326 | 6.00 | -0.09 | 0 | 0 |  | O | HA1 |
| GLY485 | LEU79 | 6.03 | -0.06 | 0 | 0 |  | C | HD22 |
| ASP405 | ALA386 | 6.05 | -0.09 | -1 | 0 |  | OD2 | HB2 |
| GLY502 | PHE327 | 6.07 | -0.01 | 0 | 0 |  | HA1 | H |
| PHE486 | GLN24 | 6.07 | -0.26 | 0 | 0 |  | HZ | OE1 |
| ALA475 | THR20 | 6.08 | 0.02 | 0 | 0 |  | O | O |
| CYS488 | TYR83 | 6.09 | -0.09 | 0 | 0 |  | H | HH |
| PRO499 | GLU329 | 6.09 | 0.29 | 0 | -1 |  | O | OE1 |
| CYS488 | LYS31 | 6.10 | 0.00 | 0 | 1 |  | O | HE1 |
| LYS417 | ASN33 | 6.10 | 0.24 | 1 | 0 |  | HE2 | HD21 |
| TYR473 | PHE28 | 6.10 | -0.10 | 0 | 0 |  | HE2 | N |
| ALA475 | ALA25 | 6.15 | -0.08 | 0 | 0 |  | HB1 | N |
| GLU406 | HIS34 | 6.15 | 0.48 | -1 | 0 |  | OE2 | HD2 |
| GLN498 | ASP355 | 6.16 | -0.11 | 0 | -1 |  | HB1 | HB1 |
| TYR495 | HIS34 | 6.16 | -0.23 | 0 | 0 |  | HE1 | HD2 |
| SER494 | ASP38 | 6.17 | -0.22 | 0 | -1 |  | HB2 | OD2 |
| ASN501 | PHE356 | 6.19 | -0.19 | 0 | 0 |  | HA | H |
| TYR453 | ASP30 | 6.21 | -0.06 | 0 | -1 |  | HE2 | HB2 |
| LEU455 | LEU29 | 6.23 | -0.09 | 0 | 0 |  | HD12 | O |
| TYR449 | LEU39 | 6.24 | -0.21 | 0 | 0 |  | HH | N |
| PHE456 | GLU35 | 6.24 | -0.03 | 0 | -1 |  | HE2 | HG1 |
| ASN501 | THR324 | 6.25 | -0.07 | 0 | 0 |  | O | HG1 |
| TYR453 | GLU37 | 6.26 | -0.05 | 0 | -1 |  | HH | HB2 |
| GLN474 | GLN24 | 6.29 | -0.05 | 0 | 0 |  | O | HG1 |
| THR500 | GLY352 | 6.30 | -0.04 | 0 | 0 |  | O | O |
| TYR473 | LYS31 | 6.30 | -0.25 | 0 | 1 |  | HD2 | HB1 |
| ASN501 | LEU351 | 6.30 | -0.09 | 0 | 0 |  | HA | HB1 |
| PRO499 | LEU45 | 6.32 | -0.18 | 0 | 0 |  | HD1 | HD13 |
| GLY476 | ILE21 | 6.32 | -0.03 | 0 | 0 |  | HA1 | HA |
| GLN474 | THR27 | 6.33 | -0.16 | 0 | 0 |  | O | HG22 |
| TYR489 | ASP30 | 6.34 | -0.18 | 0 | -1 |  | HE2 | HB1 |
| ALA475 | ILE21 | 6.34 | -0.04 | 0 | 0 |  | O | HA |
| ASN501 | ASP38 | 6.36 | -0.14 | 0 | -1 |  | HD22 | HA |
| TYR489 | ALA25 | 6.37 | -0.18 | 0 | 0 |  | HH | HA |
| TYR453 | ASN33 | 6.38 | -0.25 | 0 | 0 |  | HH | C |
| SER494 | GLU35 | 6.38 | -0.11 | 0 | -1 |  | H | OE2 |
| GLN498 | GLU37 | 6.39 | 0.09 | 0 | -1 |  | HE22 | O |
| ASN487 | ILE21 | 6.39 | -0.03 | 0 | 0 |  | HD22 | HG22 |
| GLY446 | ASP38 | 6.40 | 0.03 | 0 | -1 |  | O | OD1 |
| LYS417 | LYS31 | 6.40 | 0.62 | 1 | 1 |  | HE2 | N |
| GLY502 | GLN325 | 6.41 | 0.13 | 0 | 0 |  | HA1 | HB1 |
| VAL445 | LEU45 | 6.42 | -0.09 | 0 | 0 |  | HG11 | HD12 |
| PHE497 | ASP38 | 6.43 | -0.02 | 0 | -1 |  | N | OD1 |
| GLY504 | ALA386 | 6.44 | -0.03 | 0 | 0 |  | HA2 | HB2 |
| ASN448 | LYS353 | 6.45 | -0.17 | 0 | 1 |  | H | HZ2 |
| TYR505 | PRO389 | 6.48 | -0.05 | 0 | 0 |  | HH | HA |
| ASN487 | ALA25 | 6.49 | 0.01 | 0 | 0 |  | OD1 | N |
| TYR489 | GLU35 | 6.51 | -0.20 | 0 | -1 |  | HB2 | OE2 |
| GLY446 | LYS353 | 6.52 | 0.01 | 0 | 1 |  | O | HZ1 |
| PHE486 | GLN81 | 6.53 | -0.11 | 0 | 0 |  | HE1 | H |
| TYR449 | LYS68 | 6.54 | -0.07 | 0 | 1 |  | HH | HZ1 |
| TYR449 | HIS41 | 6.55 | -0.10 | 0 | 0 |  | HH | HB2 |
| GLY502 | ASN330 | 6.55 | -0.11 | 0 | 0 |  | HA1 | HD22 |
| GLY502 | HIS41 | 6.55 | -0.12 | 0 | 0 |  | H | HE1 |
| TYR489 | PHE32 | 6.55 | -0.19 | 0 | 0 |  | OH | H |
| GLY485 | LYS31 | 6.55 | -0.19 | 0 | 1 |  | O | HE1 |
| VAL503 | ASP355 | 6.56 | 0.05 | 0 | -1 |  | H | HA |
| GLN506 | GLU329 | 6.57 | -0.39 | 0 | -1 |  | HE22 | OE2 |
| GLY404 | GLN354 | 6.57 | -0.09 | 0 | 0 |  | H | HG2 |
| GLN493 | ASP38 | 6.59 | -0.40 | 0 | -1 |  | HG2 | HB1 |
| TYR495 | ASP38 | 6.59 | -0.27 | 0 | -1 |  | C | OD1 |
| GLY447 | GLU42 | 6.60 | 0.37 | 0 | -1 |  | HA2 | OE2 |
| PHE486 | PRO84 | 6.61 | -0.06 | 0 | 0 |  | HZ | HD2 |
| TYR505 | PHE356 | 6.62 | -0.12 | 0 | 0 |  | HD1 | HZ |
| GLU484 | GLU35 | 6.62 | 1.80 | -1 | -1 |  | OE1 | OE2 |
| GLY447 | HIS41 | 6.62 | -0.04 | 0 | 0 |  | HA1 | HD2 |
| TYR473 | ASP30 | 6.62 | -0.04 | 0 | -1 |  | HE2 | HB1 |
| GLN506 | ASP355 | 6.64 | -0.18 | 0 | -1 |  | HE21 | HB1 |
| THR500 | TRP48 | 6.64 | -0.09 | 0 | 0 |  | HG22 | HD1 |
| PRO499 | HIS41 | 6.65 | -0.04 | 0 | 0 |  | HD1 | HE2 |
| GLY476 | THR27 | 6.66 | -0.06 | 0 | 0 |  | N | OG1 |
| VAL445 | ASN49 | 6.68 | -0.02 | 0 | 0 |  | HG11 | HD22 |
| SER494 | LYS31 | 6.68 | -0.12 | 0 | 1 |  | H | HZ3 |
| GLN498 | ARG357 | 6.69 | 0.01 | 0 | 1 |  | HB2 | HH12 |
| THR500 | THR324 | 6.69 | -0.14 | 0 | 0 |  | O | HG1 |
| ASN439 | GLU329 | 6.70 | -0.28 | 0 | -1 |  | HD21 | OE1 |
| ASN439 | ASN330 | 6.71 | 0.01 | 0 | 0 |  | HD22 | HD22 |
| ASN487 | LYS31 | 6.73 | -0.09 | 0 | 1 |  | HA | HG1 |
| LYS458 | THR27 | 6.73 | -0.06 | 1 | 0 |  | HA | HG22 |
| TYR505 | HIS41 | 6.75 | -0.15 | 0 | 0 |  | HD2 | HB1 |
| PRO499 | ASP355 | 6.77 | -0.43 | 0 | -1 |  | O | HB1 |
| ASN487 | SER19 | 6.77 | -0.11 | 0 | 1 |  | HB2 | HB1 |
| ASP405 | LYS353 | 6.78 | -0.97 | -1 | 1 |  | OD2 | HA |
| TYR489 | LYS26 | 6.78 | 0.03 | 0 | 1 |  | HE2 | C |
| ARG403 | ASN33 | 6.79 | -0.02 | 1 | 0 |  | HH12 | HB2 |
| GLY496 | GLU37 | 6.79 | 0.00 | 0 | -1 |  | H | HG1 |
| ASN501 | GLU37 | 6.80 | -0.29 | 0 | -1 |  | HD22 | HG2 |
| GLN498 | LEU39 | 6.81 | -0.17 | 0 | 0 |  | HE22 | N |
| LEU455 | PHE28 | 6.82 | -0.11 | 0 | 0 |  | HD21 | O |
| TYR473 | LYS26 | 6.83 | -0.11 | 0 | 1 |  | HE2 | C |
| PHE456 | ASN33 | 6.83 | -0.09 | 0 | 0 |  | HZ | H |
| ALA475 | LYS31 | 6.84 | -0.01 | 0 | 1 |  | HB2 | HB1 |
| GLY447 | LEU45 | 6.86 | -0.04 | 0 | 0 |  | HA1 | HD13 |
| THR500 | PHE327 | 6.86 | -0.07 | 0 | 0 |  | O | N |
| TYR505 | GLN388 | 6.87 | -0.02 | 0 | 0 |  | OH | O |
| GLY504 | ASP355 | 6.89 | -0.17 | 0 | -1 |  | H | N |
| CYS488 | LEU79 | 6.89 | -0.04 | 0 | 0 |  | H | HD23 |
| PHE456 | GLU23 | 6.91 | -0.22 | 0 | -1 |  | HE1 | O |
| GLY476 | THR20 | 6.91 | -0.04 | 0 | 0 |  | HA1 | N |
| GLN493 | ASN33 | 6.93 | 0.04 | 0 | 0 |  | HG1 | C |
| SER494 | LYS353 | 6.94 | 0.02 | 0 | 1 |  | O | HZ2 |
| GLY496 | HIS34 | 6.94 | -0.04 | 0 | 0 |  | H | O |
| PHE490 | GLU35 | 6.95 | 0.02 | 0 | -1 |  | HB2 | OE2 |
| ASN437 | GLN325 | 6.96 | -0.13 | 0 | 0 |  | HD21 | OE1 |
| VAL503 | PHE327 | 6.97 | -0.02 | 0 | 0 |  | H | H |
| TYR453 | ASP38 | 6.97 | -0.03 | 0 | -1 |  | HH | HB1 |
| GLY446 | HIS41 | 6.97 | -0.06 | 0 | 0 |  | H | HD2 |
| LYS417 | LEU29 | 6.99 | 0.46 | 1 | 0 |  | HZ1 | C |
| CYS488 | PHE28 | 6.99 | -0.10 | 0 | 0 |  | H | HD1 |
| LEU455 | LYS26 | 7.00 | -0.09 | 0 | 1 |  | HD12 | O |
| TYR489 | GLU23 | 7.00 | -0.06 | 0 | -1 |  | HE2 | O |
| PHE456 | ALA25 | 7.01 | -0.03 | 0 | 0 |  | HE1 | O |
| TYR449 | HIS34 | 7.03 | -0.07 | 0 | 0 |  | HE1 | O |
| THR478 | GLN24 | 7.03 | -0.05 | 0 | 0 |  | H | HE22 |
| THR500 | ASN49 | 7.06 | -0.05 | 0 | 0 |  | HG22 | HD22 |
| TYR449 | GLU35 | 7.09 | -0.09 | 0 | -1 |  | HE1 | HA |
| GLY485 | GLU75 | 7.11 | -0.10 | 0 | -1 |  | HA1 | OE1 |
| GLN506 | PHE327 | 7.12 | -0.04 | 0 | 0 |  | HE22 | H |
| PHE486 | GLU75 | 7.13 | -0.13 | 0 | -1 |  | HD1 | HG1 |
| ARG403 | PRO389 | 7.15 | -0.04 | 1 | 0 |  | HH12 | HA |
| ASN439 | GLY326 | 7.15 | -0.02 | 0 | 0 |  | HD22 | HA1 |
| PHE497 | HIS41 | 7.16 | -0.08 | 0 | 0 |  | C | HD2 |
| TYR495 | GLU37 | 7.17 | -0.11 | 0 | -1 |  | HD1 | HG1 |
| LEU492 | GLU35 | 7.18 | -0.09 | 0 | -1 |  | O | OE2 |
| GLY496 | GLU42 | 7.19 | 0.00 | 0 | -1 |  | HA2 | HG2 |
| GLN498 | GLY352 | 7.21 | -0.08 | 0 | 0 |  | OE1 | O |
| PRO499 | ARG357 | 7.22 | 0.23 | 0 | 1 |  | C | HH11 |
| TYR453 | LYS31 | 7.22 | -0.18 | 0 | 1 |  | OH | HA |
| ASN501 | PHE327 | 7.23 | -0.03 | 0 | 0 |  | O | H |
| GLN493 | PHE32 | 7.23 | -0.06 | 0 | 0 |  | OE1 | N |
| TYR421 | GLU23 | 7.24 | -0.02 | 0 | -1 |  | HH | OE1 |
| ASP405 | ARG393 | 7.25 | -1.34 | -1 | 1 |  | OD2 | HH21 |
| SER477 | ILE21 | 7.26 | -0.04 | 0 | 0 |  | H | HG12 |
| GLY504 | ALA387 | 7.27 | -0.03 | 0 | 0 |  | HA2 | HA |
| ASN487 | GLU23 | 7.29 | -0.02 | 0 | -1 |  | OD1 | O |
| GLY485 | TYR83 | 7.30 | -0.04 | 0 | 0 |  | O | HH |
| TYR449 | GLU37 | 7.30 | 0.14 | 0 | -1 |  | HH | C |
| GLY502 | MET383 | 7.31 | -0.02 | 0 | 0 |  | HA2 | HG1 |
| ARG403 | GLN388 | 7.34 | -0.34 | 1 | 0 |  | HH22 | O |
| VAL503 | MET383 | 7.35 | -0.04 | 0 | 0 |  | HB | HE1 |
| ASN501 | GLU42 | 7.35 | -0.12 | 0 | -1 |  | HD22 | N |
| GLY502 | LEU351 | 7.36 | -0.01 | 0 | 0 |  | H | HB1 |
| PHE486 | GLN76 | 7.37 | -0.10 | 0 | 0 |  | HD1 | HE22 |
| PHE486 | ILE21 | 7.37 | -0.04 | 0 | 0 |  | HZ | HG22 |
| ALA475 | LYS26 | 7.37 | -0.17 | 0 | 1 |  | HB2 | C |
| GLY502 | ARG357 | 7.38 | -0.12 | 0 | 1 |  | N | HH11 |
| TYR449 | LEU45 | 7.39 | -0.02 | 0 | 0 |  | OH | HD11 |
| PHE456 | GLN24 | 7.39 | -0.05 | 0 | 0 |  | HE1 | O |
| GLY485 | PHE28 | 7.40 | -0.03 | 0 | 0 |  | O | HD1 |
| TYR505 | ALA36 | 7.41 | 0.19 | 0 | 0 |  | HH | C |
| ARG408 | ALA386 | 7.43 | 0.03 | 1 | 0 |  | HH12 | HB1 |
| ASN501 | GLN325 | 7.47 | -0.26 | 0 | 0 |  | O | HB1 |
| THR478 | SER19 | 7.48 | -0.12 | 0 | 1 |  | H | H1 |
| VAL503 | MET323 | 7.50 | 0.04 | 0 | 0 |  | HG21 | O |
| LYS417 | PHE28 | 7.50 | -0.16 | 1 | 0 |  | HZ1 | N |
| ASP405 | PHE356 | 7.51 | 0.03 | -1 | 0 |  | OD2 | HZ |
| ASN487 | PRO84 | 7.52 | -0.01 | 0 | 0 |  | HD22 | HD2 |
| ASN501 | GLU329 | 7.52 | 0.30 | 0 | -1 |  | O | OE1 |
| TYR508 | GLN325 | 7.54 | -0.06 | 0 | 0 |  | HH | OE1 |
| TYR421 | LYS26 | 7.55 | -0.07 | 0 | 1 |  | HE2 | HB2 |
| TYR421 | HIS34 | 7.55 | -0.05 | 0 | 0 |  | HD2 | HE1 |
| GLY496 | LEU45 | 7.56 | -0.02 | 0 | 0 |  | O | HD13 |
| TYR489 | GLU75 | 7.57 | -0.02 | 0 | -1 |  | HE1 | HG1 |
| CYS488 | THR27 | 7.58 | -0.07 | 0 | 0 |  | N | HB |
| ARG457 | ASP30 | 7.60 | -0.68 | 1 | -1 |  | H | OD2 |
| GLN498 | ASN43 | 7.62 | -0.05 | 0 | 0 |  | HE22 | H |
| PHE486 | LYS31 | 7.62 | -0.06 | 0 | 1 |  | HA | HG1 |
| GLN493 | ALA36 | 7.62 | -0.08 | 0 | 0 |  | HG1 | H |
| ASN448 | ASP38 | 7.65 | 0.05 | 0 | -1 |  | O | OD1 |
| SER494 | GLU37 | 7.69 | -0.01 | 0 | -1 |  | O | HB2 |
| PRO499 | LYS353 | 7.70 | -0.02 | 0 | 1 |  | N | HD1 |
| TYR505 | PHE40 | 7.70 | -0.04 | 0 | 0 |  | HH | HD2 |
| GLN498 | PHE40 | 7.71 | -0.03 | 0 | 0 |  | HE22 | C |
| ASN487 | GLN76 | 7.71 | -0.02 | 0 | 0 |  | H | HE22 |
| LYS444 | LEU45 | 7.73 | 0.00 | 1 | 0 |  | O | HD13 |
| GLN493 | GLU37 | 7.75 | -0.05 | 0 | -1 |  | HG2 | HB2 |
| GLY502 | ALA386 | 7.76 | 0.01 | 0 | 0 |  | HA2 | HB2 |
| GLN498 | ASN330 | 7.78 | -0.07 | 0 | 0 |  | HB2 | HD22 |
| GLN409 | HIS34 | 7.78 | -0.12 | 0 | 0 |  | HE22 | NE2 |
| ALA475 | ASP30 | 7.80 | 0.05 | 0 | -1 |  | HB2 | HB1 |
| SER477 | TYR83 | 7.80 | -0.02 | 0 | 0 |  | H | HE2 |
| ILE418 | HIS34 | 7.82 | -0.09 | 0 | 0 |  | HG11 | NE2 |
| GLY485 | GLN76 | 7.84 | -0.02 | 0 | 0 |  | O | HE22 |
| LEU455 | ALA36 | 7.84 | -0.02 | 0 | 0 |  | HD21 | H |
| ILE472 | LYS31 | 7.86 | 0.16 | 0 | 1 |  | HG23 | HE1 |
| GLY476 | GLU23 | 7.88 | 0.02 | 0 | -1 |  | N | O |
| TYR505 | THR324 | 7.90 | 0.01 | 0 | 0 |  | H | HG21 |
| VAL445 | HIS41 | 7.92 | -0.01 | 0 | 0 |  | HA | HE2 |
| GLY504 | GLU37 | 7.92 | 0.03 | 0 | -1 |  | O | HG1 |
| ARG454 | HIS34 | 7.93 | 0.05 | 1 | 0 |  | O | CE1 |
| TYR453 | ALA36 | 7.93 | -0.02 | 0 | 0 |  | HH | H |
| ASN439 | GLN325 | 7.93 | -0.04 | 0 | 0 |  | HD21 | HB1 |
| GLN493 | LEU39 | 7.94 | -0.04 | 0 | 0 |  | HE22 | HD11 |
| PHE486 | LEU97 | 7.94 | -0.01 | 0 | 0 |  | HE1 | HD21 |
| GLN493 | PHE72 | 7.96 | -0.01 | 0 | 0 |  | HE22 | HE1 |
| ARG454 | ASP30 | 7.96 | -0.76 | 1 | -1 |  | O | HB2 |
| TYR473 | TYR83 | 7.96 | -0.03 | 0 | 0 |  | HE2 | HH |
| GLY496 | LEU39 | 7.97 | -0.01 | 0 | 0 |  | HA2 | N |
| TYR453 | LYS353 | 7.98 | 0.01 | 0 | 1 |  | HH | HE1 |
| SER477 | THR20 | 7.98 | -0.05 | 0 | 0 |  | H | N |
| THR500 | GLU42 | 7.99 | -0.08 | 0 | -1 |  | HG1 | HA |
| TYR489 | THR82 | 8.00 | -0.02 | 0 | 0 |  | OH | HG1 |

**Supplementary** **table 9**. Interactions between SARS-CoV-2 RBD e *Macaca nemestrina* ACE2.

| **Interaction** | **SARS-CoV-2  RBD** | ***Macaca nemestrina* ACE2** |
| --- | --- | --- |
|  | Residue | Residue |
| Hydrophobic | TYR489 | PHE28 |
|  | PHE486 | LEU79 |
|  | PHE486 | MET82 |
|  | TYR505 | ALA386 |
| Hydrogen bonds | GLY502 | GLY354 |
|  | ASN501 | LYS353 |
|  | TYR505 | ALA386 |
|  | GLN493 | GLU35 |
|  | ASN487 | TYR83 |
|  | THR500 | ASN330 |
|  | TYR505 | ARG393 |
|  | LYS417 | ASP30 |
|  | TYR449 | ASP38 |
|  | ASN487 | TYR83 |
|  | GLN493 | GLU35 |
|  | THR500 | ASN330 |
|  | THR500 | ASP355 |
| Ionic | LYS417 | ASP30 |
|  | GLU484 | LYS31 |
|  | ARG403 | GLU37 |
| Aromatic-Sulphur | PHE486 | MET82 |
| Cation-Pi | TYR449 | LYS353 |
|  | TYR489 | LYS31 |

**Supplementary table 10**. Quantum biochemistry between SARS-CoV-2 RBD e *Macaca nemestrina* ACE2.

| **Amino acid residues** | | **Distance** | **Interaction energy** | **Residue charge** | |  | **Residue atom** | |
| --- | --- | --- | --- | --- | --- | --- | --- | --- |
| SARS-CoV-2  RBD | *Macaca nemestrina* ACE2 | (Å) | (kcal mol-1) | SARS-CoV-2  RBD | *Macaca nemestrina* ACE2 |  | SARS-CoV-2  RBD | *Macaca nemestrina* ACE2 |
| LYS417 | ASP30 | 1.59 | -9.24 | 1 | -1 |  | HZ1 | OD2 |
| GLN493 | GLU35 | 1.77 | -5.79 | 0 | -1 |  | HE22 | OE2 |
| TYR449 | ASP38 | 1.82 | -10.70 | 0 | -1 |  | HH | OD1 |
| GLY502 | GLY354 | 1.88 | 0.95 | 0 | 0 |  | H | HA2 |
| ASN487 | TYR83 | 1.89 | -2.90 | 0 | 0 |  | HD22 | OH |
| THR500 | ASP355 | 2.04 | -7.21 | 0 | -1 |  | HG1 | OD2 |
| GLY496 | LYS353 | 2.08 | 0.22 | 0 | 1 |  | HA2 | HZ1 |
| ASN501 | LYS353 | 2.14 | -1.80 | 0 | 1 |  | HD22 | HB1 |
| TYR505 | ALA386 | 2.15 | 1.08 | 0 | 0 |  | HE1 | HB1 |
| GLN493 | HIS34 | 2.26 | -3.65 | 0 | 0 |  | HG1 | HB2 |
| PHE456 | ASP30 | 2.28 | -2.32 | 0 | -1 |  | HZ | HB1 |
| THR500 | ASN330 | 2.31 | -1.92 | 0 | 0 |  | HB | HD22 |
| ASN501 | ASP355 | 2.36 | -3.22 | 0 | -1 |  | HD21 | HB1 |
| TYR505 | LYS353 | 2.41 | -4.58 | 0 | 1 |  | HB2 | O |
| THR500 | GLY326 | 2.47 | -2.42 | 0 | 0 |  | HB | HA1 |
| GLN498 | TYR41 | 2.48 | -2.09 | 0 | 0 |  | HE22 | HE2 |
| PHE486 | LEU79 | 2.48 | -2.82 | 0 | 0 |  | HD1 | HD21 |
| PHE456 | LYS31 | 2.51 | -2.49 | 0 | 1 |  | HE2 | HB1 |
| PHE456 | THR27 | 2.53 | -1.38 | 0 | 0 |  | HZ | O |
| TYR473 | THR27 | 2.58 | -1.50 | 0 | 0 |  | HE2 | HG21 |
| TYR505 | GLY354 | 2.59 | -1.69 | 0 | 0 |  | HD1 | HA2 |
| THR500 | TYR41 | 2.59 | 0.54 | 0 | 0 |  | HG1 | HH |
| TYR489 | THR27 | 2.61 | -1.09 | 0 | 0 |  | HE2 | O |
| LEU455 | ASP30 | 2.65 | -1.26 | 0 | -1 |  | HD11 | HB2 |
| TYR453 | HIS34 | 2.66 | -2.09 | 0 | 0 |  | HH | CD2 |
| TYR489 | PHE28 | 2.66 | -3.32 | 0 | 0 |  | OH | HA |
| ALA475 | THR27 | 2.71 | -1.56 | 0 | 0 |  | HB3 | HB |
| GLN493 | LYS31 | 2.73 | -0.42 | 0 | 1 |  | HE22 | HZ2 |
| LEU455 | LYS31 | 2.75 | -1.21 | 0 | 1 |  | HD21 | HA |
| PHE486 | MET82 | 2.76 | -3.56 | 0 | 0 |  | HE1 | HE3 |
| TYR489 | LYS31 | 2.77 | -6.09 | 0 | 1 |  | HB2 | HZ3 |
| LEU455 | HIS34 | 2.80 | -3.33 | 0 | 0 |  | HD21 | HD1 |
| THR500 | GLU329 | 2.90 | -1.95 | 0 | -1 |  | HG21 | HB1 |
| TYR449 | GLN42 | 2.92 | -0.51 | 0 | 0 |  | HH | HE21 |
| TYR505 | ARG393 | 2.93 | -2.40 | 0 | 1 |  | HH | NH1 |
| GLY502 | LYS353 | 2.96 | -1.08 | 0 | 1 |  | H | O |
| ASN501 | GLY354 | 3.04 | -1.94 | 0 | 0 |  | HA | C |
| TYR449 | LYS353 | 3.11 | 0.47 | 0 | 1 |  | HH | HZ3 |
| ASN501 | TYR41 | 3.13 | -1.43 | 0 | 0 |  | HD22 | OH |
| ALA475 | GLN24 | 3.15 | -1.65 | 0 | 0 |  | O | HG1 |
| GLU484 | LYS31 | 3.16 | -5.93 | -1 | 1 |  | OE1 | HZ1 |
| GLY476 | GLN24 | 3.16 | -1.50 | 0 | 0 |  | HA1 | OE1 |
| GLY502 | THR324 | 3.17 | -0.87 | 0 | 0 |  | HA1 | HB |
| LYS417 | HIS34 | 3.17 | -0.59 | 1 | 0 |  | HE2 | HE1 |
| THR500 | ARG357 | 3.20 | -0.52 | 0 | 1 |  | HG1 | HH11 |
| GLN498 | GLN42 | 3.23 | -0.18 | 0 | 0 |  | HE22 | HE22 |
| GLN498 | LEU45 | 3.27 | -0.62 | 0 | 0 |  | HE21 | HD12 |
| ASN487 | GLN24 | 3.35 | -0.40 | 0 | 0 |  | HD22 | HB2 |
| ASN487 | PHE28 | 3.39 | -0.71 | 0 | 0 |  | OD1 | HB1 |
| TYR505 | GLU37 | 3.52 | -0.36 | 0 | -1 |  | HE2 | OE1 |
| ASN501 | GLY352 | 3.57 | -0.38 | 0 | 0 |  | HD21 | O |
| PHE490 | LYS31 | 3.69 | -1.52 | 0 | 1 |  | H | HZ3 |
| THR500 | GLY354 | 3.83 | -0.06 | 0 | 0 |  | O | O |
| GLN506 | GLN325 | 3.91 | -0.11 | 0 | 0 |  | HE22 | HB1 |
| ASN487 | THR27 | 3.91 | -0.34 | 0 | 0 |  | OD1 | HG1 |
| TYR505 | PHE356 | 4.06 | -0.25 | 0 | 0 |  | HE1 | HZ |
| GLY502 | PHE356 | 4.09 | -0.26 | 0 | 0 |  | HA2 | HE2 |
| PHE486 | TYR83 | 4.10 | -1.21 | 0 | 0 |  | HE1 | OH |
| GLY496 | ASP38 | 4.21 | -0.19 | 0 | -1 |  | HA2 | OD1 |
| TYR489 | TYR83 | 4.28 | -0.16 | 0 | 0 |  | HH | HH |
| GLY502 | ASP355 | 4.32 | -0.02 | 0 | -1 |  | H | N |
| GLN498 | LYS353 | 4.32 | -1.79 | 0 | 1 |  | OE1 | HD1 |
| ALA475 | SER19 | 4.34 | -0.55 | 0 | 1 |  | O | HB2 |
| SER477 | GLN24 | 4.35 | -0.81 | 0 | 0 |  | H | OE1 |
| THR500 | THR324 | 4.41 | -0.30 | 0 | 0 |  | O | HG1 |
| ASN501 | THR324 | 4.41 | -1.11 | 0 | 0 |  | O | HB |
| GLY446 | GLN42 | 4.42 | -0.83 | 0 | 0 |  | O | HE22 |
| ASN501 | GLY326 | 4.43 | -1.37 | 0 | 0 |  | O | H |
| ARG403 | ALA387 | 4.43 | 0.06 | 1 | 0 |  | HH22 | HA |
| THR500 | GLN325 | 4.48 | -0.90 | 0 | 0 |  | HA | C |
| PHE456 | HIS34 | 4.54 | -0.51 | 0 | 0 |  | HZ | HD1 |
| TYR505 | ALA387 | 4.55 | 0.11 | 0 | 0 |  | OH | HA |
| VAL503 | THR324 | 4.56 | -0.49 | 0 | 0 |  | H | HG22 |
| THR500 | PHE356 | 4.58 | -0.36 | 0 | 0 |  | O | H |
| TYR495 | LYS353 | 4.60 | 0.20 | 0 | 1 |  | C | HZ1 |
| VAL503 | GLN325 | 4.64 | -0.34 | 0 | 0 |  | HG22 | H |
| TYR489 | GLN24 | 4.65 | -0.25 | 0 | 0 |  | HH | O |
| GLY496 | TYR41 | 4.65 | -0.23 | 0 | 0 |  | O | HE2 |
| PHE456 | PHE28 | 4.77 | -0.21 | 0 | 0 |  | HZ | N |
| GLY502 | GLY326 | 4.81 | -0.31 | 0 | 0 |  | HA1 | H |
| GLN506 | GLY326 | 4.81 | 0.08 | 0 | 0 |  | HE21 | H |
| PHE456 | LYS26 | 4.84 | -0.33 | 0 | 1 |  | HE1 | O |
| GLY476 | SER19 | 4.86 | -0.09 | 0 | 1 |  | HA1 | HB2 |
| ASN487 | LEU79 | 4.87 | -0.45 | 0 | 0 |  | H | HD23 |
| ASP405 | ALA387 | 4.87 | -0.50 | -1 | 0 |  | OD2 | HB2 |
| TYR449 | TYR41 | 4.88 | -0.21 | 0 | 0 |  | HH | HD2 |
| TYR505 | GLY352 | 4.92 | -0.42 | 0 | 0 |  | HH | HA1 |
| PRO499 | GLY326 | 4.93 | -0.07 | 0 | 0 |  | O | HA1 |
| GLY502 | GLN325 | 4.93 | -0.54 | 0 | 0 |  | HA1 | H |
| GLN498 | ASP355 | 4.93 | -0.68 | 0 | -1 |  | HB1 | HB1 |
| THR500 | PHE327 | 4.95 | -0.28 | 0 | 0 |  | HB | N |
| PHE497 | LYS353 | 4.97 | -0.52 | 0 | 1 |  | N | HD2 |
| TYR489 | ASP30 | 5.00 | -0.31 | 0 | -1 |  | HE2 | HB1 |
| ARG403 | ALA386 | 5.01 | -0.54 | 1 | 0 |  | HH22 | O |
| PRO499 | GLU329 | 5.03 | -0.23 | 0 | -1 |  | HB1 | OE2 |
| ARG403 | GLU37 | 5.09 | -1.97 | 1 | -1 |  | HH12 | OE1 |
| LYS417 | THR27 | 5.11 | -0.30 | 1 | 0 |  | HZ1 | HA |
| TYR421 | ASP30 | 5.13 | -0.40 | 0 | -1 |  | HE2 | OD2 |
| PHE456 | PHE32 | 5.14 | -0.20 | 0 | 0 |  | HZ | H |
| ASN439 | GLN325 | 5.14 | -0.22 | 0 | 0 |  | HD21 | OE1 |
| TYR489 | LEU79 | 5.17 | -0.12 | 0 | 0 |  | HE1 | HD12 |
| ASN487 | ALA25 | 5.18 | 0.13 | 0 | 0 |  | HD22 | HA |
| GLY447 | GLN42 | 5.19 | -0.22 | 0 | 0 |  | HA2 | HE22 |
| PHE486 | PHE28 | 5.19 | -0.45 | 0 | 0 |  | HD1 | HD1 |
| THR500 | LEU45 | 5.21 | -0.10 | 0 | 0 |  | HG1 | HD21 |
| PRO499 | GLN325 | 5.26 | -0.62 | 0 | 0 |  | O | HB1 |
| GLY447 | TYR41 | 5.28 | -0.08 | 0 | 0 |  | HA1 | HE2 |
| GLN506 | THR324 | 5.29 | 0.05 | 0 | 0 |  | HE21 | HB |
| CYS488 | LYS31 | 5.29 | 0.04 | 0 | 1 |  | O | HE1 |
| LEU455 | GLU35 | 5.32 | -0.30 | 0 | -1 |  | HD21 | HG1 |
| GLY446 | LEU45 | 5.32 | -0.12 | 0 | 0 |  | HA1 | HD11 |
| PHE456 | LEU29 | 5.32 | -0.11 | 0 | 0 |  | HZ | C |
| ARG403 | HIS34 | 5.33 | -0.50 | 1 | 0 |  | HH11 | HD2 |
| ASN501 | GLN325 | 5.37 | -2.90 | 0 | 0 |  | O | H |
| ALA475 | PHE28 | 5.37 | -0.17 | 0 | 0 |  | HB3 | N |
| ARG403 | ARG393 | 5.39 | 1.49 | 1 | 1 |  | HH12 | HH22 |
| TYR505 | GLN388 | 5.39 | -0.11 | 0 | 0 |  | OH | O |
| SER494 | LYS353 | 5.43 | -0.21 | 0 | 1 |  | O | HZ1 |
| ASN501 | PHE356 | 5.45 | -0.42 | 0 | 0 |  | HA | H |
| ARG403 | LYS353 | 5.46 | 0.97 | 1 | 1 |  | HH12 | HA |
| SER494 | HIS34 | 5.47 | -0.54 | 0 | 0 |  | O | HB2 |
| VAL503 | GLY354 | 5.49 | -0.04 | 0 | 0 |  | H | HA2 |
| GLY504 | GLY354 | 5.49 | -0.22 | 0 | 0 |  | H | HA2 |
| GLY447 | LYS353 | 5.50 | 0.09 | 0 | 1 |  | HA2 | HZ3 |
| GLN498 | ARG357 | 5.50 | 0.21 | 0 | 1 |  | HG1 | HH12 |
| SER477 | SER19 | 5.50 | -0.02 | 0 | 1 |  | HB2 | H1 |
| ARG408 | ALA387 | 5.57 | 0.09 | 1 | 0 |  | HH12 | HB1 |
| GLN498 | ASN330 | 5.57 | -0.09 | 0 | 0 |  | HB2 | HD22 |
| GLN493 | ASP38 | 5.63 | -0.47 | 0 | -1 |  | HE21 | HB1 |
| TYR505 | MET383 | 5.67 | -0.18 | 0 | 0 |  | HE1 | HA |
| GLY446 | TYR41 | 5.68 | -0.16 | 0 | 0 |  | H | HE2 |
| TYR489 | LEU29 | 5.70 | -0.29 | 0 | 0 |  | HE2 | N |
| ALA475 | TYR83 | 5.71 | -0.09 | 0 | 0 |  | HB3 | HH |
| PRO499 | TYR41 | 5.72 | -0.01 | 0 | 0 |  | HD1 | OH |
| GLU484 | GLU35 | 5.75 | 2.26 | -1 | -1 |  | OE1 | OE2 |
| GLY502 | MET383 | 5.75 | -0.01 | 0 | 0 |  | HA2 | HE1 |
| GLY485 | LYS31 | 5.75 | -0.39 | 0 | 1 |  | O | HE1 |
| ALA475 | GLU23 | 5.78 | -0.05 | 0 | -1 |  | HB3 | O |
| TYR505 | ASP355 | 5.80 | -0.40 | 0 | -1 |  | HD1 | N |
| TYR505 | ASP350 | 5.81 | -0.35 | 0 | -1 |  | HH | OD1 |
| TYR489 | GLN76 | 5.81 | -0.05 | 0 | 0 |  | HE1 | HE22 |
| THR500 | LYS353 | 5.82 | -0.62 | 0 | 1 |  | O | O |
| LEU492 | LYS31 | 5.83 | -0.45 | 0 | 1 |  | O | HZ3 |
| VAL503 | GLY326 | 5.84 | -0.05 | 0 | 0 |  | HG22 | H |
| PRO499 | ASN330 | 5.84 | 0.28 | 0 | 0 |  | HD1 | HD22 |
| LEU455 | PHE32 | 5.86 | -0.17 | 0 | 0 |  | HD21 | N |
| ASN501 | ASN330 | 5.87 | -0.18 | 0 | 0 |  | N | HD22 |
| GLN506 | GLY354 | 5.87 | -0.12 | 0 | 0 |  | HG2 | HA2 |
| GLN498 | ASP38 | 5.87 | 0.06 | 0 | -1 |  | HE22 | OD1 |
| TYR489 | LYS26 | 5.88 | -0.03 | 0 | 1 |  | HE2 | O |
| VAL503 | MET383 | 5.89 | -0.04 | 0 | 0 |  | H | HE1 |
| TYR473 | GLN24 | 5.91 | -0.08 | 0 | 0 |  | HE2 | HA |
| GLY485 | LEU79 | 5.92 | 0.00 | 0 | 0 |  | C | HD23 |
| PHE497 | TYR41 | 5.92 | -0.18 | 0 | 0 |  | C | HE2 |
| TYR489 | PHE32 | 5.92 | -0.28 | 0 | 0 |  | HE2 | H |
| THR500 | LEU351 | 5.93 | -0.12 | 0 | 0 |  | HG1 | HD22 |
| GLN506 | LYS353 | 5.94 | -0.24 | 0 | 1 |  | N | O |
| LEU455 | THR27 | 5.95 | -0.30 | 0 | 0 |  | HD21 | O |
| LYS417 | LYS26 | 5.96 | 0.99 | 1 | 1 |  | HZ1 | O |
| TYR421 | THR27 | 5.97 | -0.08 | 0 | 0 |  | HH | HG22 |
| VAL445 | ARG357 | 5.98 | 0.09 | 0 | 1 |  | HG12 | HH12 |
| ASN487 | MET82 | 5.98 | -0.40 | 0 | 0 |  | HD21 | HE1 |
| ASN487 | LYS31 | 6.00 | -0.13 | 0 | 1 |  | HA | HG1 |
| TYR489 | GLU35 | 6.03 | -0.29 | 0 | -1 |  | HB2 | OE2 |
| TYR505 | TYR385 | 6.06 | -0.04 | 0 | 0 |  | HH | O |
| GLN493 | ASP30 | 6.07 | -0.08 | 0 | -1 |  | HG1 | O |
| ASN501 | LEU351 | 6.09 | -0.08 | 0 | 0 |  | HD21 | HB1 |
| PHE456 | GLU35 | 6.09 | -0.03 | 0 | -1 |  | HE2 | HG1 |
| TYR505 | THR324 | 6.09 | -0.04 | 0 | 0 |  | HD1 | HG21 |
| GLY496 | GLN42 | 6.09 | -0.04 | 0 | 0 |  | HA2 | HE22 |
| ASN501 | LEU45 | 6.11 | -0.04 | 0 | 0 |  | HD22 | HD12 |
| LEU455 | ASN33 | 6.12 | -0.20 | 0 | 0 |  | HD11 | HB1 |
| ARG457 | THR27 | 6.13 | -0.06 | 1 | 0 |  | H | HG22 |
| ASN501 | ARG357 | 6.15 | -0.15 | 0 | 1 |  | HD21 | HH11 |
| VAL503 | PHE356 | 6.17 | -0.04 | 0 | 0 |  | H | HE2 |
| GLY496 | GLU37 | 6.19 | -0.03 | 0 | -1 |  | HA2 | HG1 |
| PHE486 | ALA80 | 6.19 | -0.08 | 0 | 0 |  | HE1 | N |
| GLN474 | THR27 | 6.20 | -0.01 | 0 | 0 |  | O | HG21 |
| TYR489 | ALA25 | 6.21 | -0.09 | 0 | 0 |  | HH | HA |
| LYS417 | LYS31 | 6.23 | 0.70 | 1 | 1 |  | HZ1 | N |
| GLY476 | THR27 | 6.25 | -0.08 | 0 | 0 |  | N | OG1 |
| GLY502 | GLY352 | 6.25 | 0.00 | 0 | 0 |  | H | O |
| GLN493 | ALA36 | 6.25 | -0.21 | 0 | 0 |  | HE21 | N |
| GLY447 | ASP38 | 6.25 | -0.04 | 0 | -1 |  | HA2 | OD1 |
| TYR495 | HIS34 | 6.30 | -0.21 | 0 | 0 |  | HA | HD2 |
| SER494 | ASP38 | 6.30 | -0.08 | 0 | -1 |  | HB2 | OD1 |
| TYR453 | ASP30 | 6.30 | -0.12 | 0 | -1 |  | HH | O |
| PRO499 | ASP355 | 6.32 | -0.88 | 0 | -1 |  | C | HB1 |
| ASP405 | ALA386 | 6.33 | 0.04 | -1 | 0 |  | OD2 | HB1 |
| VAL445 | ASN330 | 6.34 | -0.11 | 0 | 0 |  | HG12 | HD22 |
| PHE486 | GLN24 | 6.34 | -0.33 | 0 | 0 |  | HZ | HB2 |
| VAL445 | LEU45 | 6.43 | -0.13 | 0 | 0 |  | HG12 | HD21 |
| PRO491 | LYS31 | 6.44 | -0.41 | 0 | 1 |  | N | HZ3 |
| GLY502 | PHE327 | 6.44 | -0.01 | 0 | 0 |  | HA1 | H |
| ALA475 | LYS31 | 6.46 | 0.02 | 0 | 1 |  | HB1 | HB1 |
| TYR495 | ASP38 | 6.50 | 0.00 | 0 | -1 |  | O | OD1 |
| PHE456 | ASN33 | 6.50 | -0.10 | 0 | 0 |  | HZ | H |
| TYR473 | GLU23 | 6.51 | -0.05 | 0 | -1 |  | HE2 | O |
| PHE486 | THR78 | 6.51 | -0.07 | 0 | 0 |  | HE1 | O |
| TYR505 | ASP382 | 6.51 | -0.13 | 0 | -1 |  | HE1 | O |
| TYR505 | PHE390 | 6.52 | -0.09 | 0 | 0 |  | HH | HA |
| THR478 | GLN24 | 6.53 | -0.09 | 0 | 0 |  | H | OE1 |
| PHE490 | GLU35 | 6.53 | 0.00 | 0 | -1 |  | HB2 | OE2 |
| TYR505 | HIS34 | 6.54 | -0.04 | 0 | 0 |  | HE2 | HD2 |
| GLN493 | GLU37 | 6.55 | -0.14 | 0 | -1 |  | HE21 | HB2 |
| ASN439 | GLY326 | 6.57 | -0.04 | 0 | 0 |  | HD22 | H |
| GLY504 | LYS353 | 6.57 | -0.57 | 0 | 1 |  | H | O |
| GLY476 | TYR83 | 6.57 | -0.06 | 0 | 0 |  | HA2 | OH |
| TYR473 | LYS31 | 6.61 | -0.29 | 0 | 1 |  | HD2 | HB1 |
| TYR473 | PHE28 | 6.62 | -0.07 | 0 | 0 |  | HE2 | N |
| THR500 | GLY352 | 6.62 | -0.03 | 0 | 0 |  | O | O |
| SER494 | GLU35 | 6.64 | -0.10 | 0 | -1 |  | H | OE2 |
| GLN498 | GLY352 | 6.64 | -0.10 | 0 | 0 |  | HB1 | O |
| GLY446 | LYS353 | 6.65 | -0.15 | 0 | 1 |  | O | HZ3 |
| VAL445 | TYR41 | 6.67 | -0.09 | 0 | 0 |  | HA | OH |
| ASN439 | GLU329 | 6.69 | -0.07 | 0 | -1 |  | HD22 | HG2 |
| GLY502 | ALA386 | 6.69 | -0.01 | 0 | 0 |  | HA2 | HB1 |
| ASN501 | PHE327 | 6.70 | 0.00 | 0 | 0 |  | O | H |
| TYR449 | LEU39 | 6.70 | -0.16 | 0 | 0 |  | HH | N |
| ASN437 | GLN325 | 6.76 | -0.03 | 0 | 0 |  | HD22 | OE1 |
| GLN493 | ASN33 | 6.76 | 0.08 | 0 | 0 |  | HG1 | C |
| GLY504 | THR324 | 6.77 | -0.01 | 0 | 0 |  | H | HG21 |
| PRO499 | THR324 | 6.79 | -0.33 | 0 | 0 |  | O | HB |
| LYS444 | TYR41 | 6.79 | -0.07 | 1 | 0 |  | O | HE2 |
| LEU492 | GLU35 | 6.79 | 0.06 | 0 | -1 |  | O | OE2 |
| LEU455 | LEU29 | 6.80 | -0.02 | 0 | 0 |  | HD21 | O |
| ASN501 | GLU37 | 6.83 | -0.24 | 0 | -1 |  | HD22 | HG2 |
| ASN487 | LEU29 | 6.83 | -0.06 | 0 | 0 |  | OD1 | H |
| GLN493 | PHE32 | 6.83 | -0.15 | 0 | 0 |  | HE22 | O |
| GLY446 | ASP38 | 6.84 | 0.07 | 0 | -1 |  | O | OD1 |
| ALA475 | ALA25 | 6.84 | -0.07 | 0 | 0 |  | HB3 | N |
| GLY504 | PHE356 | 6.87 | -0.03 | 0 | 0 |  | H | HE2 |
| TYR453 | ASN33 | 6.88 | 0.02 | 0 | 0 |  | HH | HB2 |
| ASN448 | LYS353 | 6.88 | -0.12 | 0 | 1 |  | H | HZ3 |
| VAL503 | LYS353 | 6.88 | -0.05 | 0 | 1 |  | N | O |
| THR500 | TRP328 | 6.89 | -0.05 | 0 | 0 |  | HG21 | C |
| THR500 | SER331 | 6.90 | -0.06 | 0 | 0 |  | HB | H |
| TYR505 | PRO389 | 6.90 | -0.03 | 0 | 0 |  | OH | HA |
| TYR449 | GLU37 | 6.91 | 0.11 | 0 | -1 |  | HH | C |
| PHE486 | GLN76 | 6.93 | -0.11 | 0 | 0 |  | HA | HE22 |
| ALA475 | LYS26 | 6.93 | -0.14 | 0 | 1 |  | HB3 | C |
| GLU406 | HIS34 | 6.99 | 0.18 | -1 | 0 |  | OE2 | NE2 |
| GLY504 | ALA387 | 7.00 | -0.03 | 0 | 0 |  | HA2 | HB2 |
| ASN448 | TYR41 | 7.00 | -0.03 | 0 | 0 |  | H | HE2 |
| ILE472 | LYS31 | 7.01 | 0.19 | 0 | 1 |  | HG23 | HZ3 |
| SER494 | GLU37 | 7.02 | 0.07 | 0 | -1 |  | O | HB2 |
| PRO499 | ARG357 | 7.03 | 0.17 | 0 | 1 |  | HD1 | HH12 |
| TYR449 | LEU45 | 7.03 | -0.04 | 0 | 0 |  | OH | HD12 |
| CYS488 | PHE28 | 7.03 | -0.08 | 0 | 0 |  | H | HD1 |
| GLY446 | ARG357 | 7.05 | 0.07 | 0 | 1 |  | H | HH12 |
| GLY504 | ALA386 | 7.06 | -0.06 | 0 | 0 |  | HA2 | HB1 |
| LYS417 | ASN33 | 7.07 | 0.15 | 1 | 0 |  | HE2 | HB1 |
| LEU455 | PHE28 | 7.08 | -0.09 | 0 | 0 |  | HD21 | O |
| CYS488 | LEU79 | 7.09 | -0.04 | 0 | 0 |  | H | HD23 |
| TYR495 | GLU37 | 7.09 | -0.10 | 0 | -1 |  | HA | HG1 |
| ARG403 | GLY354 | 7.10 | -0.12 | 1 | 0 |  | HH22 | HA2 |
| TYR473 | SER19 | 7.11 | -0.05 | 0 | 1 |  | OH | HG |
| TYR473 | ASP30 | 7.12 | -0.05 | 0 | -1 |  | HE2 | HB1 |
| GLN506 | ASP355 | 7.12 | -0.10 | 0 | -1 |  | HE21 | HA |
| LYS417 | LEU29 | 7.12 | 0.52 | 1 | 0 |  | HZ1 | C |
| GLY447 | LEU45 | 7.15 | -0.02 | 0 | 0 |  | HA2 | HD12 |
| ASN487 | GLU23 | 7.17 | -0.06 | 0 | -1 |  | OD1 | O |
| LEU455 | LYS26 | 7.17 | -0.09 | 0 | 1 |  | HD11 | O |
| GLY485 | PHE28 | 7.20 | -0.02 | 0 | 0 |  | O | HD1 |
| PHE486 | ALA25 | 7.21 | -0.08 | 0 | 0 |  | HE1 | HA |
| TYR453 | LYS31 | 7.21 | -0.16 | 0 | 1 |  | HH | HA |
| ASN487 | GLN76 | 7.23 | -0.04 | 0 | 0 |  | H | HE22 |
| ASN501 | GLU329 | 7.23 | 0.05 | 0 | -1 |  | N | HB1 |
| PHE486 | LYS31 | 7.24 | -0.08 | 0 | 1 |  | HA | HG1 |
| VAL445 | GLU329 | 7.27 | -0.14 | 0 | -1 |  | HG23 | HB2 |
| PHE497 | ASP38 | 7.27 | 0.01 | 0 | -1 |  | N | OD1 |
| GLN506 | GLU329 | 7.28 | -0.09 | 0 | -1 |  | HE21 | HG2 |
| GLN474 | GLN24 | 7.28 | -0.11 | 0 | 0 |  | O | HG1 |
| VAL445 | ASN49 | 7.28 | -0.02 | 0 | 0 |  | HG11 | HD22 |
| SER494 | LYS31 | 7.28 | -0.07 | 0 | 1 |  | H | HZ2 |
| ALA475 | THR20 | 7.32 | 0.04 | 0 | 0 |  | O | O |
| ASN487 | ILE21 | 7.33 | -0.05 | 0 | 0 |  | HD22 | O |
| TYR489 | GLU23 | 7.34 | -0.06 | 0 | -1 |  | HE2 | O |
| ARG403 | GLN388 | 7.34 | -0.23 | 1 | 0 |  | HH12 | O |
| GLY502 | TYR41 | 7.35 | -0.04 | 0 | 0 |  | H | HH |
| GLN498 | GLY326 | 7.36 | -0.07 | 0 | 0 |  | HB2 | HA1 |
| GLN493 | PHE72 | 7.36 | -0.01 | 0 | 0 |  | HE22 | HE1 |
| VAL503 | MET323 | 7.37 | -0.01 | 0 | 0 |  | HG23 | O |
| TYR453 | GLU35 | 7.38 | 0.06 | 0 | -1 |  | OH | N |
| LYS458 | THR27 | 7.39 | -0.05 | 1 | 0 |  | HA | HG21 |
| GLY485 | GLN76 | 7.39 | -0.02 | 0 | 0 |  | O | HE22 |
| GLN493 | LYS353 | 7.39 | 0.24 | 0 | 1 |  | HE21 | HZ2 |
| GLN474 | SER19 | 7.39 | 0.02 | 0 | 1 |  | O | HB1 |
| TYR449 | HIS34 | 7.40 | -0.05 | 0 | 0 |  | HE1 | O |
| TYR473 | LYS26 | 7.41 | -0.08 | 0 | 1 |  | HE2 | C |
| TYR421 | HIS34 | 7.42 | -0.03 | 0 | 0 |  | HE2 | HE1 |
| PRO499 | LEU45 | 7.42 | -0.04 | 0 | 0 |  | HD1 | HD21 |
| GLY496 | GLY352 | 7.43 | 0.00 | 0 | 0 |  | O | O |
| ALA475 | ASP30 | 7.46 | 0.02 | 0 | -1 |  | HB3 | HB1 |
| TYR453 | GLU37 | 7.47 | -0.01 | 0 | -1 |  | OH | HB2 |
| VAL503 | PRO321 | 7.48 | 0.00 | 0 | 0 |  | HB | HB1 |
| CYS488 | THR27 | 7.49 | -0.08 | 0 | 0 |  | N | HB |
| PRO499 | GLY354 | 7.49 | 0.03 | 0 | 0 |  | O | O |
| GLY504 | MET383 | 7.49 | -0.04 | 0 | 0 |  | H | HE1 |
| GLN493 | LEU39 | 7.49 | -0.04 | 0 | 0 |  | HE21 | H |
| PHE456 | GLU23 | 7.50 | -0.13 | 0 | -1 |  | HE1 | O |
| ASN487 | LYS26 | 7.51 | -0.08 | 0 | 1 |  | OD1 | C |
| TYR505 | ASN33 | 7.53 | -0.04 | 0 | 0 |  | HE2 | O |
| GLY496 | HIS34 | 7.54 | -0.03 | 0 | 0 |  | H | HD2 |
| GLY502 | MET323 | 7.54 | 0.03 | 0 | 0 |  | HA1 | C |
| PHE456 | ALA25 | 7.56 | -0.01 | 0 | 0 |  | HZ | O |
| VAL445 | ASP355 | 7.57 | -0.15 | 0 | -1 |  | HG12 | OD2 |
| VAL503 | ASN322 | 7.58 | -0.10 | 0 | 0 |  | HG23 | O |
| GLN498 | GLY354 | 7.58 | 0.01 | 0 | 0 |  | O | HA2 |
| TYR453 | LYS353 | 7.59 | -0.12 | 0 | 1 |  | HE1 | HZ1 |
| PHE486 | GLN81 | 7.59 | -0.01 | 0 | 0 |  | HE1 | H |
| ARG403 | ASN33 | 7.61 | -0.13 | 1 | 0 |  | HH12 | HB2 |
| ARG403 | PRO389 | 7.62 | -0.07 | 1 | 0 |  | HH12 | HA |
| PHE486 | GLU75 | 7.64 | -0.16 | 0 | -1 |  | HD1 | HG1 |
| ARG457 | ASP30 | 7.65 | -0.62 | 1 | -1 |  | H | OD2 |
| GLN506 | PHE327 | 7.66 | -0.02 | 0 | 0 |  | HE21 | H |
| ASN487 | SER19 | 7.66 | -0.06 | 0 | 1 |  | HB2 | HB2 |
| LEU492 | HIS34 | 7.67 | -0.07 | 0 | 0 |  | C | HB2 |
| ASN501 | ASP38 | 7.67 | -0.04 | 0 | -1 |  | HD22 | HA |
| ASN487 | ALA80 | 7.68 | -0.03 | 0 | 0 |  | HD22 | HA |
| ASN487 | LEU97 | 7.71 | -0.02 | 0 | 0 |  | HD22 | HD21 |
| GLY496 | ASP355 | 7.72 | 0.06 | 0 | -1 |  | O | HB1 |
| ASN501 | GLN42 | 7.72 | -0.04 | 0 | 0 |  | HD22 | HE22 |
| PRO499 | PHE327 | 7.77 | -0.07 | 0 | 0 |  | O | H |
| GLY485 | MET82 | 7.78 | 0.02 | 0 | 0 |  | C | HE1 |
| LYS417 | PHE28 | 7.78 | -0.14 | 1 | 0 |  | HZ1 | N |
| GLY496 | LEU45 | 7.79 | -0.02 | 0 | 0 |  | O | HD12 |
| TYR505 | LEU351 | 7.82 | 0.01 | 0 | 0 |  | HH | C |
| VAL503 | ASP355 | 7.83 | 0.00 | 0 | -1 |  | H | HA |
| TYR489 | HIS34 | 7.84 | -0.05 | 0 | 0 |  | HD2 | HD1 |
| ALA475 | LEU29 | 7.87 | -0.03 | 0 | 0 |  | HB3 | H |
| ASP405 | GLY354 | 7.88 | 0.05 | -1 | 0 |  | OD2 | HA2 |
| PRO507 | LYS353 | 7.89 | -0.03 | 0 | 1 |  | HD2 | O |
| GLY476 | THR20 | 7.91 | -0.01 | 0 | 0 |  | HA1 | O |
| GLY446 | ASP355 | 7.93 | -0.10 | 0 | -1 |  | H | OD2 |
| PHE486 | PRO84 | 7.94 | -0.05 | 0 | 0 |  | HZ | HD2 |
| LEU455 | ALA36 | 7.97 | -0.02 | 0 | 0 |  | HD21 | H |
| ASN448 | GLN42 | 7.99 | -0.02 | 0 | 0 |  | H | HE22 |
| TYR505 | ALA384 | 7.99 | -0.04 | 0 | 0 |  | HE1 | N |

**Supplementary table 11**. Interactions between SARS-CoV-2 RBD e *Pan paniscus* ACE2.

| **Interaction** | **SARS-CoV-2  RBD** | ***Pan paniscus*  ACE2** |
| --- | --- | --- |
|  | Residue | Residue |
| Hydrophobic | TYR489 | PHE28 |
|  | PHE486 | LEU79 |
|  | PHE486 | MET82 |
|  | TYR505 | ALA386 |
| Hydrogen bonds | GLY502 | GLY354 |
|  | GLY496 | LYS353 |
|  | ASN501 | LYS353 |
|  | TYR505 | ALA386 |
|  | GLU484 | LYS31 |
|  | ASN487 | TYR83 |
|  | TYR449 | LYS353 |
|  | GLN498 | LYS353 |
|  | TYR505 | ARG393 |
|  | LYS417 | ASP30 |
|  | TYR449 | ASP38 |
|  | ASN487 | TYR83 |
|  | THR500 | ASP355 |
| Ionic | LYS417 | ASP30 |
|  | GLU484 | LYS31 |
|  | ARG403 | GLU37 |
| Aromatic-Sulphur | PHE486 | MET82 |
| Cation-Pi | TYR449 | LYS353 |
|  | TYR489 | LYS31 |
|  | TYR505 | ARG393 |

**Supplementary** **table 12**. Quantum biochemistry between SARS-CoV-2 RBD e *Pan paniscus* ACE2.

| **Amino acid residues** | | **Distance** | **Interaction energy** | **Residue charge** | |  | **Residue atom** | |
| --- | --- | --- | --- | --- | --- | --- | --- | --- |
| SARS-CoV-2  RBD | *Pan paniscus* ACE2 | (Å) | (kcal mol-1) | SARS-CoV-2  RBD | *Pan paniscus* ACE2 |  | SARS-CoV-2  RBD | *Pan paniscus* ACE2 |
| LYS417 | ASP30 | 1.59 | -10.87 | 1 | -1 |  | HZ1 | OD2 |
| TYR505 | ALA386 | 1.67 | -5.28 | 0 | 0 |  | HH | O |
| TYR449 | ASP38 | 1.72 | -8.17 | 0 | -1 |  | HH | OD1 |
| GLU484 | LYS31 | 1.81 | -9.78 | -1 | 1 |  | OE1 | HZ3 |
| THR500 | ASN330 | 1.93 | -0.09 | 0 | 0 |  | HG21 | HD22 |
| ASN487 | TYR83 | 2.02 | -4.58 | 0 | 0 |  | OD1 | HH |
| GLN498 | LYS353 | 2.05 | -6.09 | 0 | 1 |  | OE1 | HZ3 |
| GLY496 | LYS353 | 2.08 | -0.75 | 0 | 1 |  | HA2 | HZ1 |
| LEU455 | HIS34 | 2.09 | -2.83 | 0 | 0 |  | HD22 | HD1 |
| THR500 | ASP355 | 2.13 | -7.47 | 0 | -1 |  | HG1 | OD2 |
| TYR449 | LYS353 | 2.14 | 1.09 | 0 | 1 |  | HH | HZ2 |
| TYR489 | PHE28 | 2.18 | -2.07 | 0 | 0 |  | HH | HD1 |
| GLN498 | TYR41 | 2.23 | -2.73 | 0 | 0 |  | HG1 | HE2 |
| PHE456 | ASP30 | 2.25 | -1.86 | 0 | -1 |  | HE1 | HB2 |
| TYR489 | LYS31 | 2.25 | -5.39 | 0 | 1 |  | HB2 | HD2 |
| TYR473 | THR27 | 2.32 | -1.49 | 0 | 0 |  | HE2 | HG23 |
| VAL503 | THR324 | 2.32 | -1.29 | 0 | 0 |  | HG21 | HG23 |
| ALA475 | GLN24 | 2.33 | -2.74 | 0 | 0 |  | O | HG1 |
| PHE456 | THR27 | 2.37 | -2.30 | 0 | 0 |  | HE1 | HA |
| THR500 | GLY326 | 2.38 | -2.49 | 0 | 0 |  | HG22 | HA1 |
| PHE456 | LYS31 | 2.39 | -2.60 | 0 | 1 |  | HZ | HB1 |
| PHE486 | MET82 | 2.40 | -2.35 | 0 | 0 |  | HE1 | HE1 |
| ASN501 | LYS353 | 2.40 | -3.66 | 0 | 1 |  | HD22 | HD1 |
| PHE486 | LEU79 | 2.42 | -3.22 | 0 | 0 |  | HD1 | HD21 |
| LEU455 | ASP30 | 2.42 | -1.97 | 0 | -1 |  | HD22 | O |
| GLY502 | GLY354 | 2.42 | 0.10 | 0 | 0 |  | H | O |
| TYR453 | HIS34 | 2.49 | -2.42 | 0 | 0 |  | HH | CD2 |
| TYR505 | LYS353 | 2.50 | -1.78 | 0 | 1 |  | HD2 | HA |
| GLY502 | THR324 | 2.54 | -1.18 | 0 | 0 |  | HA1 | HG22 |
| ASN501 | ASP355 | 2.56 | -4.31 | 0 | -1 |  | HD21 | HB1 |
| THR500 | ARG357 | 2.76 | -0.15 | 0 | 1 |  | HG1 | HH11 |
| THR500 | TYR41 | 2.82 | 0.11 | 0 | 0 |  | HG1 | HH |
| GLN493 | HIS34 | 2.84 | -2.03 | 0 | 0 |  | HG1 | HB1 |
| ALA475 | THR27 | 2.94 | -1.08 | 0 | 0 |  | HB3 | OG1 |
| ASP405 | ALA387 | 3.02 | -1.73 | -1 | 0 |  | OD1 | HB2 |
| GLY502 | LYS353 | 3.04 | -1.24 | 0 | 1 |  | H | O |
| GLY502 | PHE356 | 3.04 | -0.37 | 0 | 0 |  | HA2 | HE2 |
| TYR505 | ARG393 | 3.06 | -1.40 | 0 | 1 |  | OH | CZ |
| TYR449 | GLN42 | 3.07 | -0.54 | 0 | 0 |  | HH | HE21 |
| TYR489 | THR27 | 3.08 | -1.44 | 0 | 0 |  | HE2 | O |
| TYR505 | GLY354 | 3.12 | -1.55 | 0 | 0 |  | HB1 | HA2 |
| PHE490 | LYS31 | 3.23 | 0.21 | 0 | 1 |  | H | HZ3 |
| LEU455 | LYS31 | 3.24 | -0.99 | 0 | 1 |  | HD21 | HA |
| TYR489 | LEU79 | 3.25 | -0.48 | 0 | 0 |  | HH | HD22 |
| ASN501 | GLY354 | 3.25 | -7.15 | 0 | 0 |  | HA | C |
| ARG403 | ALA387 | 3.33 | -0.47 | 1 | 0 |  | HH22 | HA |
| TYR505 | GLU37 | 3.35 | -0.75 | 0 | -1 |  | HE2 | OE1 |
| THR500 | GLU329 | 3.35 | -0.91 | 0 | -1 |  | HG22 | HB1 |
| ASN487 | LEU79 | 3.48 | -0.79 | 0 | 0 |  | H | HD21 |
| TYR505 | ALA387 | 3.48 | -2.70 | 0 | 0 |  | HH | HA |
| ARG408 | ALA387 | 3.58 | -0.21 | 1 | 0 |  | HH12 | HB2 |
| GLN493 | LYS31 | 3.65 | -0.45 | 0 | 1 |  | HE21 | HZ3 |
| ASN501 | TYR41 | 3.67 | -0.93 | 0 | 0 |  | HD22 | OH |
| GLY502 | ASP355 | 3.71 | -0.64 | 0 | -1 |  | H | N |
| GLY476 | GLN24 | 3.76 | -0.87 | 0 | 0 |  | HA1 | CD |
| ASN487 | GLN24 | 3.87 | -0.59 | 0 | 0 |  | OD1 | HB2 |
| GLN498 | LEU45 | 3.88 | -0.38 | 0 | 0 |  | HG1 | HD11 |
| GLN506 | THR324 | 3.94 | -0.12 | 0 | 0 |  | HE22 | HG23 |
| GLN498 | ASP38 | 3.98 | -0.05 | 0 | -1 |  | HE22 | OD1 |
| ARG403 | ALA386 | 3.98 | -0.36 | 1 | 0 |  | HH22 | O |
| GLN493 | GLU35 | 4.02 | -1.45 | 0 | -1 |  | HE21 | OE2 |
| GLN498 | GLN42 | 4.02 | -0.20 | 0 | 0 |  | HE22 | HE22 |
| GLY496 | ASP38 | 4.04 | -0.16 | 0 | -1 |  | HA2 | OD1 |
| THR500 | PHE356 | 4.08 | -0.56 | 0 | 0 |  | O | H |
| TYR473 | SER19 | 4.11 | -0.55 | 0 | 1 |  | HH | OG |
| THR500 | GLY354 | 4.11 | -0.47 | 0 | 0 |  | O | O |
| LYS417 | THR27 | 4.28 | -0.21 | 1 | 0 |  | HZ1 | HG21 |
| THR500 | GLN325 | 4.28 | -0.32 | 0 | 0 |  | HG22 | O |
| ALA475 | SER19 | 4.33 | -1.19 | 0 | 1 |  | O | HB1 |
| GLN506 | GLN325 | 4.34 | -0.09 | 0 | 0 |  | HE22 | H |
| ASN501 | GLY352 | 4.34 | -0.30 | 0 | 0 |  | HD21 | O |
| PHE497 | LYS353 | 4.36 | -0.93 | 0 | 1 |  | N | HZ3 |
| TYR421 | THR27 | 4.36 | -0.13 | 0 | 0 |  | HH | HG21 |
| ASN487 | PHE28 | 4.38 | -0.57 | 0 | 0 |  | OD1 | HB1 |
| ALA475 | TYR83 | 4.40 | -0.27 | 0 | 0 |  | HB2 | HH |
| GLY446 | GLN42 | 4.42 | -0.78 | 0 | 0 |  | O | HE22 |
| TYR505 | GLN388 | 4.45 | -0.26 | 0 | 0 |  | HH | O |
| TYR489 | TYR83 | 4.49 | -0.34 | 0 | 0 |  | OH | OH |
| TYR505 | PHE356 | 4.51 | -0.50 | 0 | 0 |  | HE1 | HZ |
| ASN501 | THR324 | 4.53 | -0.31 | 0 | 0 |  | O | HB |
| ARG403 | ARG393 | 4.54 | 1.94 | 1 | 1 |  | HH12 | HH21 |
| SER477 | GLN24 | 4.58 | -0.35 | 0 | 0 |  | HG | HE22 |
| LYS417 | HIS34 | 4.60 | -0.20 | 1 | 0 |  | HG2 | HE1 |
| GLY502 | MET383 | 4.62 | -0.22 | 0 | 0 |  | HA2 | HE2 |
| PHE456 | LYS26 | 4.65 | -0.15 | 0 | 1 |  | HE1 | O |
| TYR449 | TYR41 | 4.67 | -0.29 | 0 | 0 |  | HH | HD2 |
| ARG403 | HIS34 | 4.71 | -1.08 | 1 | 0 |  | HH11 | HD2 |
| TYR421 | ASP30 | 4.71 | -0.40 | 0 | -1 |  | HE2 | OD2 |
| PRO499 | GLY326 | 4.75 | 0.10 | 0 | 0 |  | O | HA1 |
| THR500 | PHE327 | 4.75 | -0.55 | 0 | 0 |  | HB | N |
| THR500 | THR324 | 4.77 | -0.07 | 0 | 0 |  | O | HB |
| GLY447 | LYS353 | 4.78 | -0.02 | 0 | 1 |  | HA2 | HZ3 |
| ASN487 | MET82 | 4.79 | -0.38 | 0 | 0 |  | HD22 | HB1 |
| GLU484 | GLU35 | 4.82 | 2.93 | -1 | -1 |  | OE1 | OE2 |
| TYR495 | LYS353 | 4.85 | 1.04 | 0 | 1 |  | C | HZ1 |
| ASN501 | GLY326 | 4.86 | -0.32 | 0 | 0 |  | O | HA2 |
| TYR489 | GLN76 | 4.88 | -0.19 | 0 | 0 |  | HH | HE22 |
| ALA475 | PHE28 | 4.92 | -0.18 | 0 | 0 |  | HB3 | H |
| TYR489 | GLN24 | 4.93 | -0.21 | 0 | 0 |  | HE2 | O |
| THR500 | LEU45 | 4.95 | -0.13 | 0 | 0 |  | HG1 | HD21 |
| GLY504 | GLY354 | 5.00 | -0.27 | 0 | 0 |  | H | HA2 |
| ALA475 | GLU23 | 5.00 | -0.37 | 0 | -1 |  | HB3 | O |
| ARG403 | GLU37 | 5.00 | -2.46 | 1 | -1 |  | HH12 | OE1 |
| PHE456 | PHE28 | 5.01 | -0.07 | 0 | 0 |  | HZ | HA |
| PHE456 | HIS34 | 5.03 | -0.43 | 0 | 0 |  | HZ | HD1 |
| ASP405 | ALA386 | 5.06 | 0.32 | -1 | 0 |  | OD2 | O |
| VAL503 | PHE356 | 5.07 | -0.14 | 0 | 0 |  | H | HE2 |
| VAL503 | GLN325 | 5.08 | -0.20 | 0 | 0 |  | HG21 | H |
| GLN498 | ASP355 | 5.10 | -0.41 | 0 | -1 |  | HB1 | HB1 |
| VAL503 | MET383 | 5.10 | -0.16 | 0 | 0 |  | H | HE1 |
| GLY446 | TYR41 | 5.10 | -0.24 | 0 | 0 |  | O | HE2 |
| PHE486 | TYR83 | 5.10 | -0.65 | 0 | 0 |  | HE1 | OH |
| TYR505 | TYR385 | 5.13 | -0.02 | 0 | 0 |  | HH | O |
| LEU455 | ASN33 | 5.13 | -0.32 | 0 | 0 |  | HD22 | HB1 |
| PHE456 | PHE32 | 5.15 | -0.20 | 0 | 0 |  | HZ | H |
| TYR473 | GLN24 | 5.17 | -0.16 | 0 | 0 |  | HH | HA |
| GLY504 | ALA387 | 5.17 | -0.08 | 0 | 0 |  | HA2 | HB2 |
| TYR473 | GLU23 | 5.22 | -0.35 | 0 | -1 |  | HH | O |
| ASN487 | THR27 | 5.26 | -0.28 | 0 | 0 |  | OD1 | HG1 |
| LYS417 | LYS26 | 5.29 | 1.02 | 1 | 1 |  | HZ1 | O |
| GLY504 | PHE356 | 5.29 | -0.08 | 0 | 0 |  | H | HE2 |
| VAL503 | GLY354 | 5.34 | 0.09 | 0 | 0 |  | H | O |
| ASN501 | PHE356 | 5.35 | -0.38 | 0 | 0 |  | HA | H |
| GLN506 | GLY326 | 5.36 | -0.11 | 0 | 0 |  | HE21 | HA2 |
| PRO499 | GLU329 | 5.37 | 0.04 | 0 | -1 |  | HB1 | OE1 |
| SER494 | HIS34 | 5.37 | -0.46 | 0 | 0 |  | O | HB2 |
| GLY502 | GLY326 | 5.39 | -0.03 | 0 | 0 |  | HA1 | HA2 |
| LEU455 | THR27 | 5.40 | -0.40 | 0 | 0 |  | O | HG21 |
| GLY446 | LEU45 | 5.41 | -0.10 | 0 | 0 |  | HA1 | HD13 |
| GLY476 | SER19 | 5.41 | -0.55 | 0 | 1 |  | HA1 | H3 |
| GLN474 | SER19 | 5.44 | -0.12 | 0 | 1 |  | O | OG |
| ARG457 | THR27 | 5.45 | -0.15 | 1 | 0 |  | H | HG21 |
| TYR489 | ASP30 | 5.48 | -0.39 | 0 | -1 |  | HE2 | HB1 |
| TYR505 | ASP355 | 5.48 | -0.36 | 0 | -1 |  | HB1 | N |
| GLY504 | ALA386 | 5.53 | -0.09 | 0 | 0 |  | HA2 | HB2 |
| GLN493 | ASP30 | 5.55 | -0.08 | 0 | -1 |  | HG1 | O |
| CYS488 | LYS31 | 5.56 | 0.06 | 0 | 1 |  | O | HZ3 |
| GLY504 | THR324 | 5.57 | -0.04 | 0 | 0 |  | H | HG23 |
| GLY485 | LYS31 | 5.58 | -0.68 | 0 | 1 |  | O | HE1 |
| SER494 | LYS353 | 5.58 | -0.19 | 0 | 1 |  | O | HZ1 |
| TYR505 | GLY352 | 5.60 | -0.35 | 0 | 0 |  | HE2 | C |
| ASN439 | GLN325 | 5.60 | -0.26 | 0 | 0 |  | HD21 | HB1 |
| TYR489 | LEU29 | 5.61 | -0.27 | 0 | 0 |  | HE2 | N |
| ARG408 | ARG559 | 5.62 | 1.36 | 1 | 1 |  | HH21 | HH22 |
| LEU455 | GLU35 | 5.62 | -0.24 | 0 | -1 |  | HD21 | HG1 |
| PRO499 | GLN325 | 5.62 | 0.00 | 0 | 0 |  | O | O |
| TYR453 | ASP30 | 5.62 | -0.22 | 0 | -1 |  | HH | O |
| ALA475 | ALA25 | 5.66 | -0.06 | 0 | 0 |  | HB3 | N |
| PHE486 | ALA80 | 5.69 | -0.13 | 0 | 0 |  | HE1 | N |
| GLY502 | ALA386 | 5.69 | 0.00 | 0 | 0 |  | HA2 | HB3 |
| PHE456 | LEU29 | 5.69 | -0.15 | 0 | 0 |  | HZ | C |
| GLN474 | THR27 | 5.70 | -0.30 | 0 | 0 |  | O | HG23 |
| GLY447 | GLN42 | 5.73 | -0.14 | 0 | 0 |  | HA2 | HE22 |
| GLY485 | LEU79 | 5.74 | -0.03 | 0 | 0 |  | O | HD11 |
| GLY447 | TYR41 | 5.75 | -0.12 | 0 | 0 |  | HA2 | HE2 |
| PRO499 | TYR41 | 5.76 | -0.18 | 0 | 0 |  | HD1 | OH |
| GLY476 | TYR83 | 5.78 | -0.09 | 0 | 0 |  | HA2 | HH |
| TYR495 | HIS34 | 5.78 | -0.29 | 0 | 0 |  | HA | HD2 |
| GLY446 | LYS353 | 5.79 | -0.12 | 0 | 1 |  | O | HZ3 |
| GLN506 | LYS353 | 5.81 | -0.14 | 0 | 1 |  | N | O |
| PHE486 | THR78 | 5.83 | 0.00 | 0 | 0 |  | HE1 | O |
| ARG403 | GLN388 | 5.84 | -0.29 | 1 | 0 |  | HH22 | O |
| TYR489 | PHE32 | 5.87 | -0.26 | 0 | 0 |  | HH | H |
| GLY502 | GLN325 | 5.88 | -0.07 | 0 | 0 |  | HA1 | H |
| GLN506 | GLY354 | 5.90 | -0.12 | 0 | 0 |  | HE21 | O |
| ARG403 | LYS353 | 5.90 | 0.93 | 1 | 1 |  | NH1 | HA |
| ASN501 | ASN330 | 5.91 | -0.31 | 0 | 0 |  | N | HD22 |
| THR500 | LEU351 | 5.93 | -0.11 | 0 | 0 |  | HG1 | HD22 |
| LEU455 | PHE32 | 5.93 | -0.20 | 0 | 0 |  | HD22 | N |
| ASN501 | ARG357 | 5.96 | -0.11 | 0 | 1 |  | HD21 | HH11 |
| ASN487 | LYS31 | 5.99 | -0.05 | 0 | 1 |  | H | HG1 |
| VAL503 | MET323 | 6.00 | -0.05 | 0 | 0 |  | HG21 | C |
| TYR453 | ASN33 | 6.03 | 0.05 | 0 | 0 |  | HH | O |
| PHE486 | PHE28 | 6.05 | -0.23 | 0 | 0 |  | HE1 | HD1 |
| LYS458 | SER19 | 6.06 | 0.98 | 1 | 1 |  | HA | HG |
| GLN498 | ARG357 | 6.07 | 0.07 | 0 | 1 |  | HB2 | HH11 |
| ASN501 | LEU45 | 6.07 | -0.05 | 0 | 0 |  | HD22 | HD11 |
| GLY502 | GLY352 | 6.08 | -0.01 | 0 | 0 |  | H | O |
| GLY496 | GLU37 | 6.08 | -0.02 | 0 | -1 |  | H | HG1 |
| GLY504 | LYS353 | 6.10 | -0.45 | 0 | 1 |  | C | O |
| PHE490 | GLU35 | 6.12 | -0.12 | 0 | -1 |  | HB2 | OE2 |
| PRO499 | ASN330 | 6.12 | 0.16 | 0 | 0 |  | C | HD22 |
| PHE456 | GLU35 | 6.12 | -0.02 | 0 | -1 |  | HE2 | HG1 |
| GLY496 | TYR41 | 6.13 | -0.09 | 0 | 0 |  | O | HE2 |
| ASN448 | LYS353 | 6.14 | -0.14 | 0 | 1 |  | H | HZ3 |
| LEU455 | LEU29 | 6.14 | -0.09 | 0 | 0 |  | HD22 | O |
| THR500 | LYS353 | 6.15 | -0.42 | 0 | 1 |  | O | O |
| TYR505 | PHE390 | 6.16 | -0.13 | 0 | 0 |  | OH | HA |
| GLY447 | ASP38 | 6.18 | 0.00 | 0 | -1 |  | HA2 | OD1 |
| CYS488 | LEU79 | 6.18 | -0.07 | 0 | 0 |  | H | HD21 |
| GLN474 | GLN24 | 6.20 | 0.01 | 0 | 0 |  | O | HG1 |
| ASN439 | GLY326 | 6.20 | -0.04 | 0 | 0 |  | HD22 | HA1 |
| TYR505 | HIS34 | 6.20 | -0.06 | 0 | 0 |  | HE2 | HD2 |
| ASN501 | GLN325 | 6.21 | -0.50 | 0 | 0 |  | O | H |
| GLU406 | HIS34 | 6.21 | 0.31 | -1 | 0 |  | OE2 | NE2 |
| TYR489 | GLU35 | 6.22 | -0.32 | 0 | -1 |  | HB2 | OE2 |
| TYR489 | LYS26 | 6.24 | -0.04 | 0 | 1 |  | HE2 | C |
| VAL503 | PRO321 | 6.26 | -0.06 | 0 | 0 |  | HG21 | HB1 |
| TYR473 | PHE28 | 6.28 | -0.11 | 0 | 0 |  | HE2 | H |
| ASN487 | ALA25 | 6.28 | -0.03 | 0 | 0 |  | OD1 | HA |
| TYR505 | MET383 | 6.29 | -0.13 | 0 | 0 |  | HE1 | HA |
| GLY502 | PHE327 | 6.32 | -0.02 | 0 | 0 |  | HA1 | H |
| SER494 | ASP38 | 6.33 | -0.16 | 0 | -1 |  | O | HB1 |
| ALA475 | THR20 | 6.33 | 0.06 | 0 | 0 |  | O | O |
| PRO499 | THR324 | 6.34 | -0.31 | 0 | 0 |  | O | HB |
| VAL503 | ASN322 | 6.38 | -0.05 | 0 | 0 |  | HG21 | O |
| TYR473 | LYS31 | 6.40 | -0.25 | 0 | 1 |  | HD2 | HB1 |
| GLY504 | MET383 | 6.41 | -0.05 | 0 | 0 |  | H | HE1 |
| SER477 | SER19 | 6.42 | -0.06 | 0 | 1 |  | HG | H3 |
| ASN501 | LEU351 | 6.42 | -0.04 | 0 | 0 |  | HD21 | HB1 |
| VAL445 | ARG357 | 6.42 | 0.05 | 0 | 1 |  | HG13 | HH12 |
| TYR505 | PRO389 | 6.45 | -0.08 | 0 | 0 |  | OH | HA |
| ASN487 | ALA80 | 6.45 | -0.10 | 0 | 0 |  | HD22 | HA |
| ARG403 | GLY354 | 6.49 | -0.15 | 1 | 0 |  | HH21 | HA2 |
| GLN498 | ASN330 | 6.51 | -0.06 | 0 | 0 |  | HB2 | HD22 |
| GLY446 | ASP38 | 6.53 | 0.10 | 0 | -1 |  | O | OD1 |
| PRO491 | LYS31 | 6.54 | -0.24 | 0 | 1 |  | HA | HD2 |
| PHE456 | ASN33 | 6.54 | -0.10 | 0 | 0 |  | HZ | H |
| GLY496 | GLN42 | 6.55 | -0.02 | 0 | 0 |  | HA2 | HE21 |
| GLN506 | ASP355 | 6.60 | -0.15 | 0 | -1 |  | HE21 | HA |
| ARG403 | PRO389 | 6.61 | -0.14 | 1 | 0 |  | HH12 | HA |
| PHE486 | LYS31 | 6.61 | -0.12 | 0 | 1 |  | HA | HE1 |
| ILE472 | LYS31 | 6.62 | 0.13 | 0 | 1 |  | HG23 | HZ3 |
| TYR505 | ASP382 | 6.63 | -0.08 | 0 | -1 |  | HH | O |
| ASN501 | PHE327 | 6.65 | -0.08 | 0 | 0 |  | O | H |
| TYR453 | LYS31 | 6.66 | -0.12 | 0 | 1 |  | HH | HA |
| LYS417 | LYS31 | 6.67 | 0.54 | 1 | 1 |  | HZ1 | H |
| PRO499 | ASP355 | 6.68 | -0.60 | 0 | -1 |  | C | OD2 |
| PHE486 | GLN76 | 6.68 | -0.12 | 0 | 0 |  | HA | HE22 |
| VAL445 | GLU329 | 6.69 | -0.14 | 0 | -1 |  | HG23 | OE1 |
| TYR453 | GLU35 | 6.69 | -0.03 | 0 | -1 |  | HH | N |
| TYR489 | ALA25 | 6.70 | -0.07 | 0 | 0 |  | HE2 | HA |
| GLN493 | ASN33 | 6.71 | -0.01 | 0 | 0 |  | HG1 | C |
| PHE497 | TYR41 | 6.71 | -0.11 | 0 | 0 |  | C | HE2 |
| TYR505 | THR324 | 6.71 | -0.02 | 0 | 0 |  | H | HG23 |
| ASN501 | GLU37 | 6.73 | -0.23 | 0 | -1 |  | HD22 | HG2 |
| LYS458 | THR27 | 6.75 | -0.06 | 1 | 0 |  | HA | HG23 |
| TYR449 | LEU39 | 6.76 | -0.21 | 0 | 0 |  | HH | N |
| TYR505 | ASN33 | 6.76 | -0.07 | 0 | 0 |  | HE2 | O |
| GLY496 | HIS34 | 6.76 | -0.07 | 0 | 0 |  | H | HD2 |
| SER494 | GLU35 | 6.76 | -0.20 | 0 | -1 |  | H | OE1 |
| GLY476 | THR27 | 6.77 | -0.03 | 0 | 0 |  | N | OG1 |
| ALA475 | ILE21 | 6.77 | -0.03 | 0 | 0 |  | O | HA |
| ASN487 | GLN76 | 6.78 | -0.03 | 0 | 0 |  | H | HE22 |
| PHE486 | GLN24 | 6.78 | -0.16 | 0 | 0 |  | HZ | OE1 |
| THR500 | TRP328 | 6.78 | -0.09 | 0 | 0 |  | HG22 | H |
| ALA475 | LYS26 | 6.79 | -0.16 | 0 | 1 |  | HB3 | H |
| LEU455 | LYS26 | 6.79 | -0.11 | 0 | 1 |  | HD13 | O |
| VAL503 | GLY326 | 6.80 | -0.02 | 0 | 0 |  | HG21 | H |
| CYS488 | PHE28 | 6.80 | -0.08 | 0 | 0 |  | H | HD1 |
| GLN498 | GLY352 | 6.80 | -0.09 | 0 | 0 |  | HB1 | O |
| TYR495 | GLU37 | 6.82 | -0.11 | 0 | -1 |  | HA | HG1 |
| TYR449 | LEU45 | 6.83 | -0.04 | 0 | 0 |  | HH | HD12 |
| ALA475 | LYS31 | 6.83 | 0.01 | 0 | 1 |  | HB1 | HB1 |
| ARG403 | ASN33 | 6.84 | -0.30 | 1 | 0 |  | HH12 | O |
| LYS417 | ASN33 | 6.84 | 0.12 | 1 | 0 |  | HE2 | HB1 |
| TYR495 | ASP38 | 6.88 | -0.13 | 0 | -1 |  | C | OD1 |
| ASN501 | MET383 | 6.89 | -0.05 | 0 | 0 |  | C | HE2 |
| TYR473 | ASP30 | 6.89 | -0.02 | 0 | -1 |  | HE2 | HB1 |
| VAL445 | ASN330 | 6.89 | -0.06 | 0 | 0 |  | HG13 | HD22 |
| THR500 | GLY352 | 6.90 | -0.03 | 0 | 0 |  | O | O |
| ASP405 | GLY354 | 6.91 | 0.08 | -1 | 0 |  | OD2 | HA2 |
| PRO499 | ARG357 | 6.93 | 0.18 | 0 | 1 |  | HD1 | HH12 |
| VAL503 | LYS353 | 6.93 | -0.04 | 0 | 1 |  | N | O |
| PHE456 | GLN24 | 6.94 | -0.04 | 0 | 0 |  | HE1 | O |
| PHE456 | GLU23 | 6.95 | -0.15 | 0 | -1 |  | HE1 | O |
| SER494 | GLU37 | 6.96 | -0.02 | 0 | -1 |  | O | HB2 |
| TYR449 | GLU37 | 6.96 | 0.10 | 0 | -1 |  | HH | C |
| PHE486 | GLU75 | 6.99 | -0.27 | 0 | -1 |  | HD1 | HG1 |
| TYR505 | ASP350 | 7.00 | -0.11 | 0 | -1 |  | HH | OD1 |
| TYR449 | HIS34 | 7.01 | -0.07 | 0 | 0 |  | HE1 | O |
| TYR473 | LYS26 | 7.01 | 0.01 | 0 | 1 |  | HE2 | C |
| ARG457 | ASP30 | 7.04 | -0.67 | 1 | -1 |  | H | OD2 |
| GLN506 | PHE327 | 7.04 | -0.02 | 0 | 0 |  | HE21 | H |
| TYR453 | GLU37 | 7.05 | -0.02 | 0 | -1 |  | HH | HB2 |
| PHE497 | ASP38 | 7.05 | 0.01 | 0 | -1 |  | N | OD1 |
| TYR489 | GLU23 | 7.06 | -0.03 | 0 | -1 |  | HE2 | O |
| ARG408 | PHE555 | 7.07 | -0.09 | 1 | 0 |  | HH22 | HZ |
| ASN439 | GLU329 | 7.08 | -0.07 | 0 | -1 |  | HD22 | OE1 |
| TYR489 | ALA80 | 7.08 | -0.03 | 0 | 0 |  | HH | HA |
| ASN439 | THR324 | 7.09 | -0.01 | 0 | 0 |  | HD22 | HB |
| ASP405 | GLN388 | 7.10 | 0.11 | -1 | 0 |  | OD2 | N |
| VAL445 | TYR41 | 7.10 | -0.09 | 0 | 0 |  | HA | OH |
| VAL445 | LEU45 | 7.15 | -0.05 | 0 | 0 |  | HG13 | HD22 |
| CYS488 | TYR83 | 7.15 | -0.05 | 0 | 0 |  | H | HH |
| PHE486 | GLN81 | 7.17 | -0.05 | 0 | 0 |  | HE1 | H |
| ARG408 | ALA386 | 7.18 | 0.04 | 1 | 0 |  | HH12 | C |
| ASN501 | ASP38 | 7.20 | -0.07 | 0 | -1 |  | HD22 | HA |
| TYR421 | GLU23 | 7.22 | -0.08 | 0 | -1 |  | HH | OE1 |
| TYR421 | LYS26 | 7.24 | -0.03 | 0 | 1 |  | HH | HB2 |
| LYS417 | LEU29 | 7.25 | 0.49 | 1 | 0 |  | HZ1 | C |
| VAL503 | ALA386 | 7.25 | -0.02 | 0 | 0 |  | H | HB3 |
| GLY446 | ARG357 | 7.25 | 0.07 | 0 | 1 |  | H | HH12 |
| THR500 | MET383 | 7.26 | -0.03 | 0 | 0 |  | O | HE2 |
| GLY476 | ILE21 | 7.28 | -0.01 | 0 | 0 |  | HA1 | HA |
| GLN409 | ALA387 | 7.30 | -0.04 | 0 | 0 |  | HE21 | HB1 |
| GLN493 | PHE32 | 7.30 | -0.10 | 0 | 0 |  | HG1 | N |
| THR500 | SER331 | 7.30 | -0.07 | 0 | 0 |  | HB | H |
| LYS444 | TYR41 | 7.31 | -0.06 | 1 | 0 |  | O | HE2 |
| LEU492 | LYS31 | 7.32 | -0.10 | 0 | 1 |  | O | HZ3 |
| GLY502 | MET323 | 7.33 | 0.04 | 0 | 0 |  | HA1 | C |
| GLN506 | PHE356 | 7.35 | -0.07 | 0 | 0 |  | HE21 | HE2 |
| GLU406 | ALA387 | 7.35 | 0.00 | -1 | 0 |  | HG2 | HB1 |
| ARG403 | PHE390 | 7.36 | -0.06 | 1 | 0 |  | HH12 | H |
| VAL503 | ASP355 | 7.36 | 0.03 | 0 | -1 |  | H | HA |
| GLN493 | ALA36 | 7.37 | -0.04 | 0 | 0 |  | HG1 | H |
| PRO499 | LEU45 | 7.42 | -0.06 | 0 | 0 |  | HD1 | HD11 |
| LEU455 | PHE28 | 7.42 | -0.07 | 0 | 0 |  | HD22 | O |
| CYS488 | THR27 | 7.43 | -0.07 | 0 | 0 |  | N | HB |
| ARG403 | TYR385 | 7.43 | -0.11 | 1 | 0 |  | HH22 | O |
| GLY447 | LEU45 | 7.44 | -0.03 | 0 | 0 |  | N | HD11 |
| ALA475 | LEU79 | 7.45 | -0.01 | 0 | 0 |  | HB2 | HD21 |
| SER477 | TYR83 | 7.46 | -0.03 | 0 | 0 |  | H | HE2 |
| GLY502 | TYR41 | 7.48 | -0.05 | 0 | 0 |  | H | HH |
| PRO499 | LYS353 | 7.53 | -0.05 | 0 | 1 |  | N | HD1 |
| ASN487 | GLU23 | 7.57 | 0.04 | 0 | -1 |  | OD1 | O |
| PRO499 | PHE327 | 7.58 | -0.04 | 0 | 0 |  | O | H |
| GLY476 | THR20 | 7.58 | 0.07 | 0 | 0 |  | HA1 | O |
| GLY485 | PHE28 | 7.59 | -0.02 | 0 | 0 |  | O | HD1 |
| TYR489 | HIS34 | 7.60 | -0.03 | 0 | 0 |  | HB2 | HB1 |
| GLN493 | GLU37 | 7.60 | -0.07 | 0 | -1 |  | HB2 | HB2 |
| ARG457 | SER19 | 7.60 | 0.66 | 1 | 1 |  | O | HG |
| VAL503 | PHE327 | 7.60 | -0.01 | 0 | 0 |  | HG21 | H |
| LEU492 | HIS34 | 7.62 | -0.07 | 0 | 0 |  | C | HB2 |
| LYS417 | PHE28 | 7.63 | -0.14 | 1 | 0 |  | HZ1 | N |
| ASP405 | PHE356 | 7.65 | 0.02 | -1 | 0 |  | OD2 | HZ |
| GLY485 | GLU75 | 7.66 | 0.01 | 0 | -1 |  | HA1 | OE1 |
| ALA475 | ASP30 | 7.68 | 0.02 | 0 | -1 |  | HB1 | HB1 |
| ARG403 | PHE356 | 7.69 | -0.08 | 1 | 0 |  | HH21 | HZ |
| PHE456 | ALA25 | 7.69 | -0.02 | 0 | 0 |  | HE1 | O |
| GLY485 | GLN76 | 7.69 | -0.03 | 0 | 0 |  | O | HE22 |
| ASP405 | ARG559 | 7.70 | -1.26 | -1 | 1 |  | OD1 | HH22 |
| GLN498 | GLY354 | 7.70 | -0.01 | 0 | 0 |  | HB1 | C |
| GLY404 | ALA387 | 7.70 | -0.03 | 0 | 0 |  | C | HB2 |
| GLN506 | MET383 | 7.70 | -0.01 | 0 | 0 |  | HE21 | HE2 |
| ASN437 | THR324 | 7.70 | 0.00 | 0 | 0 |  | HD22 | HG23 |
| TYR505 | TYR41 | 7.72 | -0.07 | 0 | 0 |  | HE2 | HB1 |
| LEU492 | GLU35 | 7.72 | -0.05 | 0 | -1 |  | O | OE2 |
| GLN498 | GLU37 | 7.73 | 0.09 | 0 | -1 |  | OE1 | HG2 |
| GLN506 | MET323 | 7.74 | 0.02 | 0 | 0 |  | HE22 | O |
| TYR473 | THR20 | 7.74 | -0.06 | 0 | 0 |  | HH | O |
| ASN437 | GLN325 | 7.76 | -0.02 | 0 | 0 |  | HD22 | OE1 |
| ILE418 | HIS34 | 7.77 | -0.06 | 0 | 0 |  | HD1 | NE2 |
| ALA475 | LEU29 | 7.77 | -0.03 | 0 | 0 |  | HB3 | H |
| LYS417 | GLU23 | 7.78 | -1.50 | 1 | -1 |  | HZ1 | O |
| PHE490 | HIS34 | 7.78 | -0.07 | 0 | 0 |  | O | HB1 |
| PRO491 | ASP30 | 7.78 | 0.09 | 0 | -1 |  | HA | HB2 |
| ASP405 | MET383 | 7.79 | 0.17 | -1 | 0 |  | OD2 | O |
| ASP405 | LYS353 | 7.80 | -0.78 | -1 | 1 |  | OD2 | O |
| THR478 | GLN24 | 7.81 | -0.04 | 0 | 0 |  | H | HE22 |
| GLY504 | ASP355 | 7.81 | -0.08 | 0 | -1 |  | H | N |
| GLN493 | ASP38 | 7.81 | -0.17 | 0 | -1 |  | HB2 | HB1 |
| ARG408 | GLN388 | 7.83 | -0.21 | 1 | 0 |  | HH12 | N |
| TYR449 | GLU35 | 7.83 | -0.08 | 0 | -1 |  | HE1 | HA |
| ASN448 | TYR41 | 7.83 | -0.02 | 0 | 0 |  | H | HE2 |
| ASN440 | GLN325 | 7.85 | -0.04 | 0 | 0 |  | HB2 | OE1 |
| TYR453 | LYS353 | 7.86 | -0.15 | 0 | 1 |  | HE1 | HZ1 |
| GLY496 | GLY352 | 7.87 | -0.02 | 0 | 0 |  | O | O |
| LYS444 | LYS353 | 7.88 | 0.72 | 1 | 1 |  | O | HZ3 |
| GLN498 | GLY326 | 7.89 | -0.06 | 0 | 0 |  | O | HA1 |
| SER443 | LYS353 | 7.90 | 0.11 | 0 | 1 |  | HB1 | HZ3 |
| TYR489 | PHE72 | 7.91 | -0.04 | 0 | 0 |  | HE1 | HE1 |
| GLU484 | GLN76 | 7.94 | -0.06 | -1 | 0 |  | HG1 | HE22 |
| GLY496 | ASP355 | 7.95 | 0.04 | 0 | -1 |  | O | HB1 |
| ASP405 | TYR385 | 7.95 | -0.10 | -1 | 0 |  | OD2 | C |
| TYR473 | ALA25 | 7.95 | -0.02 | 0 | 0 |  | HE2 | N |
| LEU455 | ALA36 | 7.96 | -0.01 | 0 | 0 |  | HD22 | H |
| TYR489 | MET82 | 7.97 | -0.03 | 0 | 0 |  | HH | HE1 |
| ASP405 | ARG393 | 7.97 | -1.22 | -1 | 1 |  | OD2 | HH21 |
| GLN474 | GLU23 | 7.98 | 0.05 | 0 | -1 |  | O | O |

**Supplementary** **table 13**. Interactions between SARS-CoV-2 RBD e *Sapajus apella* ACE2.

| **Interaction** | **SARS-CoV-2  RBD** | ***Sapajus apella*  ACE2** |
| --- | --- | --- |
|  | Residue | Residue |
| Hydrophobic | TYR489 | PHE28 |
|  | PHE486 | LEU79 |
|  | PHE486 | TYR83 |
| Hydrogen bonds | GLY502 | LYS353 |
|  | THR500 | ASN330 |
|  | GLY496 | LYS353 |
|  | THR500 | ASP355 |
|  | ASN487 | GLN24 |
|  | GLN493 | LYS31 |
|  | GLN493 | GLU35 |
|  | TYR489 | TYR83 |
|  | GLN498 | LYS353 |
|  | TYR505 | ARG393 |
|  | LYS417 | ASP30 |
|  | TYR449 | ASP38 |
|  | ASN487 | TYR83 |
|  | GLN498 | ASP38 |
|  | ASN501 | HIS41 |
|  | TYR505 | GLU37 |
| Ionic | LYS417 | ASP30 |
|  | GLU484 | LYS31 |
|  | ARG403 | GLU37 |
| Aromatic-Aromatic | PHE486 | TYR83 |

**Supplementary** **table 14**. Quantum biochemistry between SARS-CoV-2 RBD e *Sapajus apella* ACE2.

| **Amino acid residues** | | **Distance** | **Interaction energy** | **Residue charge** | |  | **Residue atom** | |
| --- | --- | --- | --- | --- | --- | --- | --- | --- |
| SARS-CoV-2  RBD | *Sapajus apella* ACE2 | (Å) | (kcal mol-1) | SARS-CoV-2  RBD | *Sapajus apella* ACE2 |  | SARS-CoV-2  RBD | *Sapajus apella* ACE2 |
| LYS417 | ASP30 | 1.66 | -12.24 | 1 | -1 |  | HZ1 | OD2 |
| GLN493 | GLU35 | 1.77 | -4.58 | 0 | -1 |  | HE22 | OE2 |
| ASN487 | TYR83 | 1.80 | -5.92 | 0 | 0 |  | OD1 | HH |
| TYR505 | GLU37 | 1.84 | -9.03 | 0 | -1 |  | HH | OE1 |
| TYR449 | ASP38 | 1.93 | -9.44 | 0 | -1 |  | HH | OD2 |
| GLY502 | LYS353 | 1.98 | -3.95 | 0 | 1 |  | H | O |
| GLN493 | LYS31 | 1.99 | -1.55 | 0 | 1 |  | HE22 | HZ2 |
| GLN498 | ASP38 | 2.02 | -3.40 | 0 | -1 |  | HE22 | OD1 |
| GLY502 | GLN354 | 2.04 | -5.70 | 0 | 0 |  | HA2 | HB1 |
| PHE456 | LYS31 | 2.12 | -2.72 | 0 | 1 |  | HZ | HB1 |
| PHE456 | THR27 | 2.14 | -2.13 | 0 | 0 |  | HE1 | HA |
| THR500 | LEU45 | 2.15 | -0.37 | 0 | 0 |  | HG1 | HD21 |
| PHE486 | LEU79 | 2.16 | -2.67 | 0 | 0 |  | HD1 | HD23 |
| TYR473 | THR27 | 2.25 | -1.76 | 0 | 0 |  | HE2 | HG21 |
| TYR505 | LYS353 | 2.29 | -8.15 | 0 | 1 |  | HB2 | HB2 |
| TYR453 | HIS34 | 2.29 | -1.41 | 0 | 0 |  | HH | CD2 |
| LEU455 | LYS31 | 2.30 | -2.00 | 0 | 1 |  | HD22 | HA |
| GLN498 | LYS353 | 2.32 | -0.22 | 0 | 1 |  | HE22 | HZ1 |
| GLY496 | LYS353 | 2.32 | -2.85 | 0 | 1 |  | O | HZ2 |
| TYR489 | PHE28 | 2.33 | -3.26 | 0 | 0 |  | HH | HB1 |
| GLY504 | GLN354 | 2.34 | -1.82 | 0 | 0 |  | HA2 | HG2 |
| PHE456 | ASP30 | 2.36 | -1.78 | 0 | -1 |  | HE1 | HB2 |
| THR500 | ASN330 | 2.37 | -3.89 | 0 | 0 |  | O | HD22 |
| TYR505 | ARG393 | 2.40 | 1.18 | 0 | 1 |  | HH | HH22 |
| TYR489 | THR27 | 2.40 | -2.20 | 0 | 0 |  | HE2 | OG1 |
| GLN498 | LEU45 | 2.42 | -1.71 | 0 | 0 |  | HG1 | HD11 |
| ASN501 | LYS353 | 2.43 | -8.73 | 0 | 1 |  | HD22 | HD1 |
| PHE486 | TYR83 | 2.46 | -3.99 | 0 | 0 |  | HE1 | HE1 |
| ASN487 | GLN24 | 2.47 | -2.89 | 0 | 0 |  | HD22 | OE1 |
| ASN501 | HIS41 | 2.48 | -0.81 | 0 | 0 |  | HD21 | NE2 |
| TYR505 | GLN354 | 2.49 | -2.96 | 0 | 0 |  | HD1 | HG2 |
| LEU455 | ASP30 | 2.50 | -2.09 | 0 | -1 |  | HD22 | O |
| THR500 | ARG357 | 2.52 | -0.18 | 0 | 1 |  | HG1 | HH11 |
| ASN501 | ASP355 | 2.52 | -1.54 | 0 | -1 |  | HA | HB1 |
| ALA475 | GLN24 | 2.53 | -2.19 | 0 | 0 |  | O | HG1 |
| LEU455 | HIS34 | 2.63 | -3.55 | 0 | 0 |  | HD23 | HB1 |
| ALA475 | THR27 | 2.63 | -1.23 | 0 | 0 |  | HB2 | OG1 |
| GLY476 | GLN24 | 2.67 | -1.90 | 0 | 0 |  | HA1 | HE21 |
| TYR489 | TYR83 | 2.69 | -0.78 | 0 | 0 |  | HH | OH |
| GLY502 | ASP355 | 2.75 | -2.41 | 0 | -1 |  | H | HB1 |
| VAL503 | GLN325 | 2.75 | -1.76 | 0 | 0 |  | HG21 | HB1 |
| GLN493 | HIS34 | 2.76 | -1.72 | 0 | 0 |  | HG1 | HB2 |
| THR500 | ASP355 | 2.78 | -1.64 | 0 | -1 |  | HG1 | OD2 |
| THR500 | HIS41 | 2.94 | 0.61 | 0 | 0 |  | HG1 | HE2 |
| TYR489 | LYS31 | 3.08 | -4.38 | 0 | 1 |  | HB2 | HD2 |
| GLY496 | ASP38 | 3.23 | 0.08 | 0 | -1 |  | HA2 | OD1 |
| LYS417 | HIS34 | 3.25 | -0.73 | 1 | 0 |  | HE2 | HE1 |
| GLN498 | HIS41 | 3.45 | -0.87 | 0 | 0 |  | OE1 | HD2 |
| VAL503 | THR324 | 3.48 | -0.73 | 0 | 0 |  | HG23 | HB |
| PHE486 | THR82 | 3.51 | -2.00 | 0 | 0 |  | HZ | OG1 |
| VAL503 | GLY326 | 3.57 | -0.20 | 0 | 0 |  | HG23 | H |
| GLU484 | LYS31 | 3.60 | -4.32 | -1 | 1 |  | OE1 | HE1 |
| ASP405 | GLN354 | 3.64 | -0.61 | -1 | 0 |  | OD2 | HG2 |
| SER477 | GLN24 | 3.71 | -0.20 | 0 | 0 |  | H | HE22 |
| TYR449 | GLU42 | 3.76 | -0.21 | 0 | -1 |  | HH | HG2 |
| PHE490 | LYS31 | 3.87 | -1.61 | 0 | 1 |  | O | HZ3 |
| GLN498 | GLU42 | 3.88 | -1.10 | 0 | -1 |  | HE21 | HG1 |
| VAL503 | GLN354 | 3.97 | -2.29 | 0 | 0 |  | N | HB1 |
| TYR489 | GLN24 | 4.03 | -0.34 | 0 | 0 |  | HE2 | O |
| PHE497 | LYS353 | 4.04 | -1.49 | 0 | 1 |  | HA | HZ2 |
| TYR449 | LYS353 | 4.11 | -0.16 | 0 | 1 |  | HH | HZ1 |
| ASN487 | LEU79 | 4.13 | -0.38 | 0 | 0 |  | H | HD23 |
| TYR489 | LEU79 | 4.15 | -0.43 | 0 | 0 |  | HE1 | HD23 |
| ASN501 | LEU45 | 4.15 | -0.43 | 0 | 0 |  | HD22 | HD11 |
| GLY502 | GLY326 | 4.23 | 0.45 | 0 | 0 |  | HA1 | HA2 |
| GLN506 | GLY326 | 4.28 | 0.00 | 0 | 0 |  | HE22 | HA2 |
| ARG403 | GLN354 | 4.33 | -0.05 | 1 | 0 |  | HH21 | HG2 |
| ASN501 | ASN330 | 4.35 | -1.24 | 0 | 0 |  | N | HD22 |
| ALA475 | TYR83 | 4.38 | -0.19 | 0 | 0 |  | HB1 | HH |
| PHE456 | LYS26 | 4.40 | -0.05 | 0 | 1 |  | HE1 | O |
| GLN506 | GLN325 | 4.43 | 0.18 | 0 | 0 |  | HE22 | HB1 |
| GLY502 | GLY352 | 4.52 | -0.17 | 0 | 0 |  | H | O |
| THR500 | LEU351 | 4.55 | -0.33 | 0 | 0 |  | HG1 | HD22 |
| GLY502 | PHE356 | 4.55 | -0.17 | 0 | 0 |  | HA2 | H |
| PHE456 | PHE28 | 4.56 | -0.18 | 0 | 0 |  | HZ | HA |
| PRO499 | ASN330 | 4.58 | -0.10 | 0 | 0 |  | O | HD22 |
| GLY502 | HIS41 | 4.58 | -0.15 | 0 | 0 |  | H | HE1 |
| THR500 | GLY326 | 4.59 | -0.56 | 0 | 0 |  | O | HA2 |
| GLY446 | GLU42 | 4.60 | 0.37 | 0 | -1 |  | O | OE2 |
| TYR495 | LYS353 | 4.61 | 1.06 | 0 | 1 |  | HA | HZ3 |
| GLY485 | LEU79 | 4.63 | -0.29 | 0 | 0 |  | C | HD22 |
| PHE486 | PHE28 | 4.64 | -0.62 | 0 | 0 |  | HE1 | HB1 |
| ASN501 | GLY326 | 4.69 | -0.51 | 0 | 0 |  | O | HA2 |
| ASN501 | GLY352 | 4.70 | -0.31 | 0 | 0 |  | HD21 | O |
| ARG403 | HIS34 | 4.70 | -0.58 | 1 | 0 |  | HH11 | HD2 |
| ASN487 | PHE28 | 4.70 | -0.47 | 0 | 0 |  | OD1 | HB1 |
| PHE456 | PHE32 | 4.70 | -0.27 | 0 | 0 |  | HZ | H |
| ASN501 | ARG357 | 4.80 | -0.44 | 0 | 1 |  | N | HH11 |
| GLY446 | LEU45 | 4.83 | -0.11 | 0 | 0 |  | H | HD13 |
| ASN501 | GLN354 | 4.84 | -2.71 | 0 | 0 |  | C | HB1 |
| ALA475 | GLU23 | 4.85 | -0.42 | 0 | -1 |  | HB2 | O |
| TYR505 | ALA386 | 4.93 | -0.39 | 0 | 0 |  | HE1 | O |
| ASN487 | THR27 | 4.96 | -0.40 | 0 | 0 |  | HA | OG1 |
| PHE486 | GLN24 | 4.97 | -0.52 | 0 | 0 |  | HZ | OE1 |
| TYR505 | HIS34 | 4.99 | -0.19 | 0 | 0 |  | HE2 | HD2 |
| GLY502 | THR324 | 4.99 | 0.11 | 0 | 0 |  | HA1 | HB |
| LYS417 | THR27 | 5.02 | -0.27 | 1 | 0 |  | HZ1 | HA |
| LEU455 | GLU35 | 5.04 | -0.39 | 0 | -1 |  | HD23 | HG1 |
| PHE486 | ALA80 | 5.07 | -0.12 | 0 | 0 |  | HE1 | HA |
| PHE456 | HIS34 | 5.14 | -0.45 | 0 | 0 |  | HZ | HD1 |
| GLY504 | LYS353 | 5.17 | -0.31 | 0 | 1 |  | H | O |
| TYR495 | HIS34 | 5.23 | -0.33 | 0 | 0 |  | HE1 | HD2 |
| VAL503 | LYS353 | 5.26 | 0.19 | 0 | 1 |  | N | O |
| ASN501 | ASP38 | 5.26 | -0.23 | 0 | -1 |  | HD22 | OD1 |
| ALA475 | PHE28 | 5.26 | -0.16 | 0 | 0 |  | HB2 | H |
| GLN506 | ASN330 | 5.28 | 0.04 | 0 | 0 |  | HE21 | HD22 |
| LEU455 | PHE32 | 5.30 | -0.27 | 0 | 0 |  | HD22 | N |
| ARG408 | ALA387 | 5.30 | -0.08 | 1 | 0 |  | HH12 | HB2 |
| PHE456 | LEU29 | 5.30 | -0.15 | 0 | 0 |  | HZ | C |
| LEU455 | ASN33 | 5.30 | -0.10 | 0 | 0 |  | HD13 | HB1 |
| TYR421 | ASP30 | 5.30 | -0.45 | 0 | -1 |  | HE2 | OD2 |
| ARG403 | ALA387 | 5.32 | -0.14 | 1 | 0 |  | HH22 | HA |
| TYR489 | ALA25 | 5.32 | -0.11 | 0 | 0 |  | HH | HA |
| TYR473 | GLN24 | 5.36 | -0.14 | 0 | 0 |  | HE2 | HA |
| GLY502 | ASN330 | 5.37 | -0.11 | 0 | 0 |  | HA1 | HD21 |
| GLN506 | GLN354 | 5.40 | -0.31 | 0 | 0 |  | H | HB1 |
| LEU455 | THR27 | 5.40 | -0.39 | 0 | 0 |  | HD22 | O |
| TYR489 | LEU29 | 5.40 | -0.30 | 0 | 0 |  | HE2 | H |
| TYR453 | ASP30 | 5.42 | -0.27 | 0 | -1 |  | HH | O |
| TYR489 | ASP30 | 5.48 | -0.33 | 0 | -1 |  | HE2 | HB1 |
| GLY476 | TYR83 | 5.50 | -0.08 | 0 | 0 |  | HA2 | HE2 |
| LEU492 | LYS31 | 5.51 | -0.37 | 0 | 1 |  | O | HZ3 |
| ARG403 | GLU37 | 5.53 | -1.90 | 1 | -1 |  | HH12 | OE1 |
| THR500 | GLU329 | 5.55 | -0.18 | 0 | -1 |  | HG22 | O |
| TYR505 | PHE390 | 5.57 | -0.12 | 0 | 0 |  | HH | HD1 |
| PHE486 | PRO84 | 5.57 | -0.15 | 0 | 0 |  | HZ | HD1 |
| ARG403 | ARG393 | 5.58 | 1.47 | 1 | 1 |  | HH12 | HH21 |
| TYR489 | LYS26 | 5.59 | 0.04 | 0 | 1 |  | HE2 | C |
| GLY447 | ASP38 | 5.63 | -0.15 | 0 | -1 |  | HA2 | OD1 |
| GLN506 | LYS353 | 5.63 | -0.59 | 0 | 1 |  | N | HB2 |
| VAL445 | ASN49 | 5.64 | -0.07 | 0 | 0 |  | HG11 | HD22 |
| ARG403 | LYS353 | 5.64 | 1.05 | 1 | 1 |  | HD1 | HD2 |
| LYS417 | LYS26 | 5.64 | 0.79 | 1 | 1 |  | HZ1 | O |
| ASN487 | VAL21 | 5.65 | -0.10 | 0 | 0 |  | HD22 | HG23 |
| SER494 | HIS34 | 5.65 | -0.06 | 0 | 0 |  | O | O |
| TYR449 | HIS41 | 5.67 | -0.08 | 0 | 0 |  | HH | HB2 |
| GLY504 | THR324 | 5.67 | -0.04 | 0 | 0 |  | H | HG23 |
| CYS488 | LYS31 | 5.70 | -0.01 | 0 | 1 |  | O | HE1 |
| PRO499 | LEU45 | 5.70 | -0.20 | 0 | 0 |  | HD1 | HD11 |
| TYR505 | ALA387 | 5.70 | -0.17 | 0 | 0 |  | HE1 | HA |
| SER494 | ASP38 | 5.71 | -0.34 | 0 | -1 |  | O | HB1 |
| VAL503 | ASP355 | 5.71 | -0.21 | 0 | -1 |  | H | HA |
| VAL503 | PHE327 | 5.73 | -0.04 | 0 | 0 |  | HG23 | H |
| TYR495 | ASP38 | 5.74 | -0.73 | 0 | -1 |  | HA | HB1 |
| TYR421 | THR27 | 5.76 | -0.04 | 0 | 0 |  | HH | HG22 |
| VAL445 | LEU45 | 5.77 | -0.11 | 0 | 0 |  | HA | HD13 |
| ASN501 | LEU351 | 5.79 | -0.17 | 0 | 0 |  | HD21 | HB1 |
| GLY447 | GLU42 | 5.79 | 0.43 | 0 | -1 |  | HA2 | HG1 |
| PHE497 | ASP38 | 5.80 | -0.15 | 0 | -1 |  | N | OD1 |
| GLY502 | PHE327 | 5.80 | -0.01 | 0 | 0 |  | HA1 | H |
| TYR505 | ASP355 | 5.82 | -0.32 | 0 | -1 |  | HB1 | HB1 |
| GLY476 | THR20 | 5.83 | 0.08 | 0 | 1 |  | HA1 | H2 |
| ASN439 | GLU329 | 5.84 | -0.51 | 0 | -1 |  | HD21 | OE1 |
| THR500 | TRP48 | 5.86 | -0.16 | 0 | 0 |  | HG21 | HD1 |
| CYS488 | LEU79 | 5.86 | -0.07 | 0 | 0 |  | H | HD23 |
| GLY504 | PHE356 | 5.86 | -0.04 | 0 | 0 |  | H | HE2 |
| PRO491 | LYS31 | 5.86 | -0.56 | 0 | 1 |  | HA | HD2 |
| TYR473 | GLU23 | 5.87 | -0.18 | 0 | -1 |  | HE2 | O |
| GLN474 | THR27 | 5.87 | -0.33 | 0 | 0 |  | O | HG21 |
| TYR505 | GLY352 | 5.87 | -0.31 | 0 | 0 |  | HH | HA1 |
| ALA475 | ALA25 | 5.89 | -0.05 | 0 | 0 |  | HB2 | N |
| ASN487 | ALA25 | 5.89 | -0.08 | 0 | 0 |  | OD1 | N |
| GLN493 | ASP30 | 5.90 | -0.04 | 0 | -1 |  | OE1 | O |
| PHE486 | VAL21 | 5.93 | -0.10 | 0 | 0 |  | HZ | HG11 |
| ARG403 | ALA386 | 5.94 | -0.39 | 1 | 0 |  | HH22 | O |
| GLN498 | ASP355 | 5.94 | 0.07 | 0 | -1 |  | HB1 | OD2 |
| PHE486 | ALA25 | 5.96 | -0.08 | 0 | 0 |  | HE1 | HA |
| GLY447 | LYS353 | 5.99 | 0.13 | 0 | 1 |  | HA1 | HZ2 |
| GLN493 | ASP38 | 6.00 | -0.40 | 0 | -1 |  | HE21 | HB2 |
| GLY476 | THR27 | 6.00 | -0.06 | 0 | 0 |  | N | HG21 |
| GLN506 | GLU329 | 6.02 | -0.34 | 0 | -1 |  | HE22 | OE1 |
| THR500 | LYS353 | 6.04 | 0.03 | 0 | 1 |  | O | O |
| ASP405 | ALA387 | 6.06 | -0.11 | -1 | 0 |  | OD1 | HB3 |
| ASN437 | GLN325 | 6.07 | -0.13 | 0 | 0 |  | HD22 | OE1 |
| TYR449 | LEU39 | 6.08 | -0.33 | 0 | 0 |  | HH | N |
| PHE456 | GLU35 | 6.09 | -0.01 | 0 | -1 |  | HE2 | HG1 |
| ASN439 | ASN330 | 6.14 | -0.03 | 0 | 0 |  | HD22 | HD22 |
| SER494 | LYS353 | 6.14 | -0.01 | 0 | 1 |  | O | HZ3 |
| GLY502 | GLN325 | 6.16 | 0.94 | 0 | 0 |  | HA1 | HB1 |
| TYR505 | ASN33 | 6.17 | -0.17 | 0 | 0 |  | HH | O |
| TYR489 | PHE32 | 6.17 | -0.20 | 0 | 0 |  | CE2 | H |
| GLY496 | HIS41 | 6.17 | -0.08 | 0 | 0 |  | O | HB2 |
| LEU455 | LEU29 | 6.17 | -0.09 | 0 | 0 |  | HD22 | O |
| TYR449 | LYS68 | 6.18 | -0.17 | 0 | 1 |  | OH | HZ1 |
| TYR508 | GLN325 | 6.18 | -0.15 | 0 | 0 |  | HH | OE1 |
| THR500 | ASN49 | 6.19 | -0.09 | 0 | 0 |  | HG21 | HD22 |
| THR478 | GLN24 | 6.21 | -0.15 | 0 | 0 |  | H | HE22 |
| CYS488 | TYR83 | 6.22 | -0.11 | 0 | 0 |  | H | HH |
| THR500 | GLN354 | 6.23 | -0.25 | 0 | 0 |  | O | O |
| GLN498 | ARG357 | 6.23 | -0.16 | 0 | 1 |  | HB2 | HH12 |
| GLN506 | ASP355 | 6.23 | -0.23 | 0 | -1 |  | HE21 | HB1 |
| ARG408 | GLN354 | 6.24 | -0.11 | 1 | 0 |  | HH22 | HE22 |
| PRO499 | GLY326 | 6.26 | -0.08 | 0 | 0 |  | O | HA2 |
| GLN493 | PHE72 | 6.31 | -0.03 | 0 | 0 |  | HE22 | HE1 |
| VAL503 | PHE356 | 6.32 | -0.06 | 0 | 0 |  | H | HD2 |
| GLU406 | HIS34 | 6.33 | 0.18 | -1 | 0 |  | OE2 | NE2 |
| GLY502 | LEU351 | 6.35 | -0.02 | 0 | 0 |  | H | HB1 |
| GLY485 | LYS31 | 6.36 | -0.22 | 0 | 1 |  | O | HG1 |
| ALA475 | THR20 | 6.36 | -0.26 | 0 | 1 |  | O | O |
| TYR453 | ASN33 | 6.38 | 0.07 | 0 | 0 |  | HH | HB2 |
| GLY476 | VAL21 | 6.38 | -0.04 | 0 | 0 |  | HA1 | HA |
| TYR505 | PHE356 | 6.39 | -0.12 | 0 | 0 |  | HE1 | HZ |
| GLU484 | GLU35 | 6.39 | 1.67 | -1 | -1 |  | OE1 | OE2 |
| GLY447 | LEU45 | 6.40 | -0.06 | 0 | 0 |  | HA1 | HD11 |
| SER494 | GLU35 | 6.41 | -0.22 | 0 | -1 |  | H | OE2 |
| GLN493 | LEU39 | 6.42 | -0.07 | 0 | 0 |  | HE22 | HD13 |
| TYR505 | HIS41 | 6.42 | -0.20 | 0 | 0 |  | HB2 | ND1 |
| GLY496 | GLU42 | 6.43 | -0.08 | 0 | -1 |  | HA2 | HG2 |
| ARG457 | THR27 | 6.43 | -0.09 | 1 | 0 |  | H | HG22 |
| GLY502 | ARG357 | 6.43 | -0.13 | 0 | 1 |  | H | HH11 |
| THR500 | PHE356 | 6.44 | 0.00 | 0 | 0 |  | O | H |
| PHE456 | ASN33 | 6.44 | -0.09 | 0 | 0 |  | HZ | H |
| TYR505 | PRO389 | 6.44 | -0.04 | 0 | 0 |  | HH | HA |
| GLN506 | THR324 | 6.48 | -0.03 | 0 | 0 |  | HE22 | HB |
| PHE456 | GLN24 | 6.48 | -0.06 | 0 | 0 |  | HE1 | O |
| TYR453 | GLU35 | 6.50 | -0.05 | 0 | -1 |  | OH | N |
| PRO499 | GLU329 | 6.51 | 0.29 | 0 | -1 |  | O | OE1 |
| SER477 | THR20 | 6.52 | 0.12 | 0 | 1 |  | HB2 | H2 |
| SER494 | LYS31 | 6.53 | -0.15 | 0 | 1 |  | H | HZ2 |
| PHE490 | GLU35 | 6.53 | 0.00 | 0 | -1 |  | HB2 | OE2 |
| PHE486 | GLN81 | 6.53 | -0.19 | 0 | 0 |  | HZ | C |
| TYR505 | ASP38 | 6.54 | -0.09 | 0 | -1 |  | HD2 | HB1 |
| LYS417 | ASN33 | 6.54 | 0.11 | 1 | 0 |  | HE2 | HB1 |
| TYR489 | GLU23 | 6.54 | -0.06 | 0 | -1 |  | HE2 | O |
| TYR473 | LYS31 | 6.56 | -0.18 | 0 | 1 |  | HD2 | HB1 |
| GLN498 | LEU39 | 6.56 | -0.13 | 0 | 0 |  | HE22 | N |
| ASN501 | PHE356 | 6.58 | -0.13 | 0 | 0 |  | C | H |
| GLN474 | GLN24 | 6.58 | -0.09 | 0 | 0 |  | C | HG1 |
| LEU455 | PHE28 | 6.58 | -0.13 | 0 | 0 |  | HD22 | O |
| TYR453 | LYS31 | 6.58 | -0.20 | 0 | 1 |  | HH | HA |
| THR500 | PHE327 | 6.59 | -0.08 | 0 | 0 |  | O | N |
| GLY476 | GLU23 | 6.61 | -0.01 | 0 | -1 |  | HA1 | HB1 |
| PHE486 | LYS78 | 6.61 | -0.08 | 0 | 1 |  | HE1 | O |
| LYS417 | LYS31 | 6.61 | 0.57 | 1 | 1 |  | HZ1 | N |
| TYR505 | GLN388 | 6.63 | 0.00 | 0 | 0 |  | OH | O |
| ALA475 | LYS26 | 6.64 | -0.24 | 0 | 1 |  | HB2 | C |
| ASP405 | LYS353 | 6.66 | -1.01 | -1 | 1 |  | OD2 | HA |
| PRO499 | ARG357 | 6.68 | 0.42 | 0 | 1 |  | C | HH11 |
| PRO499 | ASP355 | 6.68 | -0.35 | 0 | -1 |  | C | OD2 |
| ASN501 | GLU42 | 6.70 | -0.13 | 0 | -1 |  | HD22 | H |
| TYR449 | LEU45 | 6.70 | -0.05 | 0 | 0 |  | HH | HD12 |
| ASP405 | ALA386 | 6.71 | 0.11 | -1 | 0 |  | OD2 | HB1 |
| GLY446 | ASP38 | 6.72 | -0.01 | 0 | -1 |  | O | OD2 |
| GLN493 | ALA36 | 6.72 | -0.08 | 0 | 0 |  | HE22 | H |
| GLN498 | GLU37 | 6.74 | 0.12 | 0 | -1 |  | HE22 | O |
| GLY485 | PHE28 | 6.75 | -0.04 | 0 | 0 |  | O | HD1 |
| ALA475 | VAL21 | 6.75 | -0.01 | 0 | 0 |  | O | HA |
| ALA475 | LYS31 | 6.77 | 0.04 | 0 | 1 |  | HB3 | HB1 |
| GLY496 | GLU37 | 6.79 | -0.01 | 0 | -1 |  | H | HG1 |
| GLN493 | ASN33 | 6.79 | 0.00 | 0 | 0 |  | HG1 | C |
| GLY504 | ASP355 | 6.79 | -0.15 | 0 | -1 |  | H | N |
| TYR489 | GLN76 | 6.81 | -0.07 | 0 | 0 |  | HE1 | OE1 |
| ASN487 | LYS31 | 6.81 | -0.05 | 0 | 1 |  | H | HG1 |
| ASN448 | LYS353 | 6.82 | -0.09 | 0 | 1 |  | H | HZ2 |
| PRO499 | HIS41 | 6.82 | 0.01 | 0 | 0 |  | C | HE2 |
| ASN501 | GLU37 | 6.83 | -0.35 | 0 | -1 |  | HD22 | HG2 |
| GLN498 | ASN330 | 6.87 | -0.12 | 0 | 0 |  | O | HD22 |
| GLN506 | PHE327 | 6.87 | -0.03 | 0 | 0 |  | HE22 | H |
| LEU455 | LYS26 | 6.88 | -0.05 | 0 | 1 |  | HD13 | O |
| GLY496 | HIS34 | 6.89 | -0.04 | 0 | 0 |  | H | O |
| CYS488 | PHE28 | 6.90 | -0.11 | 0 | 0 |  | H | HB1 |
| TYR473 | PHE28 | 6.90 | -0.10 | 0 | 0 |  | HE2 | H |
| THR500 | GLY352 | 6.91 | -0.05 | 0 | 0 |  | O | O |
| GLN493 | PHE32 | 6.93 | -0.11 | 0 | 0 |  | OE1 | N |
| PHE456 | ALA25 | 6.96 | -0.03 | 0 | 0 |  | HE1 | O |
| GLY485 | GLU75 | 6.99 | -0.15 | 0 | -1 |  | HA1 | OE1 |
| PHE486 | LEU97 | 7.00 | -0.03 | 0 | 0 |  | HE1 | HD23 |
| GLY485 | TYR83 | 7.04 | -0.09 | 0 | 0 |  | O | HH |
| ASN439 | GLY326 | 7.04 | -0.01 | 0 | 0 |  | HD21 | HA1 |
| ILE472 | LYS31 | 7.06 | 0.18 | 0 | 1 |  | HG21 | HE1 |
| PHE456 | GLU23 | 7.07 | -0.15 | 0 | -1 |  | HE1 | O |
| CYS488 | THR27 | 7.11 | -0.11 | 0 | 0 |  | N | OG1 |
| TYR449 | GLU35 | 7.12 | -0.15 | 0 | -1 |  | HE1 | HA |
| GLY504 | ALA386 | 7.12 | -0.02 | 0 | 0 |  | HA2 | HB2 |
| TYR489 | GLU35 | 7.14 | -0.11 | 0 | -1 |  | HB2 | HG1 |
| GLY496 | LEU45 | 7.15 | -0.02 | 0 | 0 |  | O | HD11 |
| VAL503 | ASN330 | 7.15 | -0.02 | 0 | 0 |  | H | HD22 |
| LYS417 | LEU29 | 7.16 | 0.47 | 1 | 0 |  | HZ1 | C |
| LYS444 | LEU45 | 7.17 | -0.02 | 1 | 0 |  | O | HD13 |
| GLY446 | HIS41 | 7.18 | -0.09 | 0 | 0 |  | H | HD2 |
| GLN493 | GLN76 | 7.18 | 0.01 | 0 | 0 |  | HE22 | HE22 |
| PHE497 | LEU45 | 7.21 | 0.00 | 0 | 0 |  | C | HD11 |
| ASN501 | GLN325 | 7.21 | -0.32 | 0 | 0 |  | O | HB1 |
| PHE486 | GLN76 | 7.21 | -0.12 | 0 | 0 |  | HE1 | O |
| TYR495 | GLU37 | 7.22 | -0.14 | 0 | -1 |  | HD1 | HG1 |
| ASN501 | PHE327 | 7.24 | -0.03 | 0 | 0 |  | O | H |
| TYR473 | LYS26 | 7.27 | 0.02 | 0 | 1 |  | HE2 | C |
| LEU492 | GLU35 | 7.27 | -0.09 | 0 | -1 |  | O | OE2 |
| ASN501 | THR324 | 7.28 | -0.06 | 0 | 0 |  | O | HB |
| GLY447 | HIS41 | 7.30 | -0.06 | 0 | 0 |  | HA1 | HD2 |
| ASN487 | PRO84 | 7.31 | 0.01 | 0 | 0 |  | HD22 | HD1 |
| ASN487 | GLU23 | 7.32 | -0.07 | 0 | -1 |  | OD1 | C |
| ARG403 | PRO389 | 7.32 | -0.03 | 1 | 0 |  | HH12 | HA |
| GLN493 | GLU37 | 7.32 | -0.05 | 0 | -1 |  | HG1 | HB2 |
| GLY404 | GLN354 | 7.34 | -0.08 | 0 | 0 |  | C | HG2 |
| TYR449 | HIS34 | 7.36 | -0.07 | 0 | 0 |  | HE1 | O |
| TYR473 | ASP30 | 7.37 | -0.03 | 0 | -1 |  | HE2 | HB1 |
| PHE486 | LYS31 | 7.38 | -0.05 | 0 | 1 |  | HA | HG1 |
| ASN448 | ASP38 | 7.39 | -0.04 | 0 | -1 |  | H | OD1 |
| SER477 | TYR83 | 7.41 | -0.01 | 0 | 0 |  | H | HE2 |
| ARG403 | ASN33 | 7.43 | -0.14 | 1 | 0 |  | HH12 | HB2 |
| GLY504 | GLY326 | 7.46 | 0.01 | 0 | 0 |  | H | H |
| ASN501 | SER44 | 7.46 | 0.00 | 0 | 0 |  | HD21 | HB1 |
| GLY446 | ASN49 | 7.47 | -0.01 | 0 | 0 |  | HA1 | HD22 |
| TYR453 | ASP38 | 7.48 | 0.06 | 0 | -1 |  | HE1 | HB1 |
| THR500 | SER331 | 7.51 | -0.07 | 0 | 0 |  | HB | N |
| ALA475 | ASP30 | 7.52 | -0.01 | 0 | -1 |  | HB3 | HB1 |
| ASN487 | THR82 | 7.53 | -0.11 | 0 | 0 |  | HD22 | O |
| TYR453 | GLU37 | 7.54 | -0.03 | 0 | -1 |  | OH | HB2 |
| PRO499 | LYS353 | 7.56 | -0.09 | 0 | 1 |  | N | HZ2 |
| TYR449 | GLU37 | 7.56 | 0.08 | 0 | -1 |  | HH | C |
| THR500 | SER44 | 7.57 | -0.06 | 0 | 0 |  | HG1 | C |
| GLY504 | GLN325 | 7.57 | 0.00 | 0 | 0 |  | H | HB1 |
| SER477 | VAL21 | 7.57 | -0.04 | 0 | 0 |  | H | HA |
| PHE486 | THR27 | 7.57 | -0.04 | 0 | 0 |  | HE1 | HG1 |
| LYS458 | THR27 | 7.58 | -0.04 | 1 | 0 |  | HA | HG22 |
| PHE486 | GLN101 | 7.59 | 0.00 | 0 | 0 |  | HZ | HE21 |
| PRO491 | ASP30 | 7.60 | 0.12 | 0 | -1 |  | HA | HB2 |
| ASP405 | PHE356 | 7.61 | 0.02 | -1 | 0 |  | OD2 | HE2 |
| GLN498 | GLY352 | 7.63 | -0.02 | 0 | 0 |  | OE1 | HA2 |
| VAL503 | MET323 | 7.63 | 0.05 | 0 | 0 |  | HG23 | O |
| GLY476 | ALA25 | 7.64 | 0.00 | 0 | 0 |  | HA1 | H |
| PHE497 | HIS41 | 7.64 | -0.14 | 0 | 0 |  | C | HD2 |
| VAL503 | TRP328 | 7.65 | -0.02 | 0 | 0 |  | HG23 | H |
| ARG457 | ASP30 | 7.66 | -0.67 | 1 | -1 |  | H | OD2 |
| GLU484 | LEU79 | 7.66 | -0.07 | -1 | 0 |  | C | HD22 |
| GLY446 | LYS68 | 7.66 | -0.14 | 0 | 1 |  | O | HZ3 |
| GLY502 | LEU45 | 7.67 | -0.03 | 0 | 0 |  | H | HD21 |
| ASN487 | THR20 | 7.67 | -0.04 | 0 | 1 |  | HD22 | O |
| VAL503 | MET383 | 7.67 | -0.04 | 0 | 0 |  | HB | HE3 |
| THR500 | GLN325 | 7.67 | -0.27 | 0 | 0 |  | O | C |
| GLY502 | ASP350 | 7.68 | -0.06 | 0 | -1 |  | H | OD1 |
| ASN439 | GLN325 | 7.70 | -0.06 | 0 | 0 |  | HD21 | HB2 |
| GLN498 | ASN49 | 7.71 | 0.01 | 0 | 0 |  | HG1 | HD22 |
| VAL503 | GLU329 | 7.71 | -0.06 | 0 | -1 |  | HG22 | OE1 |
| TYR453 | LYS353 | 7.72 | -0.10 | 0 | 1 |  | HE1 | HZ3 |
| GLY446 | LYS353 | 7.74 | 0.01 | 0 | 1 |  | O | HZ1 |
| GLY496 | LEU39 | 7.74 | 0.01 | 0 | 0 |  | HA2 | N |
| PRO507 | LYS353 | 7.75 | -0.05 | 0 | 1 |  | HD2 | HZ2 |
| LEU455 | ALA36 | 7.77 | -0.01 | 0 | 0 |  | HD22 | H |
| TYR421 | HIS34 | 7.78 | -0.02 | 0 | 0 |  | HE2 | HE1 |
| THR478 | TYR83 | 7.79 | -0.05 | 0 | 0 |  | OG1 | HH |
| PRO491 | THR27 | 7.82 | 0.00 | 0 | 0 |  | HA | HB |
| GLY502 | MET383 | 7.82 | -0.02 | 0 | 0 |  | HA2 | HE3 |
| ASN501 | GLU329 | 7.82 | 0.20 | 0 | -1 |  | O | HB1 |
| GLY502 | GLU37 | 7.82 | 0.01 | 0 | -1 |  | H | OE2 |
| GLN498 | LEU351 | 7.82 | -0.05 | 0 | 0 |  | OE1 | HB2 |
| ALA475 | LEU29 | 7.85 | -0.03 | 0 | 0 |  | HB2 | H |
| GLY502 | ALA386 | 7.86 | 0.00 | 0 | 0 |  | HA2 | HB2 |
| TYR505 | ASP350 | 7.86 | -0.08 | 0 | -1 |  | HE1 | OD1 |
| ARG403 | GLN388 | 7.87 | -0.30 | 1 | 0 |  | HH22 | O |
| TYR489 | ALA80 | 7.88 | -0.03 | 0 | 0 |  | OH | HA |
| GLY504 | MET383 | 7.90 | -0.02 | 0 | 0 |  | H | HE3 |
| GLN506 | PHE356 | 7.91 | -0.06 | 0 | 0 |  | HE21 | H |
| GLN498 | ALA46 | 7.92 | -0.03 | 0 | 0 |  | HG1 | H |
| SER443 | LYS353 | 7.92 | 0.14 | 0 | 1 |  | HB1 | HZ2 |
| PHE486 | GLU75 | 7.93 | -0.08 | 0 | -1 |  | HD1 | O |
| VAL445 | ARG357 | 7.93 | 0.04 | 0 | 1 |  | HG11 | HH12 |
| CYS488 | GLN24 | 7.93 | -0.05 | 0 | 0 |  | N | HB2 |
| SER494 | GLU37 | 7.93 | -0.02 | 0 | -1 |  | O | HB2 |
| PHE490 | HIS34 | 7.96 | -0.10 | 0 | 0 |  | O | HB1 |
| THR500 | GLU42 | 7.96 | -0.05 | 0 | -1 |  | HG1 | HA |
| TYR505 | THR324 | 7.97 | -0.02 | 0 | 0 |  | H | HG23 |
| ALA475 | GLU22 | 7.97 | 0.00 | 0 | -1 |  | HB2 | O |
| GLN498 | PHE40 | 7.98 | -0.15 | 0 | 0 |  | OE1 | C |
| THR500 | THR324 | 7.98 | -0.02 | 0 | 0 |  | O | OG1 |
| ARG454 | HIS34 | 7.99 | 0.07 | 1 | 0 |  | O | NE2 |
| ASP405 | ARG393 | 7.99 | -1.20 | -1 | 1 |  | OD2 | HH21 |
| ASN501 | PHE40 | 8.00 | 0.09 | 0 | 0 |  | HD22 | C |

**Supplementary** **table 15**. Interactions between SARS-CoV-2 RBD e *Chinchilla lanigera* ACE2.

| **Interaction** | **SARS-CoV-2  RBD** | | ***Chinchilla lanigera*  ACE2** |
| --- | --- | --- | --- |
|  | Residue | Residue | |
| Hydrophobic | PHE486 | LEU78 | |
|  | PHE486 | ALA81 | |
|  | PHE486 | TYR82 | |
| Hydrogen bonds | GLY502 | LYS352 | |
|  | GLY446 | GLN41 | |
|  | GLY496 | LYS352 | |
|  | GLY504 | ASP353 | |
|  | TYR505 | ASP353 | |
|  | ASN501 | TYR40 | |
|  | TYR449 | GLN41 | |
|  | GLN498 | GLN41 | |
|  | ASN487 | TYR82 | |
|  | GLN498 | LYS352 | |
|  | LYS417 | ASP29 | |
|  | TYR449 | ASP37 | |
|  | TYR449 | GLN41 | |
|  | TYR453 | GLU33 | |
|  | ANS487 | TYR82 | |
|  | GLN498 | GLN41 | |
|  | THR500 | TYR40 | |
|  | ASN501 | TYR40 | |
|  | TYR505 | GLU36 | |
| Ionic | LYS417 | ASP29 | |
|  | ARG403 | GLU33 | |
|  | GLU484 | LYS34 | |
|  | ARG403 | GLU36 | |
| Aromatic-Aromatic | PHE486 | TYR82 | |
| Cation-Pi | TYR449 | LYS352 | |
|  | TYR489 | LYS34 | |

**Supplementary** **table 16**. Quantum biochemistry between SARS-CoV-2 RBD e *Chinchilla lanigera* ACE2.

| **Amino acid residues** | | **Distance** | **Interaction energy** | **Residue charge** | |  | **Residue atom** | |
| --- | --- | --- | --- | --- | --- | --- | --- | --- |
| SARS-CoV-2  RBD | *Chinchilla lanigera* ACE2 | (Å) | (kcal mol-1) | SARS-CoV-2  RBD | *Chinchilla lanigera* ACE2 |  | SARS-CoV-2  RBD | *Chinchilla lanigera* ACE2 |
| TYR505 | GLU36 | 1.69 | -10.73 | 0 | -1 |  | HH | OE1 |
| TYR453 | GLU33 | 1.77 | -9.92 | 0 | -1 |  | HH | OE1 |
| LYS417 | ASP29 | 1.81 | -11.39 | 1 | -1 |  | HZ1 | OD2 |
| TYR449 | ASP37 | 1.84 | -8.59 | 0 | -1 |  | HH | OD2 |
| GLY502 | ASP353 | 1.92 | -1.35 | 0 | -1 |  | H | HB1 |
| THR500 | TYR40 | 1.93 | -3.48 | 0 | 0 |  | HG1 | OH |
| GLY496 | LYS352 | 2.11 | -3.23 | 0 | 1 |  | O | HZ3 |
| GLY446 | GLN41 | 2.16 | -2.70 | 0 | 0 |  | O | HE22 |
| LEU455 | GLU33 | 2.19 | -2.95 | 0 | -1 |  | HD23 | HG2 |
| PHE486 | LEU78 | 2.25 | -2.48 | 0 | 0 |  | HD1 | HD22 |
| GLN493 | LYS34 | 2.26 | -2.23 | 0 | 1 |  | HE22 | HG1 |
| ASN501 | LYS352 | 2.26 | -7.72 | 0 | 1 |  | HD22 | HD1 |
| TYR505 | ARG392 | 2.28 | 2.65 | 0 | 1 |  | HH | HH22 |
| TYR449 | GLN41 | 2.31 | -1.04 | 0 | 0 |  | OH | HE21 |
| PHE456 | THR26 | 2.31 | -2.58 | 0 | 0 |  | HE1 | HA |
| TYR505 | ASP353 | 2.34 | -4.52 | 0 | -1 |  | HD1 | OD2 |
| GLN498 | GLN41 | 2.38 | 0.24 | 0 | 0 |  | HE22 | HE21 |
| GLY502 | LYS352 | 2.39 | -2.81 | 0 | 1 |  | H | O |
| TYR489 | ASN30 | 2.43 | -2.23 | 0 | 0 |  | CG | HD22 |
| GLN498 | LYS352 | 2.46 | -3.11 | 0 | 1 |  | OE1 | HZ3 |
| ASN487 | TYR82 | 2.47 | -1.58 | 0 | 0 |  | OD1 | HH |
| GLY504 | ASP353 | 2.51 | -4.53 | 0 | -1 |  | H | OD2 |
| PHE456 | ASN30 | 2.51 | -2.47 | 0 | 0 |  | HZ | HD21 |
| THR500 | ASN329 | 2.53 | -2.02 | 0 | 0 |  | HA | HD22 |
| GLY476 | GLN23 | 2.55 | -1.98 | 0 | 0 |  | HA1 | HE21 |
| TYR505 | LYS352 | 2.55 | -7.79 | 0 | 1 |  | HB1 | O |
| GLN498 | TYR40 | 2.60 | -2.50 | 0 | 0 |  | HG1 | HE2 |
| PHE486 | ALA81 | 2.62 | -1.83 | 0 | 0 |  | HE1 | HB3 |
| THR500 | ARG356 | 2.64 | -1.55 | 0 | 1 |  | HB | HH11 |
| PHE456 | ASP29 | 2.69 | -1.85 | 0 | -1 |  | HZ | HB2 |
| ALA475 | GLN23 | 2.74 | -2.21 | 0 | 0 |  | O | HG1 |
| LEU455 | ASP29 | 2.75 | -0.90 | 0 | -1 |  | HD23 | O |
| GLN493 | GLU33 | 2.78 | -2.22 | 0 | -1 |  | HG1 | HB2 |
| THR500 | ASP354 | 2.81 | -2.53 | 0 | -1 |  | HB | OD2 |
| ASN501 | TYR40 | 2.89 | -2.21 | 0 | 0 |  | HD21 | OH |
| ALA475 | THR26 | 2.89 | -1.39 | 0 | 0 |  | HB1 | HG1 |
| TYR489 | PHE27 | 2.94 | -2.58 | 0 | 0 |  | HH | HB1 |
| TYR489 | LYS34 | 3.10 | -0.77 | 0 | 1 |  | HD1 | HZ3 |
| ASN501 | ASP354 | 3.14 | -1.11 | 0 | -1 |  | HA | HB1 |
| LEU455 | ASN30 | 3.28 | -0.86 | 0 | 0 |  | HD23 | HA |
| TYR473 | THR26 | 3.36 | -0.74 | 0 | 0 |  | HE2 | HG23 |
| GLN498 | ASP37 | 3.41 | -1.79 | 0 | -1 |  | HE22 | OD1 |
| TYR489 | THR26 | 3.44 | 0.29 | 0 | 0 |  | HE2 | HG1 |
| TYR449 | LYS352 | 3.46 | 1.24 | 0 | 1 |  | HE1 | HZ2 |
| GLY496 | ASP37 | 3.48 | -0.25 | 0 | -1 |  | HA2 | OD1 |
| SER477 | GLN23 | 3.51 | -0.55 | 0 | 0 |  | H | HE22 |
| TYR489 | TYR82 | 3.56 | -0.41 | 0 | 0 |  | HH | OH |
| THR500 | LEU44 | 3.67 | -0.61 | 0 | 0 |  | HG1 | HD22 |
| GLN498 | LEU44 | 3.70 | -0.78 | 0 | 0 |  | HG1 | HD13 |
| PHE486 | TYR82 | 3.71 | -1.00 | 0 | 0 |  | HE1 | HH |
| ASN501 | ASP353 | 3.74 | -0.67 | 0 | -1 |  | HA | HB1 |
| ASN487 | GLN23 | 3.92 | -0.59 | 0 | 0 |  | OD1 | HB2 |
| ASN487 | LEU78 | 3.95 | -0.39 | 0 | 0 |  | H | HD22 |
| THR500 | GLY325 | 4.07 | -0.65 | 0 | 0 |  | O | HA2 |
| GLU484 | LYS34 | 4.08 | -3.84 | -1 | 1 |  | OE1 | HZ2 |
| GLY502 | THR323 | 4.08 | -0.09 | 0 | 0 |  | HA2 | HG23 |
| TYR489 | GLN23 | 4.09 | -1.08 | 0 | 0 |  | HH | O |
| VAL503 | ASP353 | 4.14 | -4.99 | 0 | -1 |  | N | OD2 |
| GLY446 | LEU44 | 4.18 | -0.20 | 0 | 0 |  | H | HD12 |
| PHE497 | LYS352 | 4.22 | -1.63 | 0 | 1 |  | N | HZ3 |
| GLY447 | GLN41 | 4.22 | -0.76 | 0 | 0 |  | HA2 | HE22 |
| TYR505 | ALA385 | 4.26 | 0.26 | 0 | 0 |  | HE1 | O |
| TYR473 | ASN30 | 4.30 | -0.30 | 0 | 0 |  | HD2 | HD22 |
| THR500 | GLN328 | 4.31 | -0.12 | 0 | 0 |  | HA | OE1 |
| GLY502 | ASP354 | 4.39 | -0.63 | 0 | -1 |  | H | N |
| TYR489 | LEU78 | 4.50 | -0.40 | 0 | 0 |  | HE1 | HD13 |
| ASN501 | GLY351 | 4.51 | -0.29 | 0 | 0 |  | HD21 | O |
| PHE490 | LYS34 | 4.52 | -0.25 | 0 | 1 |  | H | HZ2 |
| GLN506 | GLN324 | 4.52 | -0.37 | 0 | 0 |  | HE22 | HB1 |
| VAL503 | THR323 | 4.55 | -0.30 | 0 | 0 |  | H | HG23 |
| VAL503 | GLN324 | 4.65 | -0.40 | 0 | 0 |  | HG23 | HB1 |
| ASN487 | PHE27 | 4.68 | -0.42 | 0 | 0 |  | OD1 | HB1 |
| LEU455 | LYS34 | 4.70 | -0.25 | 0 | 1 |  | HD22 | HE2 |
| GLY502 | GLY325 | 4.72 | -0.03 | 0 | 0 |  | HA1 | H |
| LYS417 | GLU33 | 4.77 | -2.02 | 1 | -1 |  | HE2 | HG2 |
| GLN506 | ASP353 | 4.78 | -0.44 | 0 | -1 |  | H | OD2 |
| PHE456 | PHE27 | 4.78 | -0.29 | 0 | 0 |  | HZ | N |
| ASN487 | THR26 | 4.79 | -1.10 | 0 | 0 |  | OD1 | HG1 |
| ASP405 | ASP353 | 4.87 | 3.06 | -1 | -1 |  | OD2 | OD2 |
| PHE456 | LYS34 | 4.89 | -0.22 | 0 | 1 |  | HE2 | HE2 |
| TYR489 | GLN75 | 4.91 | -0.06 | 0 | 0 |  | HE1 | HE22 |
| THR500 | ASP353 | 4.91 | 0.00 | 0 | -1 |  | O | O |
| PHE456 | LYS25 | 4.91 | -0.21 | 0 | 1 |  | HE1 | O |
| GLY447 | LYS352 | 4.92 | 0.04 | 0 | 1 |  | HA2 | HZ3 |
| ARG403 | GLU33 | 4.94 | -3.20 | 1 | -1 |  | HH11 | OE1 |
| PRO499 | GLN328 | 4.97 | 0.23 | 0 | 0 |  | O | OE1 |
| ARG403 | GLU36 | 4.98 | -1.96 | 1 | -1 |  | HH12 | OE1 |
| VAL445 | LEU44 | 4.98 | 0.01 | 0 | 0 |  | HG12 | HD12 |
| ARG408 | ILE386 | 5.01 | -0.22 | 1 | 0 |  | HH12 | HG21 |
| GLY485 | LEU78 | 5.03 | -0.12 | 0 | 0 |  | C | HD21 |
| THR500 | LEU350 | 5.03 | -0.21 | 0 | 0 |  | HB | HD21 |
| ARG403 | ILE386 | 5.05 | -0.28 | 1 | 0 |  | HH22 | HA |
| PRO499 | ASN329 | 5.06 | -0.05 | 0 | 0 |  | O | HD22 |
| THR500 | LYS352 | 5.06 | -0.48 | 0 | 1 |  | O | O |
| ASP405 | ILE386 | 5.06 | -0.16 | -1 | 0 |  | OD1 | HG21 |
| TYR495 | LYS352 | 5.07 | 1.04 | 0 | 1 |  | HA | HZ1 |
| LYS417 | THR26 | 5.07 | -0.22 | 1 | 0 |  | HZ2 | HG21 |
| ASN439 | GLN328 | 5.12 | -0.12 | 0 | 0 |  | HD21 | HE22 |
| GLN493 | ASN30 | 5.12 | -0.47 | 0 | 0 |  | HE22 | O |
| THR500 | PHE355 | 5.15 | -0.20 | 0 | 0 |  | O | H |
| TYR505 | ILE386 | 5.15 | -0.15 | 0 | 0 |  | HE1 | HA |
| ASN501 | GLY325 | 5.15 | -0.31 | 0 | 0 |  | O | HA2 |
| PHE456 | GLU33 | 5.18 | -0.29 | 0 | -1 |  | HE2 | HG2 |
| GLN506 | GLY325 | 5.23 | 0.07 | 0 | 0 |  | HE22 | H |
| ALA475 | TYR82 | 5.24 | -0.10 | 0 | 0 |  | HB3 | HH |
| PHE486 | PHE27 | 5.25 | -0.16 | 0 | 0 |  | HE1 | HD1 |
| PHE456 | PHE31 | 5.25 | -0.14 | 0 | 0 |  | HZ | H |
| PHE456 | LEU28 | 5.28 | -0.28 | 0 | 0 |  | HZ | C |
| ALA475 | ASN30 | 5.30 | -0.05 | 0 | 0 |  | HB2 | HD21 |
| ARG403 | ARG392 | 5.35 | 1.53 | 1 | 1 |  | HH12 | HH22 |
| GLN506 | GLN328 | 5.36 | -0.02 | 0 | 0 |  | HE22 | HE22 |
| ARG403 | ASP353 | 5.37 | -2.11 | 1 | -1 |  | HH21 | OD2 |
| GLY502 | GLN324 | 5.42 | 0.26 | 0 | 0 |  | HA1 | HB1 |
| GLN493 | ASP37 | 5.45 | -0.35 | 0 | -1 |  | HE21 | HB1 |
| ALA475 | PHE27 | 5.45 | -0.15 | 0 | 0 |  | HB1 | H |
| ASN487 | ALA81 | 5.46 | -0.16 | 0 | 0 |  | HD22 | HB3 |
| ALA475 | GLU22 | 5.46 | -0.18 | 0 | -1 |  | HB1 | O |
| GLY502 | PHE355 | 5.47 | -0.16 | 0 | 0 |  | HA2 | HE2 |
| PRO499 | TYR40 | 5.51 | -0.52 | 0 | 0 |  | HD1 | HE2 |
| TYR489 | ALA24 | 5.55 | -0.30 | 0 | 0 |  | HH | HA |
| ARG403 | LYS352 | 5.56 | 1.10 | 1 | 1 |  | HD1 | HD2 |
| GLY496 | GLN41 | 5.57 | -0.09 | 0 | 0 |  | HA2 | HE21 |
| PHE490 | ASN30 | 5.60 | -0.24 | 0 | 0 |  | O | OD1 |
| GLN498 | ASP354 | 5.60 | -0.21 | 0 | -1 |  | HB1 | OD2 |
| ARG403 | ALA385 | 5.63 | -0.42 | 1 | 0 |  | HH22 | O |
| LYS417 | LYS25 | 5.64 | 0.96 | 1 | 1 |  | HZ1 | HG1 |
| GLY446 | TYR40 | 5.65 | -0.20 | 0 | 0 |  | H | HE2 |
| ASN501 | ASN329 | 5.65 | -0.22 | 0 | 0 |  | N | HD22 |
| PHE486 | ALA79 | 5.66 | -0.17 | 0 | 0 |  | HE1 | N |
| TYR449 | TYR40 | 5.68 | -0.19 | 0 | 0 |  | OH | HD2 |
| GLN506 | LYS352 | 5.73 | -0.35 | 0 | 1 |  | H | O |
| PRO499 | GLY325 | 5.75 | -0.17 | 0 | 0 |  | O | HA1 |
| TYR505 | GLY351 | 5.75 | -0.44 | 0 | 0 |  | HH | HA1 |
| TYR449 | LEU38 | 5.75 | -0.24 | 0 | 0 |  | HH | N |
| GLY502 | TYR40 | 5.77 | -0.09 | 0 | 0 |  | H | HE1 |
| GLY447 | ASP37 | 5.78 | -0.01 | 0 | -1 |  | HA2 | OD1 |
| VAL503 | GLY325 | 5.80 | -0.04 | 0 | 0 |  | HG23 | H |
| TYR505 | ASP354 | 5.80 | -0.30 | 0 | -1 |  | HB1 | H |
| ASN501 | ARG356 | 5.81 | -0.18 | 0 | 1 |  | N | HH11 |
| GLY502 | GLY351 | 5.83 | 0.01 | 0 | 0 |  | H | O |
| ASN501 | LEU44 | 5.85 | -0.08 | 0 | 0 |  | HD22 | HD22 |
| ASN487 | ASN30 | 5.86 | -0.19 | 0 | 0 |  | HA | HD22 |
| PHE486 | ASN77 | 5.87 | -0.03 | 0 | 0 |  | HE1 | O |
| SER494 | LYS352 | 5.88 | -0.24 | 0 | 1 |  | O | HZ1 |
| GLN498 | ARG356 | 5.89 | 0.01 | 0 | 1 |  | HB2 | HH12 |
| TYR505 | PHE389 | 5.91 | -0.06 | 0 | 0 |  | HH | HD1 |
| TYR421 | ASP29 | 5.91 | 0.05 | 0 | -1 |  | HE2 | OD2 |
| PHE486 | GLN23 | 5.94 | -0.36 | 0 | 0 |  | HZ | OE1 |
| VAL445 | ASN48 | 5.95 | -0.04 | 0 | 0 |  | HG12 | HD22 |
| PRO491 | ASN30 | 5.96 | -0.05 | 0 | 0 |  | HA | HD22 |
| THR478 | GLN23 | 5.97 | -0.12 | 0 | 0 |  | H | HE22 |
| TYR505 | GLU33 | 6.01 | -0.19 | 0 | -1 |  | HE2 | HA |
| TYR489 | LEU28 | 6.03 | -0.20 | 0 | 0 |  | HH | N |
| GLY504 | LYS352 | 6.04 | -0.28 | 0 | 1 |  | H | O |
| GLN493 | ASP29 | 6.04 | -0.14 | 0 | -1 |  | HG1 | O |
| LEU455 | PHE31 | 6.07 | -0.15 | 0 | 0 |  | HD23 | N |
| TYR505 | ASN32 | 6.07 | -0.18 | 0 | 0 |  | HH | O |
| SER494 | GLU33 | 6.07 | 0.14 | 0 | -1 |  | O | HB2 |
| CYS488 | ASN30 | 6.08 | -0.17 | 0 | 0 |  | C | HD22 |
| SER494 | ASP37 | 6.08 | -0.11 | 0 | -1 |  | O | HB1 |
| ASN487 | ALA24 | 6.08 | 0.03 | 0 | 0 |  | OD1 | HA |
| ASN501 | THR323 | 6.10 | -0.14 | 0 | 0 |  | O | HB |
| GLU406 | GLU33 | 6.13 | 2.53 | -1 | -1 |  | OE2 | OE1 |
| THR500 | THR323 | 6.14 | -0.18 | 0 | 0 |  | O | HB |
| GLY476 | TYR82 | 6.14 | -0.04 | 0 | 0 |  | HA2 | HH |
| LEU455 | ASN32 | 6.16 | -0.14 | 0 | 0 |  | HD11 | HB1 |
| GLY446 | LYS352 | 6.16 | -0.10 | 0 | 1 |  | O | HZ3 |
| PRO499 | LEU44 | 6.17 | -0.09 | 0 | 0 |  | HD1 | HD22 |
| THR500 | GLY351 | 6.18 | -0.06 | 0 | 0 |  | O | O |
| ASN501 | PHE355 | 6.22 | -0.26 | 0 | 0 |  | HA | H |
| ASN501 | LEU350 | 6.27 | -0.10 | 0 | 0 |  | HD21 | HB1 |
| GLY446 | ASP37 | 6.27 | 0.14 | 0 | -1 |  | O | OD1 |
| ASN448 | LYS352 | 6.28 | -0.11 | 0 | 1 |  | H | HZ3 |
| TYR473 | GLN23 | 6.28 | -0.08 | 0 | 0 |  | HE2 | HA |
| PHE497 | ASP37 | 6.28 | -0.03 | 0 | -1 |  | N | OD1 |
| GLY447 | LEU44 | 6.28 | -0.07 | 0 | 0 |  | N | HD12 |
| GLY447 | TYR40 | 6.28 | -0.10 | 0 | 0 |  | HA1 | HE2 |
| ALA475 | ALA24 | 6.30 | -0.09 | 0 | 0 |  | HB1 | N |
| VAL503 | LYS352 | 6.30 | 0.07 | 0 | 1 |  | N | O |
| TYR505 | GLN387 | 6.32 | -0.02 | 0 | 0 |  | OH | O |
| ASN501 | GLN324 | 6.33 | -0.36 | 0 | 0 |  | O | HB1 |
| LEU455 | THR26 | 6.33 | -0.28 | 0 | 0 |  | HG | O |
| ASN501 | GLN328 | 6.35 | -0.12 | 0 | 0 |  | O | HE22 |
| VAL445 | GLN41 | 6.35 | -0.05 | 0 | 0 |  | C | HE22 |
| CYS488 | LYS34 | 6.35 | 0.00 | 0 | 1 |  | O | HZ2 |
| TYR495 | ASP37 | 6.37 | -0.21 | 0 | -1 |  | C | OD1 |
| CYS488 | LEU78 | 6.37 | -0.06 | 0 | 0 |  | H | HD22 |
| VAL445 | TYR40 | 6.37 | -0.08 | 0 | 0 |  | HA | HE2 |
| GLN506 | THR323 | 6.39 | -0.01 | 0 | 0 |  | HE21 | HB |
| TYR453 | ASP29 | 6.39 | -0.17 | 0 | -1 |  | HE2 | O |
| TYR453 | LYS34 | 6.39 | -0.22 | 0 | 1 |  | HH | N |
| GLY504 | THR323 | 6.40 | -0.02 | 0 | 0 |  | H | HG23 |
| GLN493 | ALA35 | 6.42 | -0.12 | 0 | 0 |  | HE21 | N |
| TYR421 | THR26 | 6.42 | -0.13 | 0 | 0 |  | OH | HG21 |
| GLY496 | TYR40 | 6.43 | -0.11 | 0 | 0 |  | O | CE2 |
| THR500 | PHE326 | 6.45 | -0.15 | 0 | 0 |  | O | H |
| PRO499 | ARG356 | 6.47 | 0.40 | 0 | 1 |  | HD1 | HH12 |
| GLN493 | PHE71 | 6.48 | -0.02 | 0 | 0 |  | HE22 | HE1 |
| TYR505 | PHE355 | 6.48 | -0.12 | 0 | 0 |  | HD1 | HE2 |
| TYR495 | GLU33 | 6.49 | -0.24 | 0 | -1 |  | HE1 | OE1 |
| TYR449 | LEU44 | 6.49 | -0.05 | 0 | 0 |  | OH | HD13 |
| GLY476 | THR26 | 6.50 | -0.05 | 0 | 0 |  | N | HG1 |
| GLN493 | GLU36 | 6.51 | -0.11 | 0 | -1 |  | HE21 | HB2 |
| GLN498 | GLU36 | 6.52 | 0.12 | 0 | -1 |  | HE22 | O |
| GLU484 | ASN30 | 6.53 | 0.22 | -1 | 0 |  | OE1 | OD1 |
| ALA475 | SER19 | 6.55 | -0.26 | 0 | 1 |  | O | H3 |
| ASN439 | GLN324 | 6.56 | -0.08 | 0 | 0 |  | HD21 | OE1 |
| TYR489 | PHE31 | 6.57 | -0.12 | 0 | 0 |  | HE1 | H |
| TYR453 | ASN32 | 6.60 | 0.20 | 0 | 0 |  | HH | C |
| TYR453 | GLU36 | 6.60 | -0.08 | 0 | -1 |  | HH | HB2 |
| TYR505 | TYR40 | 6.62 | -0.15 | 0 | 0 |  | HB1 | HE1 |
| PHE486 | LYS80 | 6.64 | -0.02 | 0 | 1 |  | HE1 | C |
| ASN501 | ASP37 | 6.64 | -0.07 | 0 | -1 |  | HD22 | OD1 |
| PHE456 | GLU22 | 6.65 | -0.14 | 0 | -1 |  | HE1 | O |
| VAL445 | ARG356 | 6.66 | 0.18 | 0 | 1 |  | HG21 | HH12 |
| GLY476 | SER19 | 6.69 | 0.01 | 0 | 1 |  | HA1 | H2 |
| LEU455 | LEU28 | 6.72 | -0.17 | 0 | 0 |  | HD23 | O |
| GLN493 | LEU38 | 6.74 | -0.03 | 0 | 0 |  | HE22 | HD13 |
| SER494 | LYS34 | 6.75 | -0.14 | 0 | 1 |  | H | HG1 |
| ASN501 | GLU36 | 6.76 | -0.20 | 0 | -1 |  | HD22 | HG2 |
| TYR453 | LYS352 | 6.76 | -0.18 | 0 | 1 |  | HE1 | HZ1 |
| TYR489 | LYS25 | 6.76 | 0.10 | 0 | 1 |  | HH | C |
| LYS417 | ASN32 | 6.77 | 0.21 | 1 | 0 |  | HE2 | HB1 |
| GLY502 | PHE326 | 6.77 | 0.00 | 0 | 0 |  | HA1 | H |
| PRO499 | ASP354 | 6.79 | -0.65 | 0 | -1 |  | C | OD2 |
| TYR473 | GLU22 | 6.80 | -0.10 | 0 | -1 |  | HE2 | O |
| ASP405 | ALA385 | 6.80 | -0.04 | -1 | 0 |  | OD2 | O |
| ARG457 | THR26 | 6.83 | -0.08 | 1 | 0 |  | O | HG23 |
| GLN498 | GLY351 | 6.85 | -0.11 | 0 | 0 |  | OE1 | O |
| PHE486 | PRO83 | 6.86 | -0.06 | 0 | 0 |  | HZ | HD2 |
| GLN498 | LEU38 | 6.89 | -0.18 | 0 | 0 |  | HE22 | N |
| THR500 | TRP47 | 6.89 | -0.06 | 0 | 0 |  | HG22 | HE1 |
| THR500 | GLN324 | 6.89 | -0.26 | 0 | 0 |  | HA | C |
| TYR489 | ASP29 | 6.89 | -0.41 | 0 | -1 |  | HH | H |
| TYR505 | ASP37 | 6.91 | -0.08 | 0 | -1 |  | HE2 | N |
| PHE456 | ASN32 | 6.91 | -0.08 | 0 | 0 |  | HZ | HB1 |
| TYR505 | PRO388 | 6.92 | -0.01 | 0 | 0 |  | HH | HA |
| LYS417 | ASN30 | 6.96 | -0.24 | 1 | 0 |  | HZ1 | N |
| PHE456 | GLN23 | 6.96 | -0.05 | 0 | 0 |  | HE1 | O |
| PHE497 | TYR40 | 6.96 | -0.20 | 0 | 0 |  | C | HE2 |
| GLY485 | LYS34 | 6.97 | -0.26 | 0 | 1 |  | O | HZ3 |
| GLY496 | GLU36 | 6.97 | -0.01 | 0 | -1 |  | H | HG1 |
| GLN474 | THR26 | 7.05 | 0.03 | 0 | 0 |  | C | HG1 |
| GLY502 | GLN328 | 7.06 | -0.01 | 0 | 0 |  | HA1 | HE22 |
| LYS417 | LEU28 | 7.06 | 0.40 | 1 | 0 |  | HZ1 | C |
| GLN474 | GLN23 | 7.07 | -0.04 | 0 | 0 |  | O | HE21 |
| GLY446 | ASN48 | 7.07 | -0.01 | 0 | 0 |  | H | HD22 |
| ASN501 | GLN41 | 7.10 | -0.05 | 0 | 0 |  | HD22 | HE21 |
| LYS444 | GLN41 | 7.10 | -0.05 | 1 | 0 |  | O | HE22 |
| ASN487 | THR20 | 7.13 | -0.03 | 0 | 0 |  | HD22 | HA |
| VAL503 | MET322 | 7.14 | -0.04 | 0 | 0 |  | HG21 | O |
| PHE486 | THR20 | 7.14 | -0.04 | 0 | 0 |  | HZ | HG22 |
| LYS458 | THR26 | 7.15 | -0.03 | 1 | 0 |  | HA | HG23 |
| GLN493 | PHE31 | 7.15 | -0.12 | 0 | 0 |  | HE22 | O |
| TYR473 | PHE27 | 7.16 | -0.07 | 0 | 0 |  | HE2 | N |
| TYR449 | GLU36 | 7.18 | 0.08 | 0 | -1 |  | HH | C |
| GLY404 | ASP353 | 7.18 | -0.29 | 0 | -1 |  | HA2 | OD2 |
| GLY504 | PHE355 | 7.18 | -0.02 | 0 | 0 |  | H | HE2 |
| ASN439 | ASN329 | 7.18 | -0.02 | 0 | 0 |  | HD22 | HD22 |
| ASN448 | GLN41 | 7.19 | -0.01 | 0 | 0 |  | N | HE22 |
| PHE486 | ALA24 | 7.19 | -0.06 | 0 | 0 |  | HE1 | HA |
| GLN493 | ASN32 | 7.19 | 0.01 | 0 | 0 |  | HE21 | C |
| VAL503 | PHE355 | 7.19 | -0.03 | 0 | 0 |  | H | HE2 |
| PHE486 | GLN74 | 7.24 | -0.07 | 0 | 0 |  | HD1 | O |
| GLN506 | ASN329 | 7.24 | 0.00 | 0 | 0 |  | HE21 | HD22 |
| LYS444 | TYR40 | 7.24 | -0.06 | 1 | 0 |  | O | HE2 |
| LEU492 | LYS34 | 7.28 | -0.10 | 0 | 1 |  | O | HE1 |
| ARG403 | GLN387 | 7.29 | -0.29 | 1 | 0 |  | HH22 | O |
| ALA475 | LYS25 | 7.29 | -0.12 | 0 | 1 |  | HB1 | N |
| CYS488 | TYR82 | 7.29 | -0.06 | 0 | 0 |  | H | HH |
| PHE497 | GLN41 | 7.30 | -0.02 | 0 | 0 |  | N | HE21 |
| GLY502 | MET382 | 7.30 | -0.02 | 0 | 0 |  | HA2 | HE3 |
| ASP405 | LYS352 | 7.31 | -0.92 | -1 | 1 |  | OD2 | HA |
| TYR489 | GLU22 | 7.32 | -0.04 | 0 | -1 |  | HH | O |
| GLN498 | LEU350 | 7.33 | -0.08 | 0 | 0 |  | HG1 | HD22 |
| VAL445 | ASN329 | 7.33 | -0.04 | 0 | 0 |  | HG21 | OD1 |
| VAL503 | GLN328 | 7.34 | -0.01 | 0 | 0 |  | HG23 | HE22 |
| PRO499 | GLN324 | 7.34 | -0.25 | 0 | 0 |  | O | HB1 |
| TYR449 | LYS34 | 7.35 | -0.09 | 0 | 1 |  | HH | O |
| CYS488 | PHE27 | 7.36 | -0.06 | 0 | 0 |  | H | HD1 |
| GLY446 | ARG356 | 7.36 | 0.05 | 0 | 1 |  | H | HH12 |
| TYR453 | ASP37 | 7.37 | 0.08 | 0 | -1 |  | OH | HB1 |
| ASN487 | LYS34 | 7.40 | 0.00 | 0 | 1 |  | H | HZ3 |
| ASN437 | GLN324 | 7.41 | -0.02 | 0 | 0 |  | HD22 | OE1 |
| ASN487 | GLN75 | 7.42 | -0.02 | 0 | 0 |  | H | HE22 |
| ASN448 | ASP37 | 7.43 | -0.11 | 0 | -1 |  | O | OD1 |
| ILE418 | GLU33 | 7.44 | -0.12 | 0 | -1 |  | HD1 | OE1 |
| GLN506 | ASP354 | 7.44 | -0.13 | 0 | -1 |  | HE21 | HA |
| ASN439 | GLY325 | 7.46 | -0.01 | 0 | 0 |  | HD21 | H |
| GLY485 | PHE27 | 7.46 | -0.03 | 0 | 0 |  | O | HD1 |
| GLY476 | THR20 | 7.46 | -0.03 | 0 | 0 |  | HA1 | HA |
| TYR495 | GLU36 | 7.47 | -0.10 | 0 | -1 |  | HA | HG1 |
| ARG403 | ASN32 | 7.47 | -0.04 | 1 | 0 |  | HH12 | O |
| PHE486 | GLN75 | 7.48 | -0.13 | 0 | 0 |  | HA | HE22 |
| SER477 | SER19 | 7.49 | 0.14 | 0 | 1 |  | HB2 | H2 |
| TYR453 | ASN30 | 7.50 | -0.02 | 0 | 0 |  | HE2 | HA |
| GLN498 | ASN329 | 7.51 | -0.08 | 0 | 0 |  | HB2 | HD22 |
| GLY502 | ASN329 | 7.54 | -0.01 | 0 | 0 |  | HA1 | HD22 |
| GLY485 | GLN75 | 7.55 | -0.09 | 0 | 0 |  | O | HE22 |
| GLY504 | ILE386 | 7.55 | -0.04 | 0 | 0 |  | HA2 | HG22 |
| ASN501 | PHE326 | 7.55 | -0.03 | 0 | 0 |  | O | H |
| VAL503 | MET382 | 7.57 | -0.05 | 0 | 0 |  | HG21 | HE3 |
| PRO507 | LYS352 | 7.57 | -0.05 | 0 | 1 |  | HD2 | HD2 |
| ALA475 | THR20 | 7.57 | 0.01 | 0 | 0 |  | O | HA |
| VAL445 | LYS60 | 7.58 | 0.01 | 0 | 1 |  | HG11 | HZ3 |
| PRO491 | LYS34 | 7.59 | -0.21 | 0 | 1 |  | HA | HE2 |
| LYS444 | LEU44 | 7.60 | -0.01 | 1 | 0 |  | O | HD12 |
| VAL503 | ASP354 | 7.61 | -0.12 | 0 | -1 |  | H | HA |
| PRO499 | LYS352 | 7.61 | -0.09 | 0 | 1 |  | N | HD1 |
| LEU455 | PHE27 | 7.63 | -0.07 | 0 | 0 |  | HD23 | O |
| TYR473 | ASP29 | 7.63 | 0.01 | 0 | -1 |  | HE2 | HB1 |
| PHE456 | ALA24 | 7.65 | -0.03 | 0 | 0 |  | HE1 | O |
| TYR505 | THR323 | 7.67 | -0.02 | 0 | 0 |  | H | HG23 |
| SER494 | GLU36 | 7.68 | 0.01 | 0 | -1 |  | O | HB2 |
| THR500 | ASN48 | 7.70 | -0.03 | 0 | 0 |  | HG22 | HD22 |
| ASN487 | GLU22 | 7.70 | 0.04 | 0 | -1 |  | OD1 | O |
| TYR449 | GLU33 | 7.70 | -0.04 | 0 | -1 |  | HE1 | O |
| THR500 | SER330 | 7.72 | -0.08 | 0 | 0 |  | HG23 | N |
| CYS488 | THR26 | 7.74 | -0.07 | 0 | 0 |  | N | HG1 |
| ILE472 | ASN30 | 7.76 | -0.03 | 0 | 0 |  | HG21 | HD22 |
| GLN498 | ASP353 | 7.77 | 0.13 | 0 | -1 |  | O | HB1 |
| ARG454 | GLU33 | 7.78 | -0.48 | 1 | -1 |  | C | HG2 |
| TYR508 | ASP353 | 7.80 | 0.08 | 0 | -1 |  | HE1 | OD2 |
| ARG403 | PRO388 | 7.81 | -0.05 | 1 | 0 |  | HH12 | HA |
| GLN498 | SER39 | 7.84 | -0.08 | 0 | 0 |  | HE22 | C |
| GLN493 | LYS352 | 7.85 | 0.22 | 0 | 1 |  | HE21 | HZ1 |
| ILE472 | LYS34 | 7.85 | 0.14 | 0 | 1 |  | HG22 | HZ2 |
| GLY485 | ASN30 | 7.85 | -0.02 | 0 | 0 |  | O | HD22 |
| GLN498 | SER42 | 7.86 | -0.03 | 0 | 0 |  | HE22 | H |
| GLY496 | LEU44 | 7.89 | -0.02 | 0 | 0 |  | O | HD13 |
| ARG408 | ASP353 | 7.89 | -1.33 | 1 | -1 |  | HH22 | OD2 |
| LEU455 | LYS25 | 7.89 | -0.07 | 0 | 1 |  | HD11 | O |
| GLU484 | LEU78 | 7.89 | -0.06 | -1 | 0 |  | HB2 | HD11 |
| GLN474 | ASN30 | 7.90 | 0.00 | 0 | 0 |  | N | HD22 |
| SER477 | TYR82 | 7.91 | -0.01 | 0 | 0 |  | H | HE2 |
| GLY504 | ALA385 | 7.95 | -0.02 | 0 | 0 |  | HA2 | HB3 |
| LEU455 | ALA35 | 7.96 | -0.01 | 0 | 0 |  | HD23 | H |
| TYR489 | ALA81 | 7.96 | -0.02 | 0 | 0 |  | OH | HB3 |
| PRO499 | GLN41 | 7.97 | -0.05 | 0 | 0 |  | HD1 | HE22 |
| LYS444 | LYS352 | 7.97 | 0.66 | 1 | 1 |  | O | HZ3 |
| PRO499 | ASP353 | 7.97 | 0.10 | 0 | -1 |  | O | HB1 |
| SER443 | LYS352 | 7.97 | 0.10 | 0 | 1 |  | HB1 | HZ3 |
| TYR489 | ALA79 | 8.00 | -0.03 | 0 | 0 |  | OH | HA |

**Supplementary table 17**. Interactions between SARS-CoV-2 RBD e *Neovison vison* ACE2.

| **Interaction** | **SARS-CoV-2  RBD** | ***Neovison vison*  ACE2** |
| --- | --- | --- |
|  | Residue | Residue |
| Hydrophobic | TYR489 | PHE28 |
|  | TYR453 | TYR34 |
|  | LEU455 | TYR34 |
|  | PHE486 | TYR83 |
| Hydrogen bonds | GLY502 | LYS353 |
|  | THR500 | TYR41 |
|  | GLY496 | LYS353 |
|  | THR500 | ASP355 |
|  | ASN501 | TYR41 |
|  | GLN493 | GLU35 |
|  | ASN487 | TYR83 |
|  | ASP405 | HIS354 |
|  | TYR505 | ARG393 |
|  | ASP405 | HIS354 |
|  | LYS417 | GLU30 |
|  | ASN487 | TYR83 |
|  | GLN493 | GLU35 |
|  | THR500 | TYR41 |
|  | ASN501 | TYR41 |
|  | TYR505 | GLU37 |
| Ionic | LYS417 | GLU30 |
|  | GLU484 | LYS31 |
|  | ASP405 | HIS354 |
| Aromatic-Aromatic | TYR453 | TYR34 |
|  | PHE486 | TYR83 |

**Supplementary** **table 18**. Quantum biochemistry between SARS-CoV-2 RBD e *Neovison vison* ACE2.

| **Amino acid residues** | | **Distance** | **Interaction energy** | **Residue charge** | |  | **Residue atom** | |
| --- | --- | --- | --- | --- | --- | --- | --- | --- |
| SARS-CoV-2  RBD | *Neovison vison* ACE2 | (Å) | (kcal mol-1) | SARS-CoV-2  RBD | *Neovison vison* ACE2 |  | SARS-CoV-2  RBD | *Neovison vison* ACE2 |
| TYR505 | GLU37 | 1.75 | -12.02 | 0 | -1 |  | HH | OE1 |
| THR500 | TYR41 | 1.75 | -5.02 | 0 | 0 |  | HG1 | OH |
| LYS417 | GLU30 | 1.77 | -12.67 | 1 | -1 |  | HZ1 | OE1 |
| GLY496 | LYS353 | 1.81 | -4.83 | 0 | 1 |  | O | HZ2 |
| GLY502 | LYS353 | 1.86 | -3.71 | 0 | 1 |  | H | O |
| ASN487 | TYR83 | 1.96 | -3.25 | 0 | 0 |  | HD22 | OH |
| ASP405 | HIS354 | 1.96 | -5.67 | -1 | 0 |  | OD2 | HE2 |
| TYR505 | ARG393 | 1.97 | 3.13 | 0 | 1 |  | HH | HH22 |
| GLY504 | HIS354 | 1.97 | 2.33 | 0 | 0 |  | H | HD2 |
| GLN498 | GLN42 | 2.00 | 0.61 | 0 | 0 |  | HE22 | HE22 |
| GLY502 | HIS354 | 2.03 | -3.85 | 0 | 0 |  | HA2 | HB1 |
| PHE456 | LYS31 | 2.12 | -3.02 | 0 | 1 |  | HE2 | HD2 |
| TYR505 | LYS353 | 2.14 | -8.97 | 0 | 1 |  | HD2 | HD2 |
| LEU455 | TYR34 | 2.22 | -2.30 | 0 | 0 |  | HD12 | HD2 |
| LEU455 | LYS31 | 2.35 | -2.94 | 0 | 1 |  | HD21 | HA |
| GLY496 | GLU38 | 2.41 | -1.37 | 0 | -1 |  | HA2 | OE1 |
| GLN498 | TYR41 | 2.45 | -0.94 | 0 | 0 |  | OE1 | HE2 |
| TYR449 | GLU38 | 2.45 | -3.56 | 0 | -1 |  | HE1 | OE2 |
| ASN501 | LYS353 | 2.46 | -11.93 | 0 | 1 |  | HD22 | HD1 |
| PHE486 | THR82 | 2.48 | -1.70 | 0 | 0 |  | HE1 | HG1 |
| TYR505 | HIS354 | 2.52 | -3.41 | 0 | 0 |  | HE1 | HE2 |
| THR500 | ARG357 | 2.57 | -1.39 | 0 | 1 |  | HB | HH11 |
| ASN501 | TYR41 | 2.57 | -2.37 | 0 | 0 |  | HD21 | OH |
| PHE486 | HIS79 | 2.58 | -3.76 | 0 | 0 |  | HD1 | ND1 |
| PHE486 | TYR83 | 2.61 | -1.91 | 0 | 0 |  | HE1 | HE1 |
| PHE456 | THR27 | 2.62 | -1.11 | 0 | 0 |  | HE1 | HB |
| TYR489 | LYS31 | 2.63 | -3.63 | 0 | 1 |  | CD1 | HE1 |
| ARG403 | TYR34 | 2.77 | -2.17 | 1 | 0 |  | HH12 | HE1 |
| ALA475 | LEU24 | 2.78 | -1.30 | 0 | 0 |  | O | HD22 |
| TYR489 | PHE28 | 2.80 | -1.50 | 0 | 0 |  | OH | HD1 |
| THR500 | ASN330 | 2.80 | -1.82 | 0 | 0 |  | HG22 | OD1 |
| GLN498 | LEU45 | 2.82 | -1.31 | 0 | 0 |  | HG1 | HD12 |
| TYR453 | TYR34 | 2.84 | -1.92 | 0 | 0 |  | HH | HB2 |
| LYS417 | TYR34 | 2.85 | -1.34 | 1 | 0 |  | HG2 | HE2 |
| GLN493 | LYS31 | 2.88 | 0.22 | 0 | 1 |  | HE22 | HZ3 |
| THR500 | ASP355 | 2.89 | -1.07 | 0 | -1 |  | O | HB1 |
| GLN493 | GLU35 | 2.91 | -4.81 | 0 | -1 |  | HE21 | OE2 |
| ASN501 | ASP355 | 3.03 | -1.05 | 0 | -1 |  | HA | HB1 |
| ARG403 | HIS354 | 3.09 | 0.87 | 1 | 0 |  | HH21 | HE2 |
| THR500 | LEU45 | 3.17 | -0.99 | 0 | 0 |  | HG1 | HD23 |
| TYR449 | GLN42 | 3.53 | -0.94 | 0 | 0 |  | HH | HE21 |
| LEU455 | GLU35 | 3.65 | -0.92 | 0 | -1 |  | HD22 | HG1 |
| SER494 | GLU38 | 3.70 | -0.87 | 0 | -1 |  | O | HG2 |
| GLN498 | LYS353 | 3.70 | -0.35 | 0 | 1 |  | OE1 | HZ2 |
| GLY502 | ASP355 | 3.83 | -1.26 | 0 | -1 |  | H | HB1 |
| VAL503 | HIS354 | 3.84 | 2.87 | 0 | 0 |  | N | HB1 |
| GLU484 | LYS31 | 3.86 | -4.01 | -1 | 1 |  | OE1 | HZ1 |
| TYR489 | TYR83 | 3.87 | -0.32 | 0 | 0 |  | OH | HH |
| LEU455 | GLU30 | 3.92 | -0.55 | 0 | -1 |  | HD12 | O |
| ASN487 | LEU24 | 3.99 | -0.44 | 0 | 0 |  | OD1 | HD22 |
| PHE497 | LYS353 | 4.00 | -2.78 | 0 | 1 |  | N | HZ2 |
| ASN487 | PHE28 | 4.02 | -0.60 | 0 | 0 |  | HD22 | HD1 |
| PHE490 | LYS31 | 4.10 | -2.50 | 0 | 1 |  | O | HZ3 |
| TYR505 | TYR34 | 4.10 | -0.67 | 0 | 0 |  | HE2 | HD1 |
| GLY446 | GLN42 | 4.11 | -0.21 | 0 | 0 |  | O | OE1 |
| GLN493 | TYR34 | 4.12 | -0.72 | 0 | 0 |  | HG1 | HB1 |
| TYR495 | LYS353 | 4.20 | 2.30 | 0 | 1 |  | HA | HZ3 |
| GLY476 | LEU24 | 4.26 | -0.24 | 0 | 0 |  | HA1 | HD22 |
| TYR473 | THR27 | 4.36 | -0.31 | 0 | 0 |  | HE2 | HB |
| VAL503 | THR324 | 4.40 | -0.08 | 0 | 0 |  | HG22 | HB |
| GLY496 | GLN42 | 4.42 | -0.28 | 0 | 0 |  | HA2 | HE22 |
| TYR489 | HIS79 | 4.47 | -0.82 | 0 | 0 |  | HH | HB2 |
| ASN501 | GLY352 | 4.48 | -0.14 | 0 | 0 |  | HA | O |
| PHE486 | PHE28 | 4.49 | -0.43 | 0 | 0 |  | HD1 | HE1 |
| ALA475 | THR27 | 4.54 | -0.31 | 0 | 0 |  | HB3 | OG1 |
| ALA475 | TYR83 | 4.54 | 0.02 | 0 | 0 |  | HB3 | HH |
| GLY447 | GLN42 | 4.55 | 0.07 | 0 | 0 |  | HA2 | HE22 |
| TYR421 | GLU30 | 4.57 | -0.05 | 0 | -1 |  | HE2 | OE1 |
| PHE456 | PHE28 | 4.62 | -0.21 | 0 | 0 |  | HZ | HA |
| GLY502 | GLY326 | 4.63 | 0.01 | 0 | 0 |  | HA1 | HA2 |
| GLY502 | GLY352 | 4.65 | -0.05 | 0 | 0 |  | H | O |
| GLU406 | TYR34 | 4.67 | 0.30 | -1 | 0 |  | OE2 | HE1 |
| TYR495 | GLU38 | 4.67 | -1.81 | 0 | -1 |  | C | OE1 |
| THR500 | GLY326 | 4.68 | -0.56 | 0 | 0 |  | HA | HA1 |
| ASN501 | HIS354 | 4.69 | -3.66 | 0 | 0 |  | C | HB1 |
| GLY446 | LEU45 | 4.69 | -0.13 | 0 | 0 |  | H | HD11 |
| GLN498 | GLU38 | 4.71 | -0.81 | 0 | -1 |  | HE22 | OE1 |
| ARG403 | LYS353 | 4.75 | 1.12 | 1 | 1 |  | HD1 | HD2 |
| TYR505 | PHE390 | 4.77 | -0.15 | 0 | 0 |  | HH | HD1 |
| GLY502 | THR324 | 4.78 | 0.07 | 0 | 0 |  | HA2 | HG21 |
| PHE456 | GLU30 | 4.82 | -0.86 | 0 | -1 |  | HE1 | HB1 |
| LYS417 | THR27 | 4.83 | -0.39 | 1 | 0 |  | HZ1 | HG23 |
| VAL503 | GLY326 | 4.85 | -0.03 | 0 | 0 |  | HG22 | H |
| GLY485 | HIS79 | 4.86 | -0.32 | 0 | 0 |  | C | HE1 |
| ASN487 | THR82 | 4.89 | -0.41 | 0 | 0 |  | HD22 | OG1 |
| GLY504 | LYS353 | 4.92 | -0.20 | 0 | 1 |  | H | O |
| TYR449 | LEU39 | 4.94 | -0.43 | 0 | 0 |  | HH | HD22 |
| LYS417 | LYS31 | 4.95 | 0.34 | 1 | 1 |  | HZ2 | HA |
| TYR489 | THR27 | 4.97 | -0.32 | 0 | 0 |  | HE2 | HB |
| TYR453 | GLU38 | 4.98 | -0.19 | 0 | -1 |  | HH | HG2 |
| GLN493 | GLU38 | 5.00 | -0.81 | 0 | -1 |  | HG2 | HG2 |
| ASN501 | LEU45 | 5.03 | -0.18 | 0 | 0 |  | HD22 | HD12 |
| VAL503 | LYS353 | 5.05 | 0.50 | 0 | 1 |  | N | O |
| GLN506 | HIS354 | 5.08 | -0.30 | 0 | 0 |  | H | HD2 |
| PHE486 | ALA80 | 5.11 | -0.12 | 0 | 0 |  | HE1 | N |
| ASN487 | HIS79 | 5.12 | -0.67 | 0 | 0 |  | H | ND1 |
| VAL445 | LEU45 | 5.14 | -0.16 | 0 | 0 |  | HG12 | HD11 |
| THR500 | LEU351 | 5.16 | -0.23 | 0 | 0 |  | HB | HD22 |
| GLY496 | TYR41 | 5.18 | -0.22 | 0 | 0 |  | O | HD2 |
| VAL503 | GLU325 | 5.18 | -0.07 | 0 | -1 |  | HG22 | H |
| PRO499 | ASN330 | 5.21 | -0.33 | 0 | 0 |  | O | HD22 |
| TYR505 | ALA386 | 5.27 | -0.10 | 0 | 0 |  | HE1 | O |
| TYR505 | ALA387 | 5.29 | -0.17 | 0 | 0 |  | HE1 | HA |
| ASN501 | GLN42 | 5.30 | -0.12 | 0 | 0 |  | HD22 | HE22 |
| TYR489 | GLU76 | 5.32 | -0.76 | 0 | -1 |  | HE1 | OE2 |
| TYR453 | GLU35 | 5.34 | -0.41 | 0 | -1 |  | HH | HG1 |
| LEU455 | PHE32 | 5.34 | -0.25 | 0 | 0 |  | HD21 | N |
| GLY502 | TYR41 | 5.36 | -0.16 | 0 | 0 |  | H | HE1 |
| PHE497 | GLU38 | 5.38 | 0.17 | 0 | -1 |  | N | OE1 |
| GLN506 | LYS353 | 5.39 | -0.50 | 0 | 1 |  | H | O |
| ARG408 | ALA387 | 5.40 | -0.18 | 1 | 0 |  | HH12 | HB3 |
| THR500 | HIS354 | 5.43 | -0.39 | 0 | 0 |  | O | O |
| THR500 | LYS353 | 5.43 | -0.15 | 0 | 1 |  | O | HB1 |
| GLN409 | TYR34 | 5.45 | -0.11 | 0 | 0 |  | HE22 | HH |
| ASN501 | ARG357 | 5.51 | -0.29 | 0 | 1 |  | N | HH11 |
| PHE486 | GLN78 | 5.53 | -0.18 | 0 | 0 |  | HE1 | O |
| TYR449 | LYS353 | 5.62 | -0.29 | 0 | 1 |  | HE1 | HZ1 |
| SER494 | LYS353 | 5.63 | 0.00 | 0 | 1 |  | O | HZ3 |
| VAL445 | ASN49 | 5.63 | -0.01 | 0 | 0 |  | HG12 | HD22 |
| GLY502 | PHE356 | 5.63 | -0.21 | 0 | 0 |  | HA2 | HD2 |
| ASN501 | GLY326 | 5.70 | -0.26 | 0 | 0 |  | O | HA1 |
| GLY447 | TYR41 | 5.77 | -0.14 | 0 | 0 |  | HA1 | HE2 |
| ALA475 | PHE28 | 5.77 | -0.12 | 0 | 0 |  | HB3 | HB1 |
| THR500 | PHE356 | 5.77 | 0.02 | 0 | 0 |  | O | H |
| TYR495 | TYR34 | 5.77 | -0.25 | 0 | 0 |  | HE1 | HE1 |
| THR500 | GLY352 | 5.78 | -0.07 | 0 | 0 |  | O | O |
| TYR505 | GLY352 | 5.78 | -0.15 | 0 | 0 |  | HB1 | O |
| TYR505 | ASP355 | 5.79 | -0.25 | 0 | -1 |  | HB1 | H |
| CYS488 | LYS31 | 5.80 | 0.08 | 0 | 1 |  | O | HZ1 |
| PHE456 | PHE32 | 5.82 | -0.13 | 0 | 0 |  | HZ | H |
| THR500 | GLN329 | 5.82 | -0.19 | 0 | 0 |  | HG22 | HB2 |
| PRO499 | TYR41 | 5.83 | -0.87 | 0 | 0 |  | HD1 | HE2 |
| SER494 | GLU35 | 5.91 | -0.12 | 0 | -1 |  | H | OE1 |
| PHE456 | GLU35 | 5.92 | -0.05 | 0 | -1 |  | HE2 | HG2 |
| GLN506 | GLY326 | 5.94 | -0.01 | 0 | 0 |  | HE21 | HA1 |
| TYR505 | ASN33 | 5.97 | -0.27 | 0 | 0 |  | HH | O |
| TYR421 | THR27 | 5.97 | -0.07 | 0 | 0 |  | OH | HG23 |
| LEU455 | THR27 | 5.97 | -0.18 | 0 | 0 |  | HD21 | O |
| ASN487 | THR27 | 5.97 | -0.31 | 0 | 0 |  | OD1 | OG1 |
| GLY447 | LYS353 | 5.98 | 0.10 | 0 | 1 |  | HA2 | HZ2 |
| TYR449 | LYS68 | 5.99 | -0.02 | 0 | 1 |  | HH | HZ1 |
| GLN498 | ARG357 | 6.02 | 0.01 | 0 | 1 |  | HB2 | HH12 |
| SER477 | LEU24 | 6.04 | -0.09 | 0 | 0 |  | H | HD13 |
| TYR489 | GLU35 | 6.05 | -0.28 | 0 | -1 |  | HB2 | OE2 |
| LEU492 | LYS31 | 6.06 | -0.33 | 0 | 1 |  | O | HZ3 |
| SER494 | TYR34 | 6.06 | -0.05 | 0 | 0 |  | O | HB2 |
| GLY446 | TYR41 | 6.08 | -0.15 | 0 | 0 |  | H | HE2 |
| ARG403 | ALA387 | 6.08 | -0.10 | 1 | 0 |  | HH22 | HA |
| PRO499 | GLY326 | 6.10 | -0.04 | 0 | 0 |  | O | HA1 |
| PHE497 | TYR41 | 6.10 | -0.19 | 0 | 0 |  | C | HE2 |
| PRO491 | LYS31 | 6.11 | -0.60 | 0 | 1 |  | HA | HZ3 |
| GLN498 | ASP355 | 6.11 | -0.11 | 0 | -1 |  | HB1 | OD2 |
| GLN506 | GLU325 | 6.12 | -0.03 | 0 | -1 |  | HE22 | HB2 |
| ASN501 | GLU38 | 6.17 | -0.09 | 0 | -1 |  | HD22 | OE1 |
| TYR505 | GLU38 | 6.18 | -0.06 | 0 | -1 |  | HE2 | HG1 |
| TYR505 | GLN388 | 6.18 | -0.09 | 0 | 0 |  | HH | O |
| TYR449 | TYR41 | 6.19 | -0.14 | 0 | 0 |  | OH | HD2 |
| TYR453 | LYS353 | 6.24 | 0.00 | 0 | 1 |  | HE1 | HZ3 |
| ASP405 | LYS353 | 6.25 | -1.03 | -1 | 1 |  | OD2 | HA |
| PHE497 | GLN42 | 6.26 | -0.11 | 0 | 0 |  | N | HE22 |
| ASP405 | ALA387 | 6.28 | -0.16 | -1 | 0 |  | OD2 | HA |
| LYS417 | LYS26 | 6.29 | 0.77 | 1 | 1 |  | HZ1 | O |
| GLY447 | LEU45 | 6.32 | -0.06 | 0 | 0 |  | N | HD11 |
| TYR489 | LEU24 | 6.34 | -0.10 | 0 | 0 |  | HE2 | HD22 |
| PHE486 | LYS81 | 6.36 | -0.13 | 0 | 1 |  | HE1 | N |
| ARG408 | HIS354 | 6.37 | 0.08 | 1 | 0 |  | HH22 | HE2 |
| GLU484 | GLU35 | 6.39 | 1.87 | -1 | -1 |  | OE1 | OE2 |
| PHE486 | PRO84 | 6.40 | -0.06 | 0 | 0 |  | HZ | HD2 |
| PHE486 | LEU24 | 6.41 | -0.13 | 0 | 0 |  | HZ | HD13 |
| PRO499 | LEU45 | 6.42 | -0.15 | 0 | 0 |  | HD1 | HD12 |
| GLY447 | GLU38 | 6.43 | -0.12 | 0 | -1 |  | HA2 | OE1 |
| GLY416 | TYR34 | 6.44 | 0.00 | 0 | 0 |  | HA1 | HH |
| VAL503 | ASP355 | 6.47 | 0.05 | 0 | -1 |  | H | HA |
| ILE418 | TYR34 | 6.48 | -0.21 | 0 | 0 |  | HG11 | OH |
| PRO499 | GLN329 | 6.48 | -0.06 | 0 | 0 |  | O | OE1 |
| ARG403 | GLU37 | 6.49 | -1.76 | 1 | -1 |  | NH2 | OE1 |
| ARG403 | ARG393 | 6.50 | 1.34 | 1 | 1 |  | HH22 | HH21 |
| THR500 | THR324 | 6.51 | -0.11 | 0 | 0 |  | O | HG1 |
| GLY485 | LYS31 | 6.51 | -0.33 | 0 | 1 |  | O | HE1 |
| PHE486 | GLU75 | 6.52 | -0.14 | 0 | -1 |  | HD1 | O |
| TYR473 | LYS31 | 6.53 | -0.24 | 0 | 1 |  | HD2 | HB1 |
| VAL445 | TYR41 | 6.57 | -0.06 | 0 | 0 |  | HA | HE2 |
| PHE456 | LYS26 | 6.59 | 0.05 | 0 | 1 |  | HE1 | O |
| ASN501 | LEU351 | 6.59 | -0.07 | 0 | 0 |  | HA | HB1 |
| ASN501 | ASN330 | 6.60 | -0.14 | 0 | 0 |  | N | HD22 |
| ASN487 | LYS31 | 6.62 | 0.06 | 0 | 1 |  | HA | HE1 |
| GLY404 | HIS354 | 6.66 | 0.10 | 0 | 0 |  | HA2 | HD2 |
| ASN448 | LYS353 | 6.67 | -0.12 | 0 | 1 |  | O | HZ2 |
| THR500 | TRP48 | 6.69 | -0.09 | 0 | 0 |  | HG21 | HD1 |
| TYR421 | TYR34 | 6.70 | -0.06 | 0 | 0 |  | HE2 | HE2 |
| PHE490 | GLU35 | 6.70 | -0.01 | 0 | -1 |  | O | OE2 |
| ALA475 | LYS31 | 6.70 | -0.02 | 0 | 1 |  | HB1 | HB1 |
| LEU455 | ASN33 | 6.71 | -0.13 | 0 | 0 |  | HD12 | C |
| GLY485 | GLU75 | 6.73 | -0.16 | 0 | -1 |  | HA1 | OE1 |
| TYR505 | PRO389 | 6.74 | -0.02 | 0 | 0 |  | HH | HA |
| GLN493 | PHE72 | 6.75 | -0.04 | 0 | 0 |  | HE21 | HE1 |
| PHE456 | LEU24 | 6.76 | -0.07 | 0 | 0 |  | HE1 | O |
| LEU492 | GLU35 | 6.78 | 0.03 | 0 | -1 |  | O | OE2 |
| GLN474 | LEU24 | 6.78 | -0.07 | 0 | 0 |  | O | HD22 |
| GLY476 | TYR83 | 6.80 | -0.06 | 0 | 0 |  | HA1 | HH |
| TYR473 | LEU24 | 6.82 | -0.07 | 0 | 0 |  | HE2 | HD22 |
| GLN493 | GLU30 | 6.83 | 0.04 | 0 | -1 |  | HG1 | O |
| ASN501 | THR324 | 6.83 | -0.06 | 0 | 0 |  | O | HG1 |
| GLN506 | ASN330 | 6.88 | -0.01 | 0 | 0 |  | HE21 | HD22 |
| TYR453 | LYS31 | 6.90 | -0.14 | 0 | 1 |  | OH | O |
| LEU455 | PHE28 | 6.90 | -0.08 | 0 | 0 |  | HD21 | O |
| GLN506 | ASP355 | 6.91 | -0.18 | 0 | -1 |  | HE21 | HB1 |
| GLY502 | PHE327 | 6.92 | -0.02 | 0 | 0 |  | HA1 | H |
| PHE486 | GLU76 | 6.92 | -0.27 | 0 | -1 |  | HD1 | HA |
| TYR453 | GLU30 | 6.93 | -0.06 | 0 | -1 |  | HE2 | O |
| TYR489 | PHE32 | 6.93 | -0.23 | 0 | 0 |  | OH | H |
| ASN487 | ALA25 | 6.94 | -0.02 | 0 | 0 |  | HD22 | HA |
| GLN506 | GLN329 | 6.94 | -0.06 | 0 | 0 |  | HE22 | OE1 |
| GLY502 | GLU325 | 6.95 | 0.17 | 0 | -1 |  | HA1 | H |
| ASN501 | PHE356 | 6.95 | -0.12 | 0 | 0 |  | HA | H |
| ALA475 | ASP23 | 6.96 | -0.02 | 0 | -1 |  | HB3 | O |
| TYR505 | TYR41 | 6.96 | -0.15 | 0 | 0 |  | HB1 | HE1 |
| PRO499 | ASP355 | 6.98 | -0.49 | 0 | -1 |  | C | OD2 |
| ARG457 | THR27 | 6.98 | -0.07 | 1 | 0 |  | H | HG23 |
| TYR449 | LEU45 | 6.98 | -0.05 | 0 | 0 |  | OH | HD13 |
| ASP405 | TYR34 | 6.99 | 0.05 | -1 | 0 |  | HB1 | HE1 |
| PHE456 | LEU29 | 7.00 | -0.08 | 0 | 0 |  | HE1 | N |
| TYR489 | GLU75 | 7.00 | -0.11 | 0 | -1 |  | HE1 | HG1 |
| GLY504 | THR324 | 7.00 | 0.00 | 0 | 0 |  | H | HG21 |
| LYS444 | TYR41 | 7.00 | -0.06 | 1 | 0 |  | O | HE2 |
| PHE456 | TYR83 | 7.01 | 0.00 | 0 | 0 |  | HZ | HH |
| TYR453 | GLU37 | 7.01 | -0.03 | 0 | -1 |  | HH | HB2 |
| CYS488 | HIS79 | 7.03 | -0.10 | 0 | 0 |  | H | HE1 |
| PRO499 | ARG357 | 7.06 | 0.22 | 0 | 1 |  | C | HH11 |
| GLY496 | LEU45 | 7.06 | -0.03 | 0 | 0 |  | O | HD12 |
| LYS417 | LEU29 | 7.06 | 0.51 | 1 | 0 |  | HZ1 | C |
| ASN487 | ALA80 | 7.06 | -0.04 | 0 | 0 |  | HD22 | HA |
| ASN501 | GLU37 | 7.09 | -0.28 | 0 | -1 |  | HD22 | HG1 |
| GLY485 | PHE28 | 7.11 | -0.02 | 0 | 0 |  | O | HE1 |
| GLY496 | GLU37 | 7.11 | -0.05 | 0 | -1 |  | HA2 | O |
| ASN448 | GLU38 | 7.13 | 0.00 | 0 | -1 |  | O | OE1 |
| ARG403 | GLU38 | 7.15 | -1.09 | 1 | -1 |  | HD1 | HG1 |
| PHE456 | ASP23 | 7.16 | -0.10 | 0 | -1 |  | HE1 | O |
| ARG403 | ALA386 | 7.18 | -0.20 | 1 | 0 |  | HH21 | O |
| ASN439 | ASN330 | 7.18 | 0.02 | 0 | 0 |  | HD22 | HD22 |
| GLN498 | GLU37 | 7.19 | 0.12 | 0 | -1 |  | HE22 | O |
| VAL445 | ASN330 | 7.20 | -0.06 | 0 | 0 |  | HG21 | OD1 |
| GLY446 | ASN49 | 7.21 | 0.00 | 0 | 0 |  | H | HD22 |
| CYS488 | PHE28 | 7.22 | -0.06 | 0 | 0 |  | H | HE1 |
| GLN498 | LEU39 | 7.23 | -0.08 | 0 | 0 |  | HE22 | HA |
| ASP405 | ALA386 | 7.24 | -0.02 | -1 | 0 |  | OD2 | O |
| ASN448 | GLN42 | 7.25 | -0.03 | 0 | 0 |  | H | HE22 |
| VAL503 | PHE356 | 7.26 | -0.04 | 0 | 0 |  | H | HD2 |
| THR500 | PHE327 | 7.26 | -0.07 | 0 | 0 |  | O | N |
| VAL445 | ARG357 | 7.27 | 0.07 | 0 | 1 |  | HG21 | HH12 |
| GLU484 | HIS79 | 7.27 | -0.13 | -1 | 0 |  | HB2 | HE2 |
| TYR489 | THR82 | 7.28 | -0.02 | 0 | 0 |  | HH | HG1 |
| ILE472 | LYS31 | 7.30 | 0.22 | 0 | 1 |  | HG23 | HZ1 |
| ARG457 | GLU30 | 7.34 | -0.71 | 1 | -1 |  | H | OE1 |
| TYR505 | PHE356 | 7.36 | -0.08 | 0 | 0 |  | HD1 | HE2 |
| LEU455 | LEU29 | 7.36 | -0.09 | 0 | 0 |  | HD12 | O |
| LYS417 | ASN33 | 7.37 | 0.17 | 1 | 0 |  | HE2 | HB1 |
| LYS444 | LEU45 | 7.37 | -0.01 | 1 | 0 |  | O | HD11 |
| GLN506 | TYR41 | 7.37 | -0.06 | 0 | 0 |  | HE21 | OH |
| GLY502 | LEU351 | 7.38 | -0.01 | 0 | 0 |  | H | HB1 |
| VAL503 | PHE327 | 7.40 | -0.02 | 0 | 0 |  | HG22 | H |
| GLY504 | PHE356 | 7.40 | -0.02 | 0 | 0 |  | H | HE2 |
| TYR508 | HIS354 | 7.42 | -0.02 | 0 | 0 |  | HE1 | HD2 |
| CYS488 | TYR83 | 7.42 | -0.04 | 0 | 0 |  | H | HH |
| GLY496 | LEU39 | 7.42 | -0.02 | 0 | 0 |  | HA2 | N |
| GLY502 | ASN330 | 7.43 | -0.01 | 0 | 0 |  | HA1 | HD22 |
| GLY504 | ASP355 | 7.44 | -0.10 | 0 | -1 |  | H | N |
| THR500 | GLU325 | 7.45 | -0.24 | 0 | -1 |  | HA | C |
| GLY502 | ARG357 | 7.46 | -0.08 | 0 | 1 |  | N | HH11 |
| GLU406 | HIS354 | 7.46 | -0.20 | -1 | 0 |  | H | HE2 |
| PHE486 | LYS31 | 7.47 | -0.11 | 0 | 1 |  | HA | HE1 |
| GLN506 | THR324 | 7.47 | -0.03 | 0 | 0 |  | HE21 | HB |
| ASN439 | GLN329 | 7.51 | -0.03 | 0 | 0 |  | HD21 | HE22 |
| PHE497 | LEU45 | 7.52 | -0.01 | 0 | 0 |  | C | HD12 |
| TYR489 | ALA80 | 7.52 | -0.01 | 0 | 0 |  | HH | N |
| TYR473 | GLU30 | 7.53 | -0.07 | 0 | -1 |  | HH | OE1 |
| VAL445 | LYS61 | 7.54 | -0.04 | 0 | 1 |  | HG11 | HZ1 |
| THR500 | ASN49 | 7.56 | -0.04 | 0 | 0 |  | HG21 | HD22 |
| TYR453 | ASN33 | 7.57 | -0.16 | 0 | 0 |  | OH | C |
| GLY496 | TYR34 | 7.57 | -0.03 | 0 | 0 |  | H | O |
| THR500 | GLN42 | 7.58 | -0.07 | 0 | 0 |  | HG1 | HE22 |
| ASN448 | TYR41 | 7.58 | -0.03 | 0 | 0 |  | H | HE2 |
| TYR495 | GLN42 | 7.59 | 0.04 | 0 | 0 |  | C | HE22 |
| TYR489 | LEU29 | 7.59 | -0.08 | 0 | 0 |  | OH | N |
| ASN439 | GLY326 | 7.60 | -0.01 | 0 | 0 |  | HD22 | HA1 |
| TYR473 | PHE28 | 7.60 | -0.05 | 0 | 0 |  | HE2 | N |
| PRO507 | LYS353 | 7.61 | -0.03 | 0 | 1 |  | HD2 | HD1 |
| LEU455 | GLU38 | 7.63 | -0.05 | 0 | -1 |  | HD11 | HG2 |
| GLU484 | GLU75 | 7.64 | 1.18 | -1 | -1 |  | HB2 | OE1 |
| GLN493 | PHE32 | 7.64 | -0.08 | 0 | 0 |  | HG1 | N |
| GLN498 | GLY352 | 7.65 | -0.04 | 0 | 0 |  | HB1 | O |
| LYS417 | PHE28 | 7.65 | -0.21 | 1 | 0 |  | HZ1 | N |
| ARG454 | TYR34 | 7.67 | 0.02 | 1 | 0 |  | O | HE2 |
| PHE456 | TYR34 | 7.69 | -0.24 | 0 | 0 |  | N | HD2 |
| GLY446 | GLU38 | 7.69 | 0.02 | 0 | -1 |  | O | OE2 |
| TYR449 | GLU35 | 7.72 | -0.12 | 0 | -1 |  | HE1 | OE1 |
| TYR473 | ASP23 | 7.73 | -0.06 | 0 | -1 |  | HH | O |
| PHE486 | SER77 | 7.73 | -0.04 | 0 | 0 |  | HE1 | O |
| LYS417 | PHE32 | 7.74 | -0.07 | 1 | 0 |  | HZ2 | N |
| SER494 | LYS31 | 7.74 | -0.11 | 0 | 1 |  | H | HZ2 |
| ALA475 | ALA25 | 7.75 | -0.03 | 0 | 0 |  | HB3 | N |
| TYR489 | PHE72 | 7.75 | -0.05 | 0 | 0 |  | HE1 | HZ |
| GLY416 | GLU30 | 7.76 | -0.21 | 0 | -1 |  | HA1 | HG1 |
| GLY502 | MET383 | 7.76 | -0.02 | 0 | 0 |  | HA2 | HG1 |
| LYS458 | THR27 | 7.77 | -0.05 | 1 | 0 |  | HA | HG22 |
| GLY446 | LYS61 | 7.81 | 0.00 | 0 | 1 |  | HA1 | HZ1 |
| GLY502 | GLU37 | 7.81 | 0.06 | 0 | -1 |  | H | OE2 |
| ASP420 | GLU30 | 7.82 | 1.29 | -1 | -1 |  | OD2 | OE1 |
| ASP405 | ARG393 | 7.82 | -1.17 | -1 | 1 |  | OD2 | HH22 |
| ASN487 | ASP23 | 7.83 | 0.00 | 0 | -1 |  | OD1 | O |
| TYR449 | GLU37 | 7.83 | 0.01 | 0 | -1 |  | HH | O |
| LEU455 | PHE72 | 7.83 | -0.01 | 0 | 0 |  | HD22 | HZ |
| GLY446 | LYS353 | 7.84 | 0.00 | 0 | 1 |  | O | HZ2 |
| GLN498 | ASN330 | 7.85 | -0.08 | 0 | 0 |  | HB2 | OD1 |
| GLN498 | LEU351 | 7.90 | -0.04 | 0 | 0 |  | HG1 | HD23 |
| VAL445 | GLN42 | 7.91 | -0.30 | 0 | 0 |  | C | OE1 |
| LEU455 | ALA36 | 7.91 | -0.03 | 0 | 0 |  | HD12 | H |
| PHE486 | THR21 | 7.94 | -0.02 | 0 | 0 |  | HZ | HG22 |
| TYR489 | GLU30 | 7.94 | -0.01 | 0 | -1 |  | HE2 | HB1 |
| ASN487 | GLU76 | 7.95 | -0.24 | 0 | -1 |  | H | OE2 |
| GLY476 | THR27 | 7.96 | -0.02 | 0 | 0 |  | N | OG1 |
| SER443 | LYS353 | 7.96 | 0.08 | 0 | 1 |  | HB1 | HZ2 |
| GLN493 | ALA36 | 7.97 | -0.09 | 0 | 0 |  | HG2 | N |
| GLN498 | ASN49 | 7.98 | 0.01 | 0 | 0 |  | HG1 | HD22 |
| GLN493 | LEU39 | 7.99 | -0.04 | 0 | 0 |  | HE21 | HD11 |
| ASN439 | GLU325 | 8.00 | -0.05 | 0 | -1 |  | HD21 | HB2 |

**Supplementary** **table 19**. Interactions between SARS-CoV-2 RBD e *Emballonura alecto* ACE2.

| **Interaction** | **SARS-CoV-2  RBD** | ***Emballonura alecto*  ACE2** |
| --- | --- | --- |
|  | Residue | Residue |
| Hydrophobic | PHE486 | PHE28 |
|  | PHE486 | LEU79 |
|  | PHE486 | TYR83 |
|  | TYR505 | ALA382 |
| Hydrogen bonds | GLY502 | GLY350 |
|  | ASN501 | ASN349 |
|  | GLY496 | ASN349 |
|  | LYS417 | ASP30 |
|  | ASN487 | TYR83 |
|  | GLN498 | ASN349 |
|  | THR500 | ASN326 |
|  | THR500 | ASP351 |
|  | TYR505 | GLU37 |
|  | GLN493 | LYS31 |
|  | GLN498 | ASN349 |
|  | TYR505 | ARG389 |
| Ionic | ARG403 | GLU37 |
|  | LYS417 | ASP30 |
|  | GLU484 | LYS31 |
| Aromatic-Aromatic | PHE486 | TYR83 |
| Cation-Pi | TYR489 | LYS31 |

**Supplementary** **table 20**. Quantum biochemistry between SARS-CoV-2 RBD e *Emballonura alecto* ACE2.

| **Amino acid residues** | | **Distance** | **Interaction energy** | **Residue charge** | |  | **Residue atom** | |
| --- | --- | --- | --- | --- | --- | --- | --- | --- |
| SARS-CoV-2  RBD | *Emballonura alecto* ACE2 | (Å) | (kcal mol-1) | SARS-CoV-2  RBD | *Emballonura alecto* ACE2 |  | SARS-CoV-2  RBD | *Emballonura alecto* ACE2 |
| THR500 | ASP351 | 1.60 | -10.13 | 0 | -1 |  | HG1 | OD2 |
| LYS417 | ASP30 | 1.61 | -12.62 | 1 | -1 |  | HZ1 | OD2 |
| TYR505 | GLU37 | 1.79 | -8.39 | 0 | -1 |  | HH | OE2 |
| PHE486 | TYR83 | 1.99 | -0.14 | 0 | 0 |  | HE1 | HH |
| GLY496 | ASN349 | 1.99 | -3.57 | 0 | 0 |  | O | HD22 |
| THR500 | GLY322 | 2.11 | -0.81 | 0 | 0 |  | HB | HA1 |
| TYR473 | THR27 | 2.11 | -1.46 | 0 | 0 |  | HE2 | HG23 |
| ASN487 | TYR83 | 2.20 | -1.74 | 0 | 0 |  | HD22 | OH |
| PHE456 | THR27 | 2.21 | -2.19 | 0 | 0 |  | HE1 | HG21 |
| THR500 | ASN326 | 2.26 | -2.17 | 0 | 0 |  | HG22 | HD22 |
| ALA475 | THR27 | 2.29 | -1.46 | 0 | 0 |  | HB1 | HG1 |
| PHE456 | ASP30 | 2.31 | -1.48 | 0 | -1 |  | HE1 | HB1 |
| GLN493 | LYS31 | 2.36 | -1.59 | 0 | 1 |  | HE22 | HZ3 |
| GLN498 | ASN349 | 2.36 | -2.54 | 0 | 0 |  | OE1 | HD22 |
| TYR505 | ARG389 | 2.37 | -0.45 | 0 | 1 |  | HH | HH12 |
| PHE456 | LYS31 | 2.39 | -2.44 | 0 | 1 |  | HE2 | HD2 |
| GLN493 | SER34 | 2.39 | -1.70 | 0 | 0 |  | HG1 | HB1 |
| TYR489 | LYS31 | 2.44 | -6.90 | 0 | 1 |  | HD1 | HE1 |
| ALA475 | GLN24 | 2.49 | -2.90 | 0 | 0 |  | O | HG1 |
| TYR505 | GLY350 | 2.52 | -1.65 | 0 | 0 |  | HD1 | HA2 |
| TYR505 | ASN349 | 2.52 | -2.53 | 0 | 0 |  | HH | H |
| ASN501 | ASN349 | 2.55 | -0.24 | 0 | 0 |  | HD22 | HD21 |
| VAL503 | THR320 | 2.56 | -0.61 | 0 | 0 |  | HG21 | HG22 |
| PHE486 | LEU79 | 2.62 | -3.79 | 0 | 0 |  | HD1 | HD11 |
| GLY502 | GLY350 | 2.62 | -0.03 | 0 | 0 |  | H | HA2 |
| GLY502 | THR320 | 2.64 | -0.49 | 0 | 0 |  | HA1 | HG21 |
| LEU455 | ASP30 | 2.68 | -1.80 | 0 | -1 |  | HD23 | O |
| GLN498 | PHE41 | 2.72 | -2.21 | 0 | 0 |  | HE22 | HE1 |
| ASN501 | GLY350 | 2.79 | -6.25 | 0 | 0 |  | HA | O |
| TYR489 | THR27 | 2.79 | -1.34 | 0 | 0 |  | HE2 | O |
| LEU455 | LYS31 | 2.81 | -1.29 | 0 | 1 |  | HD23 | HA |
| LEU455 | SER34 | 2.88 | -1.88 | 0 | 0 |  | HD23 | HB2 |
| TYR505 | ALA382 | 2.92 | 0.06 | 0 | 0 |  | HE1 | HB1 |
| ASP405 | VAL383 | 3.09 | -1.23 | -1 | 0 |  | OD2 | HG12 |
| THR500 | ASN325 | 3.11 | -0.68 | 0 | 0 |  | HG22 | HB1 |
| GLY476 | GLN24 | 3.14 | -1.60 | 0 | 0 |  | HA1 | HG1 |
| ARG408 | VAL383 | 3.15 | -0.33 | 1 | 0 |  | HH12 | HG11 |
| THR500 | GLU321 | 3.22 | -1.43 | 0 | -1 |  | HA | O |
| PHE486 | PHE28 | 3.25 | -1.04 | 0 | 0 |  | HE1 | HD1 |
| GLN506 | GLU321 | 3.28 | -0.97 | 0 | -1 |  | HE22 | HB1 |
| TYR489 | PHE28 | 3.28 | -1.38 | 0 | 0 |  | HH | HA |
| GLY502 | PHE352 | 3.35 | -0.41 | 0 | 0 |  | HA2 | HE2 |
| ASN501 | THR320 | 3.40 | -0.43 | 0 | 0 |  | O | HB |
| PHE486 | ALA82 | 3.44 | -0.49 | 0 | 0 |  | HZ | HB3 |
| THR500 | ARG353 | 3.58 | 0.09 | 0 | 1 |  | HG1 | HH11 |
| GLU484 | LYS31 | 3.67 | -4.52 | -1 | 1 |  | OE1 | HZ1 |
| TYR449 | PHE41 | 3.71 | -0.31 | 0 | 0 |  | HH | HE1 |
| TYR505 | PHE352 | 3.73 | -0.71 | 0 | 0 |  | HE1 | HZ |
| ASN501 | ASP351 | 3.79 | -4.64 | 0 | -1 |  | HA | HA |
| PHE490 | LYS31 | 3.84 | -2.31 | 0 | 1 |  | O | HZ3 |
| THR500 | GLY350 | 3.86 | 0.36 | 0 | 0 |  | O | O |
| VAL503 | LYS318 | 3.92 | -0.77 | 0 | 1 |  | HG22 | HE1 |
| ASN501 | GLY322 | 3.96 | -0.77 | 0 | 0 |  | O | HA2 |
| PHE497 | ASN349 | 3.98 | -1.54 | 0 | 0 |  | N | HD22 |
| ARG403 | VAL383 | 3.98 | -0.70 | 1 | 0 |  | HH21 | HG12 |
| GLN506 | THR320 | 4.02 | -0.28 | 0 | 0 |  | HE21 | HB |
| ASN439 | GLU321 | 4.06 | -0.56 | 0 | -1 |  | HD21 | HB1 |
| TYR453 | SER34 | 4.12 | -0.70 | 0 | 0 |  | HH | HB1 |
| ASN487 | PHE28 | 4.14 | -0.54 | 0 | 0 |  | OD1 | HB1 |
| PRO499 | GLU321 | 4.22 | 1.20 | 0 | -1 |  | O | O |
| TYR505 | GLY348 | 4.28 | 1.25 | 0 | 0 |  | HH | HA1 |
| GLY502 | ASN349 | 4.30 | -0.10 | 0 | 0 |  | H | O |
| THR500 | THR320 | 4.30 | 0.02 | 0 | 0 |  | O | HB |
| ASN501 | PHE41 | 4.31 | -0.32 | 0 | 0 |  | HD21 | HZ |
| ARG403 | ALA382 | 4.32 | -1.70 | 1 | 0 |  | HH22 | O |
| THR500 | PHE352 | 4.35 | -0.81 | 0 | 0 |  | O | H |
| GLN498 | LEU45 | 4.38 | -0.28 | 0 | 0 |  | HG1 | HD13 |
| TYR449 | ASN349 | 4.38 | -0.34 | 0 | 0 |  | HH | HD22 |
| TYR489 | TYR83 | 4.40 | -0.19 | 0 | 0 |  | HH | HH |
| ASN487 | GLN24 | 4.44 | -0.02 | 0 | 0 |  | HD22 | HB2 |
| ASN501 | GLU321 | 4.48 | -0.52 | 0 | -1 |  | O | HB1 |
| GLY502 | ASP351 | 4.49 | -0.14 | 0 | -1 |  | H | N |
| THR500 | PHE323 | 4.49 | -0.59 | 0 | 0 |  | O | H |
| PHE456 | LYS26 | 4.49 | -0.18 | 0 | 1 |  | HE1 | O |
| ASN487 | THR27 | 4.55 | -1.78 | 0 | 0 |  | OD1 | HG1 |
| GLN498 | ASP351 | 4.56 | -0.62 | 0 | -1 |  | HB1 | OD2 |
| PRO499 | GLY322 | 4.56 | 0.57 | 0 | 0 |  | O | HA1 |
| VAL503 | GLU321 | 4.67 | -0.41 | 0 | -1 |  | HG23 | H |
| THR500 | PHE41 | 4.67 | -0.16 | 0 | 0 |  | HG1 | HZ |
| GLY446 | PHE41 | 4.70 | -0.16 | 0 | 0 |  | O | HE1 |
| ARG403 | ARG389 | 4.70 | 1.98 | 1 | 1 |  | HH12 | HH22 |
| ARG403 | GLU37 | 4.72 | -2.57 | 1 | -1 |  | HH12 | OE1 |
| GLY502 | GLU321 | 4.73 | -0.10 | 0 | -1 |  | HA1 | H |
| LYS417 | THR27 | 4.75 | -0.24 | 1 | 0 |  | HZ1 | HG21 |
| CYS488 | LYS31 | 4.76 | -0.13 | 0 | 1 |  | O | HE1 |
| SER477 | GLN24 | 4.80 | -0.81 | 0 | 0 |  | H | OE1 |
| ASN487 | LEU79 | 4.83 | -0.41 | 0 | 0 |  | H | HD11 |
| ALA475 | PHE28 | 4.84 | -0.25 | 0 | 0 |  | HB1 | H |
| TYR449 | SER38 | 4.86 | -0.30 | 0 | 0 |  | HH | HA |
| ALA475 | GLU23 | 4.90 | -0.19 | 0 | -1 |  | HB1 | O |
| VAL503 | MET379 | 4.91 | -0.24 | 0 | 0 |  | HG21 | HE2 |
| GLY502 | MET379 | 4.92 | -0.16 | 0 | 0 |  | HA2 | HG1 |
| LEU455 | ASN33 | 4.93 | -0.35 | 0 | 0 |  | HD13 | HB1 |
| PHE456 | PHE28 | 5.02 | -0.16 | 0 | 0 |  | HZ | N |
| GLY504 | VAL383 | 5.05 | -0.10 | 0 | 0 |  | HA2 | HG12 |
| TYR489 | LEU79 | 5.06 | -0.15 | 0 | 0 |  | OH | HD11 |
| PHE486 | GLN76 | 5.06 | -0.31 | 0 | 0 |  | HD1 | HE22 |
| TYR473 | GLU23 | 5.08 | -0.37 | 0 | -1 |  | HH | O |
| GLN506 | GLY322 | 5.11 | -0.14 | 0 | 0 |  | HE21 | HA2 |
| VAL503 | PRO317 | 5.13 | -0.17 | 0 | 0 |  | HG22 | HB1 |
| GLY502 | GLY322 | 5.13 | 0.05 | 0 | 0 |  | HA1 | HA2 |
| ASP405 | ALA382 | 5.14 | -0.27 | -1 | 0 |  | OD2 | HB1 |
| LYS417 | LYS26 | 5.16 | 0.99 | 1 | 1 |  | HZ1 | O |
| PHE456 | PHE32 | 5.17 | -0.17 | 0 | 0 |  | HZ | H |
| ASN501 | PHE352 | 5.21 | -0.31 | 0 | 0 |  | HA | HD2 |
| TYR421 | ASP30 | 5.21 | -0.35 | 0 | -1 |  | HE2 | OD2 |
| PHE456 | SER34 | 5.22 | -0.26 | 0 | 0 |  | HE2 | HG |
| VAL503 | PHE352 | 5.36 | -0.16 | 0 | 0 |  | H | HE2 |
| TYR505 | ASP351 | 5.37 | -0.51 | 0 | -1 |  | HE1 | H |
| TYR495 | ASN349 | 5.39 | 0.03 | 0 | 0 |  | C | HD22 |
| GLY504 | PHE352 | 5.41 | -0.08 | 0 | 0 |  | H | HE2 |
| GLY446 | LEU45 | 5.45 | -0.14 | 0 | 0 |  | HA1 | HD12 |
| GLY447 | PHE41 | 5.45 | -0.09 | 0 | 0 |  | HA2 | HE1 |
| TYR489 | GLN76 | 5.50 | -0.19 | 0 | 0 |  | HE1 | HE22 |
| TYR489 | ASP30 | 5.51 | -0.42 | 0 | -1 |  | HE2 | HB1 |
| ASN487 | ALA82 | 5.52 | -0.08 | 0 | 0 |  | HD22 | HB3 |
| GLY504 | ALA382 | 5.53 | -0.07 | 0 | 0 |  | HA2 | HB1 |
| GLN506 | GLY350 | 5.57 | -0.15 | 0 | 0 |  | H | HA2 |
| PRO499 | ASN325 | 5.57 | -0.35 | 0 | 0 |  | O | HD21 |
| TYR489 | GLN24 | 5.58 | -0.33 | 0 | 0 |  | HH | O |
| GLY485 | LYS31 | 5.60 | -0.35 | 0 | 1 |  | O | HE1 |
| LEU455 | GLU35 | 5.61 | -0.21 | 0 | -1 |  | HD23 | H |
| PHE456 | LEU29 | 5.61 | -0.22 | 0 | 0 |  | HZ | C |
| ASN487 | LYS31 | 5.62 | -0.17 | 0 | 1 |  | HA | HG1 |
| GLY496 | PHE41 | 5.62 | -0.18 | 0 | 0 |  | HA2 | HE1 |
| SER494 | SER34 | 5.62 | -0.05 | 0 | 0 |  | H | HB1 |
| TYR473 | GLN24 | 5.62 | -0.13 | 0 | 0 |  | HH | HA |
| GLY496 | GLU37 | 5.63 | -0.15 | 0 | -1 |  | HA2 | HG1 |
| GLN493 | ASP30 | 5.63 | -0.02 | 0 | -1 |  | HG1 | O |
| LEU492 | LYS31 | 5.66 | -0.37 | 0 | 1 |  | O | HZ3 |
| THR500 | LEU45 | 5.67 | -0.11 | 0 | 0 |  | HG1 | HD23 |
| LEU455 | PHE32 | 5.68 | -0.21 | 0 | 0 |  | HD23 | N |
| GLY504 | GLY350 | 5.68 | -0.09 | 0 | 0 |  | H | HA2 |
| LEU455 | THR27 | 5.68 | -0.34 | 0 | 0 |  | O | HG21 |
| ARG457 | THR27 | 5.70 | -0.11 | 1 | 0 |  | H | HG21 |
| GLN498 | ASN326 | 5.72 | -0.10 | 0 | 0 |  | HB2 | HD22 |
| GLY504 | THR320 | 5.73 | -0.05 | 0 | 0 |  | H | HG21 |
| ASN501 | PHE323 | 5.74 | -0.10 | 0 | 0 |  | O | H |
| TYR489 | PHE32 | 5.74 | -0.29 | 0 | 0 |  | HE2 | H |
| PHE486 | GLU75 | 5.77 | -0.39 | 0 | -1 |  | HB1 | OE1 |
| ARG403 | ASN349 | 5.77 | -0.40 | 1 | 0 |  | HH12 | HA |
| GLY447 | ASN349 | 5.78 | -0.04 | 0 | 0 |  | HA1 | HD22 |
| GLY476 | THR27 | 5.78 | -0.08 | 0 | 0 |  | N | HG1 |
| GLN493 | GLU35 | 5.80 | -0.90 | 0 | -1 |  | HG1 | H |
| ALA475 | LYS31 | 5.83 | -0.01 | 0 | 1 |  | HB2 | HB1 |
| ASN501 | GLY348 | 5.86 | -0.10 | 0 | 0 |  | HD21 | O |
| TYR505 | VAL383 | 5.88 | -0.04 | 0 | 0 |  | HE1 | N |
| LYS417 | LYS31 | 5.89 | 0.57 | 1 | 1 |  | HZ1 | H |
| TYR473 | LYS31 | 5.89 | -0.22 | 0 | 1 |  | HD2 | HB1 |
| VAL503 | GLY350 | 5.93 | 0.02 | 0 | 0 |  | H | O |
| TYR505 | MET379 | 5.94 | -0.11 | 0 | 0 |  | HE1 | HA |
| ALA475 | TYR83 | 5.94 | -0.09 | 0 | 0 |  | HB3 | OH |
| GLY502 | PHE323 | 5.98 | -0.03 | 0 | 0 |  | HA1 | H |
| ASN501 | ASN326 | 5.99 | -0.18 | 0 | 0 |  | N | HD22 |
| GLN506 | LYS318 | 5.99 | -0.09 | 0 | 1 |  | HE22 | HE2 |
| PHE486 | ALA80 | 6.01 | -0.09 | 0 | 0 |  | HE1 | N |
| ASN437 | GLU321 | 6.02 | -0.08 | 0 | -1 |  | HD22 | HG2 |
| TYR489 | LEU29 | 6.02 | -0.17 | 0 | 0 |  | HE2 | N |
| THR500 | LEU347 | 6.04 | -0.11 | 0 | 0 |  | HG1 | HD23 |
| PRO491 | LYS31 | 6.05 | -0.59 | 0 | 1 |  | N | HZ3 |
| TYR505 | TYR386 | 6.05 | -0.07 | 0 | 0 |  | HH | HD2 |
| PRO499 | ASP351 | 6.07 | -2.59 | 0 | -1 |  | C | OD2 |
| PRO499 | ASN326 | 6.08 | 0.37 | 0 | 0 |  | HG1 | HD22 |
| LYS417 | SER34 | 6.09 | 0.06 | 1 | 0 |  | HE2 | HB2 |
| VAL445 | ASN326 | 6.11 | -0.09 | 0 | 0 |  | HG13 | HD22 |
| GLN498 | ARG353 | 6.11 | -0.03 | 0 | 1 |  | HG1 | HH12 |
| GLN493 | SER38 | 6.11 | -0.10 | 0 | 0 |  | HG2 | HG |
| TYR421 | THR27 | 6.16 | -0.07 | 0 | 0 |  | HH | HG21 |
| TYR505 | GLN384 | 6.16 | -0.05 | 0 | 0 |  | OH | O |
| LYS417 | ASN33 | 6.16 | 0.16 | 1 | 0 |  | HE2 | HB1 |
| ASN439 | GLY322 | 6.19 | -0.09 | 0 | 0 |  | HD22 | HA1 |
| VAL503 | MET319 | 6.21 | -0.07 | 0 | 0 |  | HG21 | C |
| ALA475 | ALA25 | 6.22 | -0.07 | 0 | 0 |  | HB1 | N |
| LEU455 | LEU29 | 6.22 | -0.06 | 0 | 0 |  | HD23 | O |
| THR500 | ASN349 | 6.22 | -0.22 | 0 | 0 |  | HG1 | HD21 |
| TYR453 | GLU37 | 6.24 | -0.05 | 0 | -1 |  | HH | HB2 |
| TYR473 | ASP30 | 6.27 | -0.04 | 0 | -1 |  | HE2 | HB1 |
| GLY496 | SER38 | 6.28 | -0.14 | 0 | 0 |  | HA2 | HA |
| TYR505 | ASP346 | 6.28 | -0.05 | 0 | -1 |  | HE1 | OD1 |
| GLU484 | GLU35 | 6.29 | 2.30 | -1 | -1 |  | OE1 | OE2 |
| TYR473 | PHE28 | 6.30 | -0.11 | 0 | 0 |  | HE2 | N |
| GLY476 | TYR83 | 6.30 | -0.05 | 0 | 0 |  | HA2 | HE2 |
| PHE456 | ASN33 | 6.30 | -0.09 | 0 | 0 |  | HZ | H |
| GLN506 | ASN349 | 6.31 | -0.10 | 0 | 0 |  | N | O |
| ASN487 | ALA25 | 6.35 | 0.08 | 0 | 0 |  | HD22 | HA |
| ARG403 | GLY350 | 6.39 | -0.12 | 1 | 0 |  | HH22 | HA2 |
| TYR505 | THR320 | 6.40 | -0.03 | 0 | 0 |  | HD1 | HG21 |
| PRO499 | THR320 | 6.40 | -0.22 | 0 | 0 |  | O | HB |
| ASN487 | GLN76 | 6.41 | -0.05 | 0 | 0 |  | H | HE22 |
| TYR505 | ASP378 | 6.41 | -0.02 | 0 | -1 |  | HE1 | O |
| TYR449 | GLU37 | 6.42 | -0.24 | 0 | -1 |  | HH | O |
| TYR489 | GLU35 | 6.42 | -0.22 | 0 | -1 |  | HD1 | OE2 |
| LYS458 | THR27 | 6.43 | -0.05 | 1 | 0 |  | HA | HG23 |
| GLN498 | SER38 | 6.45 | -0.05 | 0 | 0 |  | HE22 | HA |
| PHE486 | GLN24 | 6.45 | -0.27 | 0 | 0 |  | HZ | HB2 |
| PHE486 | LYS31 | 6.46 | -0.10 | 0 | 1 |  | HA | HG1 |
| GLN474 | GLN24 | 6.48 | -0.11 | 0 | 0 |  | O | HG1 |
| TYR489 | LYS26 | 6.49 | 0.00 | 0 | 1 |  | HE2 | C |
| GLN474 | THR27 | 6.49 | -0.01 | 0 | 0 |  | O | HG23 |
| ALA475 | PRO20 | 6.55 | -0.29 | 0 | 1 |  | O | O |
| GLY502 | ALA382 | 6.58 | 0.00 | 0 | 0 |  | HA2 | HB1 |
| ALA475 | LYS26 | 6.58 | -0.14 | 0 | 1 |  | HB1 | C |
| GLY485 | LEU79 | 6.63 | -0.10 | 0 | 0 |  | O | HD11 |
| VAL445 | ARG353 | 6.64 | 0.06 | 0 | 1 |  | HG13 | HH12 |
| THR500 | SER327 | 6.64 | -0.08 | 0 | 0 |  | HG22 | H |
| LYS417 | LEU29 | 6.65 | 0.52 | 1 | 0 |  | HZ1 | C |
| SER494 | ASN349 | 6.65 | -0.01 | 0 | 0 |  | O | OD1 |
| PRO499 | PHE41 | 6.65 | -0.08 | 0 | 0 |  | HD1 | HZ |
| TYR489 | SER34 | 6.67 | -0.08 | 0 | 0 |  | HB2 | HG |
| THR500 | TRP324 | 6.68 | -0.02 | 0 | 0 |  | HB | N |
| GLY504 | MET379 | 6.68 | -0.09 | 0 | 0 |  | H | HE2 |
| PHE456 | GLU35 | 6.69 | 0.00 | 0 | -1 |  | HE2 | HG1 |
| GLN506 | PHE323 | 6.71 | -0.02 | 0 | 0 |  | HE21 | H |
| ARG403 | GLN384 | 6.74 | -0.33 | 1 | 0 |  | HH22 | H |
| GLN493 | ASN33 | 6.75 | -0.04 | 0 | 0 |  | HG1 | C |
| ASN439 | THR320 | 6.80 | -0.01 | 0 | 0 |  | HD22 | HB |
| TYR421 | LYS26 | 6.81 | -0.05 | 0 | 1 |  | HH | HB2 |
| GLN498 | GLY350 | 6.82 | -0.06 | 0 | 0 |  | HB1 | C |
| VAL503 | GLY322 | 6.83 | -0.03 | 0 | 0 |  | HG21 | H |
| GLY496 | GLY350 | 6.83 | 0.00 | 0 | 0 |  | O | HA2 |
| GLY476 | PRO20 | 6.84 | -0.04 | 0 | 1 |  | HA1 | O |
| PHE497 | PHE41 | 6.84 | -0.09 | 0 | 0 |  | C | HZ |
| ASN448 | ASN349 | 6.85 | -0.03 | 0 | 0 |  | H | HD22 |
| TYR473 | LYS26 | 6.85 | -0.04 | 0 | 1 |  | HE2 | C |
| SER494 | SER38 | 6.86 | -0.09 | 0 | 0 |  | O | OG |
| ASN439 | ASN325 | 6.87 | -0.01 | 0 | 0 |  | HD22 | HD21 |
| GLY485 | GLN76 | 6.89 | -0.03 | 0 | 0 |  | O | HE22 |
| GLN498 | GLY348 | 6.92 | -0.10 | 0 | 0 |  | OE1 | O |
| GLY502 | MET319 | 6.93 | 0.11 | 0 | 0 |  | HA1 | C |
| GLY446 | ASN349 | 6.94 | -0.01 | 0 | 0 |  | O | HD22 |
| ASN501 | ARG353 | 6.94 | 0.00 | 0 | 1 |  | HD21 | HH11 |
| ILE472 | LYS31 | 6.94 | 0.20 | 0 | 1 |  | HG23 | HE1 |
| ARG408 | ALA382 | 6.99 | 0.07 | 1 | 0 |  | HH12 | HB3 |
| TYR489 | ALA25 | 6.99 | -0.09 | 0 | 0 |  | HH | HA |
| LEU455 | PHE28 | 7.00 | -0.07 | 0 | 0 |  | HD23 | O |
| TYR449 | LEU45 | 7.02 | -0.03 | 0 | 0 |  | HH | HD13 |
| PHE486 | ARG78 | 7.04 | -0.04 | 0 | 1 |  | HZ | O |
| GLY476 | THR21 | 7.05 | -0.02 | 0 | 0 |  | HA1 | HA |
| ARG457 | ASP30 | 7.06 | -0.66 | 1 | -1 |  | H | OD2 |
| ARG403 | PHE352 | 7.08 | -0.10 | 1 | 0 |  | HH22 | HZ |
| GLY476 | GLU23 | 7.08 | 0.05 | 0 | -1 |  | HA1 | O |
| ALA475 | ASP30 | 7.09 | 0.01 | 0 | -1 |  | HB2 | HB1 |
| GLY502 | GLY348 | 7.09 | -0.01 | 0 | 0 |  | H | O |
| TYR421 | GLU23 | 7.10 | -0.03 | 0 | -1 |  | HH | OE1 |
| GLN498 | GLY322 | 7.11 | -0.08 | 0 | 0 |  | HB2 | HA1 |
| VAL445 | LEU45 | 7.11 | -0.08 | 0 | 0 |  | HG12 | HD12 |
| TYR495 | GLU37 | 7.12 | -0.16 | 0 | -1 |  | HA | HG1 |
| TYR453 | ASN33 | 7.12 | -0.12 | 0 | 0 |  | HH | O |
| SER494 | LYS31 | 7.15 | -0.09 | 0 | 1 |  | H | HZ2 |
| GLY485 | GLU75 | 7.16 | -0.16 | 0 | -1 |  | HA1 | OE1 |
| TYR453 | ASN349 | 7.16 | -0.10 | 0 | 0 |  | HH | OD1 |
| CYS488 | PHE28 | 7.18 | -0.07 | 0 | 0 |  | H | HD1 |
| PHE456 | GLU23 | 7.18 | -0.19 | 0 | -1 |  | HE1 | O |
| LEU455 | LYS26 | 7.20 | -0.10 | 0 | 1 |  | HG | O |
| GLY504 | ASN349 | 7.21 | -0.09 | 0 | 0 |  | C | O |
| TYR505 | ASN33 | 7.23 | -0.06 | 0 | 0 |  | HH | O |
| TYR505 | TYR381 | 7.23 | -0.04 | 0 | 0 |  | HE1 | C |
| TYR505 | PHE41 | 7.24 | -0.08 | 0 | 0 |  | HH | HB1 |
| GLY502 | LYS318 | 7.26 | -0.01 | 0 | 1 |  | HA1 | O |
| LEU492 | SER34 | 7.28 | -0.06 | 0 | 0 |  | C | HB1 |
| ASN501 | ASN325 | 7.28 | -0.12 | 0 | 0 |  | O | HD21 |
| PHE486 | ALA25 | 7.30 | -0.07 | 0 | 0 |  | HE1 | HA |
| THR500 | GLY348 | 7.31 | -0.02 | 0 | 0 |  | HG1 | O |
| ARG403 | PRO385 | 7.31 | -0.09 | 1 | 0 |  | HH12 | HA |
| THR478 | GLN24 | 7.31 | -0.07 | 0 | 0 |  | H | OE1 |
| ASN437 | LYS318 | 7.32 | 0.05 | 0 | 1 |  | HD22 | HZ1 |
| TYR449 | GLU42 | 7.32 | -0.07 | 0 | -1 |  | OH | HG2 |
| GLN506 | PHE352 | 7.34 | -0.05 | 0 | 0 |  | H | HE2 |
| PRO499 | ASN349 | 7.35 | -0.05 | 0 | 0 |  | N | HD21 |
| PRO499 | PHE323 | 7.35 | -0.03 | 0 | 0 |  | O | H |
| GLN506 | ASN325 | 7.36 | 0.03 | 0 | 0 |  | HE21 | HD21 |
| CYS488 | LEU79 | 7.36 | -0.02 | 0 | 0 |  | H | HD11 |
| ARG408 | ARG554 | 7.37 | 1.11 | 1 | 1 |  | HH11 | HD1 |
| GLY446 | ARG353 | 7.37 | 0.06 | 0 | 1 |  | H | HH12 |
| VAL445 | PHE41 | 7.37 | -0.04 | 0 | 0 |  | HA | HZ |
| PHE490 | SER34 | 7.38 | -0.06 | 0 | 0 |  | O | OG |
| ALA475 | THR21 | 7.38 | 0.02 | 0 | 0 |  | O | HA |
| GLN498 | LEU347 | 7.39 | -0.05 | 0 | 0 |  | HG1 | HD23 |
| TYR453 | ASP30 | 7.40 | 0.01 | 0 | -1 |  | OH | O |
| TYR505 | PRO385 | 7.41 | -0.01 | 0 | 0 |  | OH | HA |
| SER494 | GLU37 | 7.41 | 0.01 | 0 | -1 |  | O | HB2 |
| CYS488 | THR27 | 7.42 | -0.08 | 0 | 0 |  | N | HB |
| VAL445 | ASN325 | 7.44 | -0.03 | 0 | 0 |  | HG23 | HB2 |
| ALA475 | LEU29 | 7.45 | -0.03 | 0 | 0 |  | HB1 | H |
| GLN498 | GLU37 | 7.46 | 0.09 | 0 | -1 |  | HE22 | O |
| GLY485 | PHE28 | 7.46 | -0.01 | 0 | 0 |  | O | HD1 |
| GLY496 | GLY348 | 7.46 | -0.14 | 0 | 0 |  | O | O |
| TYR505 | LEU347 | 7.48 | -0.02 | 0 | 0 |  | HH | C |
| ASP405 | MET379 | 7.50 | 0.21 | -1 | 0 |  | OD1 | O |
| GLU406 | VAL383 | 7.50 | 0.00 | -1 | 0 |  | HG2 | HG12 |
| ASN501 | GLU37 | 7.50 | -0.15 | 0 | -1 |  | HD22 | HG1 |
| GLY446 | ASN326 | 7.51 | 0.00 | 0 | 0 |  | H | HD22 |
| TYR505 | SER34 | 7.51 | -0.03 | 0 | 0 |  | HE2 | HA |
| GLN493 | GLU37 | 7.51 | -0.09 | 0 | -1 |  | HG1 | HB2 |
| GLY447 | LEU45 | 7.53 | -0.03 | 0 | 0 |  | N | HD13 |
| ASN487 | LEU29 | 7.53 | -0.01 | 0 | 0 |  | OD1 | H |
| GLY496 | ASP351 | 7.53 | 0.10 | 0 | -1 |  | O | HB1 |
| GLN506 | MET319 | 7.54 | -0.01 | 0 | 0 |  | HE22 | O |
| TYR495 | SER34 | 7.54 | -0.02 | 0 | 0 |  | HA | HB1 |
| ASN501 | LEU347 | 7.55 | -0.03 | 0 | 0 |  | HD21 | HB1 |
| TYR489 | GLU23 | 7.56 | -0.03 | 0 | -1 |  | HE2 | O |
| ARG403 | ASN33 | 7.57 | -0.15 | 1 | 0 |  | HH12 | HB2 |
| PHE456 | ALA25 | 7.57 | -0.02 | 0 | 0 |  | HE1 | O |
| GLN506 | ASP351 | 7.58 | -0.13 | 0 | -1 |  | HE21 | HA |
| GLY502 | PHE41 | 7.58 | -0.02 | 0 | 0 |  | H | HE2 |
| ARG403 | SER34 | 7.59 | -0.09 | 1 | 0 |  | HH11 | HA |
| ASP405 | GLN384 | 7.61 | 0.12 | -1 | 0 |  | OD2 | H |
| VAL503 | PHE323 | 7.61 | -0.02 | 0 | 0 |  | H | H |
| TYR453 | SER38 | 7.62 | -0.03 | 0 | 0 |  | HH | HG |
| PRO499 | ARG353 | 7.63 | 0.23 | 0 | 1 |  | HD1 | HH12 |
| PHE497 | GLY350 | 7.64 | -0.04 | 0 | 0 |  | HA | HA2 |
| PHE486 | LYS81 | 7.67 | 0.01 | 0 | 1 |  | HZ | C |
| PRO499 | GLY350 | 7.69 | -0.01 | 0 | 0 |  | C | O |
| CYS488 | TYR83 | 7.69 | -0.02 | 0 | 0 |  | H | HH |
| PHE486 | THR27 | 7.70 | -0.01 | 0 | 0 |  | HE1 | HG1 |
| GLN409 | VAL383 | 7.70 | -0.05 | 0 | 0 |  | HE21 | HB |
| ASP405 | GLY350 | 7.70 | 0.03 | -1 | 0 |  | OD2 | HA2 |
| ASN487 | GLU23 | 7.72 | 0.00 | 0 | -1 |  | OD1 | O |
| ASN501 | MET379 | 7.73 | -0.03 | 0 | 0 |  | C | HG1 |
| TYR489 | GLU75 | 7.74 | 0.04 | 0 | -1 |  | HE1 | OE1 |
| VAL445 | ASP351 | 7.74 | -0.13 | 0 | -1 |  | HG13 | OD2 |
| ASN448 | PHE41 | 7.74 | -0.03 | 0 | 0 |  | H | HE1 |
| TYR453 | GLU35 | 7.76 | -0.03 | 0 | -1 |  | HH | N |
| ARG403 | TYR381 | 7.76 | -0.08 | 1 | 0 |  | HH22 | O |
| ASN487 | ALA80 | 7.76 | -0.02 | 0 | 0 |  | HD22 | HA |
| SER443 | ASN349 | 7.76 | -0.01 | 0 | 0 |  | HB1 | HD22 |
| ASN501 | LEU45 | 7.76 | -0.01 | 0 | 0 |  | HD21 | HD13 |
| ASP405 | PHE352 | 7.77 | 0.03 | -1 | 0 |  | OD2 | HE2 |
| VAL503 | ALA382 | 7.78 | -0.01 | 0 | 0 |  | N | HB1 |
| ASP405 | ALA380 | 7.81 | 0.29 | -1 | 0 |  | OD1 | O |
| PHE490 | GLU35 | 7.82 | 0.09 | 0 | -1 |  | H | OE2 |
| GLY404 | VAL383 | 7.82 | -0.05 | 0 | 0 |  | C | HG12 |
| PHE456 | GLN24 | 7.83 | -0.04 | 0 | 0 |  | HE1 | O |
| GLY446 | ASP351 | 7.83 | -0.15 | 0 | -1 |  | H | OD2 |
| ARG408 | ALA380 | 7.83 | -0.25 | 1 | 0 |  | HH12 | O |
| LYS417 | PHE28 | 7.84 | -0.12 | 1 | 0 |  | HZ1 | N |
| GLN498 | GLU321 | 7.84 | -0.08 | 0 | -1 |  | O | HB1 |
| TYR449 | SER34 | 7.84 | -0.04 | 0 | 0 |  | HE1 | O |
| PRO507 | ASN349 | 7.84 | -0.03 | 0 | 0 |  | HD2 | HD22 |
| SER443 | GLU321 | 7.87 | 0.01 | 0 | -1 |  | HG | HB1 |
| ASN440 | GLU321 | 7.87 | -0.02 | 0 | -1 |  | HB2 | HG2 |
| LYS417 | PRO385 | 7.88 | 0.06 | 1 | 0 |  | HE1 | HB2 |
| ASN487 | GLU75 | 7.89 | -0.10 | 0 | -1 |  | H | OE1 |
| GLN493 | PHE32 | 7.90 | -0.09 | 0 | 0 |  | HG1 | N |
| TYR505 | SER40 | 7.91 | -0.01 | 0 | 0 |  | HH | HG |
| SER477 | TYR83 | 7.93 | -0.03 | 0 | 0 |  | H | HE2 |
| LYS444 | PHE41 | 7.93 | -0.04 | 1 | 0 |  | O | HZ |
| ASN487 | THR21 | 7.94 | -0.02 | 0 | 0 |  | HD22 | O |
| ASN501 | MET319 | 7.97 | -0.02 | 0 | 0 |  | O | C |
| TYR449 | LEU39 | 7.97 | -0.02 | 0 | 0 |  | HH | N |

**Supplementary** **table 21**. Interactions between SARS-CoV-2 RBD e *Saccopteryx bilineata* ACE2.

| **Interaction** | **SARS-CoV-2  RBD** | ***Saccopteryx bilineata*  ACE2** |
| --- | --- | --- |
|  | Residue | Residue |
| Hydrophobic | TYR489 | PHE28 |
|  | TYR453 | TYR34 |
|  | LEU455 | TYR34 |
| Hydrogen bonds | GLY502 | LYS341 |
|  | SER494 | GLN38 |
|  | GLY496 | LYS341 |
|  | GLY496 | GLN38 |
|  | TYR489 | ARG31 |
|  | GLN493 | GLU35 |
|  | GLN498 | LYS341 |
|  | TYR505 | ARG381 |
|  | LYS417 | ASP30 |
|  | TYR449 | GLU42 |
|  | GLN493 | GLU35 |
|  | TYR505 | GLU37 |
| Ionic | LYS417 | ASP30 |
| Aromatic-Aromatic | TYR489 | PHE28 |
|  | TYR453 | TYR34 |
|  | PHE486 | PHE83 |
| Cation-Pi | PHE456 | ARG31 |
|  | TYR489 | ARG31 |

**Supplementary** **table 22**. Quantum biochemistry between SARS-CoV-2 RBD e *Saccopteryx bilineata* ACE2.

| **Amino acid residues** | | **Distance** | **Interaction energy** | **Residue charge** | |  | **Residue atom** | |
| --- | --- | --- | --- | --- | --- | --- | --- | --- |
| SARS-CoV-2  RBD | *Saccopteryx bilineata* ACE2 | (Å) | (kcal mol-1) | SARS-CoV-2  RBD | *Saccopteryx bilineata* ACE2 |  | SARS-CoV-2  RBD | *Saccopteryx bilineata* ACE2 |
| LYS417 | ASP30 | 1.56 | -8.12 | 1 | -1 |  | HZ1 | OD2 |
| GLN493 | GLU35 | 1.71 | -6.62 | 0 | -1 |  | HE21 | OE2 |
| TYR505 | GLU37 | 1.75 | -9.53 | 0 | -1 |  | HH | OE1 |
| SER494 | GLN38 | 1.90 | -2.34 | 0 | 0 |  | O | HE22 |
| GLN498 | LYS341 | 1.90 | -6.85 | 0 | 1 |  | OE1 | HZ2 |
| GLY502 | LYS341 | 2.00 | -4.50 | 0 | 1 |  | H | O |
| TYR505 | ARG381 | 2.04 | 1.82 | 0 | 1 |  | HH | HH22 |
| PHE486 | ARG79 | 2.17 | -2.93 | 0 | 1 |  | HD1 | HG1 |
| PHE456 | ARG31 | 2.18 | -3.14 | 0 | 1 |  | CE1 | HH21 |
| LEU455 | ARG31 | 2.22 | -2.69 | 0 | 1 |  | HD21 | HA |
| TYR495 | GLN38 | 2.22 | -1.02 | 0 | 0 |  | HA | HE22 |
| PHE486 | ASP82 | 2.22 | -2.87 | 0 | -1 |  | HZ | HB2 |
| LEU455 | ASP30 | 2.29 | -1.80 | 0 | -1 |  | HD13 | HB2 |
| GLY496 | LYS341 | 2.35 | -3.07 | 0 | 1 |  | O | HZ3 |
| ASN501 | LYS341 | 2.37 | -12.28 | 0 | 1 |  | HD22 | HD1 |
| GLN498 | PHE41 | 2.37 | -1.95 | 0 | 0 |  | HG1 | HE1 |
| GLY502 | ASN342 | 2.44 | -2.15 | 0 | 0 |  | HA2 | HB1 |
| TYR453 | TYR34 | 2.47 | -3.06 | 0 | 0 |  | OH | HB2 |
| ASN487 | PHE83 | 2.47 | -1.69 | 0 | 0 |  | OD1 | HZ |
| ARG403 | TYR34 | 2.47 | -0.99 | 1 | 0 |  | HH11 | HE1 |
| TYR505 | ASN342 | 2.47 | -2.62 | 0 | 0 |  | HD1 | HD21 |
| TYR489 | PHE28 | 2.49 | -1.79 | 0 | 0 |  | OH | HD1 |
| TYR449 | GLN38 | 2.50 | -1.98 | 0 | 0 |  | HE1 | OE1 |
| THR500 | PHE41 | 2.52 | -1.09 | 0 | 0 |  | HG1 | HZ |
| TYR505 | LYS341 | 2.55 | -7.38 | 0 | 1 |  | HB2 | HB2 |
| LEU455 | TYR34 | 2.62 | -2.64 | 0 | 0 |  | HD13 | HD2 |
| ASN501 | PHE41 | 2.66 | -2.03 | 0 | 0 |  | HD22 | HE1 |
| THR500 | ASN318 | 2.69 | -1.66 | 0 | 0 |  | HB | HD22 |
| TYR489 | ARG31 | 2.71 | -5.75 | 0 | 1 |  | CE1 | HD1 |
| GLY496 | GLN38 | 2.73 | -1.48 | 0 | 0 |  | H | HE22 |
| TYR449 | GLU42 | 2.87 | -1.31 | 0 | -1 |  | HH | OE2 |
| TYR489 | ARG79 | 2.90 | -2.98 | 0 | 1 |  | HE1 | HH21 |
| TYR489 | PHE83 | 2.95 | -0.07 | 0 | 0 |  | HH | HZ |
| THR500 | ASP343 | 2.96 | -3.42 | 0 | -1 |  | HB | OD2 |
| ALA475 | GLU24 | 3.03 | -1.01 | 0 | -1 |  | O | HG1 |
| LYS417 | THR27 | 3.06 | -0.09 | 1 | 0 |  | HZ1 | HG23 |
| GLY485 | ARG79 | 3.06 | -1.86 | 0 | 1 |  | O | HH22 |
| ALA475 | ARG31 | 3.13 | -1.18 | 0 | 1 |  | HB1 | HH12 |
| ASN487 | ARG79 | 3.20 | -0.46 | 0 | 1 |  | H | HH21 |
| GLN498 | LEU45 | 3.21 | -0.63 | 0 | 0 |  | HG1 | HD13 |
| ASN501 | ASP343 | 3.29 | -3.15 | 0 | -1 |  | HA | HB1 |
| TYR489 | ARG76 | 3.45 | -0.82 | 0 | 1 |  | HE1 | HH22 |
| TYR453 | GLN38 | 3.45 | -0.38 | 0 | 0 |  | HE1 | HE22 |
| LYS417 | TYR34 | 3.47 | -1.99 | 1 | 0 |  | HE1 | HE2 |
| THR500 | ARG345 | 3.48 | -0.06 | 0 | 1 |  | HB | HH11 |
| TYR473 | ARG31 | 3.65 | -1.24 | 0 | 1 |  | HE2 | HH22 |
| PHE456 | THR27 | 3.71 | -0.77 | 0 | 0 |  | HD1 | HB |
| LEU455 | GLU35 | 3.74 | -0.73 | 0 | -1 |  | HD22 | HG2 |
| TYR453 | GLU35 | 3.90 | -0.69 | 0 | -1 |  | HH | HG1 |
| GLN493 | TYR34 | 4.09 | -0.37 | 0 | 0 |  | HG1 | HB1 |
| ASN487 | PHE28 | 4.10 | -0.48 | 0 | 0 |  | OD1 | HD1 |
| ALA475 | PHE83 | 4.12 | -0.30 | 0 | 0 |  | HB3 | HZ |
| ASN487 | ARG31 | 4.13 | -0.68 | 0 | 1 |  | OD1 | HH11 |
| PHE456 | ASP30 | 4.15 | -0.55 | 0 | -1 |  | HE1 | HB1 |
| PHE497 | LYS341 | 4.16 | -1.82 | 0 | 1 |  | HA | HZ3 |
| PHE486 | PHE83 | 4.22 | -1.61 | 0 | 0 |  | HE1 | HE2 |
| CYS488 | ARG79 | 4.23 | -1.16 | 0 | 1 |  | H | HH22 |
| GLU484 | ARG79 | 4.27 | -1.98 | -1 | 1 |  | HB2 | HH12 |
| GLY504 | ASN342 | 4.33 | -0.34 | 0 | 0 |  | H | HB1 |
| GLY502 | ASP343 | 4.37 | -0.39 | 0 | -1 |  | H | N |
| PHE486 | ALA80 | 4.38 | -0.44 | 0 | 0 |  | HE1 | N |
| GLN493 | ARG76 | 4.39 | 0.34 | 0 | 1 |  | HE21 | HH12 |
| GLU406 | TYR34 | 4.40 | -0.43 | -1 | 0 |  | OE2 | HE1 |
| TYR505 | TYR34 | 4.42 | -0.59 | 0 | 0 |  | HE2 | HD1 |
| THR500 | LEU45 | 4.47 | -0.37 | 0 | 0 |  | OG1 | HD22 |
| ASN487 | GLU24 | 4.48 | -0.38 | 0 | -1 |  | HD22 | HB2 |
| TYR449 | LYS341 | 4.55 | -0.14 | 0 | 1 |  | HE1 | HZ1 |
| TYR473 | THR27 | 4.56 | -0.44 | 0 | 0 |  | HH | HG22 |
| VAL503 | ASN342 | 4.60 | -0.34 | 0 | 0 |  | N | HB1 |
| ASN501 | ASN342 | 4.64 | -2.01 | 0 | 0 |  | C | HB1 |
| TYR421 | THR27 | 4.65 | -0.28 | 0 | 0 |  | OH | HG22 |
| GLY446 | LEU45 | 4.66 | -0.09 | 0 | 0 |  | H | HD13 |
| GLY476 | GLU24 | 4.73 | -0.17 | 0 | -1 |  | HA1 | HG1 |
| GLN498 | GLN38 | 4.77 | -0.56 | 0 | 0 |  | HE22 | HA |
| THR500 | GLY314 | 4.81 | -0.35 | 0 | 0 |  | O | HA2 |
| ARG408 | GLU375 | 4.82 | -1.94 | 1 | -1 |  | HH12 | HG2 |
| PHE486 | MET78 | 4.83 | -0.05 | 0 | 0 |  | HE1 | O |
| TYR505 | ALA374 | 4.84 | -0.15 | 0 | 0 |  | HE1 | O |
| GLN493 | ARG31 | 4.86 | -0.61 | 0 | 1 |  | HG1 | HA |
| GLN409 | TYR34 | 4.89 | -0.06 | 0 | 0 |  | HE22 | HH |
| VAL445 | LEU45 | 4.92 | -0.19 | 0 | 0 |  | HG13 | HD13 |
| ASN501 | GLY340 | 4.95 | -0.04 | 0 | 0 |  | HD21 | O |
| LEU455 | THR27 | 4.97 | -0.50 | 0 | 0 |  | HD21 | O |
| GLN498 | GLU42 | 4.98 | -1.33 | 0 | -1 |  | HE22 | HB1 |
| LYS417 | LYS26 | 5.05 | 1.28 | 1 | 1 |  | HZ1 | O |
| GLN493 | GLN38 | 5.07 | -0.10 | 0 | 0 |  | HB2 | HE22 |
| TYR449 | LEU39 | 5.08 | -0.05 | 0 | 0 |  | HH | HD21 |
| ASN487 | ASP82 | 5.09 | -0.57 | 0 | -1 |  | HD22 | HB2 |
| GLY496 | PHE41 | 5.12 | -0.19 | 0 | 0 |  | O | HD1 |
| GLY446 | GLU42 | 5.16 | 0.17 | 0 | -1 |  | O | HG2 |
| PHE456 | PHE28 | 5.18 | -0.12 | 0 | 0 |  | HE1 | HA |
| GLY502 | THR312 | 5.19 | -0.02 | 0 | 0 |  | HA2 | HG21 |
| TYR495 | LYS341 | 5.26 | 0.99 | 0 | 1 |  | C | HZ3 |
| TYR473 | GLU24 | 5.27 | -0.29 | 0 | -1 |  | HH | HG2 |
| PHE456 | GLU35 | 5.28 | -0.07 | 0 | -1 |  | HZ | HG2 |
| TYR489 | GLU35 | 5.29 | -0.30 | 0 | -1 |  | HD1 | OE1 |
| TYR449 | LYS68 | 5.31 | -0.07 | 0 | 1 |  | HH | HZ1 |
| THR500 | LYS317 | 5.32 | -0.30 | 0 | 1 |  | HG22 | HB2 |
| GLY447 | LYS341 | 5.39 | 0.11 | 0 | 1 |  | HA2 | HZ2 |
| SER494 | GLU35 | 5.42 | -0.14 | 0 | -1 |  | H | OE2 |
| ASN501 | ARG345 | 5.43 | 0.02 | 0 | 1 |  | HD21 | HH11 |
| TYR505 | ASN33 | 5.46 | -0.22 | 0 | 0 |  | HH | O |
| GLY502 | GLY340 | 5.46 | 0.09 | 0 | 0 |  | H | O |
| LEU455 | PHE32 | 5.47 | -0.18 | 0 | 0 |  | HD21 | N |
| ARG403 | ARG381 | 5.48 | 1.56 | 1 | 1 |  | HH22 | HH21 |
| ALA475 | THR27 | 5.49 | -0.13 | 0 | 0 |  | HB3 | OG1 |
| THR500 | ASN342 | 5.49 | -0.17 | 0 | 0 |  | O | O |
| LEU455 | ARG76 | 5.50 | -0.02 | 0 | 1 |  | HD21 | HH11 |
| GLY502 | PHE344 | 5.50 | -0.12 | 0 | 0 |  | HA2 | HE2 |
| PRO499 | PHE41 | 5.50 | -0.07 | 0 | 0 |  | HD1 | HE1 |
| ALA475 | PHE28 | 5.51 | -0.14 | 0 | 0 |  | HB3 | HB1 |
| ASN501 | LEU45 | 5.51 | -0.13 | 0 | 0 |  | HD22 | HD13 |
| TYR421 | ASP30 | 5.51 | -0.04 | 0 | -1 |  | HE2 | OD2 |
| PHE486 | PHE28 | 5.52 | -0.29 | 0 | 0 |  | HE1 | HE1 |
| ARG403 | GLU37 | 5.52 | -2.02 | 1 | -1 |  | HH12 | OE1 |
| TYR505 | GLN38 | 5.53 | -0.29 | 0 | 0 |  | HE2 | HG1 |
| ASN501 | ASN318 | 5.55 | -0.23 | 0 | 0 |  | N | HD22 |
| TYR505 | GLU375 | 5.57 | -0.05 | 0 | -1 |  | HE1 | HA |
| ASP405 | ASN342 | 5.59 | -0.16 | -1 | 0 |  | OD2 | HD22 |
| THR500 | LYS341 | 5.64 | -0.15 | 0 | 1 |  | HG1 | HB1 |
| PHE486 | LYS81 | 5.65 | -0.07 | 0 | 1 |  | HE1 | H |
| GLY504 | LYS341 | 5.67 | -0.23 | 0 | 1 |  | H | O |
| GLY446 | PHE41 | 5.69 | -0.08 | 0 | 0 |  | H | HE1 |
| VAL503 | THR312 | 5.74 | -0.06 | 0 | 0 |  | HG21 | HG22 |
| PHE497 | GLN38 | 5.76 | -0.24 | 0 | 0 |  | H | HE22 |
| ARG457 | THR27 | 5.77 | -0.09 | 1 | 0 |  | H | HG22 |
| GLY476 | PHE83 | 5.79 | -0.09 | 0 | 0 |  | HA1 | HE1 |
| LYS417 | ARG31 | 5.79 | 1.08 | 1 | 1 |  | HE2 | H |
| GLN506 | LYS341 | 5.80 | -0.52 | 0 | 1 |  | H | O |
| TYR489 | GLU24 | 5.80 | -0.15 | 0 | -1 |  | HH | O |
| VAL503 | LYS341 | 5.81 | 0.11 | 0 | 1 |  | N | O |
| ARG403 | ASN342 | 5.84 | 0.04 | 1 | 0 |  | HH22 | HD21 |
| TYR505 | TYR378 | 5.87 | -0.08 | 0 | 0 |  | HH | HB1 |
| VAL445 | PHE41 | 5.88 | -0.06 | 0 | 0 |  | HA | HE1 |
| PHE456 | ARG76 | 5.90 | -0.10 | 0 | 1 |  | HZ | HH12 |
| GLN506 | ASN342 | 5.91 | -0.20 | 0 | 0 |  | H | HB1 |
| SER494 | TYR34 | 5.92 | 0.10 | 0 | 0 |  | O | O |
| GLY447 | PHE41 | 5.96 | -0.07 | 0 | 0 |  | HA1 | HE1 |
| ARG403 | LYS341 | 5.98 | 1.06 | 1 | 1 |  | HD1 | HD2 |
| LEU455 | ASN33 | 5.98 | -0.18 | 0 | 0 |  | HD13 | HB1 |
| TYR495 | TYR34 | 5.99 | -0.24 | 0 | 0 |  | HE1 | HD1 |
| PRO499 | ASN318 | 5.99 | -0.02 | 0 | 0 |  | O | HD22 |
| VAL503 | GLU313 | 6.00 | -0.09 | 0 | -1 |  | HG21 | HB1 |
| THR500 | PHE344 | 6.00 | -0.18 | 0 | 0 |  | O | H |
| ARG403 | GLN38 | 6.02 | 0.07 | 1 | 0 |  | HD1 | HE21 |
| TYR505 | GLY340 | 6.03 | -0.20 | 0 | 0 |  | HH | HA1 |
| THR500 | LEU339 | 6.04 | -0.14 | 0 | 0 |  | HG1 | HD23 |
| TYR505 | PRO377 | 6.05 | -0.04 | 0 | 0 |  | HH | HA |
| ASP405 | GLU375 | 6.05 | 1.23 | -1 | -1 |  | OD2 | HG2 |
| ARG403 | GLU375 | 6.05 | -1.31 | 1 | -1 |  | HH22 | HA |
| GLN493 | ASP30 | 6.06 | -0.06 | 0 | -1 |  | HG1 | O |
| GLU484 | ARG76 | 6.08 | -1.66 | -1 | 1 |  | OE1 | HH22 |
| TYR453 | ASP30 | 6.08 | -0.05 | 0 | -1 |  | HE2 | HB2 |
| GLY502 | GLY314 | 6.11 | 0.00 | 0 | 0 |  | HA1 | HA2 |
| PHE486 | ASP75 | 6.13 | -0.31 | 0 | -1 |  | HD1 | O |
| LEU455 | PHE28 | 6.16 | -0.13 | 0 | 0 |  | HD21 | HA |
| PHE497 | PHE41 | 6.18 | -0.09 | 0 | 0 |  | C | HE1 |
| ASN501 | GLY314 | 6.19 | -0.04 | 0 | 0 |  | O | HA2 |
| TYR505 | ASP343 | 6.20 | -0.23 | 0 | -1 |  | HB1 | H |
| TYR449 | PHE41 | 6.20 | -0.11 | 0 | 0 |  | OH | HD1 |
| TYR451 | GLN38 | 6.22 | -0.06 | 0 | 0 |  | O | HE22 |
| PRO491 | ARG31 | 6.23 | -0.15 | 0 | 1 |  | HA | HD2 |
| PRO499 | LEU45 | 6.25 | -0.10 | 0 | 0 |  | HD1 | HD13 |
| ASP405 | TYR34 | 6.27 | -0.09 | -1 | 0 |  | HB1 | HE1 |
| PHE456 | TYR34 | 6.28 | -0.18 | 0 | 0 |  | HE1 | HD2 |
| GLY447 | GLU42 | 6.30 | 0.18 | 0 | -1 |  | HA2 | OE2 |
| TYR505 | GLN376 | 6.31 | -0.01 | 0 | 0 |  | HH | O |
| GLN493 | PHE72 | 6.31 | -0.05 | 0 | 0 |  | HE21 | HE1 |
| PHE490 | ARG31 | 6.36 | -0.30 | 0 | 1 |  | O | HD2 |
| GLU484 | GLU35 | 6.38 | 1.89 | -1 | -1 |  | OE1 | OE1 |
| PHE486 | ARG76 | 6.38 | -0.10 | 0 | 1 |  | HE1 | O |
| LEU492 | GLU35 | 6.38 | -0.12 | 0 | -1 |  | O | OE2 |
| TYR489 | THR27 | 6.43 | -0.20 | 0 | 0 |  | HE2 | HB |
| TYR473 | ASN23 | 6.44 | 0.00 | 0 | 0 |  | HH | HB2 |
| TYR453 | ARG31 | 6.44 | -0.09 | 0 | 1 |  | HE2 | HA |
| ILE418 | TYR34 | 6.45 | -0.17 | 0 | 0 |  | HG11 | HH |
| GLY447 | GLN38 | 6.48 | -0.04 | 0 | 0 |  | HA2 | OE1 |
| ASN501 | LEU339 | 6.49 | -0.08 | 0 | 0 |  | HD21 | HB1 |
| ARG403 | ALA374 | 6.49 | -0.37 | 1 | 0 |  | HH22 | O |
| GLN498 | ARG345 | 6.49 | 0.02 | 0 | 1 |  | HB2 | HH12 |
| ASN448 | LYS341 | 6.50 | -0.08 | 0 | 1 |  | H | HZ2 |
| THR500 | GLY340 | 6.51 | -0.01 | 0 | 0 |  | HG1 | O |
| VAL445 | ASN49 | 6.51 | -0.02 | 0 | 0 |  | HG13 | HD22 |
| LEU455 | LEU29 | 6.52 | -0.07 | 0 | 0 |  | HD13 | O |
| GLY447 | LEU45 | 6.52 | -0.06 | 0 | 0 |  | N | HD13 |
| GLN506 | GLU313 | 6.54 | -0.18 | 0 | -1 |  | HE22 | HB2 |
| PHE490 | GLU35 | 6.55 | 0.12 | 0 | -1 |  | O | OE2 |
| CYS488 | ARG31 | 6.58 | -0.22 | 0 | 1 |  | C | HD2 |
| GLN498 | ASP343 | 6.60 | -0.07 | 0 | -1 |  | HB1 | HB1 |
| GLY485 | ASP75 | 6.60 | -0.17 | 0 | -1 |  | HA1 | OD1 |
| ASN501 | GLN38 | 6.62 | -0.11 | 0 | 0 |  | HD22 | HA |
| ARG457 | ARG31 | 6.63 | 0.52 | 1 | 1 |  | H | HH22 |
| PHE486 | SER84 | 6.63 | -0.07 | 0 | 0 |  | HZ | H |
| ASN448 | GLN38 | 6.64 | -0.09 | 0 | 0 |  | O | HE22 |
| GLY446 | LYS341 | 6.66 | -0.02 | 0 | 1 |  | O | HZ2 |
| LYS417 | PHE28 | 6.66 | -0.22 | 1 | 0 |  | HZ1 | N |
| ARG403 | ASN33 | 6.66 | 0.12 | 1 | 0 |  | HH12 | HB2 |
| PRO499 | LYS317 | 6.73 | -0.52 | 0 | 1 |  | O | HZ2 |
| ALA475 | SER20 | 6.73 | -0.28 | 0 | 1 |  | O | H2 |
| LEU452 | GLN38 | 6.74 | -0.01 | 0 | 0 |  | HA | HE22 |
| LYS444 | PHE41 | 6.76 | -0.03 | 1 | 0 |  | O | HE1 |
| PHE456 | PHE32 | 6.77 | -0.07 | 0 | 0 |  | HE1 | H |
| GLY416 | TYR34 | 6.77 | -0.02 | 0 | 0 |  | HA2 | HH |
| TYR505 | PHE344 | 6.79 | -0.09 | 0 | 0 |  | HD1 | HE2 |
| PHE490 | ARG79 | 6.81 | -0.17 | 0 | 1 |  | H | HH22 |
| SER494 | LYS341 | 6.82 | 0.00 | 0 | 1 |  | O | HZ3 |
| TYR453 | ASN33 | 6.83 | -0.29 | 0 | 0 |  | OH | C |
| ASN501 | PHE344 | 6.85 | -0.22 | 0 | 0 |  | HA | H |
| ASN487 | ALA80 | 6.85 | -0.09 | 0 | 0 |  | HD22 | HA |
| GLN506 | LYS317 | 6.86 | 0.14 | 0 | 1 |  | HE22 | HZ2 |
| LYS417 | LEU29 | 6.86 | 0.42 | 1 | 0 |  | HZ1 | C |
| LYS417 | ASN33 | 6.87 | 0.25 | 1 | 0 |  | HE1 | HB1 |
| GLN498 | GLU37 | 6.87 | 0.12 | 0 | -1 |  | OE1 | O |
| GLN474 | ARG31 | 6.88 | 0.12 | 0 | 1 |  | C | HH12 |
| ASN487 | ALA25 | 6.88 | 0.00 | 0 | 0 |  | HD22 | HA |
| PHE486 | GLU24 | 6.88 | -0.11 | 0 | -1 |  | HZ | HB2 |
| GLN498 | LEU39 | 6.89 | -0.09 | 0 | 0 |  | HE22 | N |
| GLY496 | GLU42 | 6.89 | -0.02 | 0 | -1 |  | HA2 | OE2 |
| ALA475 | ASN23 | 6.95 | -0.28 | 0 | 0 |  | HB3 | O |
| GLN506 | GLY314 | 6.96 | -0.01 | 0 | 0 |  | HE21 | HA2 |
| ALA475 | ALA25 | 6.96 | -0.01 | 0 | 0 |  | HB3 | N |
| ASN439 | LYS317 | 6.96 | 0.05 | 0 | 1 |  | HD21 | HZ2 |
| GLY496 | TYR34 | 6.96 | -0.04 | 0 | 0 |  | H | O |
| TYR453 | GLU37 | 6.97 | 0.01 | 0 | -1 |  | HH | HB2 |
| VAL445 | ARG345 | 6.99 | 0.07 | 0 | 1 |  | HG13 | HH12 |
| GLY476 | ARG31 | 7.00 | -0.23 | 0 | 1 |  | N | HH12 |
| ALA475 | THR21 | 7.05 | -0.01 | 0 | 0 |  | O | HA |
| GLN474 | GLU24 | 7.05 | -0.12 | 0 | -1 |  | O | HG2 |
| GLY502 | GLU313 | 7.06 | -0.06 | 0 | -1 |  | HA1 | H |
| ASN501 | GLU37 | 7.07 | -0.23 | 0 | -1 |  | HD22 | O |
| LYS417 | ASN23 | 7.11 | -0.34 | 1 | 0 |  | HZ1 | O |
| SER477 | GLU24 | 7.11 | -0.15 | 0 | -1 |  | H | HG1 |
| ASN501 | THR312 | 7.11 | -0.06 | 0 | 0 |  | O | HB |
| THR500 | GLU313 | 7.12 | -0.15 | 0 | -1 |  | HA | O |
| TYR449 | LEU45 | 7.12 | -0.04 | 0 | 0 |  | OH | HD11 |
| GLU484 | ARG31 | 7.13 | -1.02 | -1 | 1 |  | OE1 | HD1 |
| CYS488 | PHE28 | 7.14 | -0.07 | 0 | 0 |  | H | HE1 |
| GLY485 | PHE28 | 7.14 | -0.02 | 0 | 0 |  | O | HE1 |
| THR500 | THR312 | 7.16 | -0.08 | 0 | 0 |  | O | HB |
| PRO499 | ASP343 | 7.16 | -0.49 | 0 | -1 |  | C | OD2 |
| TYR421 | ARG31 | 7.19 | -0.21 | 0 | 1 |  | HE2 | HH22 |
| ASN487 | ARG76 | 7.19 | -0.09 | 0 | 1 |  | H | HH22 |
| GLY496 | GLU37 | 7.19 | -0.06 | 0 | -1 |  | H | HG1 |
| LEU455 | LYS26 | 7.19 | -0.12 | 0 | 1 |  | HD13 | O |
| GLY502 | PHE41 | 7.20 | -0.06 | 0 | 0 |  | H | HE2 |
| GLY485 | ARG76 | 7.20 | -0.10 | 0 | 1 |  | O | HH22 |
| PRO499 | GLY314 | 7.20 | -0.02 | 0 | 0 |  | O | HA1 |
| PHE456 | LEU29 | 7.22 | -0.06 | 0 | 0 |  | HE1 | N |
| TYR489 | ALA80 | 7.23 | -0.04 | 0 | 0 |  | HH | HA |
| GLN498 | GLY340 | 7.23 | -0.06 | 0 | 0 |  | OE1 | O |
| VAL503 | GLY314 | 7.24 | -0.01 | 0 | 0 |  | HG21 | H |
| LYS444 | LEU45 | 7.24 | -0.01 | 1 | 0 |  | O | HD13 |
| TYR489 | PHE32 | 7.27 | -0.10 | 0 | 0 |  | HE1 | N |
| CYS488 | ARG76 | 7.28 | -0.10 | 0 | 1 |  | O | HH22 |
| GLN493 | ALA36 | 7.29 | -0.15 | 0 | 0 |  | HG1 | H |
| ASN487 | THR21 | 7.29 | -0.05 | 0 | 0 |  | HD22 | HG22 |
| PHE456 | LYS26 | 7.30 | 0.00 | 0 | 1 |  | HE1 | O |
| GLN506 | ASN318 | 7.30 | 0.01 | 0 | 0 |  | HE21 | HD22 |
| ASN487 | SER84 | 7.32 | -0.02 | 0 | 0 |  | HD22 | H |
| TYR489 | PHE72 | 7.33 | -0.03 | 0 | 0 |  | HE1 | HE1 |
| TYR495 | GLU35 | 7.34 | 0.14 | 0 | -1 |  | HA | HA |
| GLY502 | ASN318 | 7.34 | -0.01 | 0 | 0 |  | HA1 | HD22 |
| TYR453 | ALA36 | 7.34 | -0.06 | 0 | 0 |  | HH | H |
| TYR489 | ASP75 | 7.35 | 0.01 | 0 | -1 |  | HE1 | OD2 |
| VAL503 | PHE344 | 7.36 | -0.03 | 0 | 0 |  | H | HE2 |
| LYS458 | THR27 | 7.36 | -0.04 | 1 | 0 |  | HA | HG22 |
| TYR473 | PHE83 | 7.37 | -0.02 | 0 | 0 |  | HE2 | HZ |
| ASN501 | GLU42 | 7.37 | -0.08 | 0 | -1 |  | HD22 | H |
| TYR489 | ALA25 | 7.37 | -0.03 | 0 | 0 |  | HH | HA |
| PHE486 | CYS77 | 7.38 | -0.07 | 0 | 0 |  | HE1 | O |
| PHE490 | ARG76 | 7.39 | -0.25 | 0 | 1 |  | H | HH22 |
| ASP405 | ALA374 | 7.41 | 0.08 | -1 | 0 |  | OD2 | O |
| TYR473 | SER20 | 7.41 | 0.05 | 0 | 1 |  | HH | H2 |
| GLY496 | LEU39 | 7.43 | -0.02 | 0 | 0 |  | HA2 | N |
| ALA475 | ARG79 | 7.44 | 0.06 | 0 | 1 |  | HB2 | HH21 |
| TYR505 | PHE41 | 7.44 | -0.13 | 0 | 0 |  | HE2 | HB1 |
| THR500 | PHE315 | 7.45 | -0.05 | 0 | 0 |  | O | H |
| TYR449 | GLU35 | 7.45 | -0.08 | 0 | -1 |  | HE1 | HA |
| GLY416 | ASP30 | 7.51 | -0.26 | 0 | -1 |  | HA1 | OD2 |
| PRO499 | LYS341 | 7.53 | -0.08 | 0 | 1 |  | N | HZ2 |
| ARG403 | PRO377 | 7.53 | -0.03 | 1 | 0 |  | HH12 | HA |
| LEU455 | ALA36 | 7.54 | -0.03 | 0 | 0 |  | HD21 | H |
| GLN493 | PHE32 | 7.54 | -0.09 | 0 | 0 |  | HG1 | N |
| ASN487 | THR27 | 7.54 | -0.06 | 0 | 0 |  | OD1 | OG1 |
| CYS488 | PHE83 | 7.55 | -0.03 | 0 | 0 |  | N | HZ |
| LYS444 | LYS341 | 7.55 | 0.68 | 1 | 1 |  | O | HZ2 |
| GLN506 | THR312 | 7.57 | 0.00 | 0 | 0 |  | HE21 | HB |
| ASP405 | LYS341 | 7.57 | -0.87 | -1 | 1 |  | OD2 | HA |
| SER443 | LYS341 | 7.57 | 0.12 | 0 | 1 |  | HB1 | HZ2 |
| THR500 | ASN49 | 7.58 | -0.03 | 0 | 0 |  | HG21 | HD22 |
| TYR505 | ALA36 | 7.59 | 0.20 | 0 | 0 |  | HH | HB2 |
| GLN493 | ASN33 | 7.60 | -0.07 | 0 | 0 |  | HG1 | C |
| TYR473 | PHE28 | 7.60 | -0.06 | 0 | 0 |  | HE2 | N |
| GLN506 | ASP343 | 7.61 | -0.14 | 0 | -1 |  | HE21 | HA |
| THR500 | TRP48 | 7.61 | -0.04 | 0 | 0 |  | HG21 | HE1 |
| GLY446 | LYS68 | 7.62 | -0.18 | 0 | 1 |  | O | HZ3 |
| GLY485 | ARG31 | 7.62 | -0.10 | 0 | 1 |  | O | HD1 |
| TYR453 | LYS341 | 7.62 | -0.05 | 0 | 1 |  | HE1 | HZ3 |
| GLY496 | LEU45 | 7.63 | -0.02 | 0 | 0 |  | O | HD11 |
| PHE486 | ARG31 | 7.63 | 0.01 | 0 | 1 |  | HE1 | HH11 |
| PRO507 | LYS341 | 7.63 | -0.05 | 0 | 1 |  | HD2 | HD2 |
| VAL503 | ASP343 | 7.64 | -0.03 | 0 | -1 |  | H | HA |
| GLY504 | GLU375 | 7.65 | -0.02 | 0 | -1 |  | HA2 | HG2 |
| ASP405 | ARG381 | 7.66 | -1.14 | -1 | 1 |  | OD2 | HH21 |
| TYR489 | LEU29 | 7.68 | -0.07 | 0 | 0 |  | OH | N |
| PRO499 | ARG345 | 7.70 | 0.21 | 0 | 1 |  | HD1 | HH12 |
| ARG403 | GLN376 | 7.70 | -0.27 | 1 | 0 |  | HH22 | O |
| GLY476 | ASP82 | 7.72 | -0.20 | 0 | -1 |  | HA2 | HB2 |
| GLU484 | ASP75 | 7.72 | 1.30 | -1 | -1 |  | HB2 | OD2 |
| ASP420 | ASP30 | 7.72 | 1.21 | -1 | -1 |  | OD2 | OD2 |
| GLY504 | PHE344 | 7.74 | -0.02 | 0 | 0 |  | H | HE2 |
| TYR505 | GLU35 | 7.75 | 0.02 | 0 | -1 |  | HE2 | N |
| GLY504 | TYR34 | 7.77 | -0.04 | 0 | 0 |  | O | HE1 |
| GLN493 | ARG79 | 7.78 | 0.05 | 0 | 1 |  | HE22 | HH22 |
| GLY446 | GLN38 | 7.78 | -0.01 | 0 | 0 |  | O | O |
| ASN448 | PHE41 | 7.79 | -0.02 | 0 | 0 |  | H | HE1 |
| ASN487 | ASP75 | 7.81 | -0.05 | 0 | -1 |  | H | OD1 |
| ASN439 | ASN318 | 7.81 | 0.00 | 0 | 0 |  | HD22 | HD22 |
| ASP420 | THR27 | 7.82 | -0.03 | -1 | 0 |  | OD2 | HG23 |
| TYR495 | GLU37 | 7.82 | -0.07 | 0 | -1 |  | HA | HG1 |
| PHE486 | GLN101 | 7.82 | 0.00 | 0 | 0 |  | HE1 | HE21 |
| LEU455 | GLN38 | 7.83 | -0.03 | 0 | 0 |  | HD22 | HG2 |
| GLN498 | ASN318 | 7.83 | -0.06 | 0 | 0 |  | HB2 | HD22 |
| GLY502 | GLU37 | 7.84 | 0.03 | 0 | -1 |  | H | OE2 |
| SER443 | PHE41 | 7.85 | 0.00 | 0 | 0 |  | HB1 | HE1 |
| VAL445 | LYS61 | 7.88 | -0.02 | 0 | 1 |  | HG12 | HZ1 |
| VAL445 | ASN318 | 7.89 | -0.04 | 0 | 0 |  | HG23 | OD1 |
| TYR421 | ASN23 | 7.89 | -0.01 | 0 | 0 |  | OH | HB2 |
| GLY496 | GLU35 | 7.89 | -0.01 | 0 | -1 |  | HA2 | HA |
| ARG408 | TYR34 | 7.90 | 0.00 | 1 | 0 |  | HH11 | HH |
| ASN501 | GLU313 | 7.92 | 0.03 | 0 | -1 |  | O | HB2 |
| GLY502 | LEU339 | 7.93 | 0.00 | 0 | 0 |  | H | HB1 |
| TYR449 | TYR34 | 7.94 | -0.03 | 0 | 0 |  | HE1 | O |
| GLY502 | PHE315 | 7.94 | 0.00 | 0 | 0 |  | HA1 | H |
| GLY476 | THR21 | 7.96 | -0.01 | 0 | 0 |  | HA1 | HA |
| TYR505 | SER40 | 7.96 | -0.02 | 0 | 0 |  | HH | OG |
| TYR421 | TYR34 | 7.97 | -0.05 | 0 | 0 |  | HD2 | HE2 |
| SER477 | PHE83 | 7.98 | -0.03 | 0 | 0 |  | H | HE1 |
| TYR453 | ARG76 | 7.98 | -0.05 | 0 | 1 |  | HH | HH11 |

**Supplementary** **table 23** Interactions between SARS-CoV-2 RBD e *Numida meleagris* ACE2.

| **Interaction** | **SARS-CoV-2  RBD** | ***Numida meleagris*  ACE2** |
| --- | --- | --- |
|  | Residue | Residue |
| Hydrophobic | PHE456 | ILE28 |
|  | TYR473 | ILE28 |
|  | ALA475 | ILE28 |
|  | TYR489 | ILE28 |
|  | PHE486 | PHE29 |
|  | TYR489 | PHE29 |
|  | PHE456 | ALA31 |
|  | TYR453 | VAL35 |
|  | LEU455 | VAL35 |
|  | PHE486 | PHE84 |
| Hydrogen bonds | GLY502 | LYS355 |
|  | GLY496 | LYS355 |
|  | THR500 | ASP357 |
|  | GLU484 | ARG36 |
|  | ASN501 | TYR42 |
|  | GLN498 | LYS355 |
|  | TYR505 | ARG395 |
|  | THR500 | TYR42 |
|  | ASN501 | TYR42 |
|  | TYR505 | GLU38 |
| Ionic | GLU484 | ARG36 |
| Aromatic-Aromatic | TYR489 | PHE29 |
|  | PHE486 | PHE84 |
| Cation-Pi | PHE486 | ARG83 |

**Supplementary** **table 24**. Quantum biochemistry between SARS-CoV-2 RBD e *Numida meleagris* ACE2.

| **Amino acid residues** | | **Distance** | **Interaction energy** | **Residue charge** | |  | **Residue atom** | |
| --- | --- | --- | --- | --- | --- | --- | --- | --- |
| SARS-CoV-2  RBD | *Numida meleagris* ACE2 | (Å) | (kcal mol-1) | SARS-CoV-2  RBD | *Numida meleagris* ACE2 |  | SARS-CoV-2  RBD | *Numida meleagris* ACE2 |
| TYR505 | GLU38 | 1.74 | -8.43 | 0 | -1 |  | HH | OE1 |
| GLU484 | ARG36 | 1.77 | -11.81 | -1 | 1 |  | OE1 | HH12 |
| GLY496 | LYS355 | 1.85 | -5.15 | 0 | 1 |  | O | HZ2 |
| THR500 | ASN332 | 1.87 | -0.76 | 0 | 0 |  | HG22 | HD22 |
| THR500 | TYR42 | 1.89 | -0.45 | 0 | 0 |  | HG1 | OH |
| GLN493 | VAL35 | 2.02 | -1.38 | 0 | 0 |  | HG1 | HG11 |
| PHE486 | PHE84 | 2.07 | -2.65 | 0 | 0 |  | HE1 | HE2 |
| PHE456 | GLU32 | 2.18 | -2.91 | 0 | -1 |  | HZ | HB1 |
| TYR505 | ASN356 | 2.20 | -1.99 | 0 | 0 |  | HD1 | HD21 |
| TYR473 | ILE28 | 2.20 | -2.44 | 0 | 0 |  | HE2 | HD2 |
| TYR505 | ARG395 | 2.22 | 2.20 | 0 | 1 |  | HH | HH22 |
| GLY502 | ASN356 | 2.27 | -3.66 | 0 | 0 |  | HA2 | HB1 |
| GLY502 | LYS355 | 2.30 | -3.32 | 0 | 1 |  | H | O |
| TYR489 | ILE28 | 2.32 | -1.29 | 0 | 0 |  | HE2 | HG23 |
| ALA475 | ILE28 | 2.33 | -2.32 | 0 | 0 |  | HB1 | HG22 |
| ASN501 | LYS355 | 2.35 | -9.91 | 0 | 1 |  | HD22 | HD1 |
| LEU455 | GLU32 | 2.37 | -1.88 | 0 | -1 |  | HD21 | HA |
| GLN498 | LYS355 | 2.38 | -2.75 | 0 | 1 |  | OE1 | HZ1 |
| TYR453 | VAL35 | 2.46 | -1.47 | 0 | 0 |  | HH | HG22 |
| TYR505 | LYS355 | 2.50 | -7.54 | 0 | 1 |  | HD2 | HG1 |
| LEU455 | VAL35 | 2.51 | -2.48 | 0 | 0 |  | HD22 | HG11 |
| TYR489 | ARG36 | 2.52 | 1.16 | 0 | 1 |  | HB2 | HH11 |
| THR500 | ASP357 | 2.53 | -2.18 | 0 | -1 |  | O | HB1 |
| PHE456 | ILE28 | 2.54 | -2.70 | 0 | 0 |  | HE1 | HG21 |
| TYR489 | PHE29 | 2.54 | -1.92 | 0 | 0 |  | OH | HD1 |
| PHE486 | ARG83 | 2.59 | -6.03 | 0 | 1 |  | HZ | HB1 |
| GLN498 | TYR42 | 2.60 | -2.45 | 0 | 0 |  | HG1 | HE2 |
| PHE486 | ASN80 | 2.67 | -2.57 | 0 | 0 |  | HD1 | HB2 |
| ASN501 | TYR42 | 2.71 | -2.26 | 0 | 0 |  | HD21 | OH |
| THR500 | ARG359 | 2.76 | -0.83 | 0 | 1 |  | HB | HH11 |
| LEU455 | ALA31 | 2.76 | -1.32 | 0 | 0 |  | HD21 | O |
| ASN501 | ASP357 | 2.80 | -2.45 | 0 | -1 |  | HA | HB1 |
| PHE490 | ARG36 | 2.89 | 0.40 | 0 | 1 |  | H | HH12 |
| TYR489 | GLU32 | 2.93 | -3.22 | 0 | -1 |  | CD2 | HG2 |
| ASN487 | PHE84 | 2.93 | -1.00 | 0 | 0 |  | OD1 | HZ |
| ALA475 | GLU25 | 3.03 | -1.88 | 0 | -1 |  | O | HG1 |
| GLN498 | LEU46 | 3.03 | -0.86 | 0 | 0 |  | HG1 | HD13 |
| TYR449 | ASP39 | 3.17 | -0.76 | 0 | -1 |  | HE1 | OD1 |
| GLY496 | ASP39 | 3.25 | -0.38 | 0 | -1 |  | HA2 | OD1 |
| PHE456 | ALA31 | 3.28 | -1.01 | 0 | 0 |  | HZ | HB1 |
| THR500 | LEU46 | 3.37 | -0.43 | 0 | 0 |  | HG1 | HD21 |
| GLY476 | GLU25 | 3.50 | -1.82 | 0 | -1 |  | HA1 | HG1 |
| GLY502 | ASP357 | 3.55 | -1.25 | 0 | -1 |  | H | N |
| GLY502 | THR326 | 3.61 | -0.44 | 0 | 0 |  | HA2 | HG22 |
| PHE486 | PHE29 | 3.63 | -1.09 | 0 | 0 |  | HD1 | HE1 |
| LYS417 | ALA31 | 3.63 | -0.33 | 1 | 0 |  | HE2 | HB2 |
| ASN487 | GLU25 | 3.65 | -1.23 | 0 | -1 |  | HD22 | HB2 |
| TYR489 | PHE84 | 3.69 | -0.02 | 0 | 0 |  | HH | HZ |
| THR500 | GLY328 | 3.78 | -0.28 | 0 | 0 |  | HA | HA1 |
| GLN493 | ARG36 | 3.86 | -1.40 | 0 | 1 |  | HG2 | HG1 |
| TYR449 | LYS355 | 3.87 | -0.25 | 0 | 1 |  | HE1 | HZ3 |
| ASN501 | ASN356 | 3.88 | -2.55 | 0 | 0 |  | HA | C |
| GLN498 | ASP39 | 3.91 | -1.33 | 0 | -1 |  | HE22 | OD1 |
| TYR505 | SER388 | 4.09 | -0.11 | 0 | 0 |  | HE1 | O |
| PHE497 | LYS355 | 4.12 | -2.41 | 0 | 1 |  | N | HZ2 |
| TYR449 | GLU43 | 4.14 | -1.45 | 0 | -1 |  | HH | HG2 |
| GLY446 | LEU46 | 4.15 | -0.32 | 0 | 0 |  | HA1 | HD12 |
| GLN493 | GLU32 | 4.15 | -0.87 | 0 | -1 |  | OE1 | HG1 |
| ASN487 | PHE29 | 4.21 | -0.85 | 0 | 0 |  | H | HD1 |
| VAL503 | ASN356 | 4.22 | -1.14 | 0 | 0 |  | N | HB1 |
| GLY504 | ASN356 | 4.24 | -1.24 | 0 | 0 |  | H | HB1 |
| GLY502 | GLY328 | 4.33 | -0.10 | 0 | 0 |  | HA1 | HA2 |
| THR500 | ASN356 | 4.47 | -0.64 | 0 | 0 |  | O | O |
| GLY502 | TYR358 | 4.49 | -0.32 | 0 | 0 |  | HA2 | HE2 |
| SER494 | VAL35 | 4.54 | -0.19 | 0 | 0 |  | O | HG13 |
| PHE486 | ALA81 | 4.55 | -0.21 | 0 | 0 |  | HE1 | HA |
| VAL503 | THR326 | 4.62 | -0.31 | 0 | 0 |  | H | HG22 |
| THR500 | TYR358 | 4.64 | -0.20 | 0 | 0 |  | O | H |
| ASN487 | ILE28 | 4.72 | -0.59 | 0 | 0 |  | HA | HG23 |
| GLN498 | GLU43 | 4.73 | -0.53 | 0 | -1 |  | HE22 | HG2 |
| TYR489 | GLU25 | 4.76 | -0.73 | 0 | -1 |  | HH | O |
| ASN501 | GLY328 | 4.76 | -0.07 | 0 | 0 |  | O | HA2 |
| PHE456 | GLN27 | 4.78 | -0.27 | 0 | 0 |  | HE1 | O |
| ASN501 | GLY354 | 4.81 | -0.28 | 0 | 0 |  | HA | O |
| PHE456 | PHE29 | 4.85 | -0.10 | 0 | 0 |  | HZ | HA |
| GLN506 | GLU327 | 4.91 | -0.20 | 0 | -1 |  | HE22 | HB1 |
| PHE456 | PHE33 | 4.92 | -0.17 | 0 | 0 |  | HZ | H |
| GLY446 | GLU43 | 4.92 | 0.24 | 0 | -1 |  | O | HG2 |
| LYS417 | ILE28 | 4.94 | -0.33 | 1 | 0 |  | HZ1 | HA |
| GLU484 | GLU32 | 4.98 | 1.56 | -1 | -1 |  | OE1 | OE2 |
| ASP405 | ASN356 | 5.03 | -0.34 | -1 | 0 |  | OD2 | HD22 |
| ARG403 | ASN356 | 5.08 | -0.02 | 1 | 0 |  | HH21 | HD22 |
| THR500 | THR331 | 5.10 | -0.54 | 0 | 0 |  | HG22 | OG1 |
| GLN506 | GLY328 | 5.13 | -0.04 | 0 | 0 |  | HE21 | HA2 |
| SER494 | ASP39 | 5.16 | -0.23 | 0 | -1 |  | HB2 | OD2 |
| PRO499 | TYR42 | 5.19 | -0.54 | 0 | 0 |  | HD1 | OH |
| PHE456 | ARG36 | 5.20 | -0.21 | 0 | 1 |  | HE2 | HH11 |
| GLY447 | LYS355 | 5.24 | 0.04 | 0 | 1 |  | HA2 | HZ2 |
| TYR505 | VAL389 | 5.26 | -0.12 | 0 | 0 |  | HE1 | HA |
| GLY446 | TYR42 | 5.28 | -0.21 | 0 | 0 |  | H | HE2 |
| THR500 | MET353 | 5.30 | -0.35 | 0 | 0 |  | HG1 | SD |
| PHE490 | GLU32 | 5.30 | 0.03 | 0 | -1 |  | H | OE2 |
| ALA475 | GLN24 | 5.36 | -0.34 | 0 | 0 |  | HB1 | O |
| ASP405 | VAL389 | 5.37 | -0.70 | -1 | 0 |  | OD1 | HG12 |
| CYS488 | ARG36 | 5.38 | 0.20 | 0 | 1 |  | O | HH12 |
| ARG403 | VAL389 | 5.39 | -0.17 | 1 | 0 |  | HH22 | HA |
| PRO499 | GLY328 | 5.39 | -0.16 | 0 | 0 |  | O | HA1 |
| TYR489 | ASN80 | 5.40 | -0.12 | 0 | 0 |  | HE1 | HD21 |
| TYR495 | LYS355 | 5.41 | 1.29 | 0 | 1 |  | C | HZ2 |
| ALA475 | PHE29 | 5.42 | -0.15 | 0 | 0 |  | HB1 | H |
| PRO499 | ASN332 | 5.42 | -0.09 | 0 | 0 |  | O | HD22 |
| VAL445 | LEU46 | 5.42 | -0.13 | 0 | 0 |  | HG11 | HD22 |
| THR500 | LYS355 | 5.42 | -0.54 | 0 | 1 |  | O | O |
| VAL503 | GLU327 | 5.42 | -0.48 | 0 | -1 |  | HG23 | HB1 |
| GLY476 | ILE28 | 5.42 | -0.14 | 0 | 0 |  | N | HD1 |
| LEU455 | ASN34 | 5.43 | -0.11 | 0 | 0 |  | HD21 | HB1 |
| LEU455 | PHE33 | 5.43 | -0.19 | 0 | 0 |  | HD21 | N |
| LEU455 | ARG36 | 5.44 | -0.19 | 0 | 1 |  | HD22 | HG2 |
| TYR505 | PHE392 | 5.45 | -0.08 | 0 | 0 |  | HH | HD1 |
| GLY502 | GLY354 | 5.46 | -0.04 | 0 | 0 |  | H | O |
| ARG403 | GLU38 | 5.47 | -1.90 | 1 | -1 |  | HH12 | OE1 |
| ARG403 | SER388 | 5.50 | -0.42 | 1 | 0 |  | HH22 | O |
| LYS417 | VAL35 | 5.54 | -0.05 | 1 | 0 |  | HE2 | HG21 |
| ARG403 | LYS355 | 5.55 | 1.04 | 1 | 1 |  | HD1 | HG1 |
| ARG403 | VAL35 | 5.57 | -0.21 | 1 | 0 |  | HH11 | HG22 |
| GLN506 | THR326 | 5.59 | -0.05 | 0 | 0 |  | HE22 | HB |
| PRO491 | GLU32 | 5.60 | 0.03 | 0 | -1 |  | HA | HG1 |
| GLN474 | ILE28 | 5.61 | 0.02 | 0 | 0 |  | O | HD2 |
| LEU455 | ILE28 | 5.61 | -0.40 | 0 | 0 |  | HD21 | O |
| GLN498 | ASP357 | 5.62 | -0.06 | 0 | -1 |  | HB1 | HB1 |
| ASN501 | LEU46 | 5.64 | -0.09 | 0 | 0 |  | HD22 | HD13 |
| GLY502 | TYR42 | 5.64 | -0.12 | 0 | 0 |  | H | HE1 |
| ASN501 | ARG359 | 5.65 | -0.14 | 0 | 1 |  | HD21 | HH11 |
| GLN506 | LYS355 | 5.65 | -0.43 | 0 | 1 |  | HG2 | O |
| GLN506 | ASN356 | 5.65 | -0.27 | 0 | 0 |  | HG2 | HB1 |
| ASN487 | ASN80 | 5.66 | -0.22 | 0 | 0 |  | H | HB2 |
| PHE486 | GLU25 | 5.67 | -0.58 | 0 | -1 |  | HZ | HB2 |
| PRO499 | LEU46 | 5.67 | -0.06 | 0 | 0 |  | HD1 | HD21 |
| TYR449 | TYR42 | 5.67 | -0.16 | 0 | 0 |  | OH | HD2 |
| TYR505 | GLY354 | 5.68 | -0.29 | 0 | 0 |  | HH | HA1 |
| SER477 | GLU25 | 5.69 | -0.83 | 0 | -1 |  | HB2 | OE2 |
| ALA475 | PHE84 | 5.71 | -0.09 | 0 | 0 |  | HB3 | HZ |
| TYR495 | ASP39 | 5.72 | -0.25 | 0 | -1 |  | C | OD1 |
| TYR495 | VAL35 | 5.74 | -0.19 | 0 | 0 |  | HA | HG13 |
| PHE456 | LEU30 | 5.75 | -0.12 | 0 | 0 |  | HZ | N |
| ARG403 | ARG395 | 5.76 | 1.45 | 1 | 1 |  | HH12 | HH22 |
| TYR505 | ASN34 | 5.76 | -0.16 | 0 | 0 |  | HH | O |
| ASN501 | MET353 | 5.78 | -0.15 | 0 | 0 |  | HD21 | HB1 |
| VAL503 | LYS355 | 5.80 | 0.08 | 0 | 1 |  | N | O |
| VAL503 | GLY328 | 5.80 | -0.05 | 0 | 0 |  | HG23 | H |
| GLY446 | LYS355 | 5.85 | -0.10 | 0 | 1 |  | O | HZ1 |
| GLN493 | ALA31 | 5.85 | -0.07 | 0 | 0 |  | HG1 | O |
| TYR505 | VAL35 | 5.85 | -0.17 | 0 | 0 |  | HE2 | HA |
| PHE456 | VAL35 | 5.88 | -0.32 | 0 | 0 |  | HE2 | HB |
| GLN498 | ARG359 | 5.90 | -0.06 | 0 | 1 |  | HB2 | HH12 |
| GLY504 | LYS355 | 5.91 | -0.29 | 0 | 1 |  | H | O |
| TYR505 | ASP357 | 5.93 | -0.21 | 0 | -1 |  | HB1 | H |
| GLY502 | GLU327 | 5.98 | -0.06 | 0 | -1 |  | HA1 | H |
| TYR473 | GLU32 | 6.00 | 0.04 | 0 | -1 |  | HD2 | HG2 |
| GLY485 | ARG36 | 6.00 | -0.39 | 0 | 1 |  | H | HH12 |
| ARG408 | VAL389 | 6.04 | -0.07 | 1 | 0 |  | HH12 | HG12 |
| GLY485 | ASN80 | 6.08 | -0.18 | 0 | 0 |  | O | HD22 |
| ILE472 | ARG36 | 6.09 | 0.14 | 0 | 1 |  | HD3 | HH12 |
| VAL445 | ASN50 | 6.09 | -0.06 | 0 | 0 |  | HG11 | HD22 |
| GLY447 | LEU46 | 6.10 | -0.06 | 0 | 0 |  | N | HD13 |
| LYS417 | GLN27 | 6.10 | 0.04 | 1 | 0 |  | HZ1 | O |
| ASN487 | ARG83 | 6.12 | -0.56 | 0 | 1 |  | HD22 | HB1 |
| LYS417 | GLU32 | 6.12 | -1.09 | 1 | -1 |  | HZ1 | N |
| PHE486 | SER82 | 6.14 | -0.18 | 0 | 0 |  | HE1 | H |
| GLY447 | ASP39 | 6.15 | -0.02 | 0 | -1 |  | HA2 | OD1 |
| PHE486 | ARG79 | 6.15 | -0.07 | 0 | 1 |  | HE1 | O |
| TYR449 | LEU46 | 6.16 | -0.04 | 0 | 0 |  | HH | HD13 |
| TYR473 | GLU25 | 6.17 | -0.03 | 0 | -1 |  | HE2 | HA |
| PHE497 | ASP39 | 6.17 | 0.00 | 0 | -1 |  | N | OD1 |
| GLY496 | TYR42 | 6.18 | -0.13 | 0 | 0 |  | O | HD2 |
| GLY447 | TYR42 | 6.18 | -0.12 | 0 | 0 |  | HA1 | HE2 |
| GLY485 | PHE29 | 6.19 | -0.03 | 0 | 0 |  | O | HE1 |
| ASN501 | TYR358 | 6.21 | -0.29 | 0 | 0 |  | HA | H |
| PRO499 | ARG359 | 6.23 | 0.39 | 0 | 1 |  | HD1 | HH12 |
| CYS488 | GLU32 | 6.23 | -0.17 | 0 | -1 |  | O | OE2 |
| GLY485 | GLU32 | 6.25 | 0.29 | 0 | -1 |  | O | OE2 |
| THR500 | GLY354 | 6.27 | -0.04 | 0 | 0 |  | O | O |
| VAL445 | TYR42 | 6.28 | -0.09 | 0 | 0 |  | HA | HE2 |
| THR500 | GLU327 | 6.29 | -0.05 | 0 | -1 |  | HA | O |
| ASN501 | ASN332 | 6.32 | -0.11 | 0 | 0 |  | N | HD22 |
| THR500 | PHE329 | 6.32 | -0.11 | 0 | 0 |  | O | N |
| SER494 | LYS355 | 6.32 | -0.18 | 0 | 1 |  | O | HZ3 |
| TYR453 | ALA31 | 6.34 | -0.05 | 0 | 0 |  | OH | O |
| PRO491 | ARG36 | 6.34 | -0.20 | 0 | 1 |  | HA | HH11 |
| ALA475 | GLU32 | 6.34 | -0.09 | 0 | -1 |  | HB2 | HG2 |
| ASN487 | GLU32 | 6.37 | -0.14 | 0 | -1 |  | HA | HG2 |
| VAL503 | TYR358 | 6.37 | -0.08 | 0 | 0 |  | H | HE2 |
| GLY476 | PHE84 | 6.38 | -0.04 | 0 | 0 |  | HA2 | HE1 |
| GLY504 | THR326 | 6.40 | -0.03 | 0 | 0 |  | H | HG22 |
| TYR489 | ALA26 | 6.40 | -0.22 | 0 | 0 |  | HH | HA |
| ASN501 | THR326 | 6.42 | -0.11 | 0 | 0 |  | C | HG22 |
| GLN506 | ASP357 | 6.43 | -0.15 | 0 | -1 |  | HE21 | HA |
| PHE486 | PRO85 | 6.45 | -0.09 | 0 | 0 |  | HZ | HD2 |
| TYR421 | ILE28 | 6.47 | -0.07 | 0 | 0 |  | HH | HG11 |
| PRO499 | ASP357 | 6.48 | -0.65 | 0 | -1 |  | C | HB1 |
| TYR505 | GLN390 | 6.48 | -0.04 | 0 | 0 |  | OH | O |
| LEU455 | LEU30 | 6.48 | -0.04 | 0 | 0 |  | HD21 | O |
| TYR473 | GLN24 | 6.52 | -0.05 | 0 | 0 |  | HE2 | O |
| TYR505 | PRO391 | 6.52 | -0.03 | 0 | 0 |  | HH | HA |
| PHE497 | TYR42 | 6.52 | -0.26 | 0 | 0 |  | C | HE2 |
| GLY485 | GLU76 | 6.55 | -0.07 | 0 | -1 |  | HA1 | OE1 |
| THR500 | THR326 | 6.55 | -0.06 | 0 | 0 |  | O | OG1 |
| ALA475 | ALA26 | 6.55 | -0.05 | 0 | 0 |  | HB1 | N |
| TYR505 | TYR358 | 6.57 | -0.12 | 0 | 0 |  | HE1 | HH |
| PHE456 | GLN24 | 6.58 | -0.04 | 0 | 0 |  | HE1 | O |
| CYS488 | PHE29 | 6.58 | -0.10 | 0 | 0 |  | H | HD1 |
| ASN448 | LYS355 | 6.60 | -0.18 | 0 | 1 |  | O | HZ2 |
| GLY502 | PHE329 | 6.61 | -0.03 | 0 | 0 |  | HA1 | H |
| ASP405 | SER388 | 6.62 | 0.26 | -1 | 0 |  | OD2 | O |
| ASN487 | ALA26 | 6.64 | 0.01 | 0 | 0 |  | OD1 | HA |
| SER494 | ARG36 | 6.64 | -0.06 | 0 | 1 |  | H | HG1 |
| GLY446 | ASP39 | 6.66 | 0.08 | 0 | -1 |  | O | OD1 |
| GLN493 | ASP39 | 6.67 | -0.32 | 0 | -1 |  | HG2 | HB1 |
| TYR489 | LEU30 | 6.67 | -0.14 | 0 | 0 |  | HH | N |
| PHE486 | ALA77 | 6.68 | -0.09 | 0 | 0 |  | HD1 | HA |
| GLN498 | GLU38 | 6.70 | 0.16 | 0 | -1 |  | OE1 | O |
| VAL445 | ARG359 | 6.70 | 0.05 | 0 | 1 |  | HG11 | HH12 |
| PHE456 | ASN34 | 6.70 | -0.08 | 0 | 0 |  | HZ | H |
| GLY504 | TYR358 | 6.71 | -0.04 | 0 | 0 |  | H | HE2 |
| GLY504 | SER388 | 6.73 | -0.03 | 0 | 0 |  | HA2 | HB1 |
| PHE486 | ALA26 | 6.73 | -0.10 | 0 | 0 |  | HE1 | HA |
| TYR453 | ASN34 | 6.74 | -0.04 | 0 | 0 |  | HH | C |
| PRO491 | VAL35 | 6.80 | -0.05 | 0 | 0 |  | O | HG11 |
| LEU455 | PHE29 | 6.81 | -0.10 | 0 | 0 |  | HD21 | O |
| ALA475 | ASP21 | 6.81 | -0.23 | 0 | 0 |  | O | O |
| LEU492 | VAL35 | 6.82 | -0.06 | 0 | 0 |  | C | HG11 |
| ARG457 | ILE28 | 6.83 | -0.10 | 1 | 0 |  | H | HG11 |
| PRO499 | THR331 | 6.84 | -0.11 | 0 | 0 |  | O | OG1 |
| TYR505 | TYR42 | 6.84 | -0.16 | 0 | 0 |  | HB2 | HE1 |
| ASN501 | ASP39 | 6.85 | -0.01 | 0 | -1 |  | HD22 | HA |
| LYS444 | TYR42 | 6.88 | -0.06 | 1 | 0 |  | O | HE2 |
| LEU492 | ARG36 | 6.89 | -0.17 | 0 | 1 |  | O | HH22 |
| TYR505 | ASP39 | 6.90 | -0.03 | 0 | -1 |  | HE2 | N |
| CYS488 | ILE28 | 6.91 | -0.12 | 0 | 0 |  | N | HG23 |
| TYR453 | ARG36 | 6.92 | -0.20 | 0 | 1 |  | HH | N |
| TYR473 | PHE29 | 6.92 | -0.08 | 0 | 0 |  | HE2 | H |
| ASN487 | VAL22 | 6.92 | -0.03 | 0 | 0 |  | HD22 | HA |
| PHE486 | VAL22 | 6.93 | -0.05 | 0 | 0 |  | HZ | HG13 |
| GLY504 | VAL389 | 6.94 | -0.02 | 0 | 0 |  | HA2 | HG12 |
| TYR449 | LYS69 | 6.96 | -0.07 | 0 | 1 |  | HH | HZ3 |
| GLY502 | MET353 | 6.98 | 0.00 | 0 | 0 |  | H | HB1 |
| GLY447 | GLU43 | 6.99 | 0.28 | 0 | -1 |  | HA2 | HG2 |
| GLY496 | VAL35 | 7.01 | -0.04 | 0 | 0 |  | H | HG13 |
| LYS417 | ASN34 | 7.02 | 0.23 | 1 | 0 |  | HE2 | HD21 |
| ASN439 | GLY328 | 7.03 | -0.02 | 0 | 0 |  | HD22 | HA1 |
| GLN493 | ASN34 | 7.03 | -0.09 | 0 | 0 |  | HG1 | C |
| LYS458 | ILE28 | 7.03 | -0.03 | 1 | 0 |  | HG1 | HD2 |
| TYR453 | GLU32 | 7.04 | 0.02 | 0 | -1 |  | OH | HA |
| ASN501 | GLU38 | 7.05 | -0.18 | 0 | -1 |  | HD22 | O |
| TYR489 | PHE33 | 7.06 | -0.15 | 0 | 0 |  | CE2 | H |
| GLY496 | GLU38 | 7.07 | -0.03 | 0 | -1 |  | O | HG1 |
| PHE486 | GLU76 | 7.09 | -0.11 | 0 | -1 |  | HD1 | O |
| GLY476 | ASP21 | 7.10 | -0.01 | 0 | 0 |  | HA1 | H2 |
| GLY502 | MET385 | 7.12 | -0.04 | 0 | 0 |  | HA2 | HG1 |
| TYR453 | GLU38 | 7.12 | -0.12 | 0 | -1 |  | HH | HB2 |
| GLN498 | ILE40 | 7.16 | -0.10 | 0 | 0 |  | HE22 | N |
| PRO499 | GLU327 | 7.18 | 0.01 | 0 | -1 |  | O | C |
| ASN501 | GLU327 | 7.22 | -0.10 | 0 | -1 |  | O | HB1 |
| LYS444 | LEU46 | 7.23 | -0.02 | 1 | 0 |  | O | HD13 |
| TYR489 | ALA31 | 7.24 | -0.18 | 0 | 0 |  | HE2 | H |
| VAL503 | ASP357 | 7.25 | 0.00 | 0 | -1 |  | H | HA |
| GLN474 | GLU25 | 7.26 | -0.17 | 0 | -1 |  | O | HG1 |
| THR500 | TRP49 | 7.26 | -0.05 | 0 | 0 |  | HG21 | HD1 |
| TYR489 | GLN24 | 7.27 | -0.02 | 0 | 0 |  | HE2 | O |
| TYR489 | GLN27 | 7.29 | -0.01 | 0 | 0 |  | HE2 | C |
| GLU406 | VAL35 | 7.30 | 0.00 | -1 | 0 |  | OE2 | HG22 |
| GLN493 | PHE33 | 7.31 | -0.10 | 0 | 0 |  | HG1 | N |
| LEU455 | GLN27 | 7.32 | -0.05 | 0 | 0 |  | HD11 | O |
| ASN439 | GLU327 | 7.32 | -0.18 | 0 | -1 |  | HD21 | HB1 |
| GLN498 | MET353 | 7.33 | -0.09 | 0 | 0 |  | HB1 | SD |
| ARG454 | VAL35 | 7.37 | 0.04 | 1 | 0 |  | C | HG11 |
| ALA475 | GLN27 | 7.38 | -0.06 | 0 | 0 |  | HB1 | C |
| ARG403 | ASN34 | 7.38 | -0.01 | 1 | 0 |  | HH12 | HB2 |
| GLN493 | ALA37 | 7.41 | -0.06 | 0 | 0 |  | HG1 | H |
| TYR449 | ILE40 | 7.41 | -0.08 | 0 | 0 |  | OH | HA |
| PHE456 | ALA26 | 7.43 | -0.03 | 0 | 0 |  | HE1 | O |
| PHE486 | GLU32 | 7.43 | -0.05 | 0 | -1 |  | HA | OE1 |
| ASN448 | ASP39 | 7.43 | 0.07 | 0 | -1 |  | O | OD1 |
| VAL445 | ASN332 | 7.45 | -0.05 | 0 | 0 |  | HG22 | OD1 |
| ASN439 | ASN332 | 7.45 | 0.00 | 0 | 0 |  | HD22 | HD22 |
| CYS488 | PHE84 | 7.46 | -0.03 | 0 | 0 |  | H | HZ |
| ASN487 | ARG36 | 7.48 | -0.01 | 0 | 1 |  | H | HH11 |
| ASP405 | LYS355 | 7.51 | -0.86 | -1 | 1 |  | OD2 | HA |
| GLY446 | ASN50 | 7.52 | -0.02 | 0 | 0 |  | HA1 | HD22 |
| GLN498 | GLY354 | 7.52 | -0.04 | 0 | 0 |  | OE1 | O |
| TYR421 | ALA31 | 7.52 | -0.02 | 0 | 0 |  | HE2 | HB2 |
| GLY476 | VAL22 | 7.52 | -0.01 | 0 | 0 |  | HA1 | HA |
| ALA475 | VAL22 | 7.54 | 0.01 | 0 | 0 |  | O | HA |
| TYR473 | ARG36 | 7.54 | -0.17 | 0 | 1 |  | H | HH12 |
| PHE456 | GLU25 | 7.55 | -0.07 | 0 | -1 |  | HE1 | HA |
| GLU484 | GLU76 | 7.56 | 1.27 | -1 | -1 |  | O | OE1 |
| GLN498 | ASN332 | 7.57 | -0.07 | 0 | 0 |  | HB2 | HD22 |
| PRO499 | LYS355 | 7.58 | -0.09 | 0 | 1 |  | N | HD2 |
| PRO491 | ILE28 | 7.59 | -0.01 | 0 | 0 |  | HB2 | HG21 |
| TYR489 | ALA77 | 7.60 | -0.02 | 0 | 0 |  | HE1 | HA |
| GLY446 | ARG359 | 7.61 | 0.06 | 0 | 1 |  | H | HH12 |
| GLY476 | GLN24 | 7.62 | -0.05 | 0 | 0 |  | HA1 | HB1 |
| GLN506 | PHE329 | 7.64 | -0.03 | 0 | 0 |  | HE22 | H |
| TYR453 | ASP39 | 7.65 | 0.00 | 0 | -1 |  | HE1 | HB1 |
| PRO507 | LYS355 | 7.69 | -0.04 | 0 | 1 |  | HD2 | HD2 |
| LEU455 | ALA37 | 7.72 | -0.01 | 0 | 0 |  | HD22 | H |
| ILE418 | VAL35 | 7.72 | -0.04 | 0 | 0 |  | HD1 | HG22 |
| PHE490 | VAL35 | 7.73 | -0.04 | 0 | 0 |  | O | HG11 |
| PHE486 | GLN102 | 7.73 | 0.00 | 0 | 0 |  | HE1 | HE21 |
| GLY502 | SER388 | 7.75 | 0.00 | 0 | 0 |  | HA2 | HB1 |
| TYR489 | ALA81 | 7.75 | -0.02 | 0 | 0 |  | HH | HA |
| ASN487 | ALA81 | 7.75 | -0.03 | 0 | 0 |  | OD1 | HA |
| ASN501 | GLU43 | 7.79 | -0.05 | 0 | -1 |  | HD22 | N |
| SER477 | ASP21 | 7.79 | 0.02 | 0 | 0 |  | HB2 | H2 |
| GLY496 | LEU46 | 7.79 | -0.02 | 0 | 0 |  | O | HD13 |
| TYR489 | ARG83 | 7.80 | -0.12 | 0 | 1 |  | OH | HD1 |
| TYR489 | GLU76 | 7.81 | 0.05 | 0 | -1 |  | HE1 | HG1 |
| PHE486 | SER78 | 7.82 | -0.04 | 0 | 0 |  | HE1 | O |
| TYR505 | THR326 | 7.82 | -0.01 | 0 | 0 |  | H | HG22 |
| ASN501 | PHE329 | 7.82 | -0.03 | 0 | 0 |  | O | H |
| VAL483 | ARG36 | 7.84 | 0.20 | 0 | 1 |  | C | HH12 |
| GLY485 | ARG83 | 7.84 | 0.17 | 0 | 1 |  | C | HD1 |
| TYR473 | GLN27 | 7.85 | -0.02 | 0 | 0 |  | HE2 | C |
| GLY502 | ASN332 | 7.85 | -0.01 | 0 | 0 |  | HA1 | HD21 |
| TYR505 | TYR387 | 7.85 | -0.04 | 0 | 0 |  | HE1 | O |
| TYR449 | GLU38 | 7.87 | -0.05 | 0 | -1 |  | HE1 | O |
| GLN506 | THR331 | 7.87 | -0.01 | 0 | 0 |  | HE22 | HG1 |
| LYS417 | PHE29 | 7.88 | -0.17 | 1 | 0 |  | HZ1 | N |
| GLN498 | ASN44 | 7.90 | -0.04 | 0 | 0 |  | HE22 | H |
| PHE497 | LEU46 | 7.92 | -0.01 | 0 | 0 |  | C | HD13 |
| TYR505 | ALA37 | 7.94 | 0.18 | 0 | 0 |  | HH | HB2 |
| LYS417 | LEU30 | 7.95 | 0.09 | 1 | 0 |  | HZ1 | C |
| PHE486 | ILE28 | 7.98 | -0.06 | 0 | 0 |  | C | HG23 |
| ASN487 | GLN24 | 7.99 | 0.00 | 0 | 0 |  | OD1 | O |
| GLN506 | ASN332 | 8.00 | 0.01 | 0 | 0 |  | HE21 | HD21 |
| ASN487 | PRO85 | 8.00 | -0.01 | 0 | 0 |  | HD22 | HD1 |
